# Which Genetics Variants in DNase-Seq Footprints Are More Likely to Alter Binding?

**DOI:** 10.1371/journal.pgen.1005875

**Published:** 2016-02-22

**Authors:** Gregory A. Moyerbrailean, Cynthia A. Kalita, Chris T. Harvey, Xiaoquan Wen, Francesca Luca, Roger Pique-Regi

**Affiliations:** 1 Center for Molecular Medicine and Genetics, Wayne State University, Detroit, Michigan, United States of America; 2 Department of Biostatistics, University of Michigan, Ann Arbor, Michigan, United States of America; 3 Department of Obstetrics and Gynecology, Wayne State University, Detroit, Michigan, United States of America; University of Chicago, UNITED STATES

## Abstract

Large experimental efforts are characterizing the regulatory genome, yet we are still missing a systematic definition of functional and silent genetic variants in non-coding regions. Here, we integrated DNaseI footprinting data with sequence-based transcription factor (TF) motif models to predict the impact of a genetic variant on TF binding across 153 tissues and 1,372 TF motifs. Each annotation we derived is specific for a cell-type condition or assay and is locally motif-driven. We found 5.8 million genetic variants in footprints, 66% of which are predicted by our model to affect TF binding. Comprehensive examination using allele-specific hypersensitivity (ASH) reveals that only the latter group consistently shows evidence for ASH (3,217 SNPs at 20% FDR), suggesting that most (97%) genetic variants in footprinted regulatory regions are indeed silent. Combining this information with GWAS data reveals that our annotation helps in computationally fine-mapping 86 SNPs in GWAS hit regions with at least a 2-fold increase in the posterior odds of picking the causal SNP. The rich meta information provided by the tissue-specificity and the identity of the putative TF binding site being affected also helps in identifying the underlying mechanism supporting the association. As an example, the enrichment for LDL level-associated SNPs is 9.1-fold higher among SNPs predicted to affect HNF4 binding sites than in a background model already including tissue-specific annotation.

## Author Summary

A large fraction of genetic variants that have been associated with complex traits are found outside of protein coding genes and likely affect gene regulation. Many experimental efforts have been dedicated to mapping regulatory regions in the genome but there are not many systematic methods that integrate functional data and regulatory sequences to predict the potential effect of any genetic variant on any given tissue and motif. Here we present a tissue and factor specific annotation that provides a predicted functional effect for both common and rare genetic variants. These predictions, certain of which are validated experimentally, show that the majority of genetic variants in gene regulatory regions are actually silent. Annotating those that are not silent allows us to investigate the molecular basis for the genetic architecture of many common traits and also to study the evolutionary properties that different types of regulatory sequences have across tissues or transcription factors. Overall, our study supports the concept that polygenic variation in binding sites for distinct classes of transcription factors has been a major target of evolutionary forces contributing to disease risk and complex trait variation in humans.

## Introduction

Despite large ongoing efforts to characterize regulatory regions in the human genome (e.g., ENCODE [1], Roadmap Epigenomics [2]), the lack of a regulatory genetic code to discriminate functional from silent non-coding variants in regulatory sequences poses severe limitations in interpreting the results of many human and population genetic analyses. For example, large numbers of genetic variants associated with disease and normal trait variation have been identified through genome-wide association studies (GWAS) [3]; yet a formidable challenge remains in determining the specific molecular mechanisms underlying association signals in non-coding regions. Similar challenges also arise when exploring the evolutionary functional significance of non-coding variants, for example through analysis of differences in genotype distribution across populations [4, 5]. This is also complicated by the fact that GWAS hits and signals of selection are usually found in large regions of association and do not directly pinpoint the true causative variants. In general, we do not know in which cell types/tissues these variants may have a functional impact.

Computationally and experimentally derived annotations for regulatory regions have been used to functionally characterize GWAS hits [1, 6–12]. However, a simple positional overlap between a genetic variant and regulatory regions is a necessary but not a sufficient condition to demonstrate an impact on TF binding. Many experimentally derived annotations are very useful to identify broad genomic regions across many cell-types, but lack the resolution necessary to pinpoint the regulatory sequences. High resolution functional assays like DNase-seq and ATAC-seq combined with computational methods that integrate sequence motif models [8, 9, 13, 14] can effectively dissect the regulatory elements; yet the motif models for transcription factor (TF) binding are generally not sufficiently well calibrated to predict the binding impact of a sequence change. Alternative ChIP based approaches (such as ChIP-seq and ChIP-exo), may provide increased TF and regulatory element specificity, but rely upon the availability of antibodies to target specific TFs or tagged TFs [15, 16]. The consequence is that we cannot provide a satisfactory answer to the following questions: Which genetic variants are more likely to impact binding of specific TFs? What is the fraction of genetic variants in regulatory regions that are not neutral? If we can adequately answer these questions, we may further ask: Did polygenic adaptation occur at binding sites for the same TF? Do variants in certain types of TF footprints and tissues contribute to variation in specific complex traits?

To help answer these questions, we have extended the CENTIPEDE approach to generate a catalog of regulatory sites and binding variants encompassing more than 600 experimental samples from the ENCODE and Roadmap Epigenomics projects with DNase-seq data, and recalibrated sequence motif models for more than 800 TFs. We then incorporated ASH information to provide additional empirical evidence, to validate the accuracy of the computational predictions and to estimate the fraction of genetic variants in regulatory regions that are not neutral. Importantly, our annotation is specific at the motif level (i.e., TF-specific) and at the sample level (i.e., tissue-specific). We then compare our results with the only alternative TF-centric annotation that has been recently published [17], but we also compare with non TF-centric SVM derived annotations [18]. Using our new catalog, we then examined genomic properties of the annotations, identifying characteristics that predict variants that disrupt binding, and demonstrated the action of natural selection on TF binding sites. Finally, we annotated and interpreted variants associated with complex traits, and we validated their allele-specific enhancer activity by reporter gene assays.

## Results

### Computational prediction of functional variants in regulatory sequences

**Fig 1:**
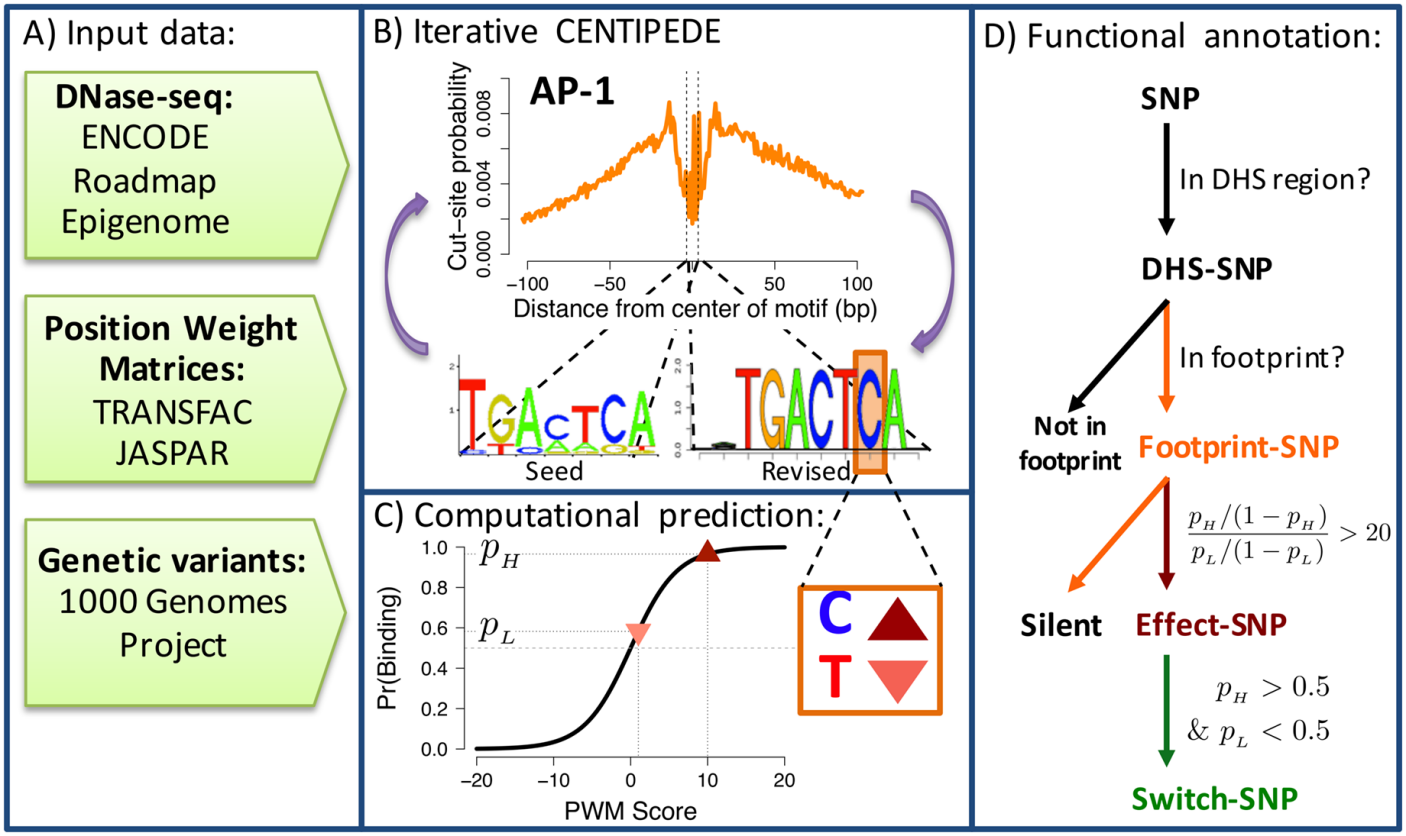
A visual description of the methods. (A) Data sources (B) Iterative process of using CENTIPEDE and seed sequence models (bottom left) to call footprints (top), then to revise the sequence models (bottom right), and call footprints again. (C) Computational predictions of genetic variant impact on factor binding. Conditional on a motif sequence match and observing a DNase-seq footprint a prediction is made using CENTIPEDE’s logistic model for the the prior probability of binding for each allele: *p*_*H*_ for the high binding allele (upward triangle), and *p*_*L*_ for the lower binding allele (downward triangle). (D) SNPs in non-coding regions are successively classified into nested categories base on being in a DHS, CENTIPEDE footprints and having a predicted functional impact on binding (based on the difference between *p*_*H*_ and *p*_*L*_.)

The CENTIPEDE approach allows to predict TF activity by integrating sequence motif models together with functional genomics data, and gains the most information from high-resolution data such as DNase-seq or ATAC-seq [19]. The spatial pattern in which reads are distributed, or footprint, is specific for each TF and can be very useful for discriminating between classes of TFs with distinct profiles [13]. In the original CENTIPEDE approach, the sequence models are pre-determined; e.g, k-mers or previously defined position weight matrix (PWM) models. However, many sequence models in existing databases were created with very few instances of known TF binding sites and do not represent the full spectrum of sequence variation that can be tolerated without affecting binding. Here, we have extended CENTIPEDE to readjust the sequence models for TF binding (Fig 1 and S1 Fig) using DNase-seq data and sequence orthologs (Methods). Compared to the original motif models the consensus sequence is largely maintained in the recalibrated motifs (S6 Fig). However, when we consider ChIP-seq peaks as validation we obtain superior precision recall characteristics (S7 Fig, Section 6.1 in S1 Text) and a much higher correlation with the prior probability of binding calculated by CENTIPEDE (S8 Fig, Section 6.2 in S1 Text).

Across all 653 DNase-seq samples, we identified a total of 6,993,953 non-overlapping footprints corresponding to 1,372 motifs active in at least one tissue and collectively spanning 4.15% of the genome. Each individual sample contained, on average, 280,000 non-overlapping footprints for 600 motifs and spanning 0.162% of the genome, indicating that footprints are highly tissue specific. Considering all SNPs from 1000 Genomes Project (1KG) at any allele frequency (even singletons), we found 5,810,227 (0.19% of the genome) unique genetic variants in active footprints (footprint-SNPs), 3,831,862 (66%) of which are predicted to alter the prior odds of binding ≥20-fold (effect-SNPs) based on the logistic sequence model hyperprior in the CENTIPEDE model (Fig 1C and 1D, Equation 2 in S1 Text). Effect-SNPs are further classified as switch-SNPs (264,965) if the allele flips the prior odds of binding. Importantly, in any of these categories we retain for each prediction the motif identity (TF-specific) and the underlying sample (cell-type specific) information.

### Allele-specific analysis confirms need for accurate prediction of function

These functional categories we computationally defined provide an answer to the question of which genetic variants in DNaseI sensitive regions are more likely to affect binding. To experimentally assess the accuracy of our answer, we used Quantitative Allele-Specific Analysis of Reads (QuASAR) [20] to perform joint genotyping and ASH analysis within DNase I hypersensitivity (DHS) regions (S2 Fig). While the initial quality filtering is the same as for the CENTIPEDE analysis, the parameters of the QuASAR model also allowed us to detect tissues with chromosomal abnormalities or samples from pooled individuals (Section 4.2 in S1 Text). These DNase-I samples were therefore excluded from ASH analysis (S9 and S10 Figs, S6 Table). Across the remaining 316 samples suitable for ASH analysis, we identified 204,757 heterozygous SNPs (hSNPs) in DHS sites (DHS-hSNPs) with coverage > 10x and with MAF > 0.05.

**Fig 2:**
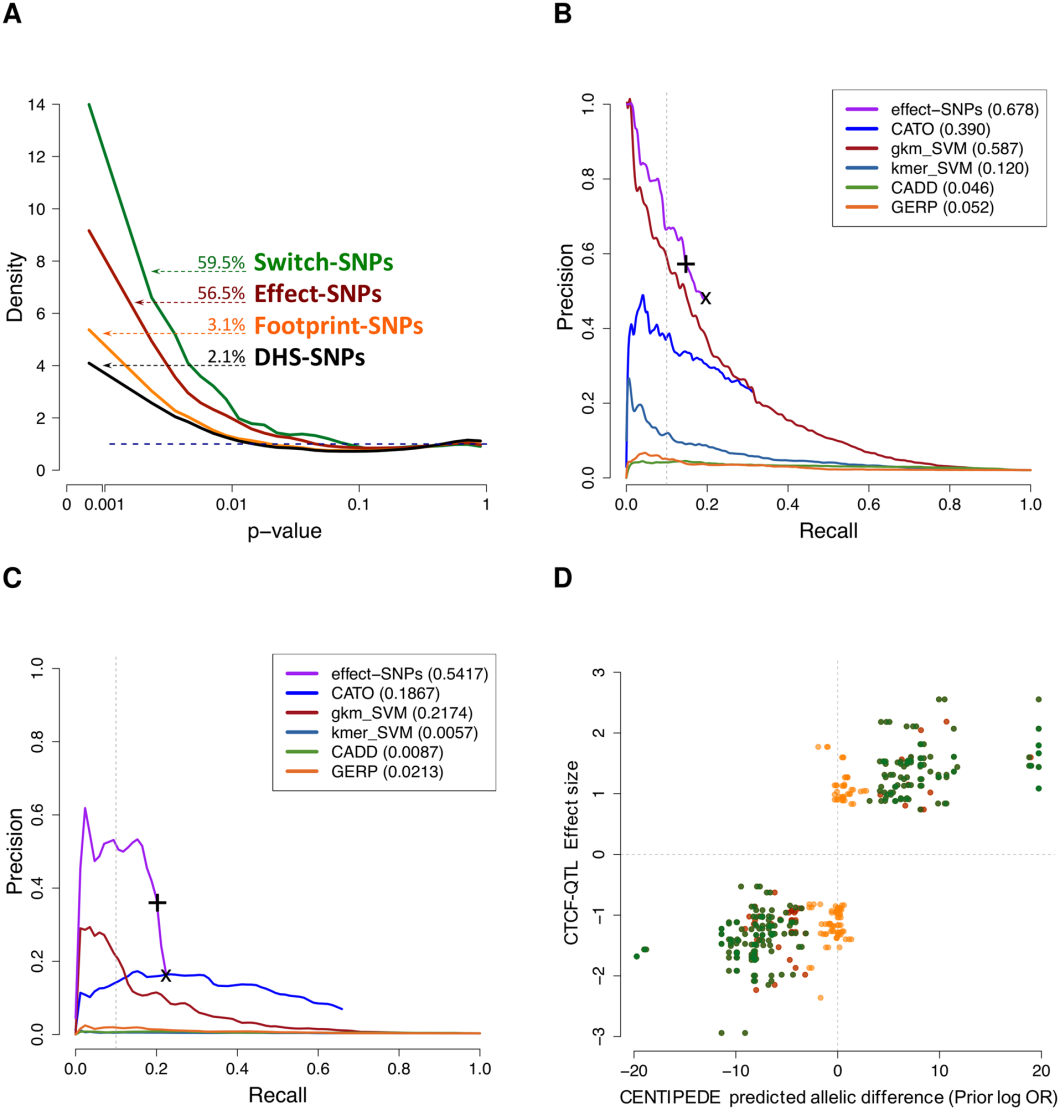
Determining which genetic variants affect TF binding. (A) ASH p-value densities for heterozygous SNPs in different categories (the dotted blue line represents the null distribution). Numbers shown are the estimated proportion of true signal, i.e., $1-{\hat{\pi }}_{0}$. (B & C) Precision versus Recall operating curve (PROC) comparing CENTIPEDE predictions to (B) dsQTLs (Degner *et al.*, 2012) [23] and (C) CTCF binding QTLs (Ding *et al.*, 2014) [24]. For our annotation (in purple), the line is drawn for different threshold on what is considered an effect-SNP, with the (x) indicating all footprint-SNPs and the (+) indicating the default threshold of 20x difference between alleles. (B) Except for CATO (Maurano *et al.*, 2015; dark blue) [17] and our annotation, the other prediction methods were already included in Lee *et al.* (2015) [18]. Note, the curve of some methods do not end at the lower-right corner because not all the dsQTLs have an annotation (e.g., if they are not in footprints). (C) For both CATO and effect-SNPs we only considered CTCF motifs, while for the methods that are not TF-centric all the scores are used. (D) Comparison of predicted binding effect for CTCF footprint-SNPs to CTCF-QTLs. Each dot represents a SNP within a CTCF binding region (ChIP-seq peak) and in a CENTIPEDE footprint with the same color annotation as in (A), the x-axis shows the predicted change in binding and the y-axis the QTL effect size for the alternate allele.

Overlapping our predictions with the DHS-hSNPs, 55,044 are footprint-hSNPs, 26,773 of these are effect-hSNPs, and 5,991 of these are switch-hSNPs. Overall, our computational predictions are highly concordant with the direction of ASH; 75% of the sequence models show positive correlation between the predicted and observed ASH (S11 Fig, S7 Table, Section 5.4 in S1 Text). Each of the nested SNP functional categories have marked differences in p-value distribution (Fig 2A) for the QuASAR test of ASH. Compared to what would be expected from the null uniform distribution, effect-hSNPs and switch-hSNPs have 8x and 14x times more SNPs with *p* < 0.001 respectively, showing that our functional annotations can predict ASH. Furthermore, these enrichments for lower p-values are much higher than those of DHS-hSNPs (4x) and footprint-hSNPs (6x), indicating that identifying SNPs in DHS regions and/or footprints alone is not enough to predict functional effects on binding. A similar observation can be made using the observed allelic ratios across CENTIPEDE annotations (S12 Fig). The result that SNPs that are just located in footprints or DHS regions tend to be silent is also true for other existing annotations (S13 Fig) or if we change the threshold for discriminating between footprints-SNPs and effect-SNP (S14 Fig). We also see that conservation score alone is not accurate enough to predict which SNPs have a functional impact on binding (S16 Fig).

To quantify the fraction of genetic variants that in each annotation will truly affect TF binding, we used ASH *p*-values as input evidence and followed the strategy of Benjamini *et al.* [21] to perform multiple testing correction in each category separately using Storey’s *q*-value procedure [22]. At an FDR threshold of 20%, we detected 3,217 unique hSNPs displaying significant ASH (Table 1), hereafter referred to as ASH-hSNPs. Taking into account LD (*R*^2^ < 0.8) these ASH-hSNPs constitute at least 3,158 independent loci. Several of the ASH-hSNPs were significant in more than one cell-type, giving a total of 4,940 observations of ASH-hSNPs across all samples. The 20% FDR threshold was chosen because this data was not originally sequenced to the depth that is generally required to call ASH at a single site with high confidence. In this reanalysis, we instead focus on the aggregate distribution of p-values to estimate the proportion of true null hypotheses (Storey’s procedure ${\hat{\pi }}_{0}$ estimate). We estimate that 56% of the effect-SNPs show evidence of ASH. While this conservative estimate can be considered a lower bound, it is still much higher than the estimates for DHS-SNPs (2.1%) and footprint-SNPs (3.1%), indicating that most SNPs in DHS regions and even in the putative binding sites do not affect binding.

**Table 1:** Summary of allele-specific hypersensitivity SNPs. Each row represents a category that is a subset of the category in the previous row. Each column reports the number of heterozygous SNPs, SNPs displaying significant ASH (20% FDR), and the estimated proportion of non-null hypotheses using Storey’s q-value approach. In parentheses are reported the numbers for SNPs that are not present in any of the subsequent subsets and are the basis for our partitioned q-value approach to detect ASH-hSNPs.

	# hSNPs	# ASH-hSNPs (20% FDR)	$1-{\hat{\pi }}_{0}$
All DHS-hSNPs	204,757 (179,137)	0 (0)	2.1 (1.7)%
Footprint-hSNPs	55,044 (42,098)	0 (0)	3.1 (0.3)%
Effect-hSNPs	26,773 (26,773)	3,217 (3,217)	56.5 (56.5)%

In addition to the DNase-seq ASH validation, we compared our annotations to the results of QTL analyses targeting DNase-seq sensitivity sites (dsQTLs, [23]), and CTCF binding sites from ChIP-seq [24]. For dsQTLs, using the same PROC analysis (see Fig 2B) as in [18] demonstrates that effect-SNPs have a good performance compared to SNPs identified using a SVM approach or CATO [17]. Note that we have not repeated the PROC analysis for the methods studied by [18], but we used directly the results provided by them, as PROC analysis could be sensitive to a redefinition of the underlying true labels of the set used to evaluate performance (see discussion in Section 7 in S1 Text). If we constrain the gk-SVM model to those predictions that overlap with our CENTIPEDE footprints, the precision (at 10% recall) improves to 80%. This indicates that SVMs are better sequence models than PWMs, but are not as specific without footprint information. To further investigate the TF-specificity accuracy of our annotations we used CTCF QTLs. CTCF is a very special type of TF with insulation [25], DNA loop organization [26], and barrier functions [27]. Compared to training an SVM on the DNase-seq data-set (non TF-centric), models that are TF-centric such as CATO and our effect-SNPs (integrating the footprint and sequence preferences) demonstrate a superior accuracy in discriminating dsQTLs that are also CTCF QTLs from those that may affect other factors (see Fig 2C). Among all CTCF footprint-SNP instances, all those that are also effect-SNPs are enriched for low CTCF QTL *p*-values and we predicted the correct direction (the allele with higher binding) in 100% of the cases (Fig 2D, Section 3.3 in S1 Text).

Some of the alternative methods include information such as conservation, distance to the TSS and allele frequency, however we have not included them in our annotation as we wanted to use those measures for analyzing the potential impact on organismal function and study differences among distinct TF motifs.

### Characterization of functional regulatory variants

**Fig 3:**
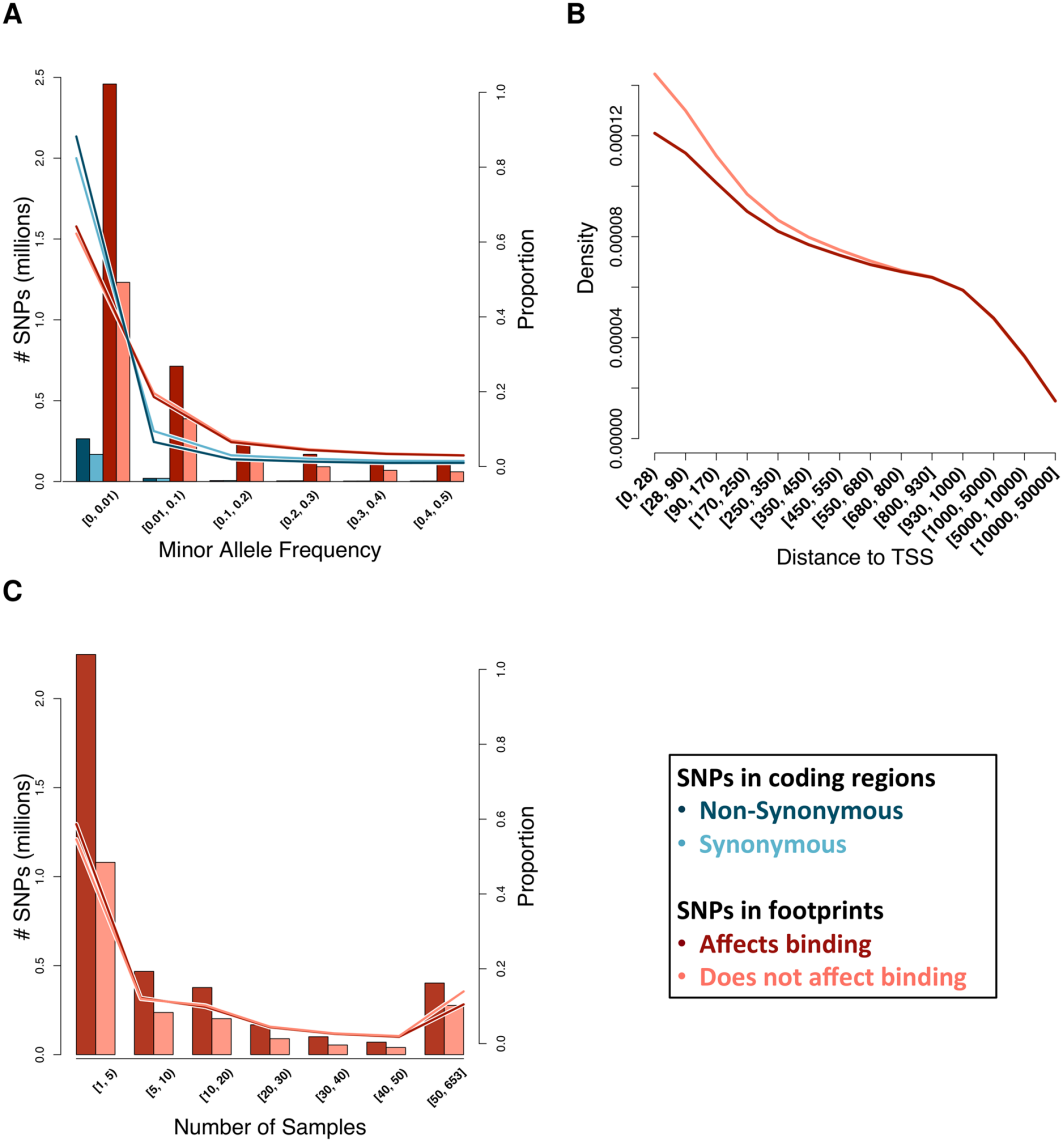
Characterization of SNPs in DNase I footprints. (A) Comparison of the minor allele frequency of SNPs predicted to affect binding or to be silent, showing both counts (bars) and proportions within SNP category (lines). Minor allele frequency at coding SNPs (from 1KG), separated into non-synonymous and synonymous, is shown for comparison. MAF is in bins of 10%, with the exception of rare (MAF < 1%) SNPs. (B) Proportion of SNPs at increasing distance from the nearest transcription start site (TSS) up to 50Kb. Distance is absolute distance, regardless of direction (up- or downstream) from TSS. (C) Stratification of footprint-SNPs by the number of tissues for which the footprint was predicted active, showing both counts (bars) and proportions within SNP category (lines). Number of tissues is binned by 5 or 10 until 50, where the remainder is binned.

Regions of the genome with demonstrated molecular function (e.g. genic regions) generally show reduced diversity [28] and a site frequency spectrum skewed towards rare variants. This is due to negative (purifying) selection, which prevents alleles from reaching high frequencies in the population if the molecular trait translates to a negative impact on organismal function. We investigated whether a similar skew in the site frequency spectrum exists at functional non-coding variants (effect-SNPs). We observed that effect-SNPs display an enrichment for rare variants (<0.5%) comparable to what it is observed in coding regions (Fig 3A), where rare variants are 1 to 2 times more likely to be non-synonymous changes than synonymous [29].

eQTL studies have found that variants associated with gene expression tend to occur close to the transcription start site (TSS) [30–33]. We detect a similar trend among our annotations, with 83% of footprint-SNPs occurring within 100kb of the TSS. However, we find a 1.12-fold depletion of effect-SNPs within 300 bases of a TSS (Fig 3B), which represents the core promoter region [34]. Effect-SNPs in this region are also enriched among rare variants (MAF < 0.001, 1.15-fold enrichment, Fisher’s test *p*-value = 6.027 × 10^−13^). This is likely because effect-SNPs in these regions have a major impact on regulatory processes that are shared across tissues. Accordingly, we also discovered a 1.18-fold enrichment for effect-SNPs in footprints active in 5 or fewer samples and a 1.38-fold depletion for effect-SNPs in footprints active in 50 or more samples (Fig 3C).

Since allele frequency can be correlated with distance to the TSS or sequence conservation, and shared footprints may also be more common at the promoter region, we tested several features (individually explored in Fig 3) in a joint model (Methods). All tested factors are significant predictors when considered together in a multiple regression logistic model, and the direction of the effect is the same as when they are considered separately (S8 Table). These results support the hypothesis that factors binding closer to the TSS and/or active in many tissues are housekeeping factors and those that recruit the transcriptional machinery and as a consequence are less likely to harbor common regulatory variants.

### Motif-wise characteristics of functional regulatory variants

**Fig 4:**
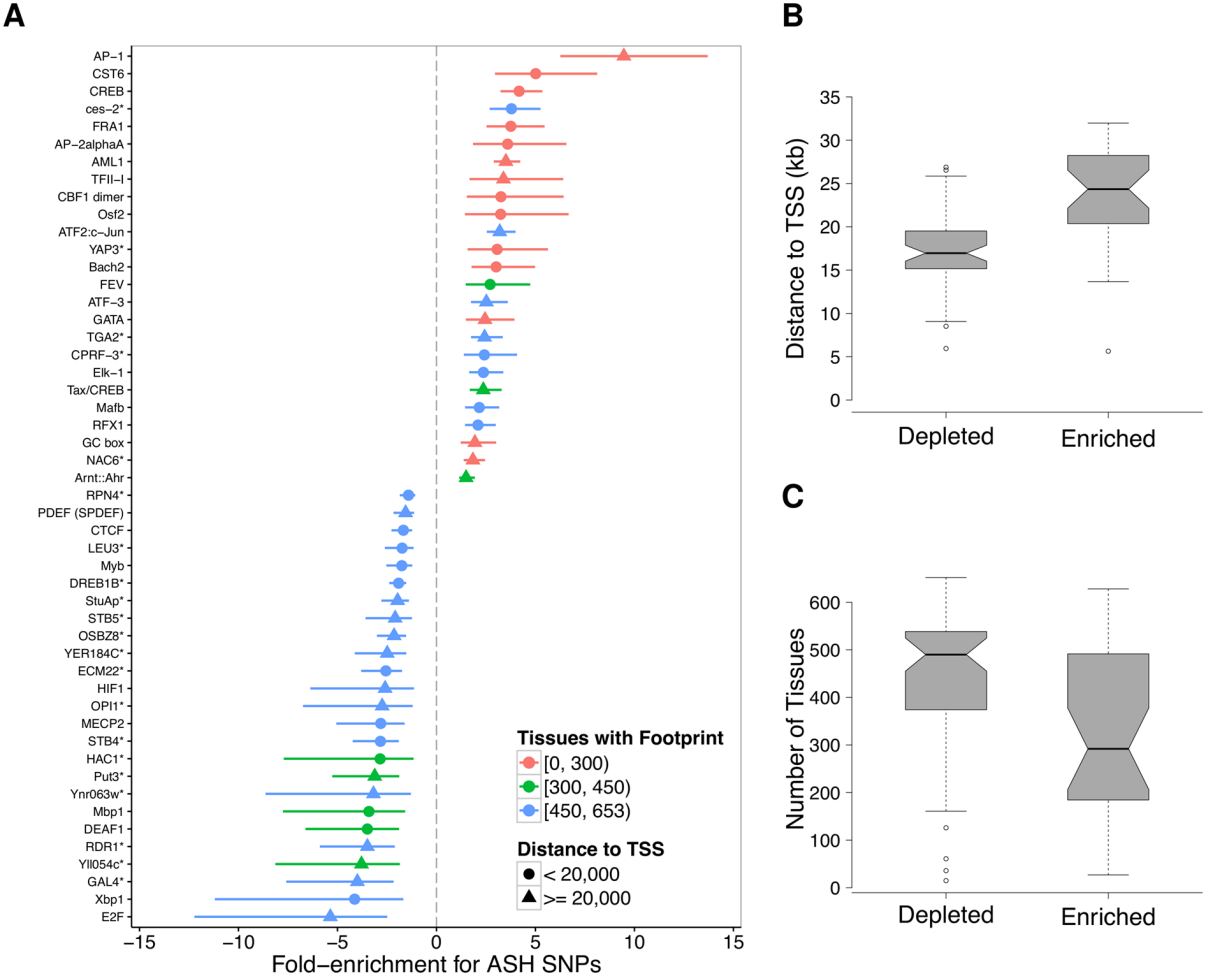
Motif-wise characteristics of functional regulatory variants. (A) Plot showing factors whose binding sites are significantly enriched (positive log_2_(fold change)) or depleted (negative log_2_(fold change)) for ASH-hSNPs, relative to the average number of ASH-hSNPs for a TF (p-value < 0.01), with indication of the number of tissues affected (color) and the median distance to the TSS (shape). Horizontal lines represent the 95% confidence interval of the ASH enrichment ratio. An asterisk denotes a possible human analog for the specified factor. Redundant motifs were excluded from the plot. (B & C) Boxplots showing the distance to the nearest TSS (B) and the number of tissues in which a motif was predicted to be active (C) for motifs either enriched or depleted for ASH-hSNPs. Notches on the boxplots are a non-parametric 95% CI interval on the median based on the inter-quartile range (IQR).

To examine the distribution of ASH-hSNPs across the different regulatory factors, we calculated the ASH enrichment ratio for each TF defined as the fraction of ASH-hSNPs over hSNPs relative to the average fraction across all TF (S17 Fig, Section 8.3 in S1 Text). At a nominal p-value cutoff of *p* < 0.01 (Binomial test), we detected 32 motifs enriched for ASH and 56 depleted for ASH (Fig 4A; S9 Table). In cases where multiple motifs correspond to the same factor, we observe similar enrichment for ASH-hSNPs (S10 Table), most notably for the factor AP-1, showing a >2.5-fold enrichment for ASH SNPs in all but one of the seven motif models. We see the same pattern for motifs significantly depleted of ASH-hSNPs, such as CTCF (1.5-fold median depletion) and E2F (1.8-fold median depletion). ASH enrichment ratios are also consistent across factors with similar functions. For example, three factors in addition to AP1 with roles in the immune response, CREB [35], c/EBP [36], and NF-*κ*B [37] are over 2-fold enriched for ASH-hSNPs within their binding sites (S11 Table).

We then examined the genomic characteristics at TF binding sites to identify features that distinguish motifs enriched for ASH versus those that are not. We found that motifs enriched for ASH are significantly farther from the TSS, having an average median distance to the TSS of 23kb compared to 17kb for those depleted (Mann-Whitney *p* = 3.2 × 10^−8^; Fig 4B). Furthermore, motifs enriched for ASH are active in significantly fewer samples, on average active in 20% vs 40% for those depleted (Mann-Whitney *p* = 1.9 × 10^−7^; Fig 4C), indicating that TFs with a high degree of ASH across their binding sites tend to be active in fewer tissues. This further confirms that changes in footprints active in a large number of tissues (constitutionally active) are more likely to have pleiotropic effects and therefore impact negatively the fitness of the organism and suggests polygenic mechanisms of evolution on motifs categories (i.e. groups of binding sites for a given TF or for TFs regulating genes with similar functions).

### Evidence for motif-wise selection in TF binding sites

**Fig 5:**
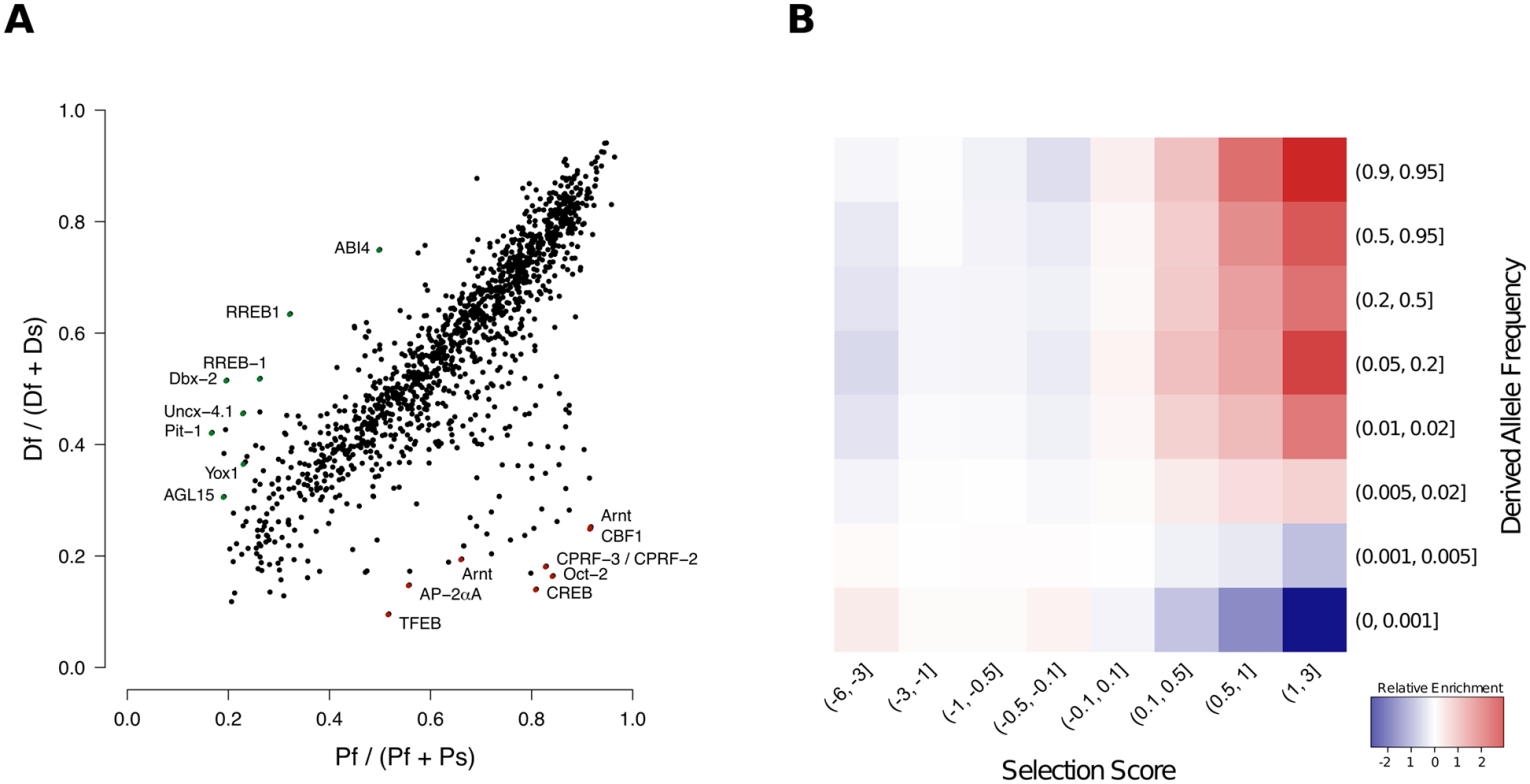
Examining selection on TF binding sites. (A) Comparison of fixed functional (D_*f*_) to fixed silent (D_*s*_) (y-axis) versus polymorphic functional (P_*f*_) to polymorphic silent (P_*s*_) (x-axis) variants across all of the binding sites for each TF examined. Scores towards the top left are suggestive of positive selection (excess of fixed functional changes) while scores towards the bottom right are suggestive of weak negative (purifying) selection. Several of the highest- and lowest-scoring factors are shown labeled with the corresponding TF. (B) Derived allele frequency for SNPs within TF binding sites. For each pairwise bin of of DAF (rows) and selection score (columns), the enrichment is defined as the ratio between the observed proportion of SNPs in that bin and the expected (i.e., the product of the two marginal probabilities).

An important question in evolutionary biology is the extent to which selection has acted on *cis*-regulatory elements in humans [38–41]. While methods are being developed to address this question [42, 43], such methods have only been applied to a narrow subset of TFs, and, in the case of [43], rely on RNA expression data to classify mutations as up- or downregulating transcription relative to the reference enhancer sequence. Given our categorization of footprint-SNPs relative to their effect on factor binding, we performed an initial survey of selection across TF binding sites using a test similar to the McDonald-Kreitman (MK) test [44] (S3 Fig, Section 8.4 in S1 Text). Applying our modified motif-wise MK test, we obtained a selection score for TF motifs with a sufficient number of binding sites (Fig 5A, S12 Table). At an FDR of 1%, we observe 84 factors whose binding sites are enriched for fixed functional differences (higher selection scores), suggestive of positive selection acting on those sites. Among the top scoring motifs are several factors that regulate neural and neuro-developmental processes, including POU1F1, PHOX2B, DBX2, UNCX, and YY1 which were not previously seen [42]. Among the factors with the lowest selection scores, we find ARNT, RBPJ, CREB1, POU2F2, and MYC which match with what has previously been observed [42]. While the interpretation of a positive selection score is generally that of positive selection, interpreting negative scores is more challenging. Generally, deleterious alleles are much less likely to reach fixation in populations than neutral alleles, however a negative selection score could also be explained by relaxation of selection or balancing selection. To identify the most likely evolutionary scenario for variation in binding motifs with negative selection scores, we calculated the derived allele frequency (DAF) for SNPs in binding sites. We observed an excess of rare alleles for SNPs in binding sites with a negative selection score (Fig 5B, S19 Fig, Section 8.5 in S1 Text), suggestive of weak purifying selection, rather than relaxation of selection (similar DAF spectrum across categories) or balancing selection (excess of intermediate frequency alleles).

We next asked whether the excess of functional polymorphism relative to functional divergence were influenced by background selection from nearby genes (S18 Fig), as functional regulatory variants may occur closer to the TSS, compared to silent variants. We find a mild but significant positive correlation between selection score and median TSS distance (Spearman *ρ* = 0.16, *p* = 5.6 × 10^−9^). Additionally, there is a negative correlation between tissue specificity and selection score (Spearman *ρ* = −0.20, *p* = 1.2 × 10^−13^). While some of the selection signal may come from nearby genes, there does appear to be a pattern of selective constraint on broadly active factors binding in promoter regions.

### Functional regulatory variants help identify and interpret causal GWAS hits

Given that our annotations comprise predicted functional effects across multiple cell-types/tissues and are anchored at footprints for known TF motifs, we asked if they could help interpret genomic hits reported in the GWAS catalog. We first considered a gross overlapping approach that considers each variant in a GWAS hit region equally likely to be causal (using an *r*^2^ cutoff of 0.8 from 1KG Project data, as in Ward *et al.* [10]). In GWAS hit regions, we compared the proportion of effect-SNPs over footprint-SNPs and found a moderate 1.11-fold enrichment for effect-SNPs (*p* < 2.2 × 10^−16^, 95% CI: 1.10—1.14). These moderate but statistically significant enrichments are typical of other annotations as well and are likely due to the fact that: i) we only consider the strongest GWAS hits (missing variants with moderate and small effects), ii) not all the factors and tissues may have the same enrichment, and iii) lack of resolution, as expanding the GWAS hit region makes the enrichment effects more moderate. Nevertheless, if we add our annotation to category 2 SNPs from the RegulomeDB [8] (SNPs with multiple regulatory annotations, but not yet shown to be functional), we detect a 1.6-fold enrichment for GWAS hits compared to category 2 SNPs alone (*p* = 6.11 × 10^−5^, 95% CI: 1.27—1.99). This result demonstrates that our annotation adds relevant information as it filters genetic variants not likely to be functional, but the overlap approach employed cannot take full advantage of the resolution and contextual information provided by our CENTIPEDE predictions.

**Fig 6:**
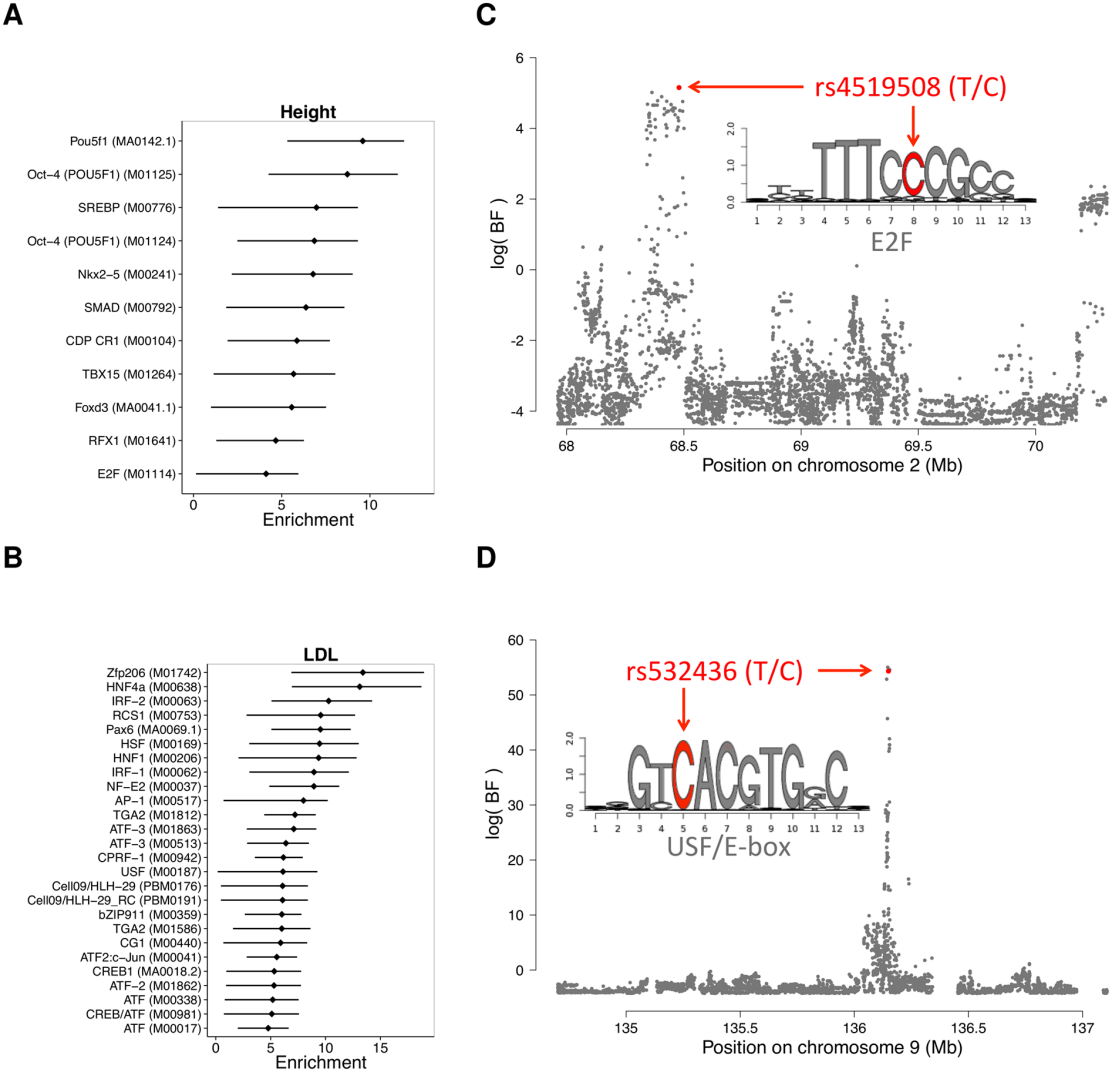
Integration of annotations into GWAS results. (A & B) Enrichment (*log*_2_(change in prior odds w.r.t the baseline model)) of factors for association with (A) height and (B) low-density lipoprotein levels. Error bars are drawn for 95% confidence intervals. (C & D) Association plots showing the Bayes factor of each SNP in the displayed region for (C) height and (D) low-density lipoprotein levels. Shown in red are SNPs with a posterior probability of association >0.4.

To better test if the annotated effect-SNPs can help fine-mapping and give a mechanistic support for variants associated with complex traits, we integrated them into GWAS meta analyses for 18 traits (see S13 Table) using the recently developed hierarchical model fgwas [45]. Importantly, in this analysis we used as input the association *p*-values measured or imputed to all known common variants in the genome. Furthermore, for each trait we compare to a baseline model [45] that considers previously defined annotations [11, 46] and confounders (e.g., distance to TSS, coding region, and others). For each trait, we identified factors whose binding sites were enriched for associated SNPs (Fig 6A and 6B, S20 Fig and S14 Table) over the baseline model (the enrichments reported by fgwas are log-odds ratios from the model parameters).

Overall, we observed high enrichments for biologically relevant factors. For example, the enrichment for effect-SNPs in OCT-4 (POU5F1, a TF with a key role in embryonic development and stem cell pluripotency [47]) regulatory sequences when considering genetic variants associated with human height is 6.6-fold higher (95%CI: 3.7-8.2) than in the baseline model. This is consistent with previous observations of genetic variants associated with height being enriched in embryonic stem cell DHS sites [48]. We also observed an enrichment for the developmental regulators TBX15 (3.9x), FOXD3 (3.9x), and NKX2-5 (4.7x) for genetic variants associated with height. From a study of low-density lipoprotein (LDL) levels in the blood, enriched factors include the liver-specific factor HNF4A (9.1x), as well as several regulators of immune function, including CREB1 (3.7x), IRF1 (6.2x), and IRF2 (7.1x).

**Fig 7:**
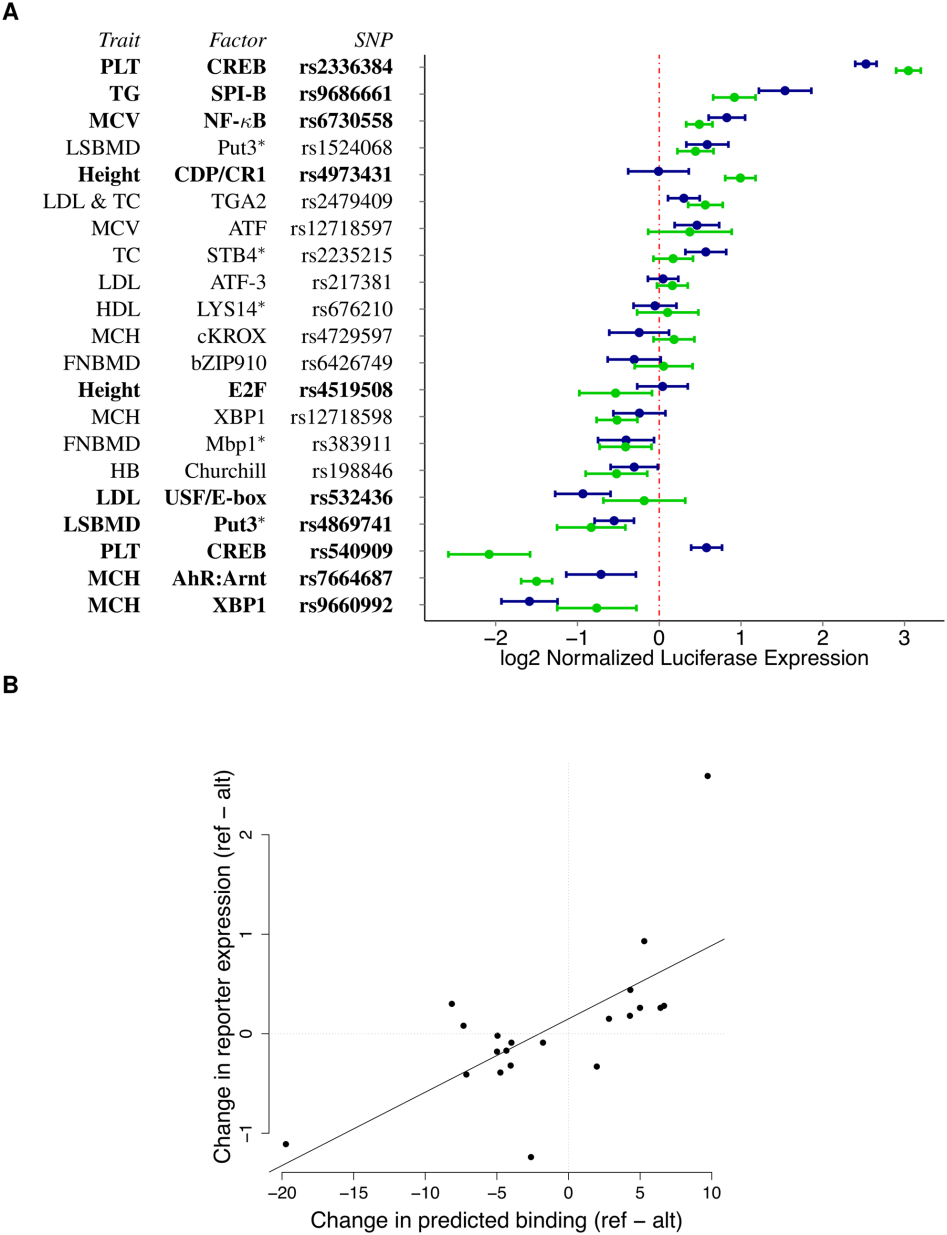
Reporter gene assay validation of allelic regulatory activity. (A) Average luciferase expression level for the constructs containing the reference (blue) and alternate (green) allele, normalized to the empty vector. SNPs with significant allele-specific effect on gene expression are listed in bold. *denotes human orthologs. (B) Change in predicted binding (prior log ratio from the sequence model, x-axis) versus normalized expression experimentally measured in the reporter assays (y-axis). The black line represents the best-fit line from a linear model fit on all points.

Our high resolution annotations allowed us to dissect the most likely functional variant (posterior probability of association, PPA > 0.2) in 88 previously identified GWAS regions (S15 Table, S23 Fig). For all 88 but 2 of these SNPs we have at least a 2-fold increase on the posterior odds of picking the potentially causal genetic variant according to fgwas (8.5x median fold increase) when compared to the comprehensive baseline annotation used by [45]. We then performed reporter gene assays for 21 SNPs to validate the predicted allelic effect on gene expression and the underlying molecular mechanism (Fig 7A and 7B, S16 Table, Methods). Among the regions tested we validated that 11 have enhancer/repressor activity and 10 have variants with allele-specific activity (*p* < 0.05, BH-FDR = 10%). This corresponds to 48% validation rate which is much greater than the 5% that would be expected by chance (Binomial test *p* = 2.01 × 10^−8^). Overall the predicted effect on binding and the change in gene expression are well correlated (Spearman *ρ* = 0.612, *p*-value = 0.0032), and the three SNPs with opposite effects may represent binding sites for repressors. Spearman correlation is robust to outliers, removing potential outlier rs540909 results in *ρ* = 0.657 (*p*-value = 0.002). We also achieve a similar correlation when we use our predictions to evaluate mutations in enhancers from a previously published reporter assay [49] that match our CENTIPEDE footprints (Spearman *ρ* = 0.76, *p*-value = 4.37 × 10^−5^, S22 Fig, Section 9.4 in S1 Text).

As an example, rs4519508, associated with a 2.1cm decrease in height [50], is in a binding site for the cell-cycle regulator family E2F (Fig 6D). Our annotation increased the PPA from a baseline of 10.5% to 44.4%, and it is the highest associated SNP in the association block (S21A Fig). This E2F footprint is active in >300 tissues (most of them fetal) and we detected ASH at this SNP in lung fibroblasts, validating that the reference allele at rs4519508 confers stronger binding than the alternate. Interestingly, in the reporter assay we observed 1.5-fold increased expression in the presence of the alternate allele, suggesting that at this location, E2F is acting as a repressor. Finally, this SNP is located within the promoter of PPP3R1, a regulatory subunit of calcineurin important for cardiac and skeletal muscle phenotypes; and a SNP in the same region has been shown to be associated with endurance [51] in humans. The p-value of association for this GWAS locus (*p* = 8.1 × 10^−6^) does not reach genome-wide significance in the height meta-analysis data we used [50]; however, in a recent more extensive meta-analysis for height [52] this locus achieves genome-wide significance *p* = 8.4 × 10^−10^, demonstrating that our annotation can be useful to rescue relevant loci.

Finally, a SNP associated with LDL levels, rs532436, is within a footprint for USF, an E-box motif (Fig 6C). Adding our annotation increased the PPA of the SNP from 39.7% to 94.7% (S21B Fig). We found that the alternate allele, associated with a 0.0785 mg/dL increase of LDL in the blood, is predicted to have a lower binding probability and results in 1.8-fold lower expression, compared to the reference allele. This SNP is identified by GTEx [53] as an eQTL for two proximal genes in whole blood: ABO (*p* = 5 × 10^−5^) and SLC2A6 (GLUT6, a class III glucose transport protein; *p* = 8 × 10^−5^). The SNP has an opposite effect on expression of the two genes, with the alternate allele showing lower expression for ABO and higher expression for SLC2A6.

These results show that our integrated analysis provides support for likely mechanisms linking regulatory sequence changes to complex organismal phenotypes. Furthermore, these mechanisms can be directly investigated through molecular studies, providing additional support that these sequence changes are truly functional.

## Discussion

We have developed an approach for assessing functional significance of non-coding genetic variants in DNase-seq footprints. Our strategy integrates sequence information with functional genomics data to predict the impact of single nucleotide changes on tissue-specific TF binding. This is achieved while integrating footprint information that preserves the identity of the underlying factor with high specificity. By borrowing data from ENCODE and Roadmap Epigenomics, we generated one of the most comprehensive catalogs available to date annotating regulatory regions and functional genetic variants across the genome.

Thus far, most common approaches for identifying regulatory variants from functional genomics data assume that each SNP in a regulatory region is equally likely to be functional. A key finding in this study is that genetic variants in active regulatory sequences, as defined by DNase I sensitivity and footprinting, are mostly silent; only 2.1% of SNPs in DHS regions and 3.1% of SNPs in CENTIPEDE footprints are estimated to have ASH. This is analogous to SNPs in coding regions, where most genetic changes are synonymous and do not result in an amino acid change [29]. The sequence model developed in this study provides a very useful filter for non-coding genetic variants that are not functional, resulting in a tissue-specific and motif-specific annotation of effect-SNPs (56.5% of which are estimated to have an impact on ASH). This is crucial information to take into account when we attempt to understand the molecular mechanism behind GWAS hits and evolutionary signals of selection. As additional functional genomics studies are performed, across larger sample sizes, tissue types and cellular conditions, it will be important to further determine the functional subset of regulatory variants within binding sites to achieve greater power in functionally annotating genetic variants associated with complex traits.

We find that genetic variants that are predicted to impact TF binding are depleted in the core promoter regions, exhibit higher sequence conservation in closely related species, tend to have low allele frequency and are enriched in tissue-specific footprints. These properties largely reflect the family-wise characteristics of motifs, which are further reflected in signals of selection. Future studies could incorporate tissue breath, conservation and distance to TSS as features to further filter effect-SNPs that may not show ASH. It should also be noted that our definition of functional regulatory variants is connected to the predicted effect on binding in the specific subset of cell-types/conditions that were available. Analyzing the allelic effects of non-coding variants in the context of other tissue types, conditions and functional genomic assays may potentially identify a functional role for some of the sites here defined as silent. In this study, we treated each TF separately, but future work should further explore the combinatorial grammar that different groups of motifs may define by cooperative binding to determine tissue specific binding sites. This will probably require more complex sequence models (e.g., SVMs [18, 54] or deep neural networks [55, 56]) than the PWMs used here. Here we show that the footprint information helps in predicting functional variants by further identifying the underlying TF compared to a sequence-fits-all model. More sophisticated footprint models [57] may also offer additional improvements to dissect the complexity of the regulatory grammar.

As not all genetic variants that have an impact on binding may lead to changes in gene expression and ultimately an organismal phenotype, combining these predictions with eQTL data across several tissues or environmental conditions would be important to further refine this annotation. As an example, Wen *et al.* [33], using an early release of this annotation in lymphoblastoid cell-lines demonstrates that effect-SNPs are 1.49 fold (with 95%CI[1.38, 1.63]) more likely than baseline SNPs (SNPs that are not located in a footprint) to be eQTLs (*p* = 4.93 × 10^−22^); in contrast, silent footprint-SNPs are 1.15 fold (with 95%CI[1.04, 1.27]) enriched in eQTLs, comparing to baseline SNPs (*p* = 0.0035).

A key feature of our annotation is that it spans a large collection of tissues and transcription factor motifs. This allowed us to trace some of the evolutionary history of TF binding and identify evolutionary constraints on specific molecular functions, which may reflect selective pressures during human history. For example, we observed that immune TFs are enriched for ASH sites, which supports the hypothesis that this may be a consequence of human adaptations to pathogen exposures [58]. On the other hand, we identified neural development TFs that may have undergone positive selection in humans. The large number of regulatory variants predicted in our study, together with previously reported eQTL signals [59–61], and the overall relevance that they have in explaining complex traits provide further support for polygenic models of complex traits in humans. By taking advantage of the factor-specific annotations in our study, we identified motifs that are enriched for regulatory variants associated with relevant GWAS traits and we provide examples of molecular mechanisms behind the association signals; e.g., immune TFs in the lipids study, and developmental TFs for height. Finally, we show how regulatory annotations improve the identification of potential causal SNPs in GWAS. Overall, the GWAS meta-analysis and selection signals in our study support the concept that polygenic variation in binding sites has been a major target of evolutionary forces and a key contributor to disease risk and complex phenotypes in human populations.

## Methods

### Identification of active regulatory sites and motif recalibration

We used 1,949 PWM sequence models (motifs) from the TRANSFAC [62] and JASPAR [63] databases to scan the genome for a set of representative motif matches (Section 3.1 in S1 Text). For each motif, we used the matching sequences to calculate a new PWM model which we then used to scan the genome and identify all genome-wide motif matches using a two step approach:

Step 1: Initial CENTIPEDE scan and motif recalibration. For each motif, we extracted DNase-seq data at sequence matches across 653 samples (corresponding to 153 unique tissues) publicly available from the ENCODE and Roadmap Epigenomics projects (Sections 1 and 2.1 in S1 Text). The motifs and samples used are summarized in S1 and S2 Tables. For each motif and only for this initial step, we used a reduced subset of motif matches that include the top 5,000 best sequence matches, and up to 10,000 additional low-scoring sequences (Section 3.1 in S1 Text, note that for Step 2 we will use all motif matches in the genome). To avoid overfitting and to heuristically reduce the search space, these low scoring motif instances are human sequences that have orthologous very high scoring motif instances in the chimp or rhesus genome. We then applied the CENTIPEDE model to survey TF activity for each 1,272,697 tissue-TF pair. For each pair we then determined that the TF is active if the sequence matches that exhibit a CENTIPEDE footprint can be predicted from the PWM score (Z-score > 5, S4 and S5 Figs). Using this criterion, we determined that 1,891 TF motifs are active in at least one tissue. The full list of motifs active in each tissue can be found in S3 Table. We then recalibrated the PWM model for each active motif using the sequences of all motif matches that have a DNase-seq footprint (CENTIPEDE posterior > 0.99).

Step 2: Full genome CENTIPEDE scan and genetic variant analysis. Using the recalibrated sequence models we scanned the human genome again for all possible sequence matches. We used the CENTIPEDE algorithm to assess the probability that each motif instance is bound by a TF, both to the reference and to alternate alleles when the match contained a genetic variant catalogued in the 1KG Project [29]. In this second step, we included all high and low scoring PWM matches down to the threshold corresponding to a CENTIPEDE prior probability of binding of 10% (Equation 2 and Section 3.2 in S1 Text).

### ChIP-seq validation of the revised sequence motif models

To evaluate whether the updated sequence models derived from DNase-seq data are better at predicting TF binding than the original seed motifs, we compared to ChIP-seq data available for a small set of TFs from the ENCODE project (as these data are generated in independent experimental assays that should be highly TF-specific). Using precision recall operating characteristic (P-ROC) curve analysis (see Section 6.1 in S1 Text), we determined that for a given precision (precision = 1—FDR, false discovery rate), the updated sequence models have higher recall (sensitivity) than the original PWM in detecting ChIP-seq peaks (S7 Fig). Additionally, we compared the correlation between the prior probability of binding (calculated by CENTIPEDE based on the PWMs) and the number of ChIP-seq reads overlapping motif matches (S8 Fig, Section 6.2 in S1 Text).

### Categorization of footprint-SNPs based on predicted functional impact

We classified a SNP in a CENTIPEDE footprint (footprint-SNP) as having a predicted effect on binding (effect-SNP) if the difference in the prior log odds ratio (from the logistic sequence model in CENTIPEDE, Equation 2 in S1 Text) between the two alleles was ≥3, indicating a ≥20-fold change in the prior odds of TF binding. We further classified an effect-SNP as switching the likelihood of binding (switch-SNP) if the prior log odds ratio flips; i.e, if it is ≥0 for one allele and ≤0 for the other. To generate a final set of annotated SNPs, we aggregated the data from each sample and motif into one table. For cases where a SNP is within multiple predicted binding sites, we selected the factor whose CENTIPEDE likelihood ratio was the greatest, i.e., the factor most likely to be binding at that location.

### Identification of allele-specific hypersensitivity (ASH)

Starting from raw sequencing reads, we used a custom mapper [23] to align the reads to the hg19 reference genome. As allele-specific analysis is extremely sensitive to mapping errors and PCR duplicates, we employed several methods to reduce these sources of potential bias (Sections 2.2—2.4 in S1 Text). To detect allele-specific hypersensitivity, we applied QuASAR [20] to the processed read data to infer genotypes for all 1KG SNPs and determine the likelihood of allelic imbalance at heterozygous sites. Note that we only test a SNP with QuASAR if it is covered by ≥10 reads. To adjust for multiple testing, we used the *q*-value method [22] on the *p*-values produced by QuASAR.

### Validation of predicted effect-SNPs using ASH-hSNP integrated analysis

We overlapped heterozygous SNPs (DHS-hSNPs) identified by QuASAR with CENTIPEDE footprints-SNPs and effect-SNPs catalogued for each sample. SNPs were then partitioned based on their predicted effect on binding into three non-overlapping categories: 1) hSNPs in predicted footprints whose binding effect is in the direction predicted, 2) all other hSNPs in footprints, 3) all other DHS-hSNPs. Because each annotation has a different prior expectation of being functional, we re-adjusted for multiple testing within each annotation separately using the *q*-value method [22] on *p*-values produced by the QuASAR model. We denote as ASH-hSNPs those hSNPs with a *q*-value < 20% in any of the partitions.

### Regression model for binding effect

To determine which features of a SNP are predictors of functional effect, we performed multiple regression analysis using a logistic model considering the dependent binary variable *E*_*l*_, indicating whether the footprint-SNP, *l*, is also an effect-SNP.

$$\begin{array}{c} \text{logit}\left(E_{l}\right)∼C_{l}+F_{l}+T_{l}+N_{l}+P_{l} \end{array}$$

We considered the following variables related to the probability of a footprint-SNP being an effect-SNP: the footprint likelihood ratio (without the sequence model) (*C*_*l*_); the minor allele frequency (*F*_*l*_); the absolute distance to the nearest transcription start site (*T*_*l*_); the number of tissues for which the motif containing the footprint-SNP was predicted to be bound (*N*_*l*_); the phyloP conservation scores calculated from primates (*P*_*l*_).

This model does not evaluate the sequence, rather it combines the results shown separately in Fig 2 into a single model to characterize the predictions made by CENTIPDE. The model was fit using the GLM function in R. The result of this regression analysis can be seen in S8 Table.

### Identification of selection signals on TF motifs

To identify divergent TF binding sites, we used the UCSC liftOver tool on binding sites without a known polymorphism to obtain orthologous regions in the chimpanzee genome. Using the PWM model, we calculated PWM scores and CENTIPEDE prior probabilities of binding on the chimpanzee sequences. Sites with a sequence change in the motif instance (prior probability of binding differs from the humans sites) were classified as divergent, and were further categorized by the difference in binding affinity: “functional” for sites that change ≥20-fold between species (analogous to effect-SNPs), and “silent” for those that do not. For the binding sites containing a polymorphism, we used the definition of effect-SNPs to identify functional for silent sites and footprint -SNPs for silent sites. For each factor motif, we then calculated the number of binding sites belonging to each of the four categories (divergent functional, divergent silent, polymorphic functional, and polymorphic silent) and calculated a selection score similar to the McDonald-Kreitman test (Section 8.4 in S1 Text).

### Integrating high-resolution functional annotations with GWAS and fine-mapping

To integrate functional annotations and GWAS results, we used the fgwas command line tool [45]. fgwas computes association statistics genome wide using all common SNPs from European populations in the 1KG Project, splitting the genome into blocks larger than LD. Summary statistics were imputed with ImpG using *Z*-scores from meta-analysis data. Using an empirical Bayesian framework implemented in the fgwas software, GWAS data were then combined with functional annotations. We then compared the informativeness of these annotations from each of the 1891 motifs with CENTIPEDE predicted regulatory sites to a baseline model (see Section 9.2 in S1 Text) consisting of previously used genomic annotations identified as relevant [45]. For each locus that contains at least one SNP with a PPA > 0.2, we only consider the SNP with the highest *p*-value or PPA from fgwas. Rather than look at a credible set, we pick a single SNP most likely to be causal and see if that SNP has a higher PPA with the annotation than without it. While reduction in size of the credible set is very important for assessing fine-mapping methodologies, here our focus is on combining annotations to identify the single most likely causal SNP per GWAS locus.

### Validation of GWAS-relevant effect-SNPs

GWAS-relevant effect-SNPs located in active footprints in LCLs (the cell line used for transfection) were ranked on the Spearman correlation coefficient in S7 Table. We initially selected the top 25 SNPs with a positive correlation, but the assays for 4 of them failed for several technical reasons (e.g., cloning step failed). To test allele-specific effects on expression for the remaining 21 SNPs, we first constructed inserts containing the reference or alternate allele for each SNP of interest (see Section 9.3 in S1 Text). Cloning of these inserts in the pGL4.23 vector was performed using the Infusion Cloning HD kit (Clontech) and DNA was extracted using the PureYield kit (Promega). Transfections were performed into GM18507 using the standard protocol for the Nucleofector electroporation (Lonza). Luciferase activity was measured for up to 20 replicate experiments using the Dual-Glo Luciferase Assay Kit (Promega). We contrasted the activity of each construct to the pGL4.23 vector, to assess enhancer/repressor activity of each region. To evaluate allele-specific effects, we contrasted the activity of the reference allele to the alternate allele for each region and we used a t-test to assess significance at a *p* < 0.05 threshold. We used the Benjamini-Hochberg [64] procedure to assess FDR across all 21 SNPs tested.

### Enrichment analyses

Unless otherwise noted, tests for enrichment on two-way categorical variables are based on Fisher’s exact test. Tests involving multiple categorical, discrete or continuous variables use a logistic regression model and Wald’s test on each enrichment parameter, and are identified as such.

### Data availability

The generated annotation files are available as supplementary tables and at http://genome.grid.wayne.edu/centisnps/. All other relevant data are available in the manuscript and its Supporting Information files.

## Supporting Information

S1 Text Supplemental Materials and Methods. This text provides more detailed explanations of how experiments and analyses were performed, arranged into the following sections: (1) Data sources, (2) Data Preprocessing, (3) Identification and mapping of active transcription factors, (4) Analysis of allele-specific hypersensitivity, (5) Annotation of ASH with binding predictions, (6) Evaluation of recalibrated sequence models, (7) Precision versus recall analysis using DNase-seq and CTCF QTLs, (8) Genomic annotation and selection signals, (9) Overlap with genome-wide association studies.
(PDF)

S1 File Full catalog of SNPs in footprints. Each row is a specific SNP / TF motif / cell type combination. Columns 1-3, bed-formatted SNP position (0-based); 4, motif ID; 5, log ratio between the prior log odds of binding for each allele; 6, prior log odds of binding for the reference allele; 7 prior log odds of binding for the alternate allele, 8 cell type.
(BGZ)

S2 File Recalibrated motif position weight matrices. (GZ)

S3 File Footprint profiles of recalibrated motif position weight matrices. For each motif, footprint profiles are aggregated across all binding sites in all 653 DNase-seq samples. Color indicates which strand the motif matches, positive (blue) or negative (red). Text in the upper left denotes the tissue with the highest Z-score from the CENTIPEDE mode, the motif ID, and the corresponding transcription factor.
(GZ) Click here for additional data file.

S4 File SNPs with significant ASH. Each row is a specific ASH-hSNP / TF motif / cell type combination for which the ASH-hSNP displays significant allelic imbalance. Columns 1-3, bed-formatted SNP position (0-based); 4, rsID; 5-6, reference and alternate alleles; 7-8 reference and alternate read counts; 9-10, reference and alternate Pr(binding) from CENTIPEDE; 11, cell type; 12 motif ID.
(GZ) Click here for additional data file.

S1 Table DNase samples and sources. Listed for each sample is the source, the sample, and the number of reads.
(XLSX)

S2 Table Sources of additional data used in analyses. Download dates and, where applicable, specific cell-types/tissues are also listed.
(XLSX)

S3 Table Active motifs in each sample. For each sample, motifs were determined active if the Z-score, obtained from Equation 2 in S1 Text, was > 5, and if the motif instances showed correlation with DHS peaks (Section 3.2 in S1 Text).
(XLSX)

S4 Table Comparison of ASH within footprints between PWM models. Shown is the number of ASH-hSNPs within footprints identified by the two sets of PWM sequence models. The counts are stratified by p-value from the QuASAR test of ASH. Note that the old models, by default, only select sites with a PWM score > 12; for comparison, the same constraint has been placed on the sequences used from the new models.
(XLSX)

S5 Table Validation of genotype predictions. A comparison of 1KG genotypes and those called by QuASAR for the 12,650 loci examined in the LCL GM12878.
(XLSX)

S6 Table Summary of post-processing filters. The first three rows show the threshold and number of samples filtered for each parameter independently. After applying the three filters, the remaining samples were manually examined and known cancer samples were removed.
(XLSX)

S7 Table Motif-wide correlation between CENTIPEDE and ASH results. For each motif, CENTIPEDE predictions were compared to ASH data using 1) Spearman correlation and 2) a logistic model using the functional predictions to predict the ASH.
(XLSX)

S8 Table Predictiveness of genomic characteristics on functional effects. We considered the following characteristics in a regression analysis to determine their predictiveness as to whether a footprint-SNP is also an effect-SNP.
(XLSX)

S9 Table Enrichment of ASH-hSNPs within binding sites. Factors with at least 100 heterozygotes in a predicted binding site are listed along with the counts, ratios, and enrichments of ASH-hSNPs, footprint-SNPs, and switch-SNPs within them.
(XLSX)

S10 Table Comparison of multiple motifs for a single factor. Motifs corresponding to the same transcription factor are similarly enriched or depleted for ASH-hSNPs.
(XLSX)

S11 Table ASH effects for several immune-related factors. For each factor listed, we calculated the aggregate ASH enrichment ratio across all sequence models corresponding to that factor.
(XLSX)

S12 Table Selection score for individual motifs. For each factor motif, we used a modified MK test to calculated a selection score. Shown for each motif is the number of binding sites belonging to each category used in the MK test (divergent functional, divergent silent, polymorphic functional, and polymorphic silent) as well as the score.
(XLSX)

S13 Table Summary of GWAS meta analysis traits examined. Shown for each trait is the trait abbreviation and the citation for the meta analysis study.
(XLSX)

S14 Table Factor binding sites enriched for GWAS SNPs. For each trait, factors whose binding sites are enriched for SNPs associated with the trait are listed. Shown also are the lower and upper limits of the 95% confidence interval.
(XLSX)

S15 Table SNPs associated with GWAS traits that fall in CENTIPEDE-predicted TF binding sites. PPA, Posterior probability of association estimated by fgwas for each SNP. “Before” indicates the PPA from the base model, “after” indicates the PPA after adding footprint annotations to the model. The p-values listed are derived from the z-scores that are used as input for fgwas.
(XLSX)

S16 Table Reporter gene assay results. For each of the SNPs tested, listed are the results for the reference allele (top) and the alternate allele (bottom). Shown is the average and standard error (across replicates) of the firefly luciferase activity normalized to the renilla luciferase activity, for each construct (Norm Expr) and for the pGL4.23 vector (Empty Vector). The last two columns are the *t*-test *p*-values comparing the activity of the reference allele to the alternate allele (vs ref), and of each allele to the pGL4.23 vector (vs empty). Underlined alleles indicate the allele predicted to have stronger binding.
(XLSX)

S1 Fig Flowchart detailing steps of the CENTIPEDE-based annotation of regulatory regions and variants. Numbers next to boxes refer to the corresponding section in the Supplement.
(PDF)

S2 Fig Flowchart detailing ASH analysis pipeline. Numbers next to boxes refer to the corresponding section in the Supplement.
(PDF)

S3 Fig Flowchart detailing analysis pipeline for identifying selection across TFBS. “Prior Odds Ratio > 20” is the same criteria as the one used to define effect-SNPs. Numbers next to boxes refer to the corresponding section in the Supplement.
(PDF)

S4 Fig Binding profiles of AP-1 motif M00172. Footprint profiles are aggregated across all binding sites in all 653 DNase-seq samples, and stratified by Z-score (color). The higher the Z-score, the more likely a factor is bound as predicted by the CENTIPEDE model.
(PDF)

S5 Fig Distribution of Z-scores across samples and motifs. Shown is the full distribution of Z-scores (calculated with Equation 2 in S1 Text) across every sample-motif pair. The dotted vertical line at Z = 5 shows the selected threshold for factor activity.
(PDF)

S6 Fig Comparison between seed and revised sequence model. For each factor motif, shown is the original seed sequence model (left) and the revised model (right). x-axis: position within motif, y-axis: information content. (A) NRSF (B) CTCF (C) PU.1 (D) AP-1.
(PDF)

S7 Fig Precision-recall curves for seed (blue) and revised (black) sequence models. For each TF binding motif, CENTIPEDE-predicted footprints in GM12878 cells were compared using ENCODE ChIP-seq data as a gold standard. (A & B) CTCF (C & D) GABP (E & F) NRSF (G & H) PU.1.
(PDF)

S8 Fig Comparison of prior Pr(binding) derived from PWM scores to ChIP-seq read data across all motif matches using seed (blue) and revised (black) sequence models. Due to thresholds on the match score (see Section 3.2 in S1 Text), few models have data Pr(binding) < 0.2. For ease of display data is binned in 10% increments. Points represent the average number of ChIP-seq reads within that bin and vertical lines represent the 95% confidence interval. Spearman correlation (legend) is calculated using the full data set without binning. (A & B) CTCF (C & D) NRSF (E & F) PU.1.
(PDF)

S9 Fig Reference allele ratio *ρ* at 1KG variants. (A) Plot showing *ρ* allele ratios for SNPs interrogated for CD34 primary cells (used for ASH analysis). Three peaks on the histogram (right) correspond to homozygous reference (top), heterozygous (middle), and homozygous alternate (bottom) SNPs. (B) Plot showing *ρ* allele ratios for SNPs interrogated for the cancer line K562 (discarded for ASH analysis). Signatures of chromosomal abnormalities are evident from the scatterplot, such as copy number variation and loss of heterozygosity.
(PDF)

S10 Fig Distribution of values used for post-ASH analysis filter criteria. On all four panels *y*-axis represents the parameter M that is reciprocally related to the dispersion of *rho* in the QuASAR model. Dotted lines represent values used to filter samples. (A) Dispersion and correlation between *ρ* and *ϕ* (B) Dispersion and *ρ* estimation. Bottom plots show zoomed view of samples with *M* < 100.
(PDF)

S11 Fig Correlation between CENTIPEDE predictions and observed ASH. SNPs identified in both the CENTIPEDE and ASH analysis are shown, shaded by p-value of allelic imbalance from QuASAR. Points circled in red display significant ASH at 20% FDR. The blue line is a logistic curve fit using points with a *p* < 0.1.
(PDF)

S12 Fig Magnitude of allelic imbalance within predicted functional annotations. Each line represents a density plot of the magnitude of allelic imbalance ∣(allele ratio—0.5)∣ for SNPs within each functional annotation.
(PDF)

S13 Fig ASH p-value densities for different SNP categories. Shown are three additional categories of SNPs from recent studies of functional variation within TF binding sites.
(PDF)

S14 Fig Comparison of thresholds for functional annotation categories. ASH p-value densities for different SNP categories. For effect-SNPs and switch-SNPs, shown are different thresholds used for defining the category (20x is the threshold used throughout this analysis). The dotted blue line represents the null distribution.
(PDF)

S15 Fig Identification of ASH using only PWM score. ASH p-value densities for different SNP categories and PWM (sequence match) match scores. Numbers in parentheses are the number of SNPs in those categories. The dotted blue line represents the null distribution.
(PDF)

S16 Fig Identification of ASH using phyloP conservation score. ASH p-value densities for different SNP categories and SNPs with indicated phyloP conservation scores. Numbers in parentheses are the number of SNPs in those categories. The dotted blue line represents the null distribution.
(PDF)

S17 Fig Distribution of ASH enrichment ratios. For all motifs with > 100 hSNPs, an ASH enrichment ratio was calculated as # ASH-hSNPs (20% FDR) / # hSNPs across all binding sites genome-wide. The black line shows the average ratio across all motifs. Several factors whose binding sites are highly enriched or depleted for ASH-hSNPs are labeled.
(PDF)

S18 Fig Identifying selection signals in TF binding sites. (A) Density plot showing the distribution of selection scores from the modified MK test. (B) Comparison of selection scores to the number of tissues each factor is predicted to be active in. (C) Comparison of selection scores to the median distance to the TSS across all sites for a given factor.
(PDF)

S19 Fig Derived allele frequency and selection score. Shown are the relative enrichments for each DAF/selection score bin, for all variants (A) and for singletons and doubletons (B).
(PDF)

S20 Fig Enrichment of transcription factors motifs from fgwas. Shown are the *log*_2_(enrichment) values with 95% confidence intervals for each factor whose binding sites are enriched for SNPs associated with the traits in S14 Table. x-axis is truncated at 10 for ease of display.
(PDF)

S21 Fig Association plots identifying SNPs in footprints. Log Bayes factor (top) and posterior probabilities (bottom) of association to the indicated trait for all genetic variants in the regions containing rs4519508 and rs532436.
(PDF)

S22 Fig Correlation of CENTIPEDE predictions and mutated enhancers in HepG2 and K562 cells. For each point, plotted is the difference in the change in probability of binding (mutated prior log ratio—reference prior log ratio, x-axis) versus the *log*_2_(fold change) between mutated and wild type reporter constructs (y-axis). The black line represents the best-fit line from a linear model fit on all 22 points.
(PDF)

S23 Fig Association plots identifying SNPs in footprints from fgwas. For each SNP in S13 Table, two plots show the log Bayes factor (top) and posterior probabilities (bottom) of association to the indicated trait for all genetic variants in the region containing the SNP.
(PDF)
