# Supplementary material for: Which Genetics Variants in DNase-Seq Footprints Are More Likely to Alter Binding?
Source: PLoS Genet. 2016 Feb 22;12(2):e1005875. doi: 10.1371/journal.pgen.1005875 (PMC4764260; doi:10.1371/journal.pgen.1005875)
Supplement: S1 Text — This text provides more detailed explanations of how experiments and analyses were performed, arranged into the following sections: (1) Data sources, (2) Data Preprocessing, (3) Identification and mapping of active transcription factors, (4) Analysis of allele-specific hypersensitivity, (5) Annotation of ASH with binding predictions, (6) Evaluation of recalibrated sequence models, (7) Precision versus recall analysis using DNase-seq and CTCF QTLs, (8) Genomic annotation and selection signals, (9) Overlap with genome-wide association studies. (PDF) [file pgen.1005875.s001.pdf]

Supplemental Material for: Which genetic variants in DNase-seq footprints are more likely to alter binding?

Gregory A. Moyerbrailean[1], Cynthia A. Kalita[1], Chris T. Harvey[1],
Xiaoquan Wen[2], Francesca Luca[1,3,*], Roger Pique-Regi[1,3,*],

[1]Center for Molecular Medicine and Genetics, Wayne State University
[2]Department of Biostatistics, University of Michigan
[3]Department of Obstetrics and Gynecology, Wayne State University

[*]To whom correspondence should be addressed: rpique@wayne.edu,
fluca@wayne.edu.

Contents

1 Data sources **3**

2 Data Preprocessing **3**
2.1 Preprocessing for CENTIPEDE analysis 3
2.2 Preprocessing for allele-specific analysis 4
2.3 Mappability filtering 4
2.4 Selection of genetic variants for ASH analysis 5

3 Identification and Mapping of Active Transcription Factors **5**
3.1 Recalibration of position weight matrices 5
3.2 Generation of CENTIPEDE binding predictions 7
3.3 Comparison of binding predictions to binding QTLs 8

4 Analysis of Allele-Specific Hypersensitivity **8**
4.1 Validation of genotype predictions 8
4.2 Postprocessing of allele-specific data 8

5 Annotation of ASH with binding predictions **9**
5.1 Combining predictions and ASH data 9
5.2 Comparison across different thresholds and using PWM score alone 9
5.3 Comparison to existing functional annotations 10
5.4 Individual motif analysis of binding predictions 10

6 Evaluation of recalibrated sequence models **11**
6.1 Precision versus recall analysis with ChIP-seq 11
6.2 Predicted binding strength correlation analysis with ChIP-seq 11
6.3 PWM recalibration step impact on ASH 11

7 Precision versus recall analysis using DNase-seq and CTCF QTLs **12**

8 Genomic Annotation and Selection Signals **13**
8.1 Allele frequency 13
8.2 Distance to transcription start sites 13
8.3 Identification of TF binding sites enriched for ASH 13
8.4 Selection on transcription factor binding sites 13
8.5 Derived allele frequency of footprint SNPs 14

9 Overlap with Genome-Wide Association Studies **14**
9.1 Analysis of SNPs in the GWAS catalog 14
9.2 Adding annotations to SNPs associated with complex traits 15
9.3 Validating putative causal SNPs by reporter gene assays 15
9.4 Overlap with previous reporter gene assays 15

1 Data sources

A summary of the data used in this paper can be found in Tables S1 and S2. Chromatin accessibility data used for the analysis presented in this study was obtained from the ENCODE Project and the Roadmap Epigenomics Project. The ENCODE Project data was downloaded from the main ENCODE data distribution center (EncodeDCC) at the University of California Santa Cruz (UCSC), publicly available at `ftp://hgdownload.cse.ucsc.edu/goldenPath/hg19/encodeDCC/` (downloaded 07/2013). The Roadmap Epigenomics Project data was downloaded in the form of sequence read archives (SRAs) from the NCBI GEO repository, `http://www.ncbi.nlm.nih.gov/geo/roadmap/epigenomics/` (downloaded 07/2013).

Positional Weight Matrices (PWMs) for 1,949 transcription factors were obtained from the online databases TRANSFAC (Matys et al. 2006) (`http://www.gene-regulation.com/pub/databases.html`, downloaded11/01/11) and JASPAR (Sandelin et al. 2004) (`http://jaspar.genereg.net/`, downloaded 09/23/11).

Known genetic variants from the 1000 Genomes (1KG) Project Phase 1 data were downloaded from `ftp://ftp.1000genomes.ebi.ac.uk/vol1/ftp/phase1/analysis_results/`. Note that except for ASH analysis (Section 2.4), we did not restrict variants used for the analysis on any criteria, including allele frequency. Linkage disequilibrium (LD) data between variants was also obtained from the 1KG Project. The LD data used in this analysis comes from European individuals.

Ensembl transcript positions used to annotate transcription start sites were obtained via the UCSC table browser (`http://genome.ucsc.edu/cgi-bin/hgTables`, downloaded 10/21/12).

Conservation (46-way primate phyloP) data was obtained via the UCSC table browser (`http://genome.ucsc.edu/cgi-bin/hgTables`, downloaded 12/17/13).

Genetic variants identified via genome-wide association studies (GWAS) were extracted from the GWAS catalog (`https://www.genome.gov/26525384`, downloaded 7/16/13).

GWAS meta-analysis data and imputed statistics used to run fgwas via Pickrell (2014) were obtained through personal communication. Annotations used in the model were downloaded from `https://github.com/joepickrell/1000-genomes` (downloaded 03/2014).

2 Data Preprocessing

2.1 Preprocessing for CENTIPEDE analysis

Pre-aligned DNase-seq reads from the Roadmap Epigenomics Project were not directly available, so raw reads were obtained in the form of Sequence Read Archives (SRA) files. We then converted the SRA files to FastQ format using the fastq-dump program from the NCBI SRA toolkit. Reads were then aligned using a custom mapper previously described in (Degner et al. 2012). To identify technical replicates, we extracted sample annotations from the SRA metadata database (downloaded 6/20/13) using the SRAdb R package from Bioconductor (Zhu et al. 2013). Aligned reads from samples identified as technical replicates were then merged using samtools (Li et al. 2009).

Aligned DNase-seq data for ENCODE samples was obtained directly from the EncodeDCC as described in Section 1. As the choice of aligner should have little impact on the ability to run the CENTIPEDE algorithm, we did not remap the reads as for the Roadmap Epigenomics samples.

2.2 Preprocessing for allele-specific analysis

Reads for allele specific analysis have to be carefully processed, as allele specific analysis is especially sensitive to biases in read data. To account for this, we aligned DNase-seq read data using a custom mapper with mappability filters (see Section 2.3). The Roadmap Epigenomics samples were previously aligned using our mapper (Section 2.1). To process the ENCODE samples in the same manner, we obtained raw sequence reads (FastQ format) directly from the EncodeDCC and reads were realigned with our custom mapper. Reads sequenced on old Solexa machines were removed, as these reads were often of lower quality and more prone to base calling errors. Samples with fewer than 25 million reads were removed from analysis, as these typically displayed too low a coverage to be informative. We want to note that the same quality filter thresholds are applied for both footprinting and allele-specific mapping. However, to further minimize mapping errors and reference biases, we also had to apply additional mappability filters (see Section 2.3) for allele-specific analysis.

2.3 Mappability filtering

We created an array of hash tables containing all possible 20-mer reads, where a 4-mer prefix indexes an array of 256 hash tables and the 16-mer suffix is used as hash key. The values of the hash tables record the locations a read can align to (up to a maximum of 128 locations). These arrays are then used for aligning the reads in our custom mapper (Degner et al. 2012). We can also use the hash tables to identify which locations of the genome can generate reads that can align to multiple locations. Two of these arrays have been created and are denoted as M0 and M1:

* M0 Hash Table - The 20-mers starting at each bp position of the genome are added into this table, as well as all 20-mers with alternate alleles (i.e., overlapping SNPs and InDels).

* M1 Hash Table - The same 20-mers as in M0 are generated, but for each genomic 20-mer (reference or variant) we consider all the possible single base pair errors that could have occurred. Each 20-mer has 3x20=60 other 20-mers at Hamming distance of 1.

The M0 hash table is used to align reads with our mapper; which means that only reads that map without mismatches are used. Both hash tables are used to create the two following mappability tracks to assess alignment quality:

* M0 mappability track - For each base of the genome we record the number of locations that match the same exact 20-mer (considering all 1KG genetic variants). When aligning for allele specific analyses, we only consider reads that originate at locations with mappability M0 value exactly equal to one (i.e., reads with unique mappability when there are no base-calling errors).

* M1 mappability track - For each base of the genome we record the number of locations that match the same exact 20-mer (considering all 1KG genetic variants) or any one base pair mismatch. For allele specific analyses we only consider reads that originate at locations with mappability M1 value ≤ 70. Using this value, a location can have up to 69 other loci with only one nucleotide different, reads from which could potentially map to the location of interest if a sequencing error occurs. Using this threshold and considering a base-calling error rate of 0.01 and an average background coverage of $\sim 1X$, we expect $<< 0.5$ reads from other loci to incorrectly map at locations of interest. This is an upper bound calculated from what would be expected when there is at most 70 locations that are one base-pair different when compared to the query sequence. We further considered that the errors that change a 20-mer so it aligns to the other location occur randomly with a probability at most 0.01. It naturally follows that

the number of errors follow a binomial distribution with parameter $N = 70$, and $p = 0.01/20$, and thus has an expected value $E = 0.01/20 * 70 = 0.035 << 0.5$. This upper bound will also hold until the base-calling probability is as high as 5%.

The motivation behind the M1 mappability filter is that very repetitive regions of the genome can generate reads that are very similar to other regions. Even when the base calling error is small, 20-mers with high similarity may generate reference calling biases when doing allele specific analyses. We do not consider a more complex filter as the probability of a read with two base-calling errors at 20bp read-length is very small.

2.4 Selection of genetic variants for ASH analysis

To create a core set of SNPs for ASH analysis, we started with bi-allelic 1KG SNPs (38,248,779) and first removed rare (MAF $< 5\%$) SNPs. To avoid the possibility of multiple SNPs in the same motif, we next removed SNPs within 25 bases up- or downstream of another SNP. Next we removed SNPs in regions prone to mapping biases, masking approximately 1% of the genome (Degner et al. 2012). Ultimately, these filters left (4,828,763) SNPs for analysis.

Aligned reads were then piled up on this set of 1KG SNPs using samtools mpileup and the hg19 reference genome. Reads were discarded if the SNP was either at the first or last base of the read to avoid the possibility of an experimental bias at these positions caused by the DNaseI cleavage preference (Degner et al. 2012). Finally, the following filters were applied to SNPs:

1. The SNP must be covered by $\geq$10 reads
2. 50% or more of the reads covering the SNP cannot start at the same position (i.e., PCR duplicates)
3. The coverage on the SNP cannot be in the top 0.01% sample-wise, as such exaggerated coverage usually indicates an unannotated copy number.

3 Identification and Mapping of Active Transcription Factors

3.1 Recalibration of position weight matrices

To recalibrate the PWMs in our two step approach we first created a reduced subset of motif matches. We obtained 1,949 seed PWMs from online databases as described in Section 1. Using these, we scanned the genome for candidate motifs and calculated the PWM score according to the following formula (Stormo 2000):

$$\begin{aligned} \text{PWM score } (S_l) &= \sum_{w=1}^{W} \log_2 \left(\frac{\Pr(\text{seeing } S_{l+w} \text{on position } w|\text{PWM})}{\Pr(\text{seeing } S_{l+w}|\text{ background})} \right) = \\ &= \sum_{w=1}^{W} \log_2 (p\,[S_{l+w}, w]) - \sum_{w=1}^{W} \log_2 (0.25) = \\ &= \sum_{w=1}^{W} \log_2 (p\,[S_{l+w}, w]) - \log_2 (0.25)\, W \end{aligned} \tag{1}$$

where S_l indicates the observed nucleotide at position l of sequence S, the PWM model is given by the probability $p[S_{l+w}, w]$ of observing the nucleotide S_{l+w}(A,C,G,T) at position w, and W is the motif

length. Here we use a background that assigns the same probability for each base. While this assumption is not true for the entire genome and may also depend on the sequence content of the open chromatin and binding site neighboring regions, when we were developing this study (results not shown) changing this background did not seem to have any major impact on the results (i.e., the average ASH validation rates essentially remained the same). Alternative approaches to PWMs, like using support vector machines (SVM)(Fletez-Brant et al. 2013), may provide a better way to incorporate the impact of the background sequence and more flexibility that the PWM model but were not considered in this study.

Using these PWM derived scores, we created for each motif a reduced set of locations (containing between 5,000 and 15,000 sites) that include both high and low scoring sequence matches to the original seed PWM. Note that we use this reduced set of motif instances to discover which TF are active for each tissue and for recalibrating the PWM model which will then be used to scan the full genome (Section 3.2). In order to construct this reduced sets, we first selected the top 5,000 best scoring sites in the human genome. Then, to expand the sequences included for a motif, we considered using two different strategies. The first strategy was to randomly select additional sequences in which one randomly chosen base w of the PWM was not considered in the PWM score calculation. For the second strategy, which is the one we used for this paper, the additional sequences are selected using a heuristic that relies on sequence conservation due to evolutionary constraints across closely related species. In short, we conducted the following steps:

1. Scan the top 5,000 motif sequence matches in the chimp and macaque genomes
2. Lift over the coordinates to the human genome using the UCSC liftOver tool (excluding chains that are $<$10,000 bases or on very repetitive regions)
3. Calculate the PWM score again using the human sequence

Using this approach we add up to 10,000 new sites from the human genome that have lower PWM scores but are more likely to retain relevant sequence information as they were highly scoring in two of the other primate genome orthologous sequences. Compared to the sequences obtained using the first approach, these sequences are more likely to maintain the TF identity of the original PWM and to harbor true binding sites that will show a footprint. This implicitly assumes that a fraction of these new instances would be functionally conserved (a footprint is observed in humans) and hence the sequence change in the human sequence is more likely to be in the set of sequences that are bound by the TF. This strategy is not expected to perform worse than random nucleotide changes (i.e., first strategy), even in the case that TF binding domain or binding sites are not conserved across the species.

Using these locations and the DNase-seq data listed in Table S1, we applied the CENTIPEDE model for each sample/motif combination. Then, we estimated the overall activity of each factor/sample combination by calculating a Z-score for the following logistic model that is used to calculate the prior probability of binding in CENTIPEDE:

$$\log\left(\frac{\pi_l}{(1-\pi_l)}\right) = \beta_0 + \beta_1 \times \text{PWM Score}_l \qquad (2)$$

where l represents each of the positions in our set of candidate sites, and π_l represents the probability of that position having a footprint. For most factors and experimental samples, a Z-score of at least 5 was

the minimum for which a modest footprint was clearly evident (Figure S4). Using this Z-score value as a threshold, we detect 1,891 factors active (Z-score > 5) in at least one cell-type/tissue.

Finally, we used the CENTIPEDE binding predictions of this initial set to generate recalibrated sequence models. For each factor, we selected the best representative tissue (i.e., the one with highest Z-score) and extracted the sequences predicted to have a factor bound (posterior probability > 0.99). The best representative tissue usually includes more bound regions, perhaps due to a higher transcription concentration factor in the nucleus. In essence, the other tissues only represent a subset of the regions of the so-called best representative tissue. Using these sequences, we calculated a new PWM from the base frequencies of each position in the motif including 20 additional nucleotides on each side to allow for a longer revised motif. We kept this extra bases if the information content at those position for the new motif were high enough, $IC_w = 2 - \sum_b p[b,w] \times log_2(p[b,w]) > 0.25$bits. In general none of these additional bases seem to be very informative, and those with higher IC were already part of the core motif.

In our experience, the recalibrated PWMs are almost always essentially identical independent of the tissue of choice. This is probably because our approach is not capable of completely changing the PWM, a side-by-side comparison for several recalibrated PWMs to the original PWM can be seen in Figure S6. It may be interesting in future research to use or calibrate multiple sequence models that can distinguish different sequence context across tissues for each TF or build models that capable of accommodating multiple modes of binding across tissues or within the same tissue (e.g., considering different cis-regulatory module contexts beyond the scope of this work). Even with this simplifications that only consider one mode of binding (i.e., one PWM model) per TF, an evaluation with ChIP-seq data and ASH in Section 6.3 shows an improvement in the recalibrated model over the old one.

3.2 Generation of CENTIPEDE binding predictions

Using the recalibrated PWM sequence models (see Section 3.1), we scanned the reference genome to identify all motif matches genome-wide. As a threshold on the scan, we calculated an automatic threshold score separately for each sequence model designed to retain all sequences with at least a 10% prior probability of binding as in Equation 2. Scanning was done in two stages. First, we identified every match above the threshold using eq. (1) as before. Next, we scanned the genome, this time only considering motifs that overlapped 1KG variants. For each of these matches we calculated two PWM scores, one for each allele.

To generate the binding predictions, we first trained the CENTIPEDE model on motif locations that do not overlap a SNP for each sample/motif pair using the updated sequence models. As a check for how well calibrated the sequence models are for the data, we examined the correlation between the PWM scores and the CENTIPEDE log ratio. Using a Spearman correlation test and a nominal threshold of $p < 10^{-7}$, we discarded 519 sequence models, leaving us with data for 1,372 sequence models. At the end of this process (Fig. S1) we generated an annotation of footprints with a CENTIPEDE posterior probability > 0.99 divided in two major sets depending on whether they overlap with 1KG sequence variants. Genetic variants in footprints (footprint-SNPs), are further classified based on CENTIPEDE's prior probability of binding (eq. (2)) for each allele into "effect-SNPs" and "switch-SNPs". Effect-SNPs are footprint-SNPs predicted to alter the prior odds of binding $\geq$20-fold based on the logistic sequence model hyperprior in the CENTIPEDE model, while switch-SNPs are effect-SNPs where the allele changes the direction on the prior odds (i.e., one allele has CENTIPEDE prior probability of being

bound > 0.5 and the other one < 0.5). We find that our results are robust to the values chosen for these thresholds (Figure S14). For many TFs, we start from different seed motifs, and the thresholds are slightly different resulting in small differences in numbers of motif instances, but overall the downstream analysis of different motif models that correspond to the same TF are highly consistent.

3.3 Comparison of binding predictions to binding QTLs

To check the accuracy of the predictions, we compared them to a recent binding QTL analysis for CTCF ChIP-seq (Ding et al. 2014). Of the 23,028 unique SNPs that are significant QTLs (1% FDR) in the study, 1,209 of them were contained within the measured CTCF binding regions (i.e., ChIP-seq peak) as defined by (Ding et al. 2014). For each of these QTLs within binding regions, we selected those within CENTIPEDE-predicted CTCF footprints, leaving 151 SNPs shared between the two analyses. For these shared SNPs, we compared the change in probability of binding (alternate prior log ratio - reference prior log ratio) to the binding QTL effect size (β-value, reference allele vs alternate allele).

The two values are well correlated (Spearman ρ=0.82, $p < 2^{-16}$), and only 16 SNPs are predicted to have the opposite effect as the QTL, none of those are predicted to have a functional effect by our model (i.e., are not effect-SNPs). These may be cases where the QTL signal is modulated by a SNP affecting another factor nearby and not the CTCF binding site being interrogated directly. All CTCF QTLs considered here that are also effect-SNPs (46) and switch-SNPs (39), are predicted to affect binding in the same allelic direction as the QTL effect.

4 Analysis of Allele-Specific Hypersensitivity

4.1 Validation of genotype predictions

To verify the genotyping accuracy, we compared the genotype calls from QuASAR for an individual fully resequenced by the 1KG Project (1KG individual NA12878). Of the 1,400 QuASAR-predicted heterozygous loci, all of them were confirmed to be true heterozygotes. Of the 11,278 QuASAR-predicted homozygous loci, only seven of them were actually heterozygotes. Additionally, all of the true homozygous calls were correct for the predicted allele. The seven miscalled heterozygotes are likely cases of extreme allelic imbalance, as QuASAR was designed to be conservative to avoid miscalling homozygous genotypes as heterozygotes with extreme allelic imbalance. The results of our comparisons are summarized in Table S5.

4.2 Postprocessing of allele-specific data

To further filter out samples not well-suited for allele specific analysis, including cancer tissues and samples from pooled individuals (e.g., Figure S9), we examined two parameters estimated by QuASAR, ρ and M, as well as the non-reference allele frequency ϕ obtained from 1KG. The ρ parameter represents the proportion of reads overlapping a SNP that match the reference allele. For heterozygous SNPs under the null model (no allelic imbalance) we would expect that the average $\hat{\rho}$ should be centered at or near 0.5. Deviation from 0.5 in a sample can be an indication of genetic aberrations, such as in a cancer sample, where copy number variation can be extensive, or very high base-calling error rates. We also examined the correlation (Pearson correlation coefficient) between the ρ estimates and ϕ for each heterozygous

locus, as the two should be independent of each other. Otherwise, this is a strong indication of sample mix-up or cross-sample contamination as new modes appear at $\rho = 0.25, 0.75$ or other intermediate frequencies with probabilities that are correlated with the reference allele frequency ϕ. The M parameter in QuASAR controls the degree of overdispersion of the beta-binomial distribution in the QuASAR model. A very high value of M indicates that the beta-binomial is almost a binomial distribution. On the other hand, a low value of M indicates more and more dispersion and a very high uncertainty in the underlying ρ being centered around 0.5, as is the case of samples with chromosomal aberrations and copy number alterations, for example in cancer cell lines (see Figure S9B). After applying all filters, 316 samples remained for ASH analysis. A summary of the post-processing results can be found in Table S6 and Figure S10.

5 Annotation of ASH with binding predictions

5.1 Combining predictions and ASH data

To determine which positions displaying ASH fall within a predicted footprint, we overlapped the allele ratios for heterozygous SNPs (hSNPs) in DHS sites (DHS-hSNPs) with CENTIPEDE footprint predictions in each sample. We then created a final set of annotated ASH-hSNPs by aggregating the data across each sample and factor. For cases where an hSNP is within multiple predicted binding sites, we selected the factor whose CENTIPEDE footprint model has the greatest log-likelihood ratio. This generated a set of 204,757 hSNPs across all samples. As the same hSNP could affect multiple cell-types, this set of 204,757 hSNPs reflects 961,297 observations of ASH. For hSNPs predicted to have an effect on binding (effect-hSNPs), we determined which ones were predicted to have an effect in the same direction as the observed allele ratio (e.g., the allele with a higher PWM score is observed more often in the DNase data). We then partitioned the data into three non-overlapping categories: 1) hSNPs in predicted footprints whose binding effect is in the direction predicted, 2) all other hSNPs in footprints, 3) all other DHS-hSNPs. Because each annotation has a different prior expectation of being functional (Figure 2A), we readjusted for multiple testing within each annotation separately by applying the Storey q-value method on the p-values obtained from the QuASAR test to estimate the false discovery rate (FDR), following the strategy of Benjamini and Bogomolov (2014). The results of this analysis are summarized in the main text, Table 1.

Additionally, we examined the observed allele ratios ($\hat{\rho}_{obs}$) across different CENTIPEDE annotations. Fig. S12 shows that effect-hSNPs and switch-hSNPs tend to have a higher magnitude of allelic imbalance. If we consider SNPs that are in the tail of the distribution $|0.5 - \hat{\rho}_{obs}| > 0.15$, effect-hSNPs show a moderate but significant enrichment (1.29-fold, Fisher p = 1.1×10^{-229}) compared to DHS-hSNPs.

5.2 Comparison across different thresholds and using PWM score alone

In order to study the impact of the threshold in defining the effect-SNP category (Section 3.2) we experimented with different settings. The results (Figure S14) show that the density of low p-values compared to the uniform distribution increases with a higher threshold on the minimum change in the prior probability of binding and vice versa. Here we opted to choose a hard-threshold but it may be possible to

develop downstream analytical methods that could take as input the CENTIPEDE calculated probabilities directly.

It is also interesting to see what is the added benefit of only considering CENTIPEDE footprint models compared to using the PWM score alone. To see how well the PWM score alone predicts ASH, we compared our ASH results for DHS-hSNPs to the change in PWM score at those SNPs. Here, we use the PWM score from the original seed motifs and we examined the degree of ASH for SNPs at various PWM score differences between the two alleles. As expected, we find that the proportion of ASH increases among SNPs with a higher PWM score difference S15. For a very large PWM score difference threshold we start to approach an ASH enrichment similar to that for effect-SNPs, but very few SNPs pass this threshold. These additional ASH enrichment results further demonstrate that the best strategy is to integrate the sequence and footprint information in defining which SNPs are more likely to be functional.

5.3 Comparison to existing functional annotations

To compare our functional predictions to existing annotations, we downloaded data from two studies seeking to annotate functional non-coding variation, (Farh et al. 2015) (7,747 SNPs), (Maurano et al. 2012) (5,654 SNPs). These lists are not the full sets of non-coding variation from each study, but those that overlap GWAS hits. For each set of SNPs, we overlapped our ASH data to determine the proportion of SNPs showing allelic imbalance, leaving 3,838 and 2,187 SNPs, respectively. For both datasets, we find that while there is an enrichment for ASH (Figure S13), it is similar to the modest enrichment seen for DHS- and footprint-SNPs, suggesting that a positional overlap with a DHS region or binding site is not enough to support a claim of functional impact on binding.

We also examined ASH at variants with a large CADD score (23,027 SNPs with CADD score $\geq$20, (Kircher et al. 2014)). Again we find only a modest enrichment for ASH within this set of SNPs (Figure S13). This is perhaps due to CADD being trained on very deleterious variants which may rely more heavily on sequence conservation and other features compared to an explicit model that only integrates sequence and functional information that is relevant for TF binding. The additional information contained in the CADD score could provide important clues on the genetic variant being functional at the organismal level, but it does not directly provide a mechanism of action or a TF specific functional score for the genetic variant impact on disrupting binding.

Looking directly at conservation scores, we examined the primate phyloP scores of ASH-hSNPs. SNPs with extreme conservation scores ($|\text{phyloP}| > 5$) do show an enrichment for ASH (Figure S16, however few SNPs have such extreme conservation scores.

5.4 Individual motif analysis of binding predictions

In order to evaluate the extent to which the newly defined sequence models accurately predict ASH, we compared CENTIPEDE predictions and ASH analysis for each motif individually. We examined motifs containing at least 10 heterozygous SNPs in footprints for which we can estimate the ASH allelic ratio $\hat{\rho}$. $\hat{\rho}$ is calculated from the sequencing data as,

$$\hat{\rho} = \frac{\text{\# reads w/ reference allele}}{\text{\# reads w/ reference or alternate allele}} \tag{3}$$

For each motif, we compared the CENTIPEDE predictions to the ASH data by calculating the Spearman's correlation between the observed allelic imbalance (proportion of reference reads) and the difference in binding predictions (ΔPr(binding)). Additionally, we fit a logistic model,

$$\text{logit}(\hat{\rho}) \sim \beta_0 + \beta_1 * \Delta\text{logit}(p) \quad (4)$$

where $\Delta\text{logit}(p)$ is the change in log prior odds predicted by the sequence model in CENTIPEDE. We fit the model on SNPs displaying some allelic imbalance (nominal ASH p-value < 0.1) to focus on how well our predictions accurately capture allelic imbalance (note that this threshold is not used for the Spearman correlation analysis where all instances are used). Figure S11 shows the correlation between our prediction and the observed ASH for the most predictive motif, belonging to the factor AP-1. Table S7 shows the correlation results for each motif.

6 Evaluation of recalibrated sequence models

6.1 Precision versus recall analysis with ChIP-seq

To compare the new sequence models to the originals, we first performed precision recall operating characteristic (P-ROC) curve analysis using PWM scores of motif matches and ENCODE ChIP-seq peaks from GM12878 samples. We annotated a list of all binding sites identified as a PWM match or as having a ChIP-peak, using the PWM score as the predictions and the presence or absence of a ChIP-seq signal as the labels. For sites with a ChIP-seq signal but no PWM match above the scan threshold a default PWM score of 0 was used (i.e., all ChIP-seq peaks are included in the analysis). For each selected factor, we compared the precision-recall curve using the original PWM models and the updated PWM models (Figure S7). The curves show that in general, for a given precision (precision = 1 - FDR, false discovery rate), the updated sequence models have higher recall (sensitivity) than the original PWM in detecting ChIP-seq peaks.

6.2 Predicted binding strength correlation analysis with ChIP-seq

We also examined the correlation between the PWM scores (for the original seed and the recalibrated motif models) and the number of ChIP-seq reads. For each PWM we identified all matching sites genome-wide and extracted the ChIP-seq read coverage. Compared to the seed PWMs, we find that the revised PWMs are better correlated with the ChIP-seq data. Data for the individual comparisons can be seen in Figure S8. The new recalibrated models, seem to better capture the relationship between the prior probability of binding derived from the PWM score and TF occupancy as measured by ChIP-seq reads. This indicates that we are also capturing a wide range of binding events including weak binding sites.

6.3 PWM recalibration step impact on ASH

We also wanted to examined whether the recalibration process preferentially selected sites with the strongest binding, and therefore most affected by variation. If so, this would potentially bias our downstream ASH analysis, as we partition the SNPs based on their predicted impact on binding (Section 5.1). To see if this was the case, we compared ASH results within footprints predicted by the two sets of sequence models. Using the original seed PWMs, we ran CENTIPEDE as in Section 3.2.

For each set of sequence models, we compared the proportion of SNPs within footprints predicted to have an effect. We find that the recalibrated sequence models discover more variation within the footprints overall. However, the proportion of SNPs with low p-values ($p < 0.05$) in footprints predicted to affect binding versus those that do not, remains extremely similar between the old models (OR 2.14, Fisher $p = 1.6 \times 10^{-289}$) and the new models (OR 2.08, Fisher $p = 1.5 \times 10^{-263}$). Table S4 shows the values used for this comparison. The new recalibrated models do not necessarily provide a much better discrimination on which genetic variants are functional, but they seem to yield a higher number of regulatory sequences and genetic variants.

7 Precision versus recall analysis using DNase-seq and CTCF QTLs

To facilitate the comparison with other methods we focused our analysis on the same DNase-seq sensitivity QTL (dsQTL) signals as in Lee et al. (2015). We downloaded the results from their Supplementary Table 1 to reproduce their Figure 2e. Using the same definition for what is the known underlying truth, we resampled the data so the genetic variants that are not dsQTLs have the average genomic background probability to be in a footprint. We then intersected the predictions from the SVM approach with our effect-SNP annotation for LCLs using different cut-off thresholds on the change of the prior log odds. The same procedure was then repeated for CATO scores Maurano et al. (2015) in which we intersected their Supplementary data set 3 with Supplementary Table 1 from Lee et al. (2015). Then we proceeded to draw the precision versus recall operating curves (PROC) for each method using R-package ROCR (version 1.0-7). In Figure 2B, we recapitulated the results from the SVM, CADD and GERP methods, and we also show on the same graph the results from CATO and our annotated effect-SNPs.

We would like to note that PROC are very useful for analyzing performance when there is no uncertainty about the underlying truth (e.g. simulated data). However, QTL analysis as many other statistical analyses using limited sample size and data will not give the perfect golden standard for a yes/no answer necessary for the PROC analysis. On its own, QTL analysis may have false negatives due to lack of power (e.g. for low allele frequency variants), or false positives due to linkage disequilibrium to the true causal SNP. This could lead to discrepancies between different PROC analyses, and for this reason we have not used our ASH results to do a PROC analysis, instead we opted for checking the distribution of p-values in which we can better manage uncertainty. In any case, with this caveat in mind to interpret the results from dsQTLs we demonstrate that our annotation is highly accurate.

In addition to predict disruption of binding, CATO and our annotation also predicts which TF motif is the most likely to be affected. In order to assess how good is this prediction compared to a non-specific sequence model we used a similar approach. Using the same type of PROC analysis, we intersected the predictions for dsQTLs described before to CTCF QTLs identified by Ding et al. (2014). We focused on CTCF QTLs that are also dsQTLs, because this facilitates the comparison with the results reported by Lee et al. Here true positives are defined as those dsQTLs that are also CTCF QTLs, and the true negatives are both dsQTLs and non-dsQTLs for which we have predictions from all methods but are not identified as CTCF QTLs. Again, we drew the precision versus recall operating curves (PROC) for each method using R-package ROCR (Figure 2C). This result demonstrates that our annotation has better performance in predicting the identity of the TF at least for CTCF. This is presumably a consequence of the footprint information integrated in our model which helps in improving TF specificity.

8 Genomic Annotation and Selection Signals

8.1 Allele frequency

Allele frequency for each 1KG SNP was obtained as described in Section 1. For each SNP, we calculated the minor allele frequency by taking the absolute value of the difference between the alleles frequencies and 0.5. Coding SNP annotations were also obtained from 1KG Project (phase 1 release). We examined bi-allelic coding SNPs categorized as either ’synonymous’ or ’nonsynonymous’.

8.2 Distance to transcription start sites

For a given locus, distance to the nearest TSS was calculated as absolute value distances to the nearest annotated TSS. Using Ensembl gene annotations (see Section 1), we determined the distance for each SNP in our set. For motif-wide analysis, we determined the median distance to the nearest TSS across all binding sites genome-wide.

8.3 Identification of TF binding sites enriched for ASH

To identify which TF binding sites are enriched or depleted for ASH-hSNPs, we calculated, for each factor, the proportion of binding sites containing ASH-hSNPs to all binding sites containing a heterozygous SNP (ASH enrichment ratio). As the proportions can be skewed at lower total numbers, we included only factors with at least 100 heterozygous SNPs across all binding sites genome-wide. For the 368 factors that met this criteria, we estimated the enrichment or depletion of ASH by calculating the fold-change between the ASH enrichment ratio and the average ASH enrichment ratio across all binding sites (with $>$100 hSNPs), using a binomial test to assess significant difference between the ratios. Factors whose binding sites are enriched or depleted for ASH-hSNPs (at a nominal p-value cutoff of $p < 0.01$) are displayed in Table S9.

For this analysis we used ASH-hSNPs detected with an FDR=20%. Results are similar for other FDR cut offs if we increase this threshold, but we cannot lower much the FDR threshold to get enough SNPs for the downstream analyses.In general we observed that effect sizes increase with lower FDR threshold as we include less false positives, but the confidence intervals get much wider. The effect of using a higher FDR threshold in downstream analyses seems an appropriate tradeoff between a more conservative estimate of the effect size (as those get shrunken to the overall mean) and a more narrow confidence interval better suited to detect the differences across TFs.

8.4 Selection on transcription factor binding sites

The McDonald-Kreitman test compares polymorphism (within species variation) and divergence (between species variation) for non-synonymous and synonymous sites. The null hypothesis being that under neutrality the ratio of non-synonymous to synonymous sites is the same within and between species.To identify transcription factors with binding sites departing from neutrality, here we defined non-synonymous and synonymous regulatory sites based on our effect-SNPs annotation. Using the footprint annotations from CENTIPEDE, we identified all footprints that do not contain known human polymorphisms. We used the UCSC liftOver tool to obtain orthologous regions in the Chimpanzee genome (panTro3 assembly), using a minimum remap threshold of 10%. At these loci in the chimp genome, we

calculated PWM scores as in Section 3.1. Next, using the model obtained from CENTIPEDE on the human sites, we calculated the sequence-based probabilities of binding for the chimpanzee sites. Sites where the prior probability of binding differ from the human sites were classified as "divergent", and were further categorized by the difference in binding affinity: "functional" (analogous to non-synonymous) for those that differ by $\geq$20-fold, and "silent" for those that do not. For the polymorphic sites, we used binding sites with effect-SNPs as "functional", and those with footprint-SNPs that are not effect-SNPs as "silent". For each factor motif, we calculated the number of binding sites across the entire genome belonging to each category to build a contingency table similar to the one built in the McDonald-Kreitman test:

	Divergent	Polymorphic
Functional	D_f	P_f
Silent	D_s	P_s

Finally, we calculated a selection score using the following formula:

$$\text{Selection score} = \frac{D_f/D_s}{P_f/P_s} \tag{5}$$

To test for enrichment, we used a fisher exact test on the contingency table, and used the Benjamini-Hochberg method to adjust for multiple testing. A full list of motif scores and the data used to calculate them can be found in Table S12. Using different thresholds (for example, calling silent SNPs those with prior odds ratios < 10 and functional SNPs those with prior odds ratio > 20) did not have any major impact on the analysis results.

8.5 Derived allele frequency of footprint SNPs

Orthologous alleles in chimpanzee were obtained via the UCSC Table Browser, using the "snp142OrthoPt4Pa2Rm3" table from the hg19 genome assembly. For 1,371 motifs with >0 binding site SNPs with known chimpanzee alleles, the derived allele frequency was calculated using the 1KG global allele frequencies (see Section 1). Derived allele frequency was then categorized into 8 bins: [0, 0.001), [0.001, 0.005), [0.005, 0.01), [0.01, 0.05), [0.05, 0.2), [0.2, 0.5), [0.5, 0.9), and [0.9, 0.95]. Similarly, the selection scores were divided into 8 bins: [-6, -3), [-3, -1), [-1, -0.5), [-0.5, -0.1), [-0.1, 0.1), [0.1, 0.5), [0.5, 1), and [1, 3]. We then calculated the enrichment for each (DAF, Selection) bin pair, defined as the ratio between the observed number of SNPs in that bin and the expected number if each DAF/Selection pair is independent of the rest. A barplot of the enrichment data, including on for singletons and doubletons, can be found in Figure S19.

9 Overlap with Genome-Wide Association Studies

9.1 Analysis of SNPs in the GWAS catalog

We created an expanded GWAS catalog by adding SNPs in linkage disequilibrium (LD) with each GWAS hit, using 1KG LD data for European populations $r^2 > 0.8$. To identify overlap between our annotations and those associated with a GWAS trait, we intersected our results with this expanded catalog, but

counting only one hit per GWAS locus. We used a Fisher exact test to determine if the proportion of effect-SNPs for a given annotation were enriched in the catalog.

9.2 Adding annotations to SNPs associated with complex traits

We integrated our CENTIPEDE footprint annotations into the combined models learned in Pickrell (2014) for GWAS meta-studies corresponding to 18 traits (Table S13), using the fgwas command line program. We assessed enrichment or depletion for footprint annotations using the log_2(enrichment) values, excluding any motifs whose 95% confidence interval (CI) spanned zero. For each TF motif whose binding sites are either significantly enriched or depleted for trait-associated SNPs (Figure S20), we examined the SNPs whose posterior probability of association (PPA) with a trait had been increased by the addition of our annotation. Overall we found 88 unique SNPs whose associations were strengthened by our footprint annotations (Table S15).

9.3 Validating putative causal SNPs by reporter gene assays

From the 88 SNPs identified by the fgwas analysis, we considered GWAS-relevant effect-SNPs located in active footprints in LCLs (the cell line used for transfection) and ranked them on the Spearman correlation coefficient in S7. We initially selected the top 25 SNPs with a positive correlation, but the assays for 4 of them failed for several technical reasons (e.g., cloning step failed). To validate the predicted allelic effects on gene expression for the remaining 21 SNPs, we first constructed inserts containing the reference or alternate allele for each SNP of interest. Each region was amplified from genomic DNA extracted from LCLs (Coriell). Primers were designed using the Infusion Clontech online primer design tool for inserts containing the SNP of interest $\pm$100bp. Primers were ordered from IDT technologies. Inserts were amplified by PCR and pGL4.23 plasmid was linearized (inverse PCR) using Clontech Hifi PCR premix and following the manufacturer's instructions. PCR products and linearized plasmid were resolved on agarose gel, excised and purified using Nucleospin gel extraction and PCR cleanup kit (Clontech). Inserts were cloned into linearized pGL4.23 using the Infusion Cloning HD kit (Clontech). Transformation was done using Stellar Competent cells (Clontech) and DNA was extracted from selected colonies using the PureYield kit (Promega). The allelic status and the absence of artifactual mutations of each clone was validated by Sanger sequencing performed by Genewiz. Transfections were performed into GM18507 using the standard protocol for the Nucleofector electroporation (Lonza). After 10 hours we measured Firefly and Renilla (transfection control) luciferase activity using the Dual-Glo Luciferase Assay Kit (Promega) on the GloMax instrument (Promega). Luciferase activity was measured for up to 20 replicate experiments. We then used a t-test to identify significant differences in the expression of the reporter gene, calculated by the ratio of the firefly to the renilla activity, normalized to the ratio of the activity in the untransfected cells. We contrasted the activity of each construct to the pGL4.23 vector, to assess enhancer/repressor activity of each region. To evaluate allele-specific effects, we contrasted the activity of the reference allele to the alternate allele for each region. These results are summarized in Table S16 and in Figure 7.

9.4 Overlap with previous reporter gene assays

To compare our predictions to existing reporter gene assays results, we investigated the overlap between our footprints and the regions described in Kheradpour et al. (2013). In the regions they tested, we

identified 47 footprints in HepG2 cells and 70 footprints in K562 cells. If the mutated base (or bases, in the case of the scrambled motifs) overlapped the footprint position, we calculated a new probability of binding using the PWM match score and the model learned from the matched tissue, either HepG2 or K562. In cases where multiple footprints overlapped, we selected the footprint model with the greatest log-likelihood ratio (as in Section 5.1). Additionally, we restricted our analysis to only constructs in which the wild type is significantly expressed (mean normalized expression > 3.5), as in Lee et al. (2015). Overall, we made predictions covering 22 construct pairs (8 in HepG2 and 14 in K562), corresponding to 9 scrambled motifs, and 13 single base mutations. Our predictions are well correlated with the reporter gene assay results (Spearman's $\rho = 0.76$, p-value = 4.37×10^{-05}, Figure S22).

References

Benjamini, Y. and Bogomolov, M., 2014. Selective inference on multiple families of hypotheses. *Journal of the Royal Statistical Society. Series B: Statistical Methodology*, **76**:297–318.

Degner, J. F., Pai, A. A., Pique-Regi, R., Veyrieras, J.-B., Gaffney, D. J., Pickrell, J. K., De Leon, S., Michelini, K., Lewellen, N., Crawford, G. E., *et al.*, 2012. DNase I sensitivity QTLs are a major determinant of human expression variation. *Nature*, **482**(7385):390–4.

Ding, Z., Ni, Y., Timmer, S. W., Lee, B.-K., Battenhouse, A., Louzada, S., Yang, F., Dunham, I., Crawford, G. E., Lieb, J. D., *et al.*, 2014. Quantitative genetics of ctcf binding reveal local sequence effects and different modes of x-chromosome association. *PLoS Genet*, **10**(11):e1004798.

Farh, K. K.-H., Marson, A., Zhu, J., Kleinewietfeld, M., Housley, W. J., Beik, S., Shoresh, N., Whitton, H., Ryan, R. J. H., Shishkin, A. A., *et al.*, 2015. Genetic and epigenetic fine mapping of causal autoimmune disease variants. *Nature*, **518**(7539):337–343.

Fletez-Brant, C., Lee, D., McCallion, A. S., and Beer, M. A., 2013. kmer-SVM: a web server for identifying predictive regulatory sequence features in genomic data sets. *Nucleic acids research*, **41**(Web Server issue).

Kheradpour, P., Ernst, J., Melnikov, A., Rogov, P., Wang, L., Zhang, X., Alston, J., Mikkelsen, T. S., and Kellis, M., 2013. Systematic dissection of regulatory motifs in 2000 predicted human enhancers using a massively parallel reporter assay. *Genome research*, **23**(5):800–811.

Kircher, M., Witten, D. M., Jain, P., O'Roak, B. J., Cooper, G. M., and Shendure, J., 2014. A general framework for estimating the relative pathogenicity of human genetic variants. *Nature genetics*, **46**(3):310–5.

Lee, D., Gorkin, D. U., Baker, M., Strober, B. J., Asoni, A. L., McCallion, A. S., and Beer, M. A., 2015. A method to predict the impact of regulatory variants from DNA sequence. *Nat Genet*, **47**(8):955–961.

Li, H., Handsaker, B., Wysoker, A., Fennell, T., Ruan, J., Homer, N., Marth, G., Abecasis, G., and Durbin, R., 2009. The Sequence Alignment/Map format and SAMtools. *Bioinformatics*, **25**:2078–2079.

Matys, V., Kel-Margoulis, O. V., Fricke, E., Liebich, I., Land, S., Barre-Dirrie, A., Reuter, I., Chekmenev, D., Krull, M., Hornischer, K., *et al.*, 2006. TRANSFAC and its module TRANSCompel: transcriptional gene regulation in eukaryotes. *Nucleic acids research*, **34**:D108–D110.

Maurano, M. T., Haugen, E., Sandstrom, R., Vierstra, J., Shafer, A., Kaul, R., and Stamatoyannopoulos, J. A., 2015. Large-scale identification of sequence variants influencing human transcription factor occupancy in vivo. *Nat Genet*, **advance online publication**.

Maurano, M. T., Humbert, R., Rynes, E., Thurman, R. E., Haugen, E., Wang, H., Reynolds, A., Sandstrom, R., Qu, H., Brody, J., *et al.*, 2012. Systematic Localization of Common Disease-Associated Variation in Regulatory DNA. *Science*, **337**(6099):1190–1195.

Pickrell, J. K., 2014. Joint Analysis of Functional Genomic Data and Genome-wide Association Studies of 18 Human Traits. *The American Journal of Human Genetics*, **94**(4):559–573.

Sandelin, A., Alkema, W., Engström, P., Wasserman, W. W., and Lenhard, B., 2004. JASPAR: an open-access database for eukaryotic transcription factor binding profiles. *Nucleic acids research*, **32**:D91–D94.

Stormo, G. D., 2000. DNA binding sites: representation and discovery. *Bioinformatics (Oxford, England)*, **16**(1):16–23.

Zhu, Y., Stephens, R., Meltzer, P., and Davis, S., 2013. SRAdb: query and use public next-generation sequencing data from within R. *BMC Bioinformatics*, **14**:19.
