# Supplementary figures and images for: Which Genetics Variants in DNase-Seq Footprints Are More Likely to Alter Binding? (part 2 of 2)
Source: PLoS Genet. 2016 Feb 22;12(2):e1005875. doi: 10.1371/journal.pgen.1005875 (PMC4764260; doi:10.1371/journal.pgen.1005875)

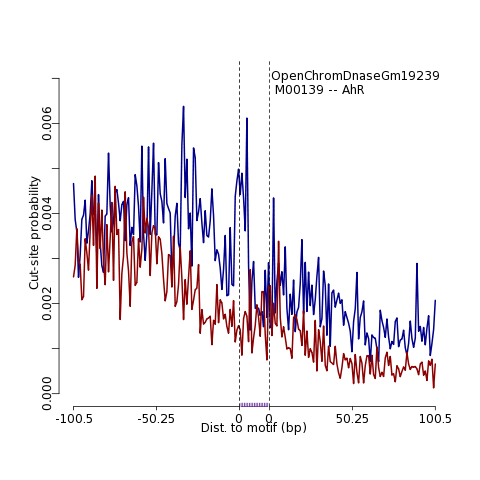

Supplement: S3 File — For each motif, footprint profiles are aggregated across all binding sites in all 653 DNase-seq samples. Color indicates which strand the motif matches, positive (blue) or negative (red). Text in the upper left denotes the tissue with the highest Z-score from the CENTIPEDE mode, the motif ID, and the corresponding transcription factor. (GZ) [file pgen.1005875.s004.tar.gz › recalibratedMotifShape/M00139.lambda.png]

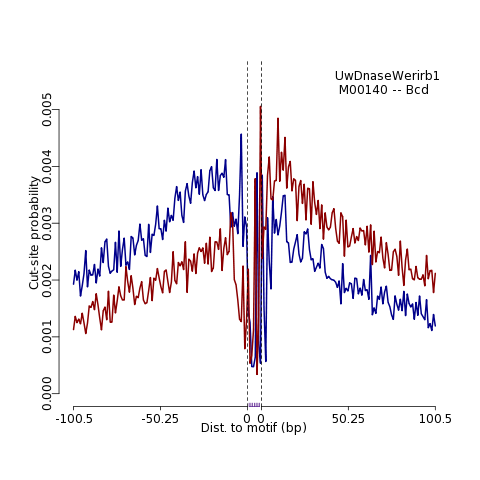

Supplement: S3 File — For each motif, footprint profiles are aggregated across all binding sites in all 653 DNase-seq samples. Color indicates which strand the motif matches, positive (blue) or negative (red). Text in the upper left denotes the tissue with the highest Z-score from the CENTIPEDE mode, the motif ID, and the corresponding transcription factor. (GZ) [file pgen.1005875.s004.tar.gz › recalibratedMotifShape/M00140.lambda.png]

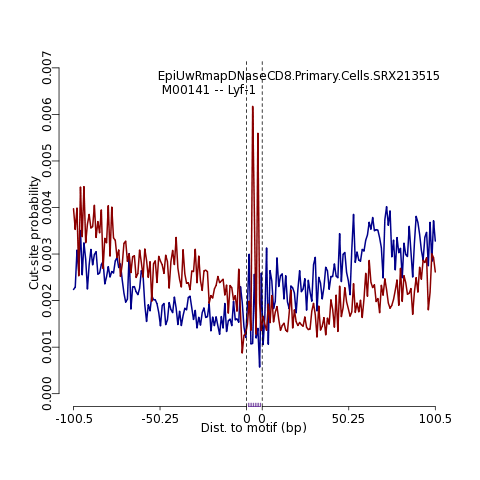

Supplement: S3 File — For each motif, footprint profiles are aggregated across all binding sites in all 653 DNase-seq samples. Color indicates which strand the motif matches, positive (blue) or negative (red). Text in the upper left denotes the tissue with the highest Z-score from the CENTIPEDE mode, the motif ID, and the corresponding transcription factor. (GZ) [file pgen.1005875.s004.tar.gz › recalibratedMotifShape/M00141.lambda.png]

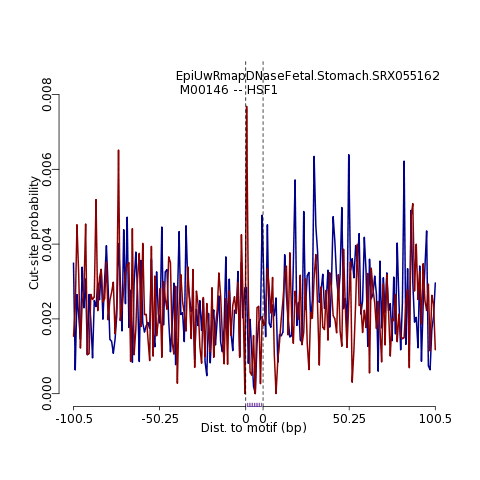

Supplement: S3 File — For each motif, footprint profiles are aggregated across all binding sites in all 653 DNase-seq samples. Color indicates which strand the motif matches, positive (blue) or negative (red). Text in the upper left denotes the tissue with the highest Z-score from the CENTIPEDE mode, the motif ID, and the corresponding transcription factor. (GZ) [file pgen.1005875.s004.tar.gz › recalibratedMotifShape/M00146.lambda.png]

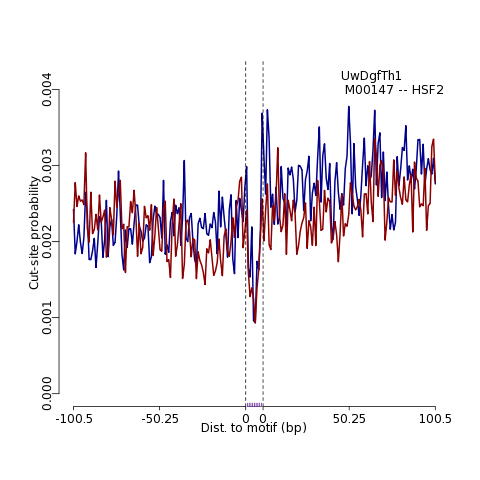

Supplement: S3 File — For each motif, footprint profiles are aggregated across all binding sites in all 653 DNase-seq samples. Color indicates which strand the motif matches, positive (blue) or negative (red). Text in the upper left denotes the tissue with the highest Z-score from the CENTIPEDE mode, the motif ID, and the corresponding transcription factor. (GZ) [file pgen.1005875.s004.tar.gz › recalibratedMotifShape/M00147.lambda.png]

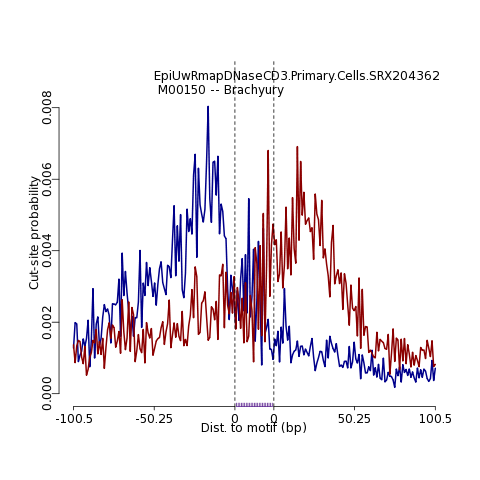

Supplement: S3 File — For each motif, footprint profiles are aggregated across all binding sites in all 653 DNase-seq samples. Color indicates which strand the motif matches, positive (blue) or negative (red). Text in the upper left denotes the tissue with the highest Z-score from the CENTIPEDE mode, the motif ID, and the corresponding transcription factor. (GZ) [file pgen.1005875.s004.tar.gz › recalibratedMotifShape/M00150.lambda.png]

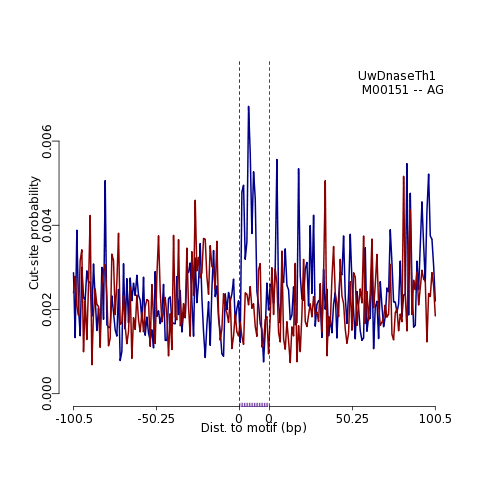

Supplement: S3 File — For each motif, footprint profiles are aggregated across all binding sites in all 653 DNase-seq samples. Color indicates which strand the motif matches, positive (blue) or negative (red). Text in the upper left denotes the tissue with the highest Z-score from the CENTIPEDE mode, the motif ID, and the corresponding transcription factor. (GZ) [file pgen.1005875.s004.tar.gz › recalibratedMotifShape/M00151.lambda.png]

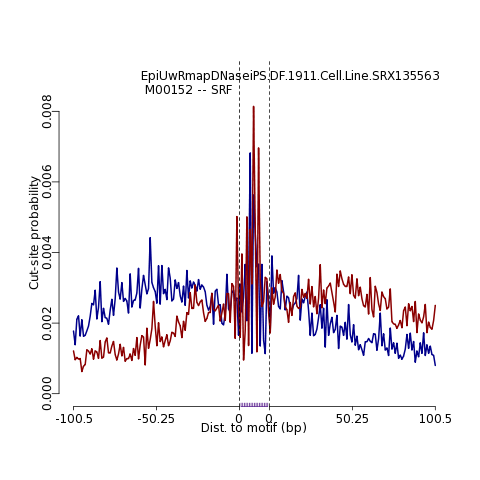

Supplement: S3 File — For each motif, footprint profiles are aggregated across all binding sites in all 653 DNase-seq samples. Color indicates which strand the motif matches, positive (blue) or negative (red). Text in the upper left denotes the tissue with the highest Z-score from the CENTIPEDE mode, the motif ID, and the corresponding transcription factor. (GZ) [file pgen.1005875.s004.tar.gz › recalibratedMotifShape/M00152.lambda.png]

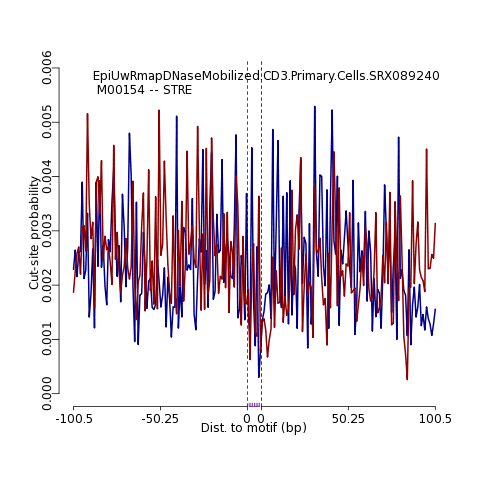

Supplement: S3 File — For each motif, footprint profiles are aggregated across all binding sites in all 653 DNase-seq samples. Color indicates which strand the motif matches, positive (blue) or negative (red). Text in the upper left denotes the tissue with the highest Z-score from the CENTIPEDE mode, the motif ID, and the corresponding transcription factor. (GZ) [file pgen.1005875.s004.tar.gz › recalibratedMotifShape/M00154.lambda.png]

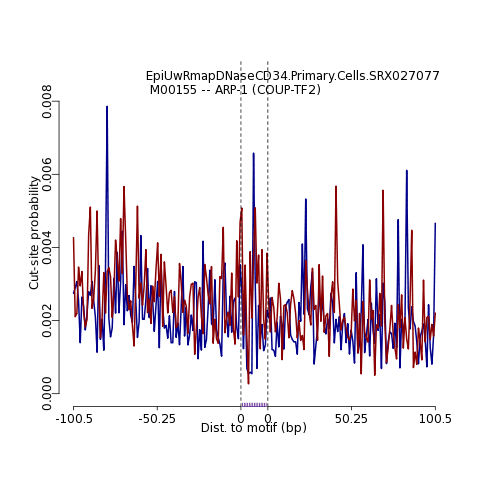

Supplement: S3 File — For each motif, footprint profiles are aggregated across all binding sites in all 653 DNase-seq samples. Color indicates which strand the motif matches, positive (blue) or negative (red). Text in the upper left denotes the tissue with the highest Z-score from the CENTIPEDE mode, the motif ID, and the corresponding transcription factor. (GZ) [file pgen.1005875.s004.tar.gz › recalibratedMotifShape/M00155.lambda.png]

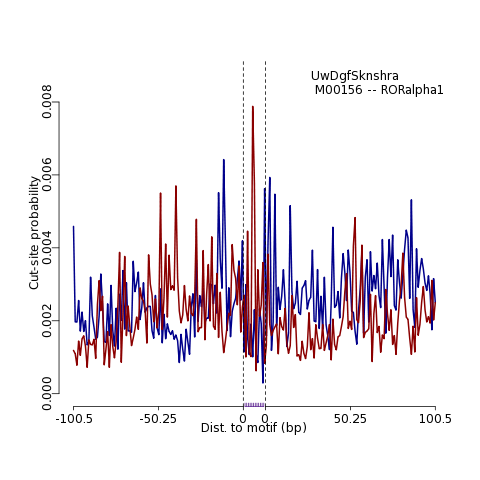

Supplement: S3 File — For each motif, footprint profiles are aggregated across all binding sites in all 653 DNase-seq samples. Color indicates which strand the motif matches, positive (blue) or negative (red). Text in the upper left denotes the tissue with the highest Z-score from the CENTIPEDE mode, the motif ID, and the corresponding transcription factor. (GZ) [file pgen.1005875.s004.tar.gz › recalibratedMotifShape/M00156.lambda.png]

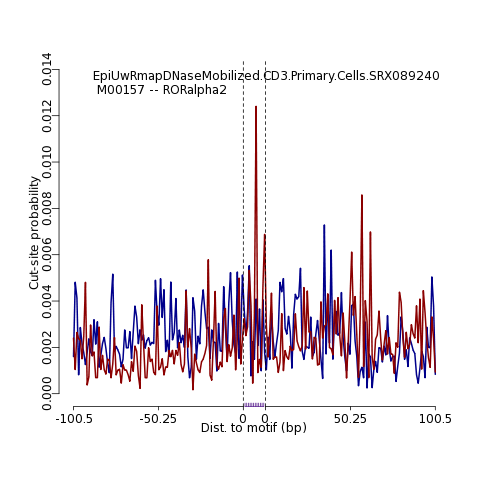

Supplement: S3 File — For each motif, footprint profiles are aggregated across all binding sites in all 653 DNase-seq samples. Color indicates which strand the motif matches, positive (blue) or negative (red). Text in the upper left denotes the tissue with the highest Z-score from the CENTIPEDE mode, the motif ID, and the corresponding transcription factor. (GZ) [file pgen.1005875.s004.tar.gz › recalibratedMotifShape/M00157.lambda.png]

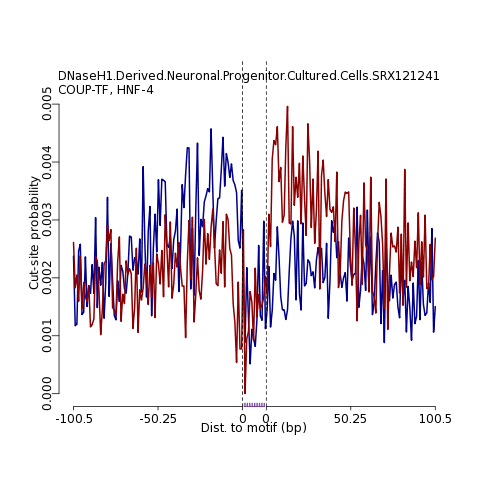

Supplement: S3 File — For each motif, footprint profiles are aggregated across all binding sites in all 653 DNase-seq samples. Color indicates which strand the motif matches, positive (blue) or negative (red). Text in the upper left denotes the tissue with the highest Z-score from the CENTIPEDE mode, the motif ID, and the corresponding transcription factor. (GZ) [file pgen.1005875.s004.tar.gz › recalibratedMotifShape/M00158.lambda.png]

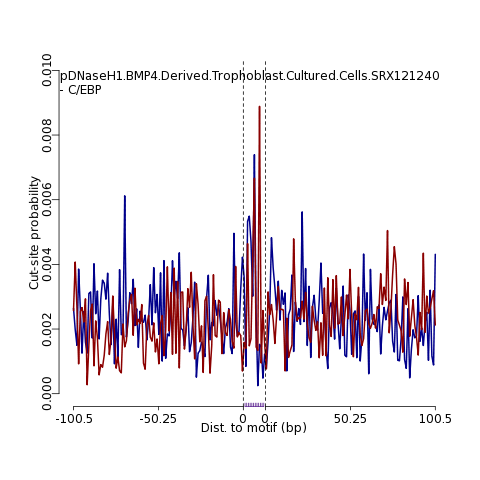

Supplement: S3 File — For each motif, footprint profiles are aggregated across all binding sites in all 653 DNase-seq samples. Color indicates which strand the motif matches, positive (blue) or negative (red). Text in the upper left denotes the tissue with the highest Z-score from the CENTIPEDE mode, the motif ID, and the corresponding transcription factor. (GZ) [file pgen.1005875.s004.tar.gz › recalibratedMotifShape/M00159.lambda.png]

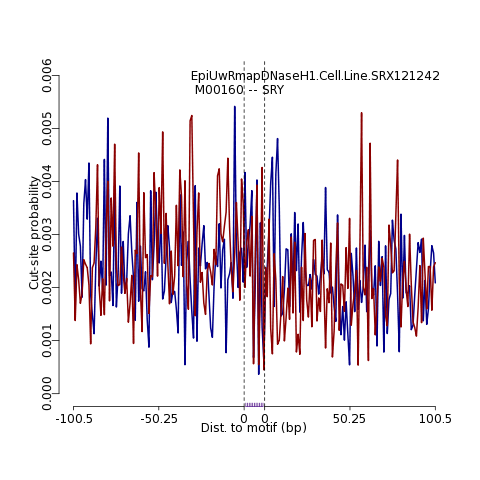

Supplement: S3 File — For each motif, footprint profiles are aggregated across all binding sites in all 653 DNase-seq samples. Color indicates which strand the motif matches, positive (blue) or negative (red). Text in the upper left denotes the tissue with the highest Z-score from the CENTIPEDE mode, the motif ID, and the corresponding transcription factor. (GZ) [file pgen.1005875.s004.tar.gz › recalibratedMotifShape/M00160.lambda.png]

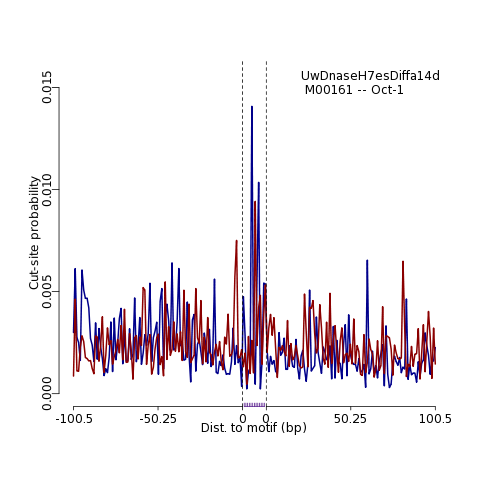

Supplement: S3 File — For each motif, footprint profiles are aggregated across all binding sites in all 653 DNase-seq samples. Color indicates which strand the motif matches, positive (blue) or negative (red). Text in the upper left denotes the tissue with the highest Z-score from the CENTIPEDE mode, the motif ID, and the corresponding transcription factor. (GZ) [file pgen.1005875.s004.tar.gz › recalibratedMotifShape/M00161.lambda.png]

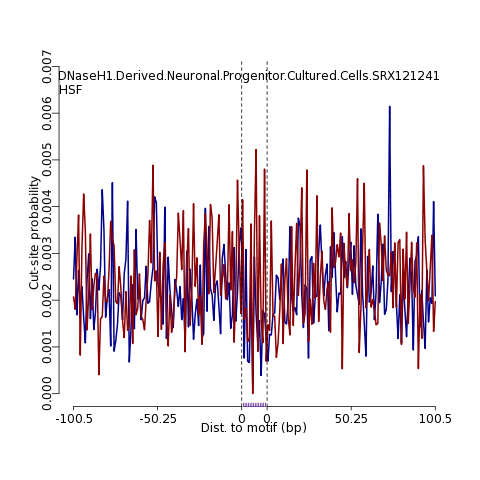

Supplement: S3 File — For each motif, footprint profiles are aggregated across all binding sites in all 653 DNase-seq samples. Color indicates which strand the motif matches, positive (blue) or negative (red). Text in the upper left denotes the tissue with the highest Z-score from the CENTIPEDE mode, the motif ID, and the corresponding transcription factor. (GZ) [file pgen.1005875.s004.tar.gz › recalibratedMotifShape/M00163.lambda.png]

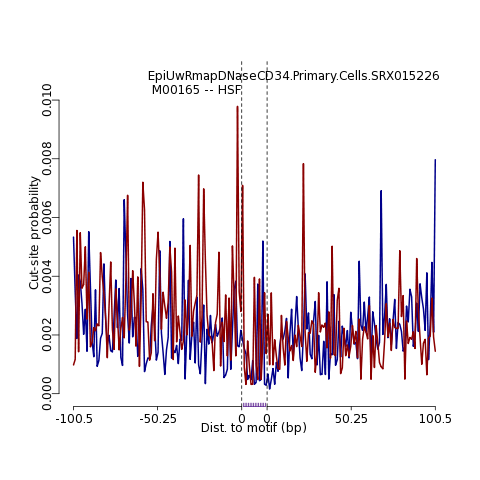

Supplement: S3 File — For each motif, footprint profiles are aggregated across all binding sites in all 653 DNase-seq samples. Color indicates which strand the motif matches, positive (blue) or negative (red). Text in the upper left denotes the tissue with the highest Z-score from the CENTIPEDE mode, the motif ID, and the corresponding transcription factor. (GZ) [file pgen.1005875.s004.tar.gz › recalibratedMotifShape/M00165.lambda.png]

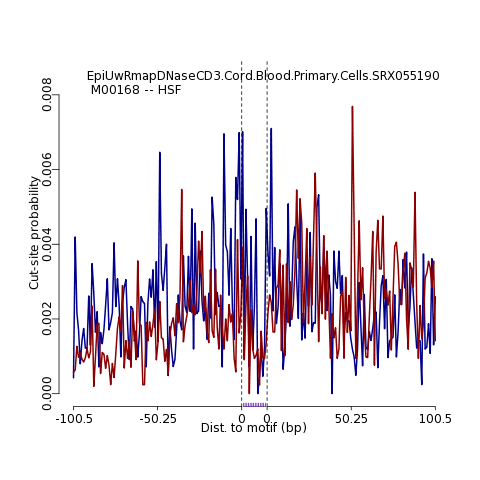

Supplement: S3 File — For each motif, footprint profiles are aggregated across all binding sites in all 653 DNase-seq samples. Color indicates which strand the motif matches, positive (blue) or negative (red). Text in the upper left denotes the tissue with the highest Z-score from the CENTIPEDE mode, the motif ID, and the corresponding transcription factor. (GZ) [file pgen.1005875.s004.tar.gz › recalibratedMotifShape/M00168.lambda.png]

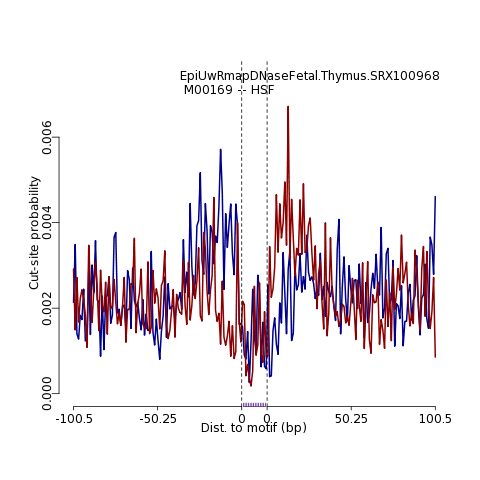

Supplement: S3 File — For each motif, footprint profiles are aggregated across all binding sites in all 653 DNase-seq samples. Color indicates which strand the motif matches, positive (blue) or negative (red). Text in the upper left denotes the tissue with the highest Z-score from the CENTIPEDE mode, the motif ID, and the corresponding transcription factor. (GZ) [file pgen.1005875.s004.tar.gz › recalibratedMotifShape/M00169.lambda.png]

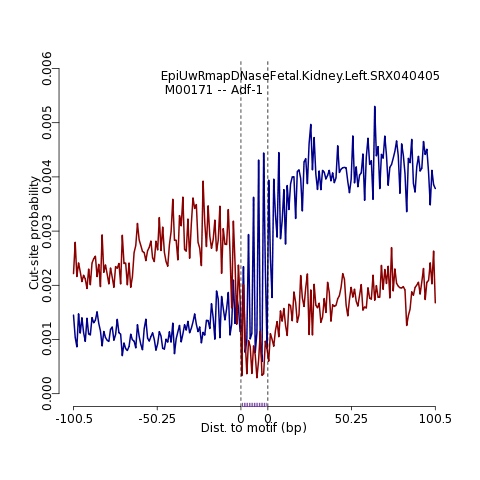

Supplement: S3 File — For each motif, footprint profiles are aggregated across all binding sites in all 653 DNase-seq samples. Color indicates which strand the motif matches, positive (blue) or negative (red). Text in the upper left denotes the tissue with the highest Z-score from the CENTIPEDE mode, the motif ID, and the corresponding transcription factor. (GZ) [file pgen.1005875.s004.tar.gz › recalibratedMotifShape/M00171.lambda.png]

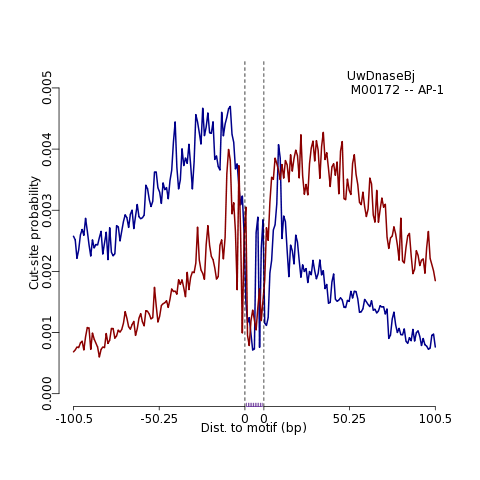

Supplement: S3 File — For each motif, footprint profiles are aggregated across all binding sites in all 653 DNase-seq samples. Color indicates which strand the motif matches, positive (blue) or negative (red). Text in the upper left denotes the tissue with the highest Z-score from the CENTIPEDE mode, the motif ID, and the corresponding transcription factor. (GZ) [file pgen.1005875.s004.tar.gz › recalibratedMotifShape/M00172.lambda.png]

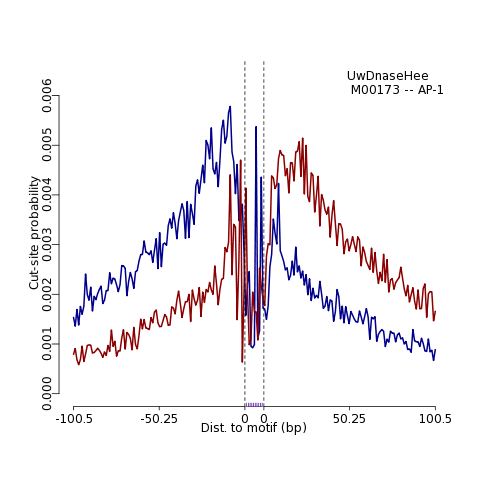

Supplement: S3 File — For each motif, footprint profiles are aggregated across all binding sites in all 653 DNase-seq samples. Color indicates which strand the motif matches, positive (blue) or negative (red). Text in the upper left denotes the tissue with the highest Z-score from the CENTIPEDE mode, the motif ID, and the corresponding transcription factor. (GZ) [file pgen.1005875.s004.tar.gz › recalibratedMotifShape/M00173.lambda.png]

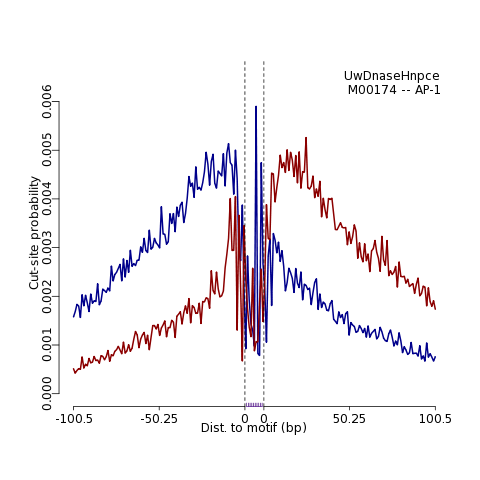

Supplement: S3 File — For each motif, footprint profiles are aggregated across all binding sites in all 653 DNase-seq samples. Color indicates which strand the motif matches, positive (blue) or negative (red). Text in the upper left denotes the tissue with the highest Z-score from the CENTIPEDE mode, the motif ID, and the corresponding transcription factor. (GZ) [file pgen.1005875.s004.tar.gz › recalibratedMotifShape/M00174.lambda.png]

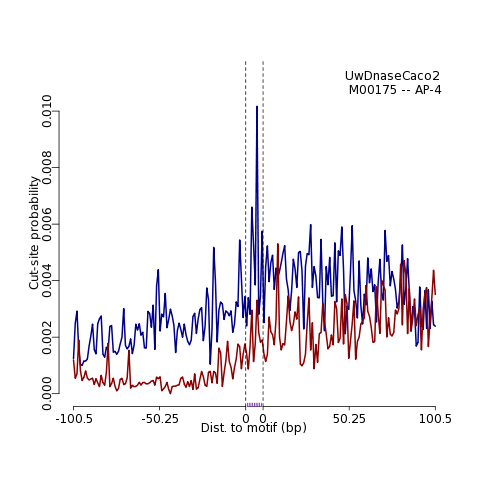

Supplement: S3 File — For each motif, footprint profiles are aggregated across all binding sites in all 653 DNase-seq samples. Color indicates which strand the motif matches, positive (blue) or negative (red). Text in the upper left denotes the tissue with the highest Z-score from the CENTIPEDE mode, the motif ID, and the corresponding transcription factor. (GZ) [file pgen.1005875.s004.tar.gz › recalibratedMotifShape/M00175.lambda.png]

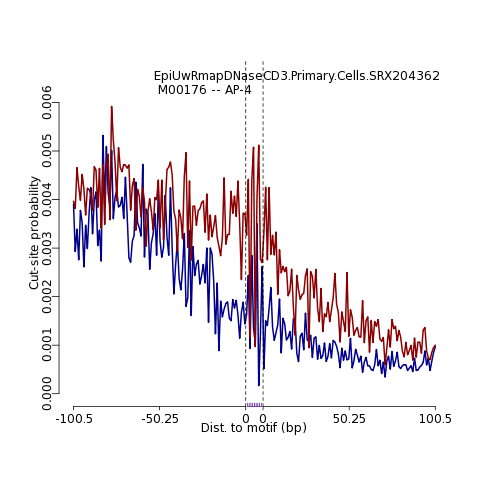

Supplement: S3 File — For each motif, footprint profiles are aggregated across all binding sites in all 653 DNase-seq samples. Color indicates which strand the motif matches, positive (blue) or negative (red). Text in the upper left denotes the tissue with the highest Z-score from the CENTIPEDE mode, the motif ID, and the corresponding transcription factor. (GZ) [file pgen.1005875.s004.tar.gz › recalibratedMotifShape/M00176.lambda.png]

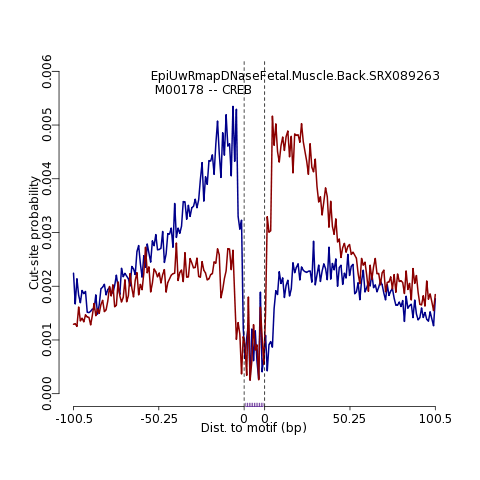

Supplement: S3 File — For each motif, footprint profiles are aggregated across all binding sites in all 653 DNase-seq samples. Color indicates which strand the motif matches, positive (blue) or negative (red). Text in the upper left denotes the tissue with the highest Z-score from the CENTIPEDE mode, the motif ID, and the corresponding transcription factor. (GZ) [file pgen.1005875.s004.tar.gz › recalibratedMotifShape/M00178.lambda.png]

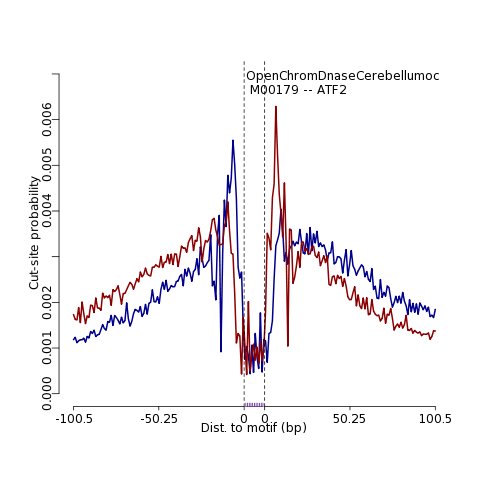

Supplement: S3 File — For each motif, footprint profiles are aggregated across all binding sites in all 653 DNase-seq samples. Color indicates which strand the motif matches, positive (blue) or negative (red). Text in the upper left denotes the tissue with the highest Z-score from the CENTIPEDE mode, the motif ID, and the corresponding transcription factor. (GZ) [file pgen.1005875.s004.tar.gz › recalibratedMotifShape/M00179.lambda.png]

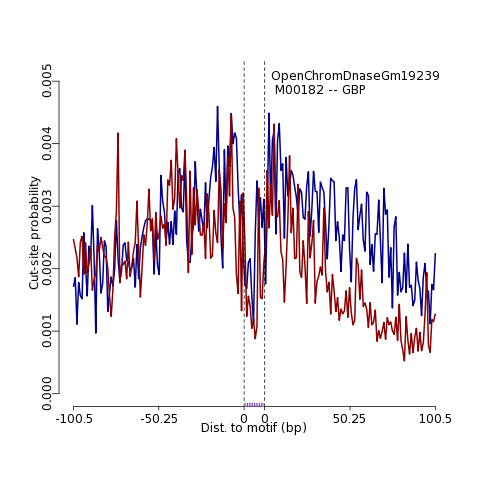

Supplement: S3 File — For each motif, footprint profiles are aggregated across all binding sites in all 653 DNase-seq samples. Color indicates which strand the motif matches, positive (blue) or negative (red). Text in the upper left denotes the tissue with the highest Z-score from the CENTIPEDE mode, the motif ID, and the corresponding transcription factor. (GZ) [file pgen.1005875.s004.tar.gz › recalibratedMotifShape/M00182.lambda.png]

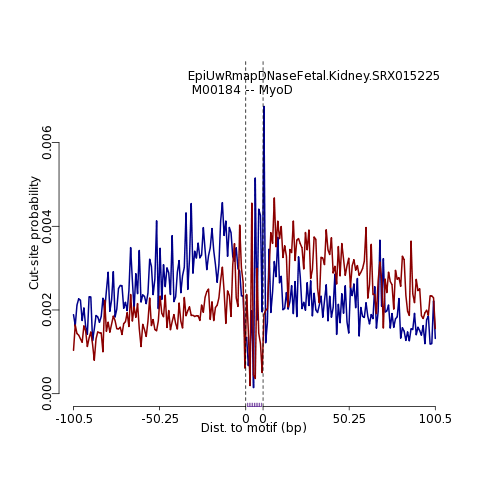

Supplement: S3 File — For each motif, footprint profiles are aggregated across all binding sites in all 653 DNase-seq samples. Color indicates which strand the motif matches, positive (blue) or negative (red). Text in the upper left denotes the tissue with the highest Z-score from the CENTIPEDE mode, the motif ID, and the corresponding transcription factor. (GZ) [file pgen.1005875.s004.tar.gz › recalibratedMotifShape/M00184.lambda.png]

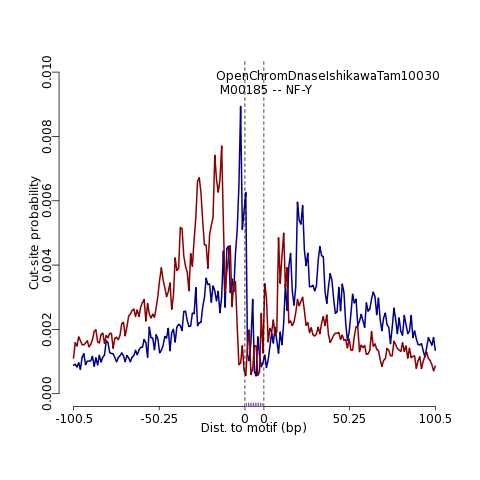

Supplement: S3 File — For each motif, footprint profiles are aggregated across all binding sites in all 653 DNase-seq samples. Color indicates which strand the motif matches, positive (blue) or negative (red). Text in the upper left denotes the tissue with the highest Z-score from the CENTIPEDE mode, the motif ID, and the corresponding transcription factor. (GZ) [file pgen.1005875.s004.tar.gz › recalibratedMotifShape/M00185.lambda.png]

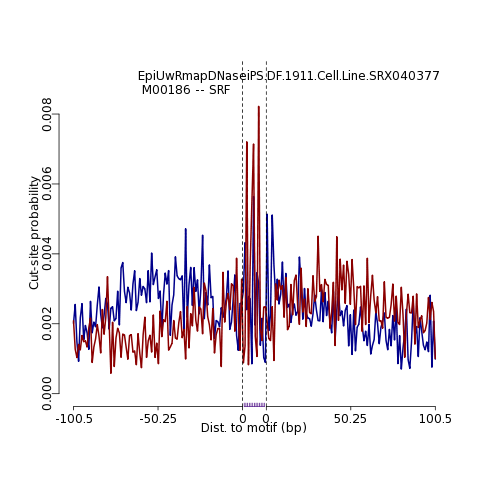

Supplement: S3 File — For each motif, footprint profiles are aggregated across all binding sites in all 653 DNase-seq samples. Color indicates which strand the motif matches, positive (blue) or negative (red). Text in the upper left denotes the tissue with the highest Z-score from the CENTIPEDE mode, the motif ID, and the corresponding transcription factor. (GZ) [file pgen.1005875.s004.tar.gz › recalibratedMotifShape/M00186.lambda.png]

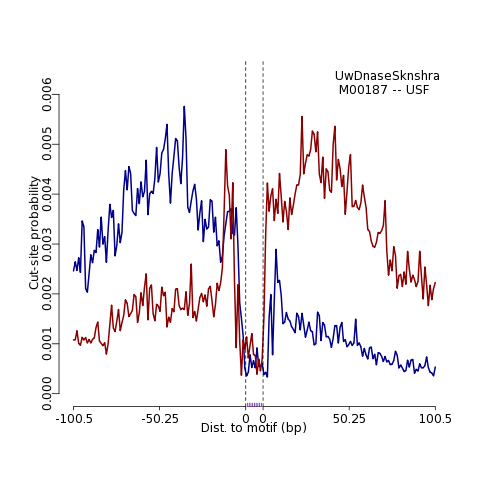

Supplement: S3 File — For each motif, footprint profiles are aggregated across all binding sites in all 653 DNase-seq samples. Color indicates which strand the motif matches, positive (blue) or negative (red). Text in the upper left denotes the tissue with the highest Z-score from the CENTIPEDE mode, the motif ID, and the corresponding transcription factor. (GZ) [file pgen.1005875.s004.tar.gz › recalibratedMotifShape/M00187.lambda.png]

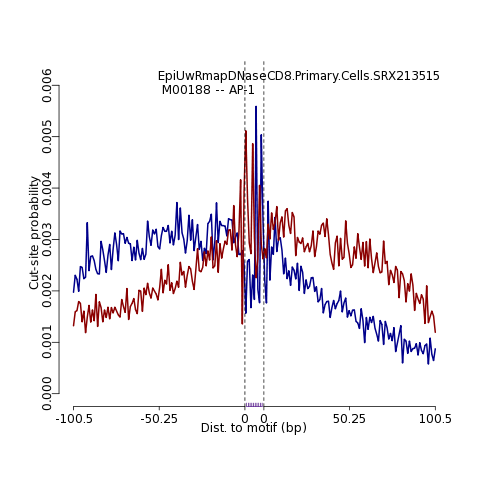

Supplement: S3 File — For each motif, footprint profiles are aggregated across all binding sites in all 653 DNase-seq samples. Color indicates which strand the motif matches, positive (blue) or negative (red). Text in the upper left denotes the tissue with the highest Z-score from the CENTIPEDE mode, the motif ID, and the corresponding transcription factor. (GZ) [file pgen.1005875.s004.tar.gz › recalibratedMotifShape/M00188.lambda.png]

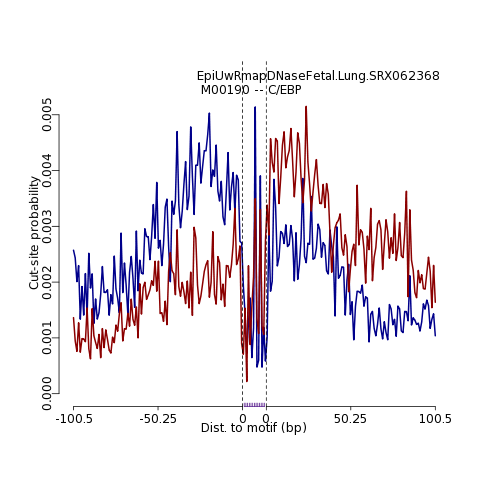

Supplement: S3 File — For each motif, footprint profiles are aggregated across all binding sites in all 653 DNase-seq samples. Color indicates which strand the motif matches, positive (blue) or negative (red). Text in the upper left denotes the tissue with the highest Z-score from the CENTIPEDE mode, the motif ID, and the corresponding transcription factor. (GZ) [file pgen.1005875.s004.tar.gz › recalibratedMotifShape/M00190.lambda.png]

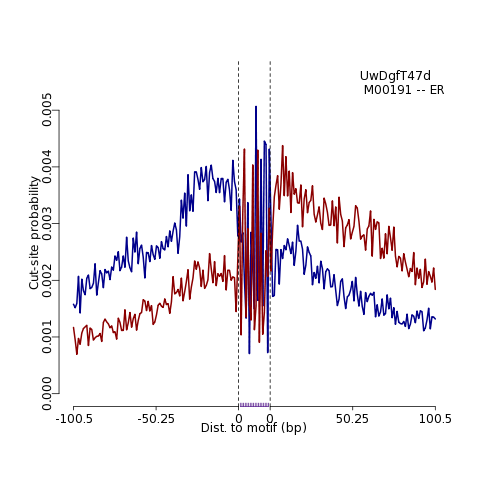

Supplement: S3 File — For each motif, footprint profiles are aggregated across all binding sites in all 653 DNase-seq samples. Color indicates which strand the motif matches, positive (blue) or negative (red). Text in the upper left denotes the tissue with the highest Z-score from the CENTIPEDE mode, the motif ID, and the corresponding transcription factor. (GZ) [file pgen.1005875.s004.tar.gz › recalibratedMotifShape/M00191.lambda.png]

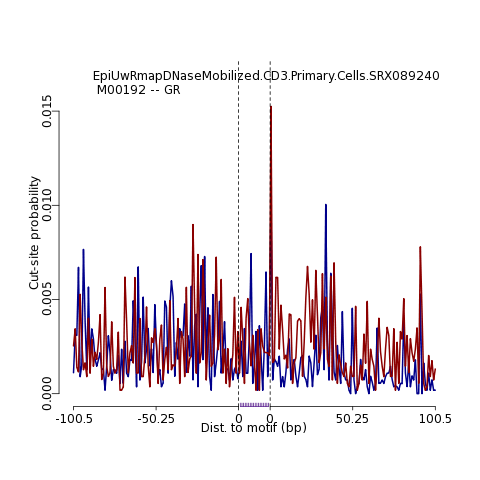

Supplement: S3 File — For each motif, footprint profiles are aggregated across all binding sites in all 653 DNase-seq samples. Color indicates which strand the motif matches, positive (blue) or negative (red). Text in the upper left denotes the tissue with the highest Z-score from the CENTIPEDE mode, the motif ID, and the corresponding transcription factor. (GZ) [file pgen.1005875.s004.tar.gz › recalibratedMotifShape/M00192.lambda.png]

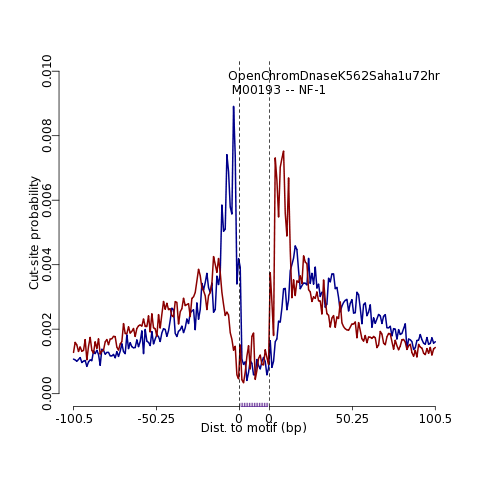

Supplement: S3 File — For each motif, footprint profiles are aggregated across all binding sites in all 653 DNase-seq samples. Color indicates which strand the motif matches, positive (blue) or negative (red). Text in the upper left denotes the tissue with the highest Z-score from the CENTIPEDE mode, the motif ID, and the corresponding transcription factor. (GZ) [file pgen.1005875.s004.tar.gz › recalibratedMotifShape/M00193.lambda.png]

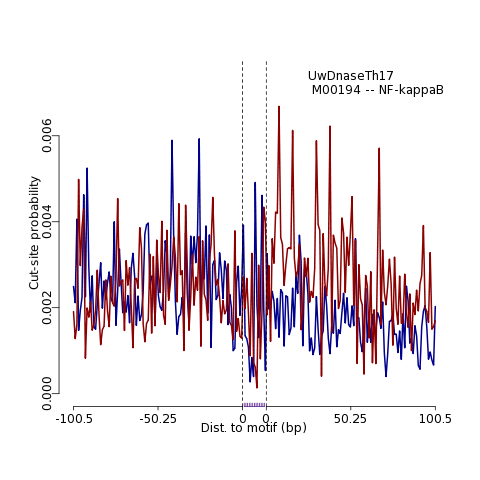

Supplement: S3 File — For each motif, footprint profiles are aggregated across all binding sites in all 653 DNase-seq samples. Color indicates which strand the motif matches, positive (blue) or negative (red). Text in the upper left denotes the tissue with the highest Z-score from the CENTIPEDE mode, the motif ID, and the corresponding transcription factor. (GZ) [file pgen.1005875.s004.tar.gz › recalibratedMotifShape/M00194.lambda.png]

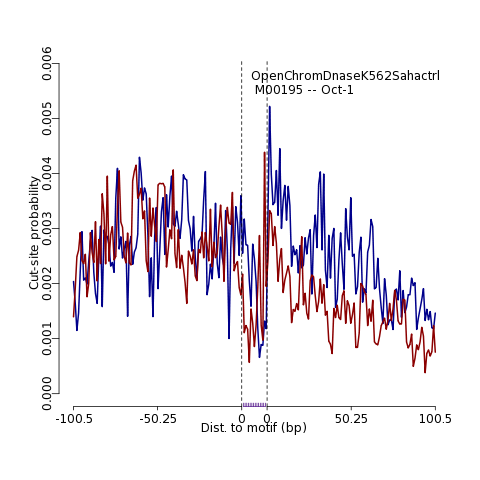

Supplement: S3 File — For each motif, footprint profiles are aggregated across all binding sites in all 653 DNase-seq samples. Color indicates which strand the motif matches, positive (blue) or negative (red). Text in the upper left denotes the tissue with the highest Z-score from the CENTIPEDE mode, the motif ID, and the corresponding transcription factor. (GZ) [file pgen.1005875.s004.tar.gz › recalibratedMotifShape/M00195.lambda.png]

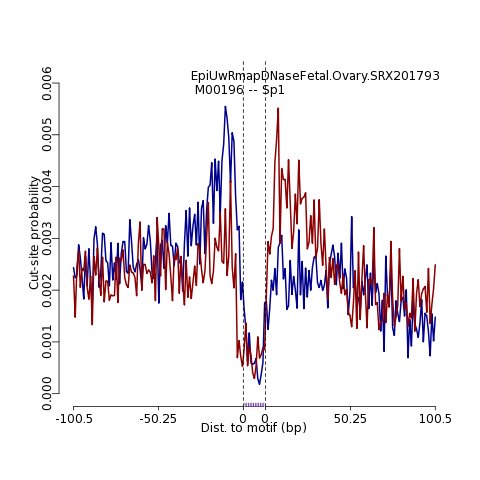

Supplement: S3 File — For each motif, footprint profiles are aggregated across all binding sites in all 653 DNase-seq samples. Color indicates which strand the motif matches, positive (blue) or negative (red). Text in the upper left denotes the tissue with the highest Z-score from the CENTIPEDE mode, the motif ID, and the corresponding transcription factor. (GZ) [file pgen.1005875.s004.tar.gz › recalibratedMotifShape/M00196.lambda.png]

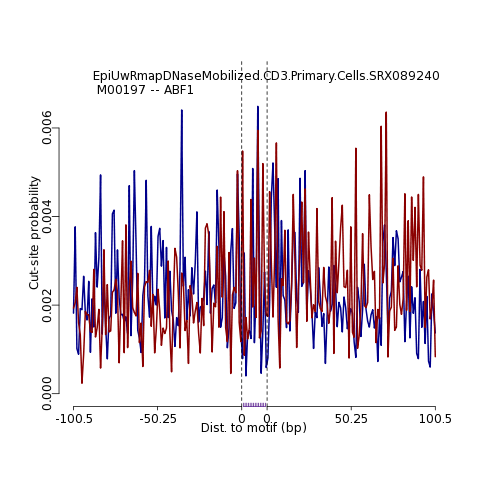

Supplement: S3 File — For each motif, footprint profiles are aggregated across all binding sites in all 653 DNase-seq samples. Color indicates which strand the motif matches, positive (blue) or negative (red). Text in the upper left denotes the tissue with the highest Z-score from the CENTIPEDE mode, the motif ID, and the corresponding transcription factor. (GZ) [file pgen.1005875.s004.tar.gz › recalibratedMotifShape/M00197.lambda.png]

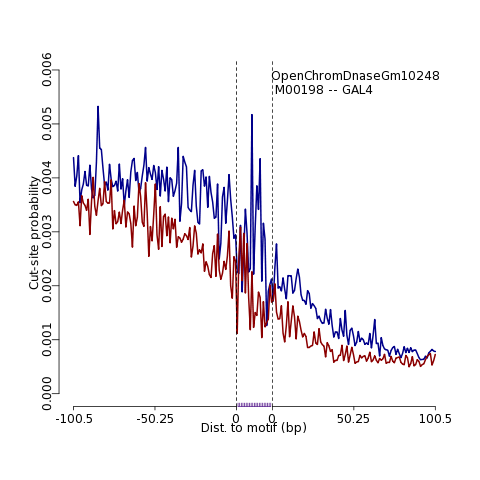

Supplement: S3 File — For each motif, footprint profiles are aggregated across all binding sites in all 653 DNase-seq samples. Color indicates which strand the motif matches, positive (blue) or negative (red). Text in the upper left denotes the tissue with the highest Z-score from the CENTIPEDE mode, the motif ID, and the corresponding transcription factor. (GZ) [file pgen.1005875.s004.tar.gz › recalibratedMotifShape/M00198.lambda.png]

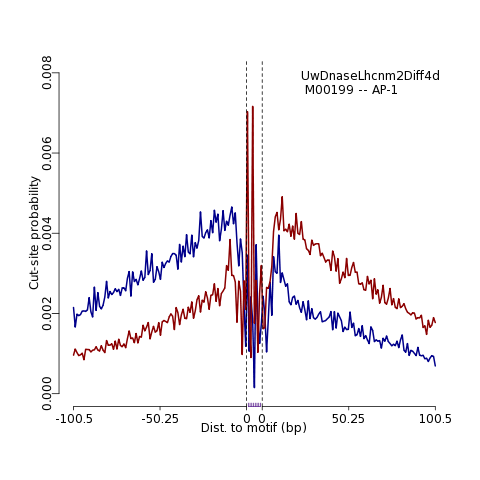

Supplement: S3 File — For each motif, footprint profiles are aggregated across all binding sites in all 653 DNase-seq samples. Color indicates which strand the motif matches, positive (blue) or negative (red). Text in the upper left denotes the tissue with the highest Z-score from the CENTIPEDE mode, the motif ID, and the corresponding transcription factor. (GZ) [file pgen.1005875.s004.tar.gz › recalibratedMotifShape/M00199.lambda.png]

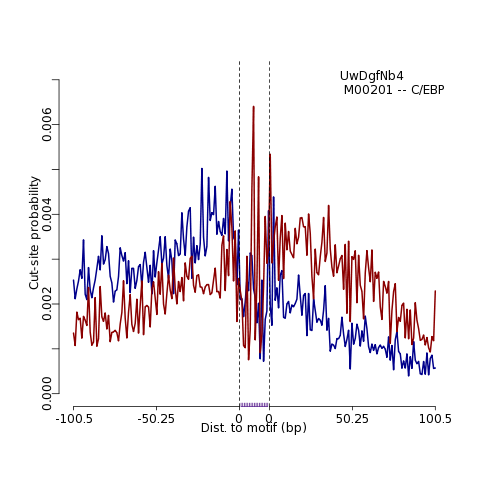

Supplement: S3 File — For each motif, footprint profiles are aggregated across all binding sites in all 653 DNase-seq samples. Color indicates which strand the motif matches, positive (blue) or negative (red). Text in the upper left denotes the tissue with the highest Z-score from the CENTIPEDE mode, the motif ID, and the corresponding transcription factor. (GZ) [file pgen.1005875.s004.tar.gz › recalibratedMotifShape/M00201.lambda.png]

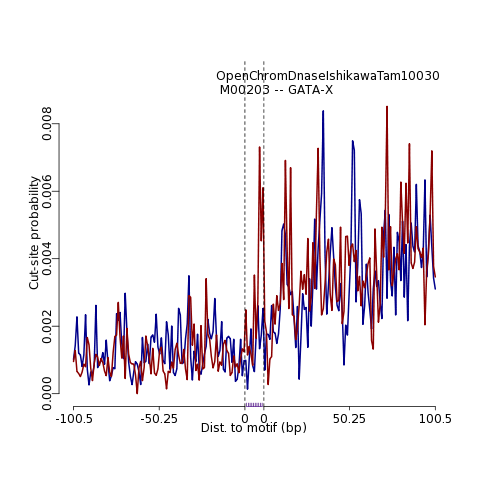

Supplement: S3 File — For each motif, footprint profiles are aggregated across all binding sites in all 653 DNase-seq samples. Color indicates which strand the motif matches, positive (blue) or negative (red). Text in the upper left denotes the tissue with the highest Z-score from the CENTIPEDE mode, the motif ID, and the corresponding transcription factor. (GZ) [file pgen.1005875.s004.tar.gz › recalibratedMotifShape/M00203.lambda.png]

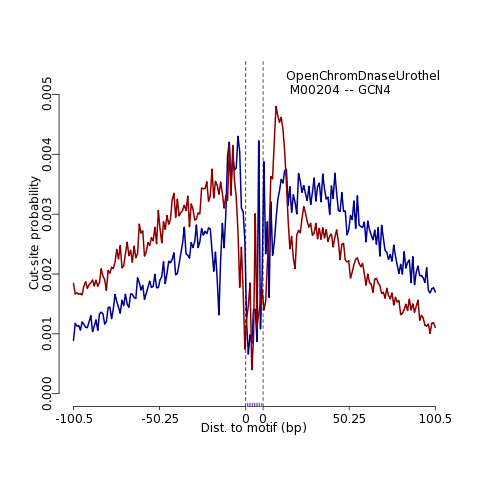

Supplement: S3 File — For each motif, footprint profiles are aggregated across all binding sites in all 653 DNase-seq samples. Color indicates which strand the motif matches, positive (blue) or negative (red). Text in the upper left denotes the tissue with the highest Z-score from the CENTIPEDE mode, the motif ID, and the corresponding transcription factor. (GZ) [file pgen.1005875.s004.tar.gz › recalibratedMotifShape/M00204.lambda.png]

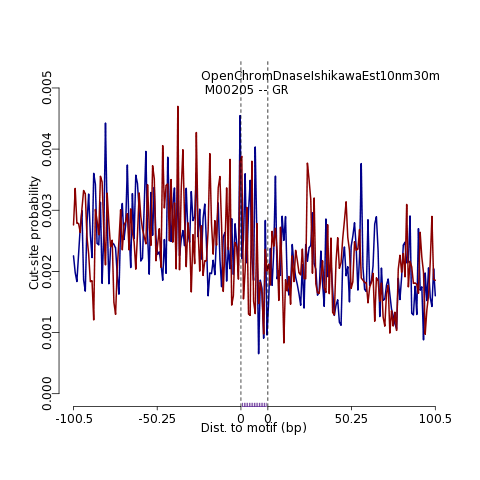

Supplement: S3 File — For each motif, footprint profiles are aggregated across all binding sites in all 653 DNase-seq samples. Color indicates which strand the motif matches, positive (blue) or negative (red). Text in the upper left denotes the tissue with the highest Z-score from the CENTIPEDE mode, the motif ID, and the corresponding transcription factor. (GZ) [file pgen.1005875.s004.tar.gz › recalibratedMotifShape/M00205.lambda.png]

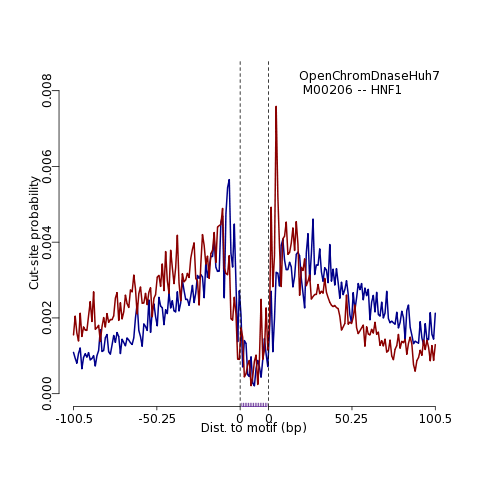

Supplement: S3 File — For each motif, footprint profiles are aggregated across all binding sites in all 653 DNase-seq samples. Color indicates which strand the motif matches, positive (blue) or negative (red). Text in the upper left denotes the tissue with the highest Z-score from the CENTIPEDE mode, the motif ID, and the corresponding transcription factor. (GZ) [file pgen.1005875.s004.tar.gz › recalibratedMotifShape/M00206.lambda.png]

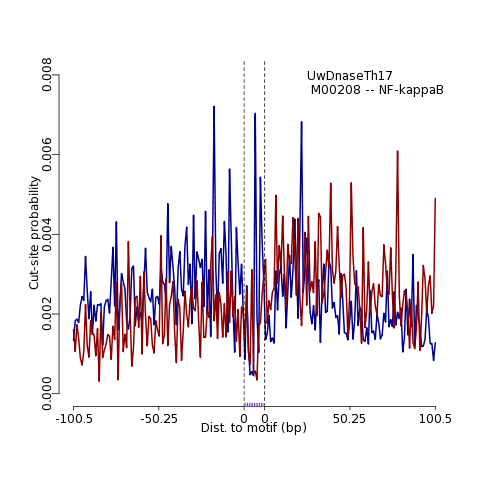

Supplement: S3 File — For each motif, footprint profiles are aggregated across all binding sites in all 653 DNase-seq samples. Color indicates which strand the motif matches, positive (blue) or negative (red). Text in the upper left denotes the tissue with the highest Z-score from the CENTIPEDE mode, the motif ID, and the corresponding transcription factor. (GZ) [file pgen.1005875.s004.tar.gz › recalibratedMotifShape/M00208.lambda.png]

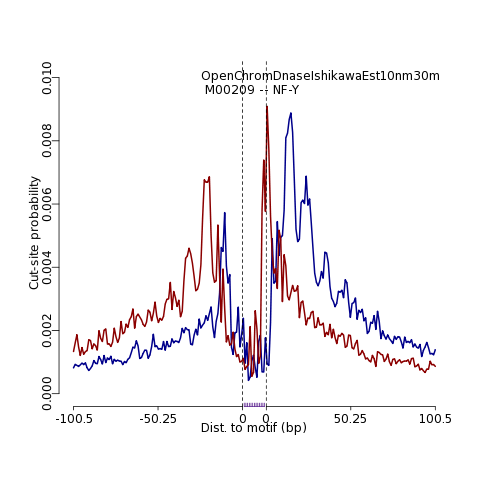

Supplement: S3 File — For each motif, footprint profiles are aggregated across all binding sites in all 653 DNase-seq samples. Color indicates which strand the motif matches, positive (blue) or negative (red). Text in the upper left denotes the tissue with the highest Z-score from the CENTIPEDE mode, the motif ID, and the corresponding transcription factor. (GZ) [file pgen.1005875.s004.tar.gz › recalibratedMotifShape/M00209.lambda.png]

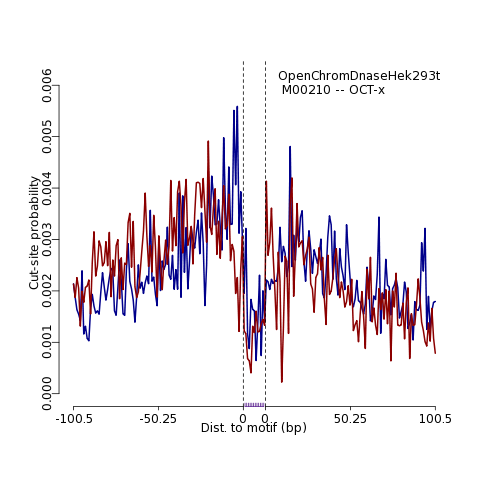

Supplement: S3 File — For each motif, footprint profiles are aggregated across all binding sites in all 653 DNase-seq samples. Color indicates which strand the motif matches, positive (blue) or negative (red). Text in the upper left denotes the tissue with the highest Z-score from the CENTIPEDE mode, the motif ID, and the corresponding transcription factor. (GZ) [file pgen.1005875.s004.tar.gz › recalibratedMotifShape/M00210.lambda.png]

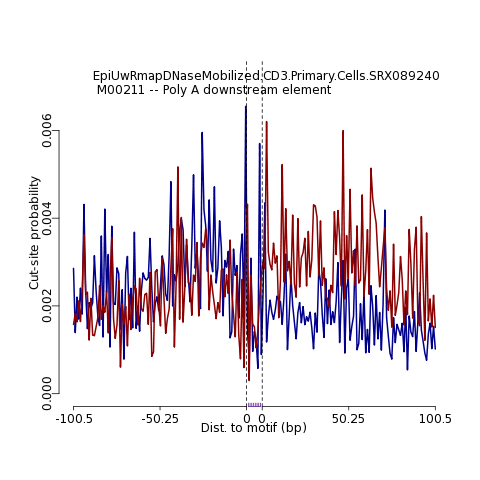

Supplement: S3 File — For each motif, footprint profiles are aggregated across all binding sites in all 653 DNase-seq samples. Color indicates which strand the motif matches, positive (blue) or negative (red). Text in the upper left denotes the tissue with the highest Z-score from the CENTIPEDE mode, the motif ID, and the corresponding transcription factor. (GZ) [file pgen.1005875.s004.tar.gz › recalibratedMotifShape/M00211.lambda.png]

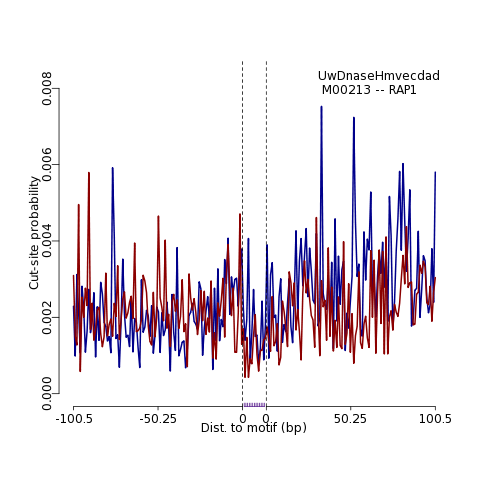

Supplement: S3 File — For each motif, footprint profiles are aggregated across all binding sites in all 653 DNase-seq samples. Color indicates which strand the motif matches, positive (blue) or negative (red). Text in the upper left denotes the tissue with the highest Z-score from the CENTIPEDE mode, the motif ID, and the corresponding transcription factor. (GZ) [file pgen.1005875.s004.tar.gz › recalibratedMotifShape/M00213.lambda.png]

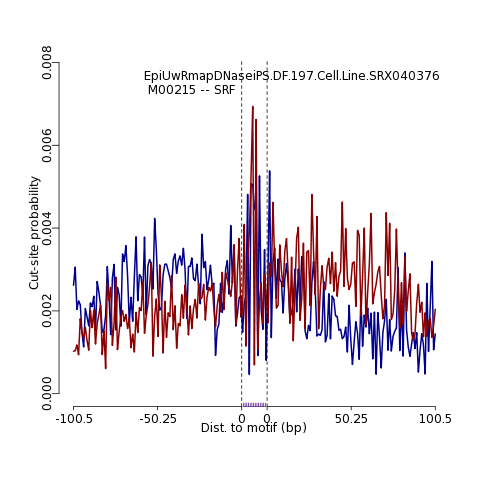

Supplement: S3 File — For each motif, footprint profiles are aggregated across all binding sites in all 653 DNase-seq samples. Color indicates which strand the motif matches, positive (blue) or negative (red). Text in the upper left denotes the tissue with the highest Z-score from the CENTIPEDE mode, the motif ID, and the corresponding transcription factor. (GZ) [file pgen.1005875.s004.tar.gz › recalibratedMotifShape/M00215.lambda.png]

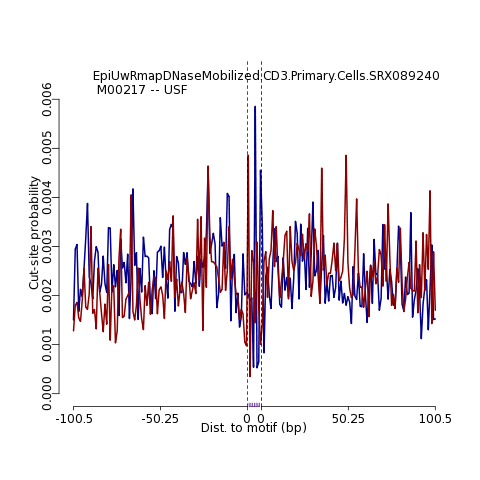

Supplement: S3 File — For each motif, footprint profiles are aggregated across all binding sites in all 653 DNase-seq samples. Color indicates which strand the motif matches, positive (blue) or negative (red). Text in the upper left denotes the tissue with the highest Z-score from the CENTIPEDE mode, the motif ID, and the corresponding transcription factor. (GZ) [file pgen.1005875.s004.tar.gz › recalibratedMotifShape/M00217.lambda.png]

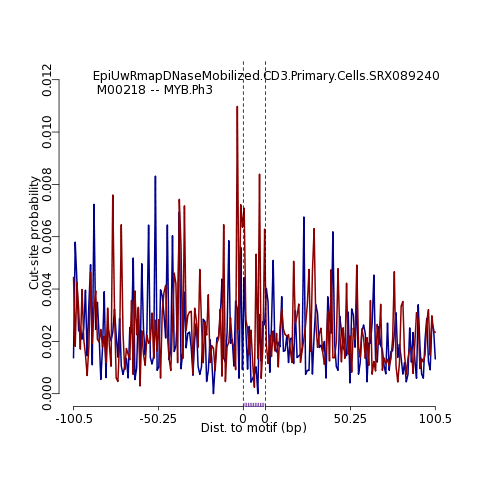

Supplement: S3 File — For each motif, footprint profiles are aggregated across all binding sites in all 653 DNase-seq samples. Color indicates which strand the motif matches, positive (blue) or negative (red). Text in the upper left denotes the tissue with the highest Z-score from the CENTIPEDE mode, the motif ID, and the corresponding transcription factor. (GZ) [file pgen.1005875.s004.tar.gz › recalibratedMotifShape/M00218.lambda.png]

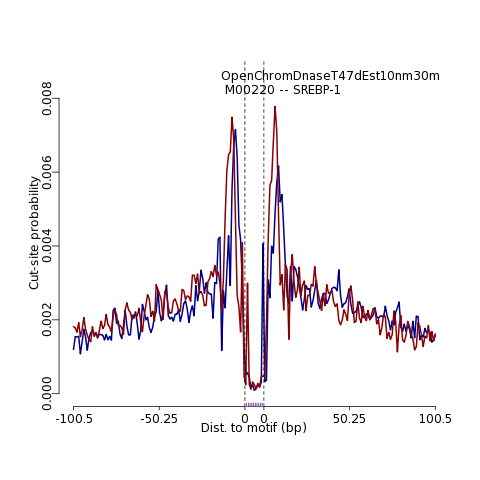

Supplement: S3 File — For each motif, footprint profiles are aggregated across all binding sites in all 653 DNase-seq samples. Color indicates which strand the motif matches, positive (blue) or negative (red). Text in the upper left denotes the tissue with the highest Z-score from the CENTIPEDE mode, the motif ID, and the corresponding transcription factor. (GZ) [file pgen.1005875.s004.tar.gz › recalibratedMotifShape/M00220.lambda.png]

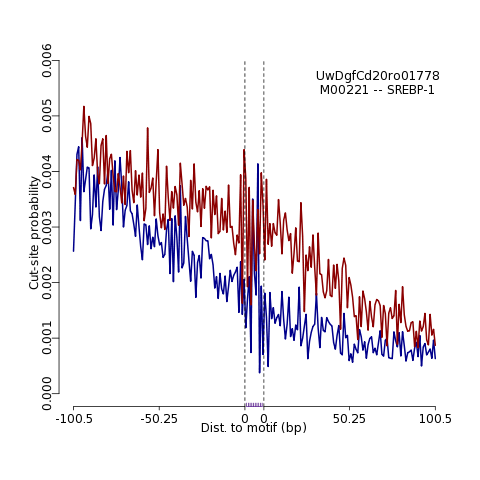

Supplement: S3 File — For each motif, footprint profiles are aggregated across all binding sites in all 653 DNase-seq samples. Color indicates which strand the motif matches, positive (blue) or negative (red). Text in the upper left denotes the tissue with the highest Z-score from the CENTIPEDE mode, the motif ID, and the corresponding transcription factor. (GZ) [file pgen.1005875.s004.tar.gz › recalibratedMotifShape/M00221.lambda.png]

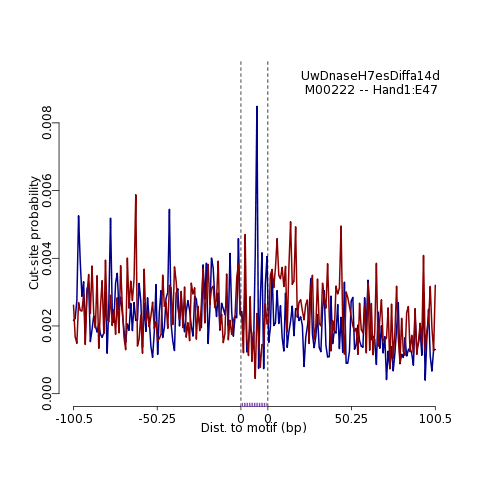

Supplement: S3 File — For each motif, footprint profiles are aggregated across all binding sites in all 653 DNase-seq samples. Color indicates which strand the motif matches, positive (blue) or negative (red). Text in the upper left denotes the tissue with the highest Z-score from the CENTIPEDE mode, the motif ID, and the corresponding transcription factor. (GZ) [file pgen.1005875.s004.tar.gz › recalibratedMotifShape/M00222.lambda.png]

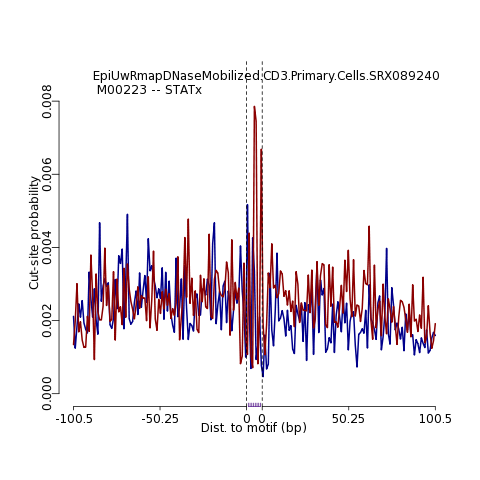

Supplement: S3 File — For each motif, footprint profiles are aggregated across all binding sites in all 653 DNase-seq samples. Color indicates which strand the motif matches, positive (blue) or negative (red). Text in the upper left denotes the tissue with the highest Z-score from the CENTIPEDE mode, the motif ID, and the corresponding transcription factor. (GZ) [file pgen.1005875.s004.tar.gz › recalibratedMotifShape/M00223.lambda.png]

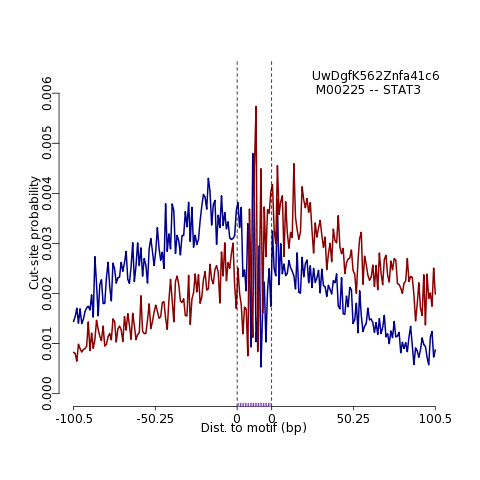

Supplement: S3 File — For each motif, footprint profiles are aggregated across all binding sites in all 653 DNase-seq samples. Color indicates which strand the motif matches, positive (blue) or negative (red). Text in the upper left denotes the tissue with the highest Z-score from the CENTIPEDE mode, the motif ID, and the corresponding transcription factor. (GZ) [file pgen.1005875.s004.tar.gz › recalibratedMotifShape/M00225.lambda.png]

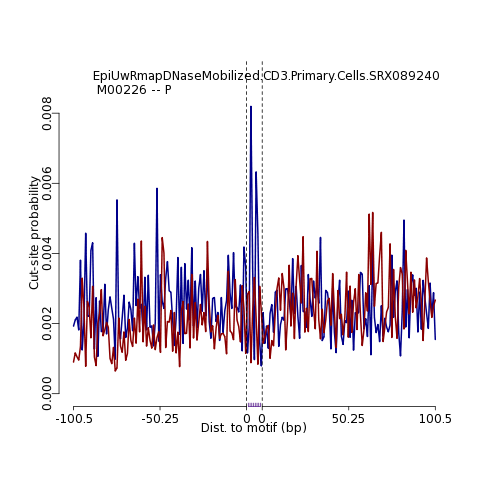

Supplement: S3 File — For each motif, footprint profiles are aggregated across all binding sites in all 653 DNase-seq samples. Color indicates which strand the motif matches, positive (blue) or negative (red). Text in the upper left denotes the tissue with the highest Z-score from the CENTIPEDE mode, the motif ID, and the corresponding transcription factor. (GZ) [file pgen.1005875.s004.tar.gz › recalibratedMotifShape/M00226.lambda.png]

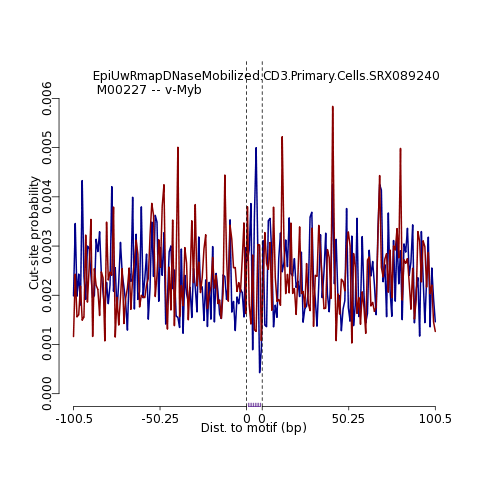

Supplement: S3 File — For each motif, footprint profiles are aggregated across all binding sites in all 653 DNase-seq samples. Color indicates which strand the motif matches, positive (blue) or negative (red). Text in the upper left denotes the tissue with the highest Z-score from the CENTIPEDE mode, the motif ID, and the corresponding transcription factor. (GZ) [file pgen.1005875.s004.tar.gz › recalibratedMotifShape/M00227.lambda.png]

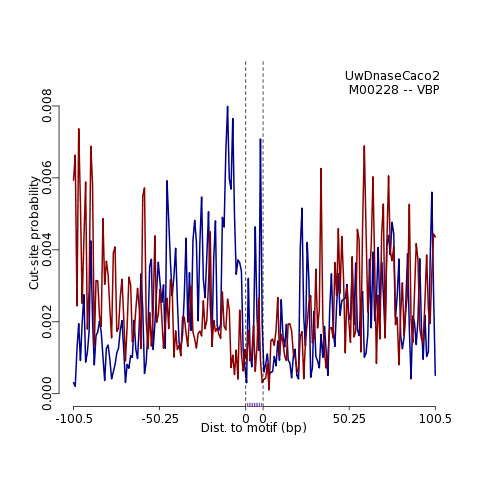

Supplement: S3 File — For each motif, footprint profiles are aggregated across all binding sites in all 653 DNase-seq samples. Color indicates which strand the motif matches, positive (blue) or negative (red). Text in the upper left denotes the tissue with the highest Z-score from the CENTIPEDE mode, the motif ID, and the corresponding transcription factor. (GZ) [file pgen.1005875.s004.tar.gz › recalibratedMotifShape/M00228.lambda.png]

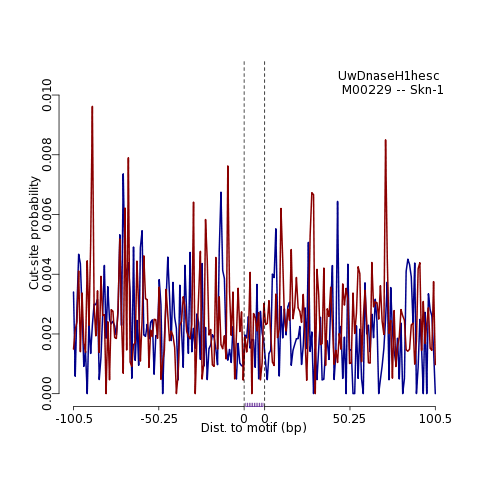

Supplement: S3 File — For each motif, footprint profiles are aggregated across all binding sites in all 653 DNase-seq samples. Color indicates which strand the motif matches, positive (blue) or negative (red). Text in the upper left denotes the tissue with the highest Z-score from the CENTIPEDE mode, the motif ID, and the corresponding transcription factor. (GZ) [file pgen.1005875.s004.tar.gz › recalibratedMotifShape/M00229.lambda.png]

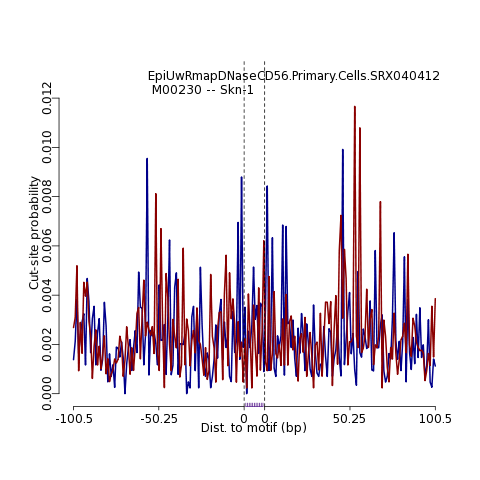

Supplement: S3 File — For each motif, footprint profiles are aggregated across all binding sites in all 653 DNase-seq samples. Color indicates which strand the motif matches, positive (blue) or negative (red). Text in the upper left denotes the tissue with the highest Z-score from the CENTIPEDE mode, the motif ID, and the corresponding transcription factor. (GZ) [file pgen.1005875.s004.tar.gz › recalibratedMotifShape/M00230.lambda.png]

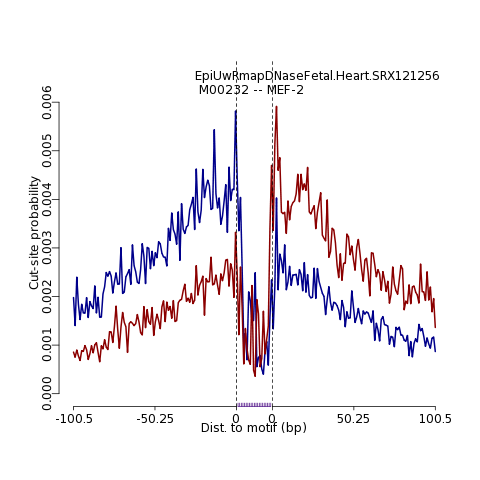

Supplement: S3 File — For each motif, footprint profiles are aggregated across all binding sites in all 653 DNase-seq samples. Color indicates which strand the motif matches, positive (blue) or negative (red). Text in the upper left denotes the tissue with the highest Z-score from the CENTIPEDE mode, the motif ID, and the corresponding transcription factor. (GZ) [file pgen.1005875.s004.tar.gz › recalibratedMotifShape/M00232.lambda.png]

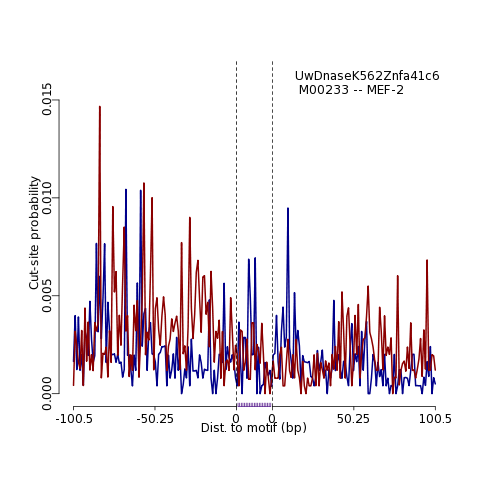

Supplement: S3 File — For each motif, footprint profiles are aggregated across all binding sites in all 653 DNase-seq samples. Color indicates which strand the motif matches, positive (blue) or negative (red). Text in the upper left denotes the tissue with the highest Z-score from the CENTIPEDE mode, the motif ID, and the corresponding transcription factor. (GZ) [file pgen.1005875.s004.tar.gz › recalibratedMotifShape/M00233.lambda.png]

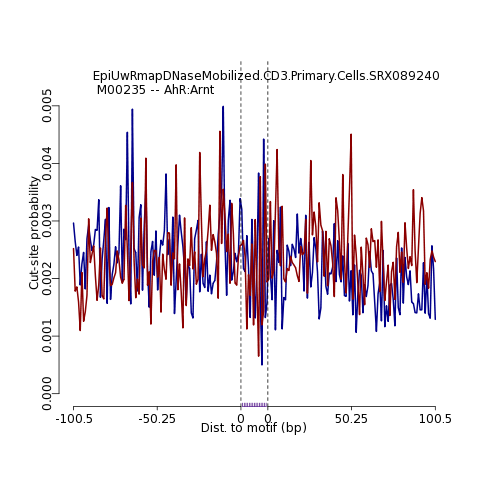

Supplement: S3 File — For each motif, footprint profiles are aggregated across all binding sites in all 653 DNase-seq samples. Color indicates which strand the motif matches, positive (blue) or negative (red). Text in the upper left denotes the tissue with the highest Z-score from the CENTIPEDE mode, the motif ID, and the corresponding transcription factor. (GZ) [file pgen.1005875.s004.tar.gz › recalibratedMotifShape/M00235.lambda.png]

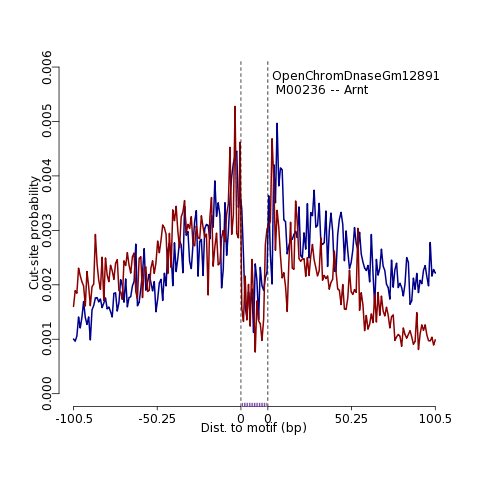

Supplement: S3 File — For each motif, footprint profiles are aggregated across all binding sites in all 653 DNase-seq samples. Color indicates which strand the motif matches, positive (blue) or negative (red). Text in the upper left denotes the tissue with the highest Z-score from the CENTIPEDE mode, the motif ID, and the corresponding transcription factor. (GZ) [file pgen.1005875.s004.tar.gz › recalibratedMotifShape/M00236.lambda.png]

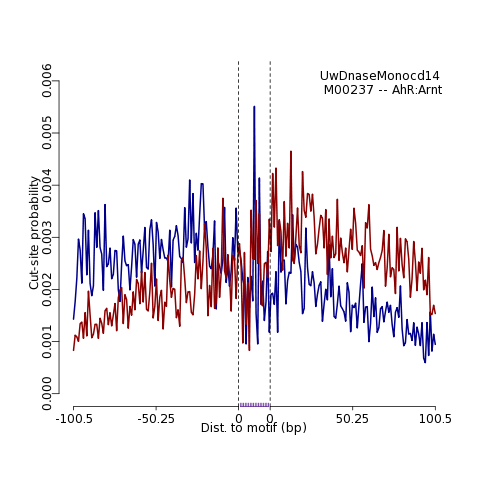

Supplement: S3 File — For each motif, footprint profiles are aggregated across all binding sites in all 653 DNase-seq samples. Color indicates which strand the motif matches, positive (blue) or negative (red). Text in the upper left denotes the tissue with the highest Z-score from the CENTIPEDE mode, the motif ID, and the corresponding transcription factor. (GZ) [file pgen.1005875.s004.tar.gz › recalibratedMotifShape/M00237.lambda.png]

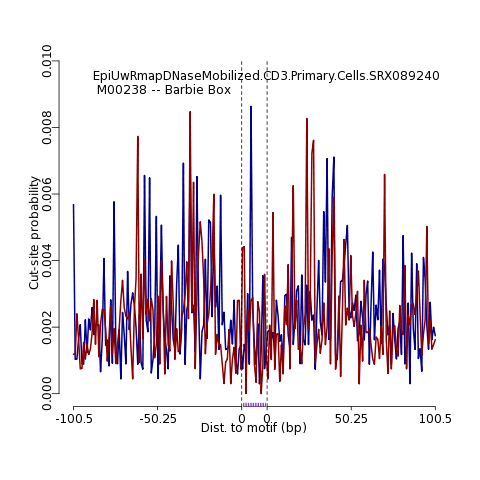

Supplement: S3 File — For each motif, footprint profiles are aggregated across all binding sites in all 653 DNase-seq samples. Color indicates which strand the motif matches, positive (blue) or negative (red). Text in the upper left denotes the tissue with the highest Z-score from the CENTIPEDE mode, the motif ID, and the corresponding transcription factor. (GZ) [file pgen.1005875.s004.tar.gz › recalibratedMotifShape/M00238.lambda.png]

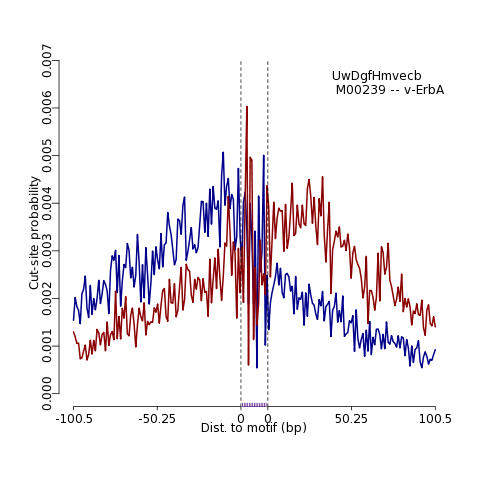

Supplement: S3 File — For each motif, footprint profiles are aggregated across all binding sites in all 653 DNase-seq samples. Color indicates which strand the motif matches, positive (blue) or negative (red). Text in the upper left denotes the tissue with the highest Z-score from the CENTIPEDE mode, the motif ID, and the corresponding transcription factor. (GZ) [file pgen.1005875.s004.tar.gz › recalibratedMotifShape/M00239.lambda.png]

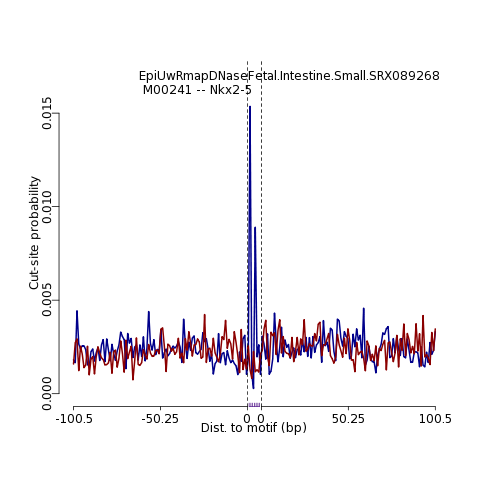

Supplement: S3 File — For each motif, footprint profiles are aggregated across all binding sites in all 653 DNase-seq samples. Color indicates which strand the motif matches, positive (blue) or negative (red). Text in the upper left denotes the tissue with the highest Z-score from the CENTIPEDE mode, the motif ID, and the corresponding transcription factor. (GZ) [file pgen.1005875.s004.tar.gz › recalibratedMotifShape/M00241.lambda.png]

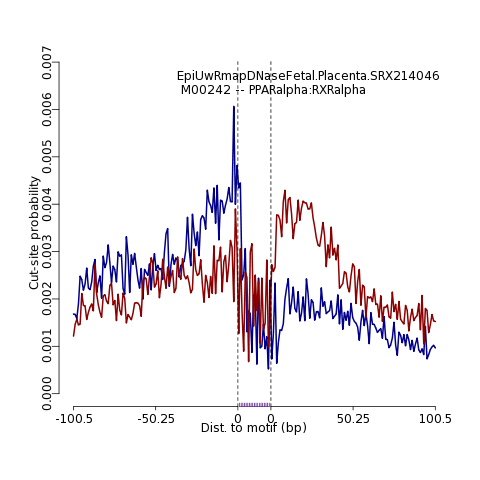

Supplement: S3 File — For each motif, footprint profiles are aggregated across all binding sites in all 653 DNase-seq samples. Color indicates which strand the motif matches, positive (blue) or negative (red). Text in the upper left denotes the tissue with the highest Z-score from the CENTIPEDE mode, the motif ID, and the corresponding transcription factor. (GZ) [file pgen.1005875.s004.tar.gz › recalibratedMotifShape/M00242.lambda.png]

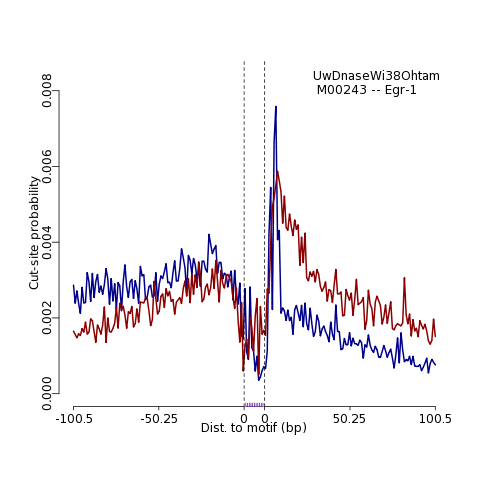

Supplement: S3 File — For each motif, footprint profiles are aggregated across all binding sites in all 653 DNase-seq samples. Color indicates which strand the motif matches, positive (blue) or negative (red). Text in the upper left denotes the tissue with the highest Z-score from the CENTIPEDE mode, the motif ID, and the corresponding transcription factor. (GZ) [file pgen.1005875.s004.tar.gz › recalibratedMotifShape/M00243.lambda.png]

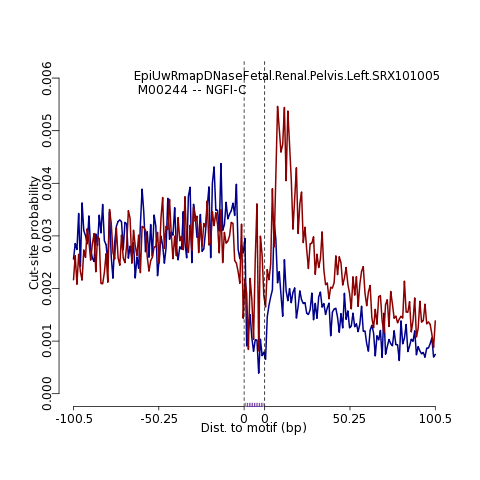

Supplement: S3 File — For each motif, footprint profiles are aggregated across all binding sites in all 653 DNase-seq samples. Color indicates which strand the motif matches, positive (blue) or negative (red). Text in the upper left denotes the tissue with the highest Z-score from the CENTIPEDE mode, the motif ID, and the corresponding transcription factor. (GZ) [file pgen.1005875.s004.tar.gz › recalibratedMotifShape/M00244.lambda.png]

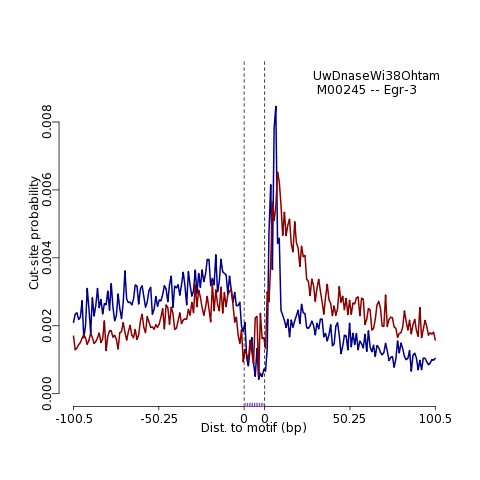

Supplement: S3 File — For each motif, footprint profiles are aggregated across all binding sites in all 653 DNase-seq samples. Color indicates which strand the motif matches, positive (blue) or negative (red). Text in the upper left denotes the tissue with the highest Z-score from the CENTIPEDE mode, the motif ID, and the corresponding transcription factor. (GZ) [file pgen.1005875.s004.tar.gz › recalibratedMotifShape/M00245.lambda.png]

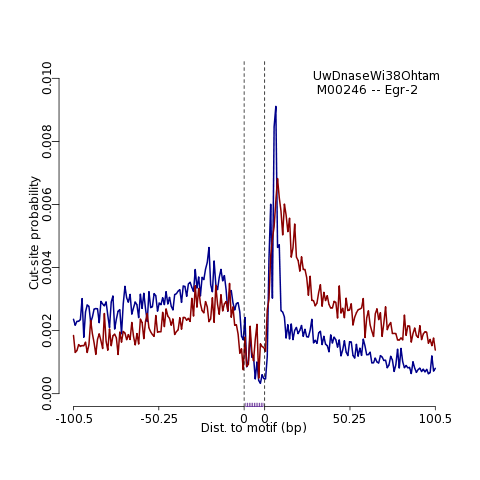

Supplement: S3 File — For each motif, footprint profiles are aggregated across all binding sites in all 653 DNase-seq samples. Color indicates which strand the motif matches, positive (blue) or negative (red). Text in the upper left denotes the tissue with the highest Z-score from the CENTIPEDE mode, the motif ID, and the corresponding transcription factor. (GZ) [file pgen.1005875.s004.tar.gz › recalibratedMotifShape/M00246.lambda.png]

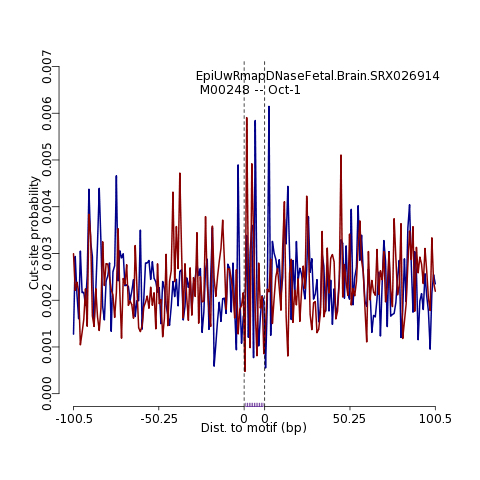

Supplement: S3 File — For each motif, footprint profiles are aggregated across all binding sites in all 653 DNase-seq samples. Color indicates which strand the motif matches, positive (blue) or negative (red). Text in the upper left denotes the tissue with the highest Z-score from the CENTIPEDE mode, the motif ID, and the corresponding transcription factor. (GZ) [file pgen.1005875.s004.tar.gz › recalibratedMotifShape/M00248.lambda.png]

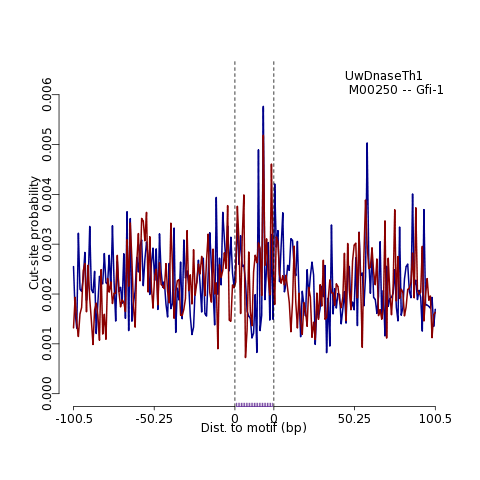

Supplement: S3 File — For each motif, footprint profiles are aggregated across all binding sites in all 653 DNase-seq samples. Color indicates which strand the motif matches, positive (blue) or negative (red). Text in the upper left denotes the tissue with the highest Z-score from the CENTIPEDE mode, the motif ID, and the corresponding transcription factor. (GZ) [file pgen.1005875.s004.tar.gz › recalibratedMotifShape/M00250.lambda.png]

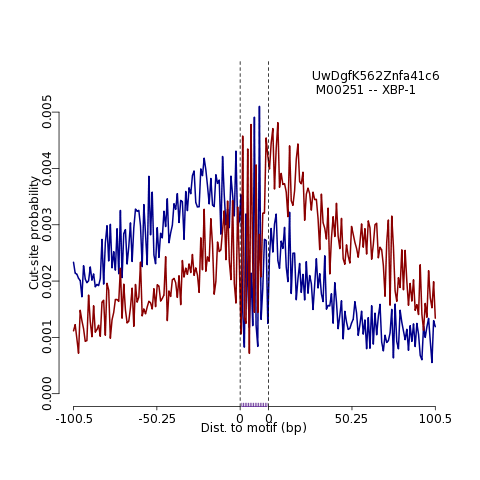

Supplement: S3 File — For each motif, footprint profiles are aggregated across all binding sites in all 653 DNase-seq samples. Color indicates which strand the motif matches, positive (blue) or negative (red). Text in the upper left denotes the tissue with the highest Z-score from the CENTIPEDE mode, the motif ID, and the corresponding transcription factor. (GZ) [file pgen.1005875.s004.tar.gz › recalibratedMotifShape/M00251.lambda.png]

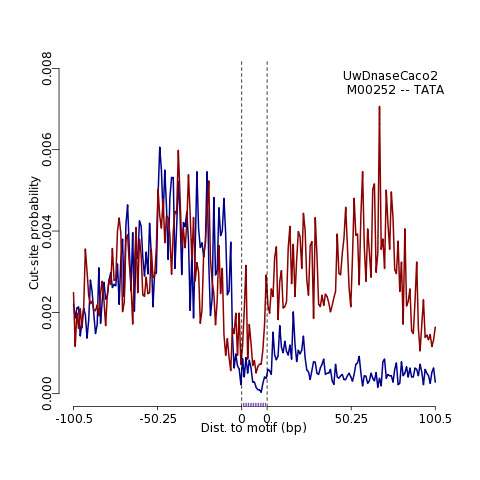

Supplement: S3 File — For each motif, footprint profiles are aggregated across all binding sites in all 653 DNase-seq samples. Color indicates which strand the motif matches, positive (blue) or negative (red). Text in the upper left denotes the tissue with the highest Z-score from the CENTIPEDE mode, the motif ID, and the corresponding transcription factor. (GZ) [file pgen.1005875.s004.tar.gz › recalibratedMotifShape/M00252.lambda.png]

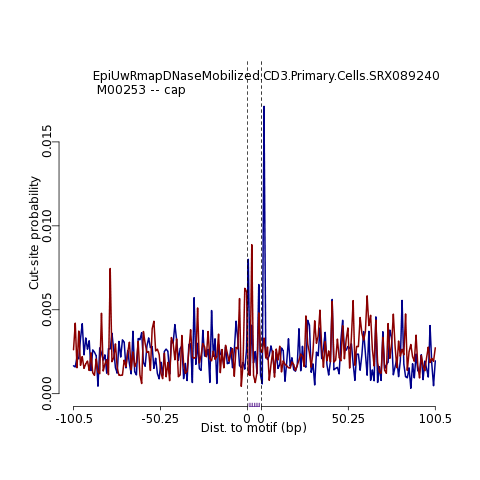

Supplement: S3 File — For each motif, footprint profiles are aggregated across all binding sites in all 653 DNase-seq samples. Color indicates which strand the motif matches, positive (blue) or negative (red). Text in the upper left denotes the tissue with the highest Z-score from the CENTIPEDE mode, the motif ID, and the corresponding transcription factor. (GZ) [file pgen.1005875.s004.tar.gz › recalibratedMotifShape/M00253.lambda.png]

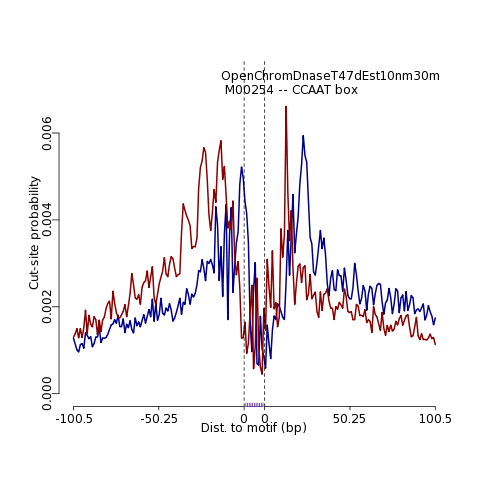

Supplement: S3 File — For each motif, footprint profiles are aggregated across all binding sites in all 653 DNase-seq samples. Color indicates which strand the motif matches, positive (blue) or negative (red). Text in the upper left denotes the tissue with the highest Z-score from the CENTIPEDE mode, the motif ID, and the corresponding transcription factor. (GZ) [file pgen.1005875.s004.tar.gz › recalibratedMotifShape/M00254.lambda.png]

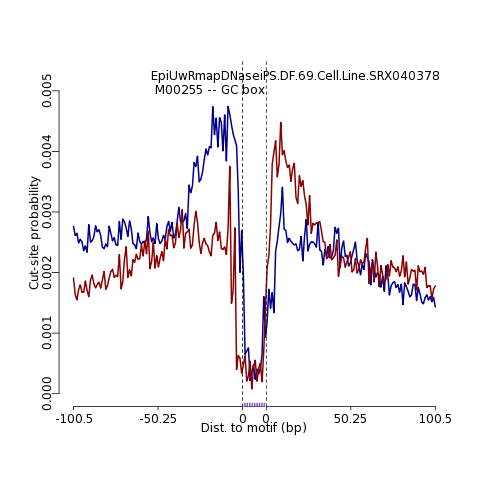

Supplement: S3 File — For each motif, footprint profiles are aggregated across all binding sites in all 653 DNase-seq samples. Color indicates which strand the motif matches, positive (blue) or negative (red). Text in the upper left denotes the tissue with the highest Z-score from the CENTIPEDE mode, the motif ID, and the corresponding transcription factor. (GZ) [file pgen.1005875.s004.tar.gz › recalibratedMotifShape/M00255.lambda.png]

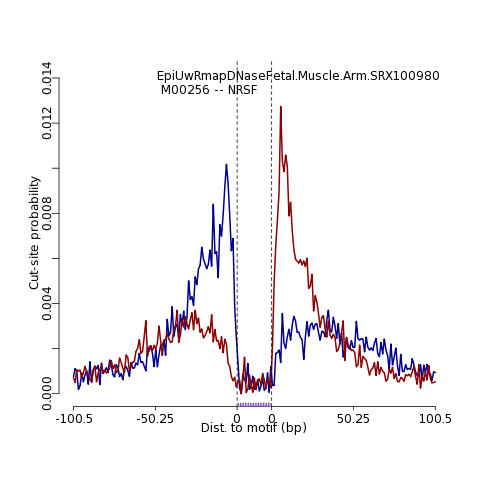

Supplement: S3 File — For each motif, footprint profiles are aggregated across all binding sites in all 653 DNase-seq samples. Color indicates which strand the motif matches, positive (blue) or negative (red). Text in the upper left denotes the tissue with the highest Z-score from the CENTIPEDE mode, the motif ID, and the corresponding transcription factor. (GZ) [file pgen.1005875.s004.tar.gz › recalibratedMotifShape/M00256.lambda.png]

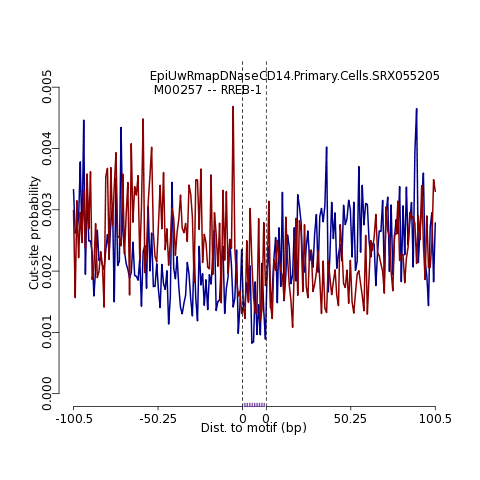

Supplement: S3 File — For each motif, footprint profiles are aggregated across all binding sites in all 653 DNase-seq samples. Color indicates which strand the motif matches, positive (blue) or negative (red). Text in the upper left denotes the tissue with the highest Z-score from the CENTIPEDE mode, the motif ID, and the corresponding transcription factor. (GZ) [file pgen.1005875.s004.tar.gz › recalibratedMotifShape/M00257.lambda.png]

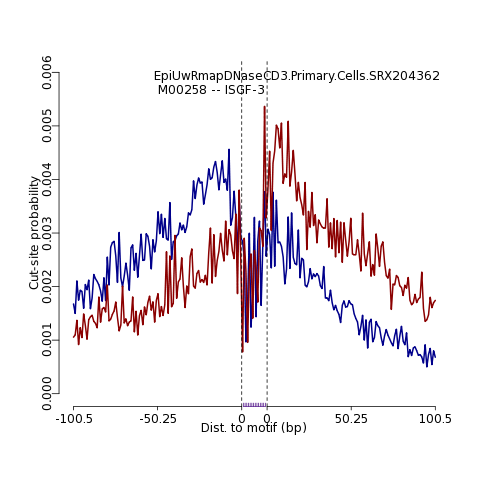

Supplement: S3 File — For each motif, footprint profiles are aggregated across all binding sites in all 653 DNase-seq samples. Color indicates which strand the motif matches, positive (blue) or negative (red). Text in the upper left denotes the tissue with the highest Z-score from the CENTIPEDE mode, the motif ID, and the corresponding transcription factor. (GZ) [file pgen.1005875.s004.tar.gz › recalibratedMotifShape/M00258.lambda.png]

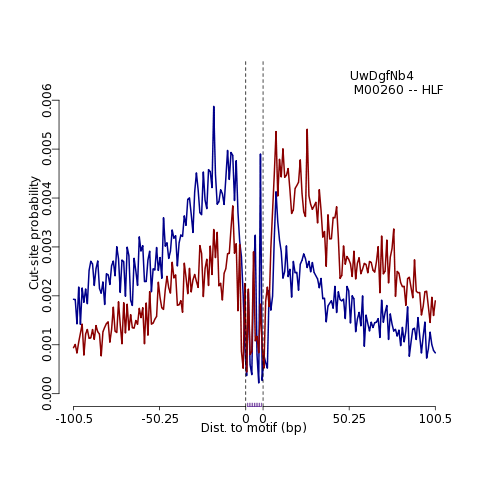

Supplement: S3 File — For each motif, footprint profiles are aggregated across all binding sites in all 653 DNase-seq samples. Color indicates which strand the motif matches, positive (blue) or negative (red). Text in the upper left denotes the tissue with the highest Z-score from the CENTIPEDE mode, the motif ID, and the corresponding transcription factor. (GZ) [file pgen.1005875.s004.tar.gz › recalibratedMotifShape/M00260.lambda.png]

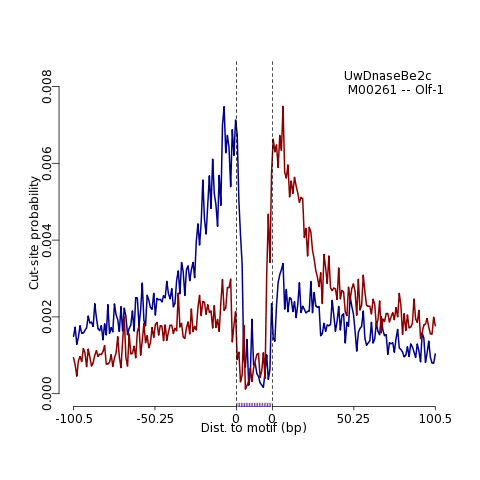

Supplement: S3 File — For each motif, footprint profiles are aggregated across all binding sites in all 653 DNase-seq samples. Color indicates which strand the motif matches, positive (blue) or negative (red). Text in the upper left denotes the tissue with the highest Z-score from the CENTIPEDE mode, the motif ID, and the corresponding transcription factor. (GZ) [file pgen.1005875.s004.tar.gz › recalibratedMotifShape/M00261.lambda.png]

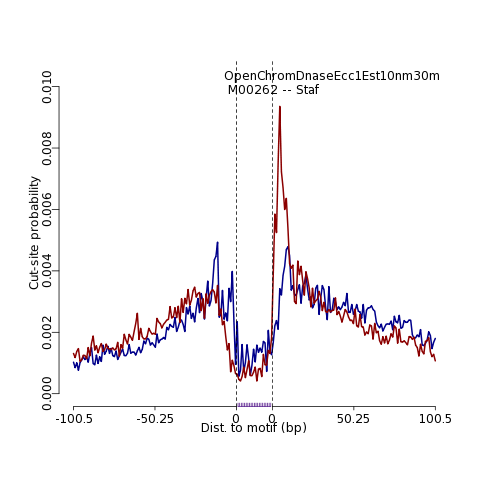

Supplement: S3 File — For each motif, footprint profiles are aggregated across all binding sites in all 653 DNase-seq samples. Color indicates which strand the motif matches, positive (blue) or negative (red). Text in the upper left denotes the tissue with the highest Z-score from the CENTIPEDE mode, the motif ID, and the corresponding transcription factor. (GZ) [file pgen.1005875.s004.tar.gz › recalibratedMotifShape/M00262.lambda.png]

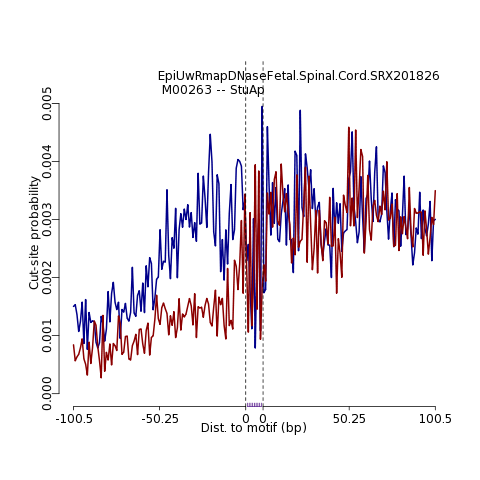

Supplement: S3 File — For each motif, footprint profiles are aggregated across all binding sites in all 653 DNase-seq samples. Color indicates which strand the motif matches, positive (blue) or negative (red). Text in the upper left denotes the tissue with the highest Z-score from the CENTIPEDE mode, the motif ID, and the corresponding transcription factor. (GZ) [file pgen.1005875.s004.tar.gz › recalibratedMotifShape/M00263.lambda.png]

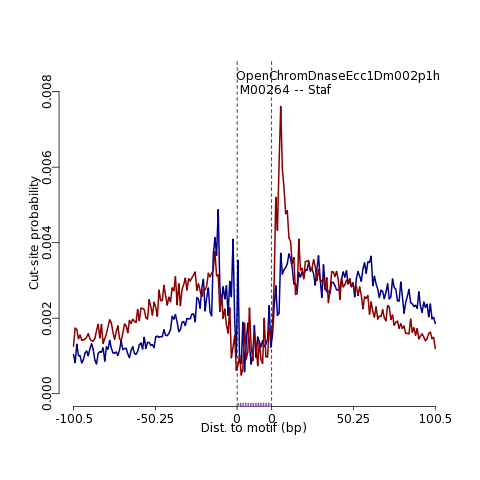

Supplement: S3 File — For each motif, footprint profiles are aggregated across all binding sites in all 653 DNase-seq samples. Color indicates which strand the motif matches, positive (blue) or negative (red). Text in the upper left denotes the tissue with the highest Z-score from the CENTIPEDE mode, the motif ID, and the corresponding transcription factor. (GZ) [file pgen.1005875.s004.tar.gz › recalibratedMotifShape/M00264.lambda.png]

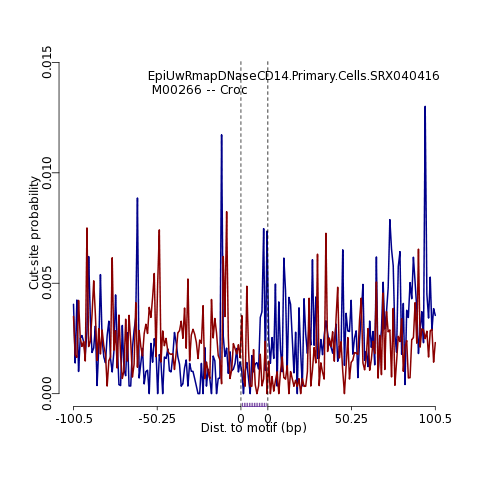

Supplement: S3 File — For each motif, footprint profiles are aggregated across all binding sites in all 653 DNase-seq samples. Color indicates which strand the motif matches, positive (blue) or negative (red). Text in the upper left denotes the tissue with the highest Z-score from the CENTIPEDE mode, the motif ID, and the corresponding transcription factor. (GZ) [file pgen.1005875.s004.tar.gz › recalibratedMotifShape/M00266.lambda.png]

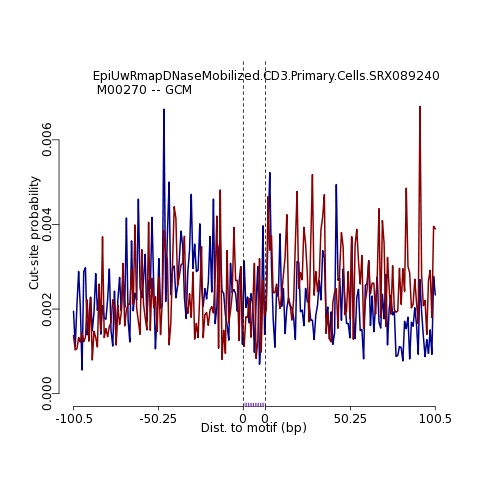

Supplement: S3 File — For each motif, footprint profiles are aggregated across all binding sites in all 653 DNase-seq samples. Color indicates which strand the motif matches, positive (blue) or negative (red). Text in the upper left denotes the tissue with the highest Z-score from the CENTIPEDE mode, the motif ID, and the corresponding transcription factor. (GZ) [file pgen.1005875.s004.tar.gz › recalibratedMotifShape/M00270.lambda.png]

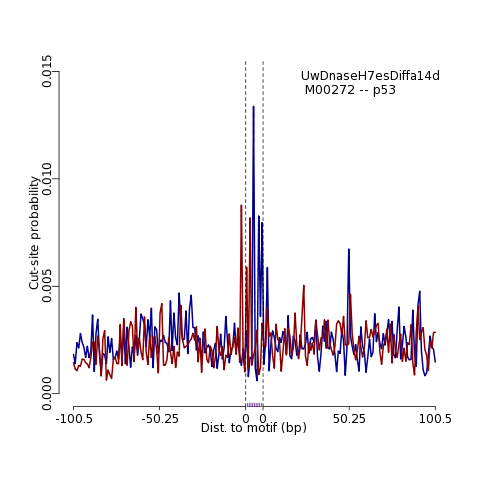

Supplement: S3 File — For each motif, footprint profiles are aggregated across all binding sites in all 653 DNase-seq samples. Color indicates which strand the motif matches, positive (blue) or negative (red). Text in the upper left denotes the tissue with the highest Z-score from the CENTIPEDE mode, the motif ID, and the corresponding transcription factor. (GZ) [file pgen.1005875.s004.tar.gz › recalibratedMotifShape/M00272.lambda.png]

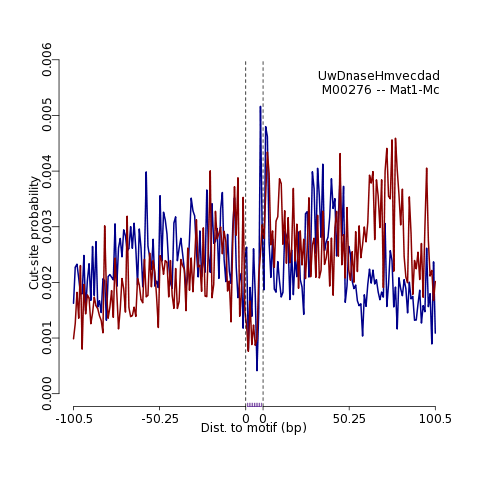

Supplement: S3 File — For each motif, footprint profiles are aggregated across all binding sites in all 653 DNase-seq samples. Color indicates which strand the motif matches, positive (blue) or negative (red). Text in the upper left denotes the tissue with the highest Z-score from the CENTIPEDE mode, the motif ID, and the corresponding transcription factor. (GZ) [file pgen.1005875.s004.tar.gz › recalibratedMotifShape/M00276.lambda.png]

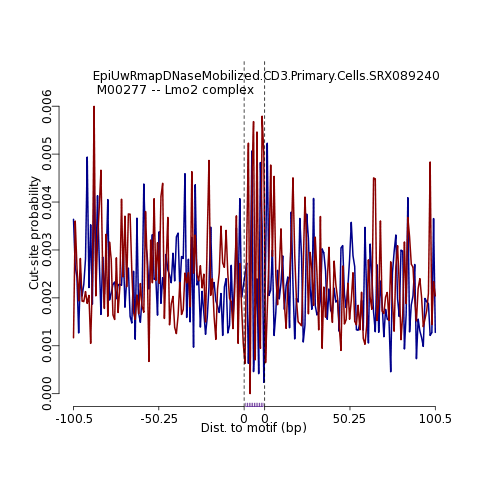

Supplement: S3 File — For each motif, footprint profiles are aggregated across all binding sites in all 653 DNase-seq samples. Color indicates which strand the motif matches, positive (blue) or negative (red). Text in the upper left denotes the tissue with the highest Z-score from the CENTIPEDE mode, the motif ID, and the corresponding transcription factor. (GZ) [file pgen.1005875.s004.tar.gz › recalibratedMotifShape/M00277.lambda.png]
