# Supplementary figures and images for: Which Genetics Variants in DNase-Seq Footprints Are More Likely to Alter Binding? (part 1 of 2)
Source: PLoS Genet. 2016 Feb 22;12(2):e1005875. doi: 10.1371/journal.pgen.1005875 (PMC4764260; doi:10.1371/journal.pgen.1005875)

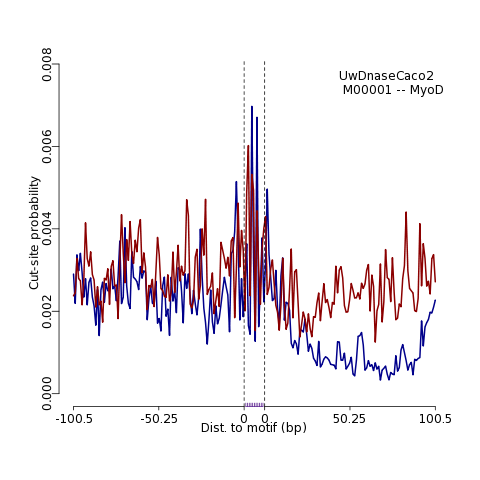

Supplement: S3 File — For each motif, footprint profiles are aggregated across all binding sites in all 653 DNase-seq samples. Color indicates which strand the motif matches, positive (blue) or negative (red). Text in the upper left denotes the tissue with the highest Z-score from the CENTIPEDE mode, the motif ID, and the corresponding transcription factor. (GZ) [file pgen.1005875.s004.tar.gz › recalibratedMotifShape/M00001.lambda.png]

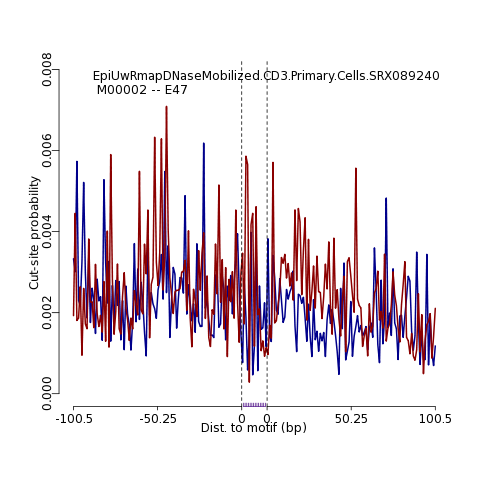

Supplement: S3 File — For each motif, footprint profiles are aggregated across all binding sites in all 653 DNase-seq samples. Color indicates which strand the motif matches, positive (blue) or negative (red). Text in the upper left denotes the tissue with the highest Z-score from the CENTIPEDE mode, the motif ID, and the corresponding transcription factor. (GZ) [file pgen.1005875.s004.tar.gz › recalibratedMotifShape/M00002.lambda.png]

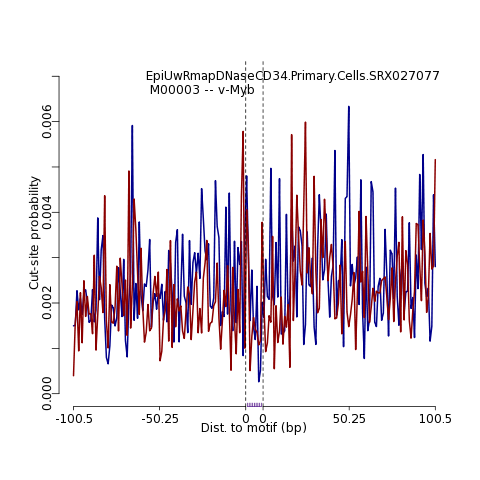

Supplement: S3 File — For each motif, footprint profiles are aggregated across all binding sites in all 653 DNase-seq samples. Color indicates which strand the motif matches, positive (blue) or negative (red). Text in the upper left denotes the tissue with the highest Z-score from the CENTIPEDE mode, the motif ID, and the corresponding transcription factor. (GZ) [file pgen.1005875.s004.tar.gz › recalibratedMotifShape/M00003.lambda.png]

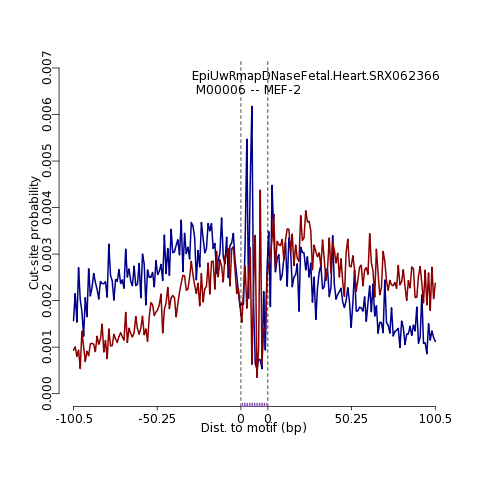

Supplement: S3 File — For each motif, footprint profiles are aggregated across all binding sites in all 653 DNase-seq samples. Color indicates which strand the motif matches, positive (blue) or negative (red). Text in the upper left denotes the tissue with the highest Z-score from the CENTIPEDE mode, the motif ID, and the corresponding transcription factor. (GZ) [file pgen.1005875.s004.tar.gz › recalibratedMotifShape/M00006.lambda.png]

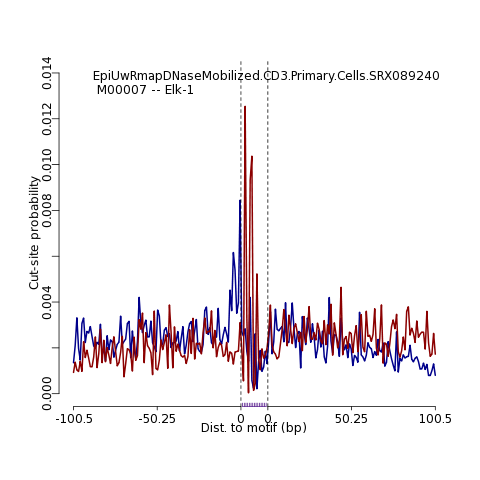

Supplement: S3 File — For each motif, footprint profiles are aggregated across all binding sites in all 653 DNase-seq samples. Color indicates which strand the motif matches, positive (blue) or negative (red). Text in the upper left denotes the tissue with the highest Z-score from the CENTIPEDE mode, the motif ID, and the corresponding transcription factor. (GZ) [file pgen.1005875.s004.tar.gz › recalibratedMotifShape/M00007.lambda.png]

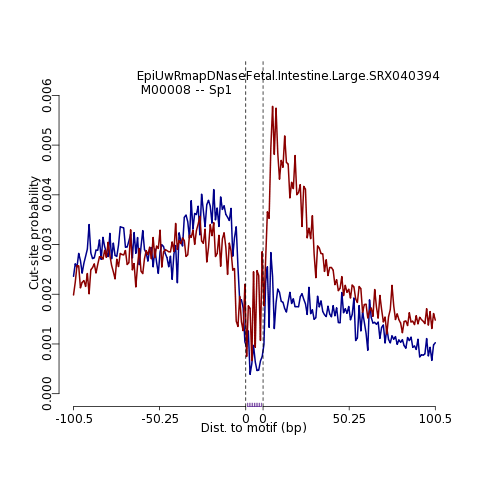

Supplement: S3 File — For each motif, footprint profiles are aggregated across all binding sites in all 653 DNase-seq samples. Color indicates which strand the motif matches, positive (blue) or negative (red). Text in the upper left denotes the tissue with the highest Z-score from the CENTIPEDE mode, the motif ID, and the corresponding transcription factor. (GZ) [file pgen.1005875.s004.tar.gz › recalibratedMotifShape/M00008.lambda.png]

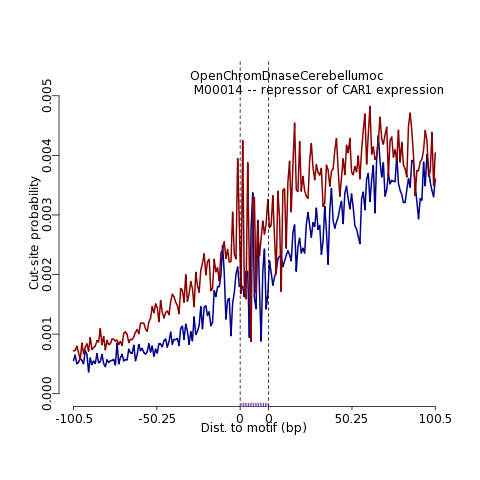

Supplement: S3 File — For each motif, footprint profiles are aggregated across all binding sites in all 653 DNase-seq samples. Color indicates which strand the motif matches, positive (blue) or negative (red). Text in the upper left denotes the tissue with the highest Z-score from the CENTIPEDE mode, the motif ID, and the corresponding transcription factor. (GZ) [file pgen.1005875.s004.tar.gz › recalibratedMotifShape/M00014.lambda.png]

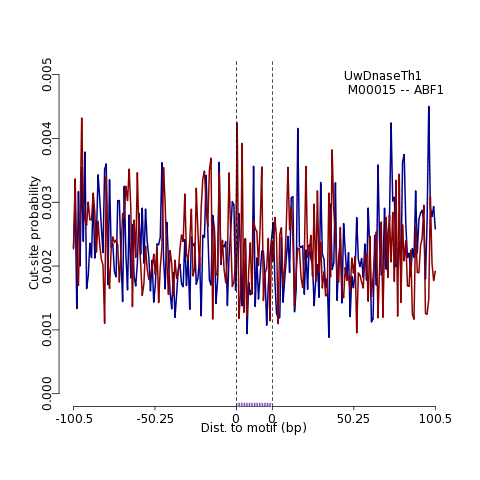

Supplement: S3 File — For each motif, footprint profiles are aggregated across all binding sites in all 653 DNase-seq samples. Color indicates which strand the motif matches, positive (blue) or negative (red). Text in the upper left denotes the tissue with the highest Z-score from the CENTIPEDE mode, the motif ID, and the corresponding transcription factor. (GZ) [file pgen.1005875.s004.tar.gz › recalibratedMotifShape/M00015.lambda.png]

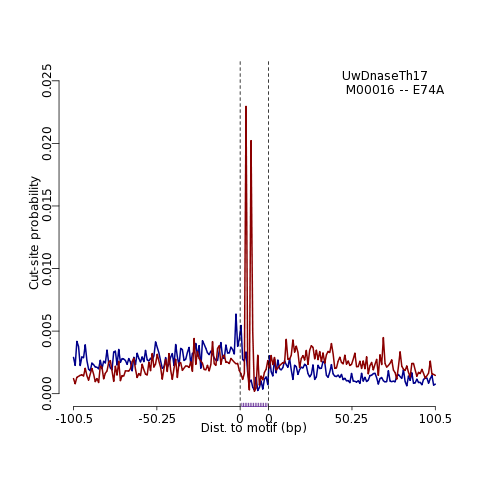

Supplement: S3 File — For each motif, footprint profiles are aggregated across all binding sites in all 653 DNase-seq samples. Color indicates which strand the motif matches, positive (blue) or negative (red). Text in the upper left denotes the tissue with the highest Z-score from the CENTIPEDE mode, the motif ID, and the corresponding transcription factor. (GZ) [file pgen.1005875.s004.tar.gz › recalibratedMotifShape/M00016.lambda.png]

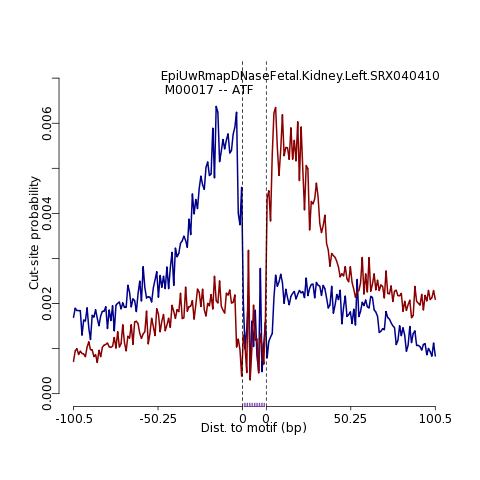

Supplement: S3 File — For each motif, footprint profiles are aggregated across all binding sites in all 653 DNase-seq samples. Color indicates which strand the motif matches, positive (blue) or negative (red). Text in the upper left denotes the tissue with the highest Z-score from the CENTIPEDE mode, the motif ID, and the corresponding transcription factor. (GZ) [file pgen.1005875.s004.tar.gz › recalibratedMotifShape/M00017.lambda.png]

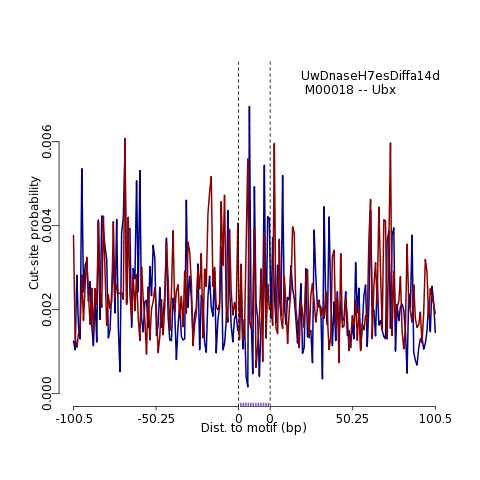

Supplement: S3 File — For each motif, footprint profiles are aggregated across all binding sites in all 653 DNase-seq samples. Color indicates which strand the motif matches, positive (blue) or negative (red). Text in the upper left denotes the tissue with the highest Z-score from the CENTIPEDE mode, the motif ID, and the corresponding transcription factor. (GZ) [file pgen.1005875.s004.tar.gz › recalibratedMotifShape/M00018.lambda.png]

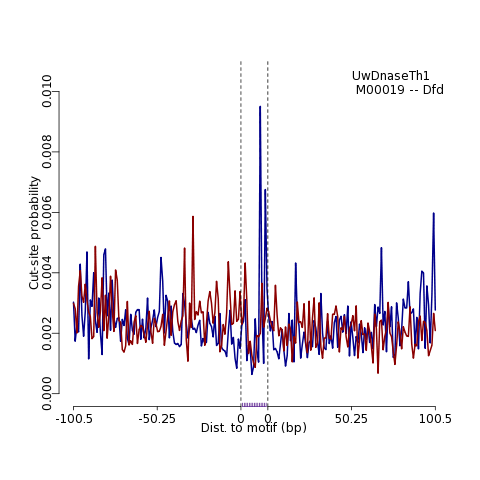

Supplement: S3 File — For each motif, footprint profiles are aggregated across all binding sites in all 653 DNase-seq samples. Color indicates which strand the motif matches, positive (blue) or negative (red). Text in the upper left denotes the tissue with the highest Z-score from the CENTIPEDE mode, the motif ID, and the corresponding transcription factor. (GZ) [file pgen.1005875.s004.tar.gz › recalibratedMotifShape/M00019.lambda.png]

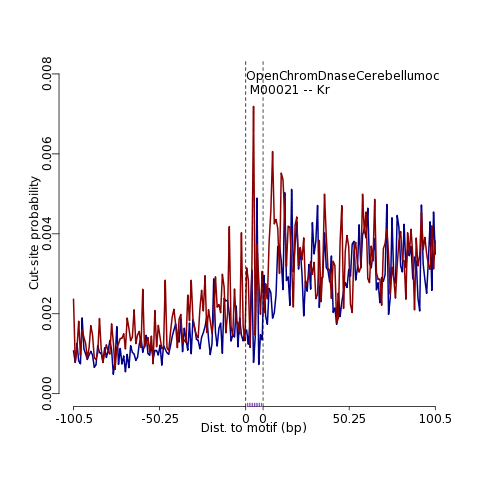

Supplement: S3 File — For each motif, footprint profiles are aggregated across all binding sites in all 653 DNase-seq samples. Color indicates which strand the motif matches, positive (blue) or negative (red). Text in the upper left denotes the tissue with the highest Z-score from the CENTIPEDE mode, the motif ID, and the corresponding transcription factor. (GZ) [file pgen.1005875.s004.tar.gz › recalibratedMotifShape/M00021.lambda.png]

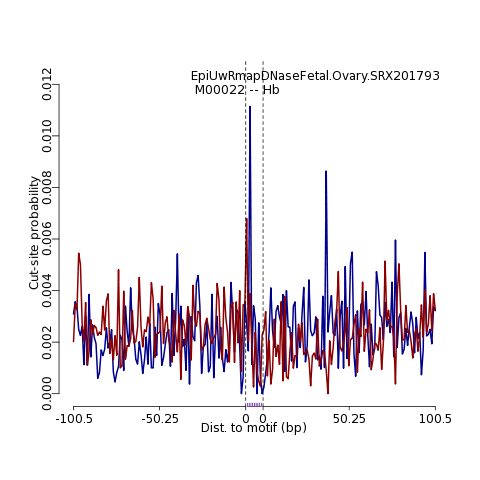

Supplement: S3 File — For each motif, footprint profiles are aggregated across all binding sites in all 653 DNase-seq samples. Color indicates which strand the motif matches, positive (blue) or negative (red). Text in the upper left denotes the tissue with the highest Z-score from the CENTIPEDE mode, the motif ID, and the corresponding transcription factor. (GZ) [file pgen.1005875.s004.tar.gz › recalibratedMotifShape/M00022.lambda.png]

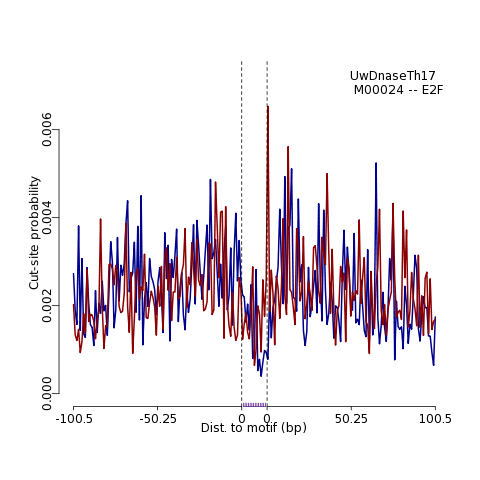

Supplement: S3 File — For each motif, footprint profiles are aggregated across all binding sites in all 653 DNase-seq samples. Color indicates which strand the motif matches, positive (blue) or negative (red). Text in the upper left denotes the tissue with the highest Z-score from the CENTIPEDE mode, the motif ID, and the corresponding transcription factor. (GZ) [file pgen.1005875.s004.tar.gz › recalibratedMotifShape/M00024.lambda.png]

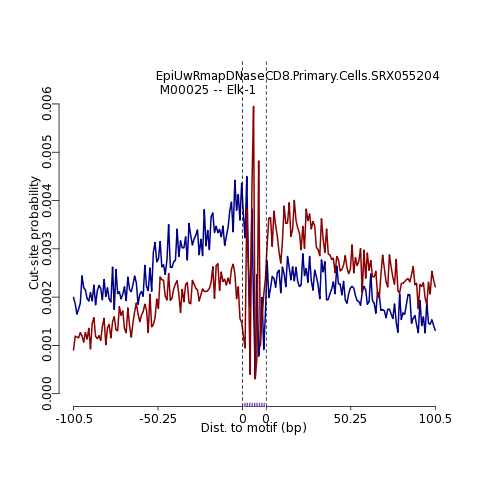

Supplement: S3 File — For each motif, footprint profiles are aggregated across all binding sites in all 653 DNase-seq samples. Color indicates which strand the motif matches, positive (blue) or negative (red). Text in the upper left denotes the tissue with the highest Z-score from the CENTIPEDE mode, the motif ID, and the corresponding transcription factor. (GZ) [file pgen.1005875.s004.tar.gz › recalibratedMotifShape/M00025.lambda.png]

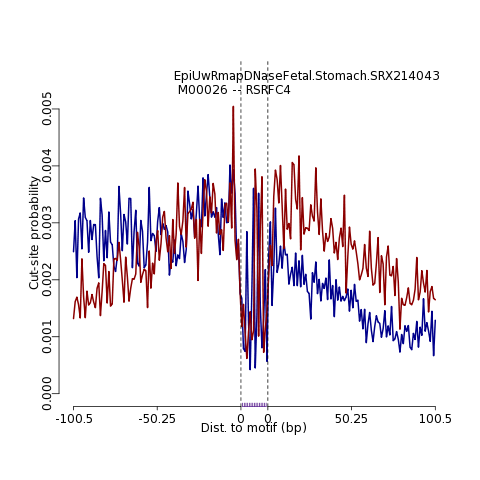

Supplement: S3 File — For each motif, footprint profiles are aggregated across all binding sites in all 653 DNase-seq samples. Color indicates which strand the motif matches, positive (blue) or negative (red). Text in the upper left denotes the tissue with the highest Z-score from the CENTIPEDE mode, the motif ID, and the corresponding transcription factor. (GZ) [file pgen.1005875.s004.tar.gz › recalibratedMotifShape/M00026.lambda.png]

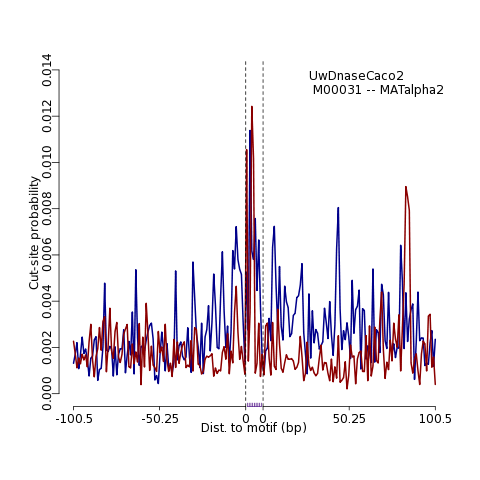

Supplement: S3 File — For each motif, footprint profiles are aggregated across all binding sites in all 653 DNase-seq samples. Color indicates which strand the motif matches, positive (blue) or negative (red). Text in the upper left denotes the tissue with the highest Z-score from the CENTIPEDE mode, the motif ID, and the corresponding transcription factor. (GZ) [file pgen.1005875.s004.tar.gz › recalibratedMotifShape/M00031.lambda.png]

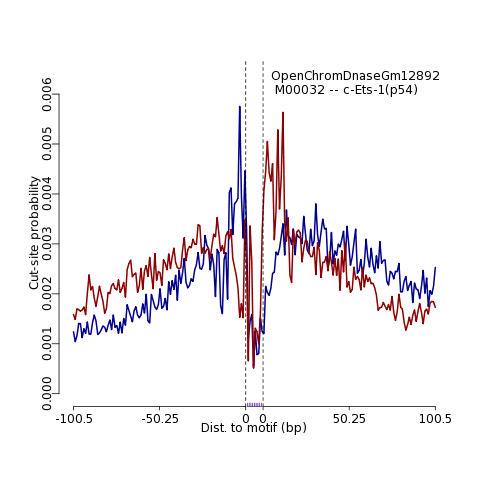

Supplement: S3 File — For each motif, footprint profiles are aggregated across all binding sites in all 653 DNase-seq samples. Color indicates which strand the motif matches, positive (blue) or negative (red). Text in the upper left denotes the tissue with the highest Z-score from the CENTIPEDE mode, the motif ID, and the corresponding transcription factor. (GZ) [file pgen.1005875.s004.tar.gz › recalibratedMotifShape/M00032.lambda.png]

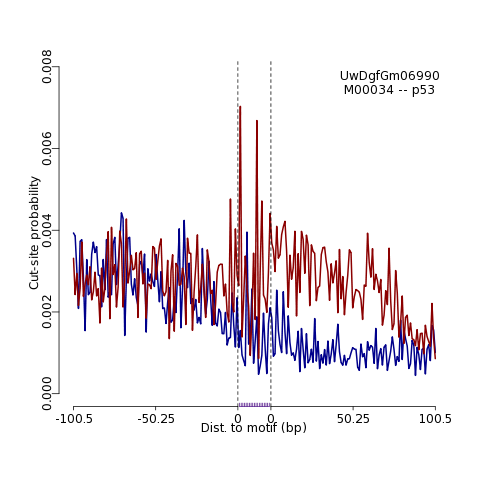

Supplement: S3 File — For each motif, footprint profiles are aggregated across all binding sites in all 653 DNase-seq samples. Color indicates which strand the motif matches, positive (blue) or negative (red). Text in the upper left denotes the tissue with the highest Z-score from the CENTIPEDE mode, the motif ID, and the corresponding transcription factor. (GZ) [file pgen.1005875.s004.tar.gz › recalibratedMotifShape/M00034.lambda.png]

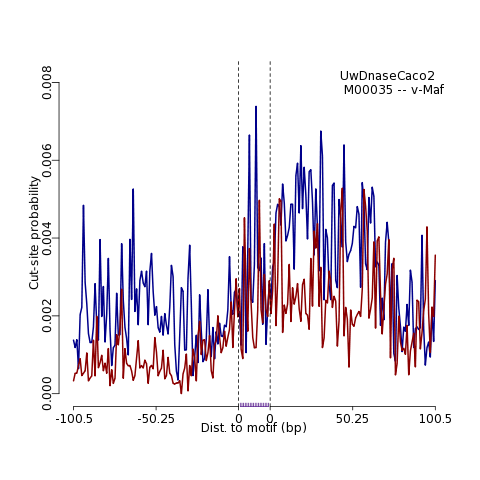

Supplement: S3 File — For each motif, footprint profiles are aggregated across all binding sites in all 653 DNase-seq samples. Color indicates which strand the motif matches, positive (blue) or negative (red). Text in the upper left denotes the tissue with the highest Z-score from the CENTIPEDE mode, the motif ID, and the corresponding transcription factor. (GZ) [file pgen.1005875.s004.tar.gz › recalibratedMotifShape/M00035.lambda.png]

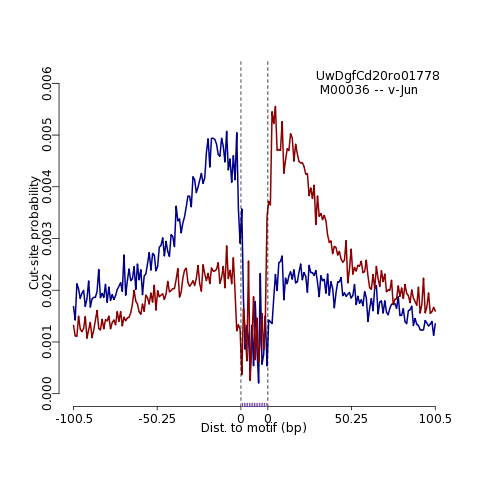

Supplement: S3 File — For each motif, footprint profiles are aggregated across all binding sites in all 653 DNase-seq samples. Color indicates which strand the motif matches, positive (blue) or negative (red). Text in the upper left denotes the tissue with the highest Z-score from the CENTIPEDE mode, the motif ID, and the corresponding transcription factor. (GZ) [file pgen.1005875.s004.tar.gz › recalibratedMotifShape/M00036.lambda.png]

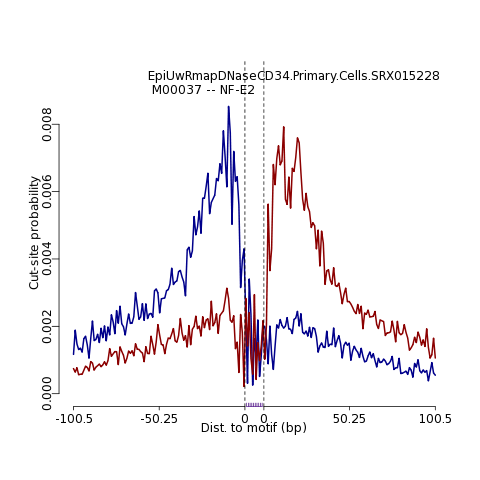

Supplement: S3 File — For each motif, footprint profiles are aggregated across all binding sites in all 653 DNase-seq samples. Color indicates which strand the motif matches, positive (blue) or negative (red). Text in the upper left denotes the tissue with the highest Z-score from the CENTIPEDE mode, the motif ID, and the corresponding transcription factor. (GZ) [file pgen.1005875.s004.tar.gz › recalibratedMotifShape/M00037.lambda.png]

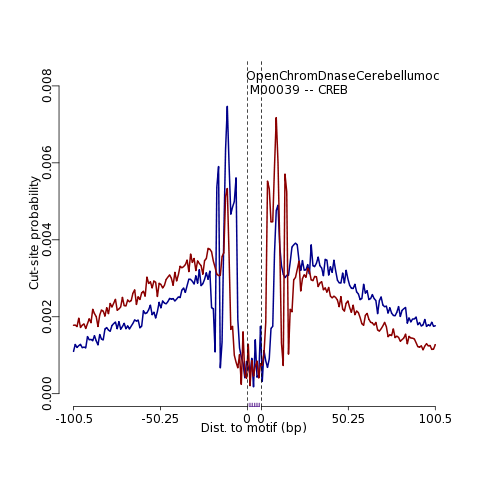

Supplement: S3 File — For each motif, footprint profiles are aggregated across all binding sites in all 653 DNase-seq samples. Color indicates which strand the motif matches, positive (blue) or negative (red). Text in the upper left denotes the tissue with the highest Z-score from the CENTIPEDE mode, the motif ID, and the corresponding transcription factor. (GZ) [file pgen.1005875.s004.tar.gz › recalibratedMotifShape/M00039.lambda.png]

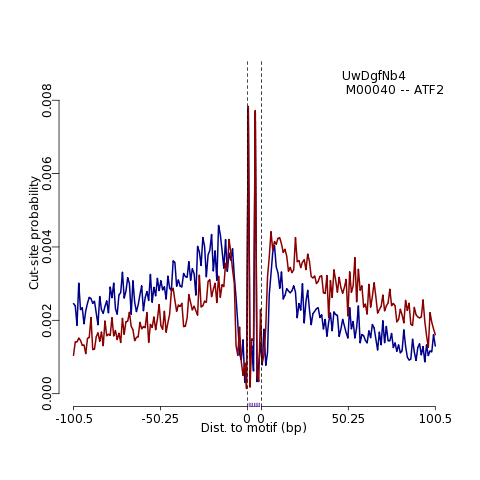

Supplement: S3 File — For each motif, footprint profiles are aggregated across all binding sites in all 653 DNase-seq samples. Color indicates which strand the motif matches, positive (blue) or negative (red). Text in the upper left denotes the tissue with the highest Z-score from the CENTIPEDE mode, the motif ID, and the corresponding transcription factor. (GZ) [file pgen.1005875.s004.tar.gz › recalibratedMotifShape/M00040.lambda.png]

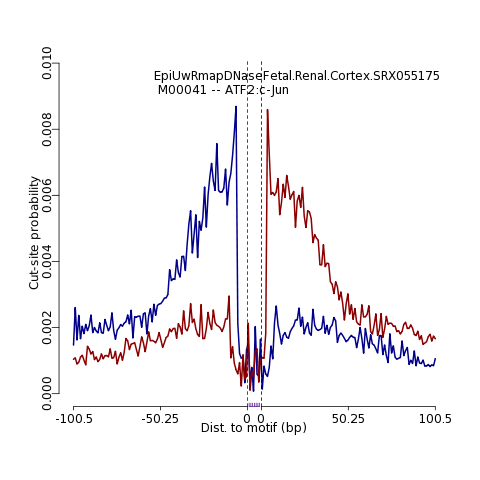

Supplement: S3 File — For each motif, footprint profiles are aggregated across all binding sites in all 653 DNase-seq samples. Color indicates which strand the motif matches, positive (blue) or negative (red). Text in the upper left denotes the tissue with the highest Z-score from the CENTIPEDE mode, the motif ID, and the corresponding transcription factor. (GZ) [file pgen.1005875.s004.tar.gz › recalibratedMotifShape/M00041.lambda.png]

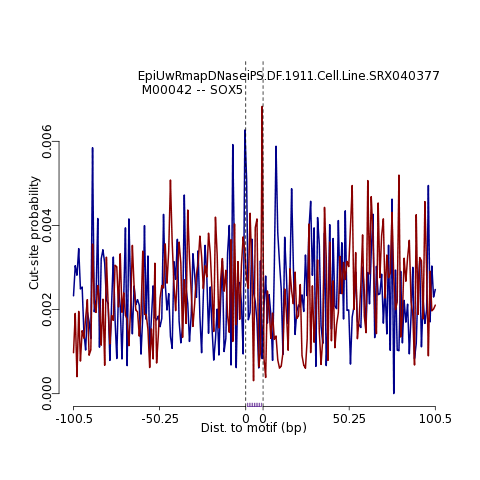

Supplement: S3 File — For each motif, footprint profiles are aggregated across all binding sites in all 653 DNase-seq samples. Color indicates which strand the motif matches, positive (blue) or negative (red). Text in the upper left denotes the tissue with the highest Z-score from the CENTIPEDE mode, the motif ID, and the corresponding transcription factor. (GZ) [file pgen.1005875.s004.tar.gz › recalibratedMotifShape/M00042.lambda.png]

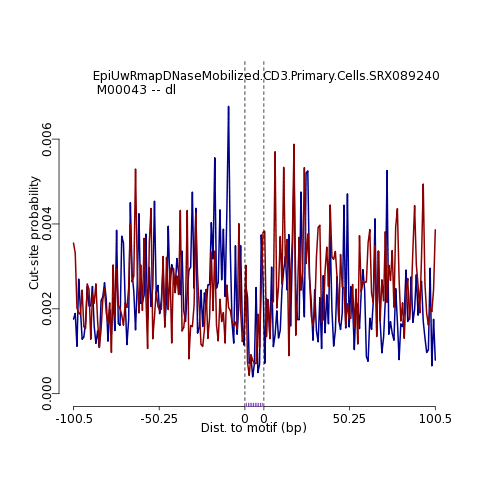

Supplement: S3 File — For each motif, footprint profiles are aggregated across all binding sites in all 653 DNase-seq samples. Color indicates which strand the motif matches, positive (blue) or negative (red). Text in the upper left denotes the tissue with the highest Z-score from the CENTIPEDE mode, the motif ID, and the corresponding transcription factor. (GZ) [file pgen.1005875.s004.tar.gz › recalibratedMotifShape/M00043.lambda.png]

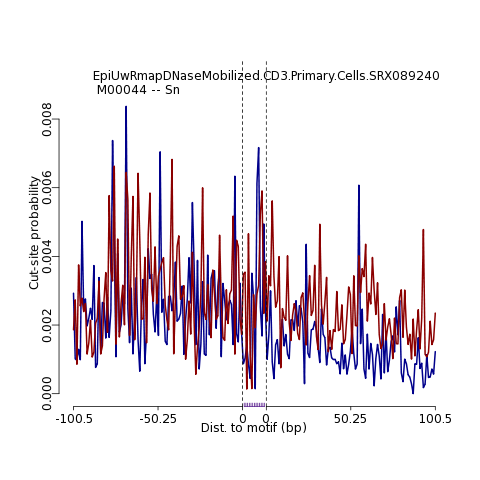

Supplement: S3 File — For each motif, footprint profiles are aggregated across all binding sites in all 653 DNase-seq samples. Color indicates which strand the motif matches, positive (blue) or negative (red). Text in the upper left denotes the tissue with the highest Z-score from the CENTIPEDE mode, the motif ID, and the corresponding transcription factor. (GZ) [file pgen.1005875.s004.tar.gz › recalibratedMotifShape/M00044.lambda.png]

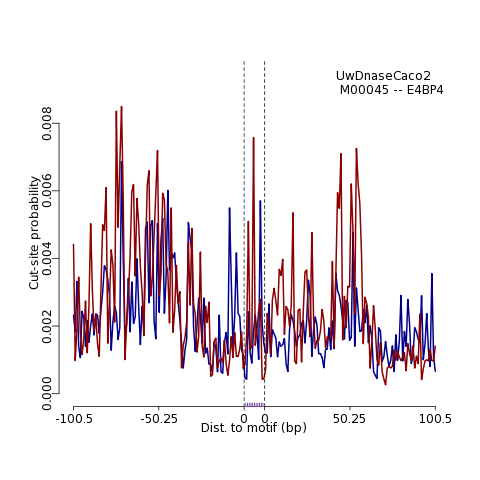

Supplement: S3 File — For each motif, footprint profiles are aggregated across all binding sites in all 653 DNase-seq samples. Color indicates which strand the motif matches, positive (blue) or negative (red). Text in the upper left denotes the tissue with the highest Z-score from the CENTIPEDE mode, the motif ID, and the corresponding transcription factor. (GZ) [file pgen.1005875.s004.tar.gz › recalibratedMotifShape/M00045.lambda.png]

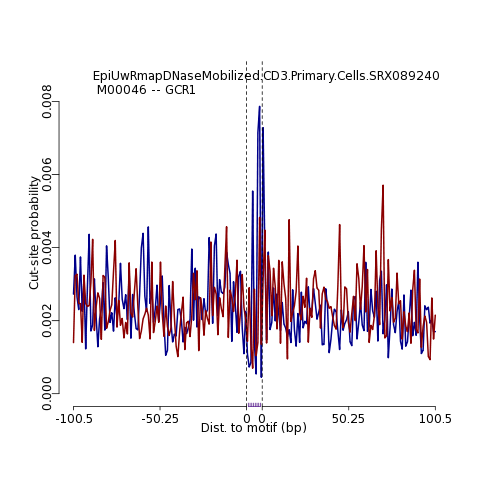

Supplement: S3 File — For each motif, footprint profiles are aggregated across all binding sites in all 653 DNase-seq samples. Color indicates which strand the motif matches, positive (blue) or negative (red). Text in the upper left denotes the tissue with the highest Z-score from the CENTIPEDE mode, the motif ID, and the corresponding transcription factor. (GZ) [file pgen.1005875.s004.tar.gz › recalibratedMotifShape/M00046.lambda.png]

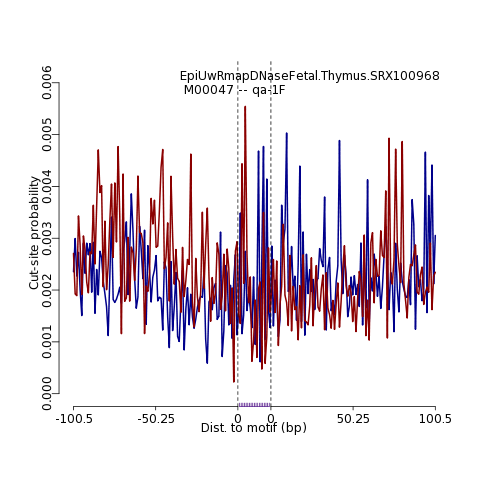

Supplement: S3 File — For each motif, footprint profiles are aggregated across all binding sites in all 653 DNase-seq samples. Color indicates which strand the motif matches, positive (blue) or negative (red). Text in the upper left denotes the tissue with the highest Z-score from the CENTIPEDE mode, the motif ID, and the corresponding transcription factor. (GZ) [file pgen.1005875.s004.tar.gz › recalibratedMotifShape/M00047.lambda.png]

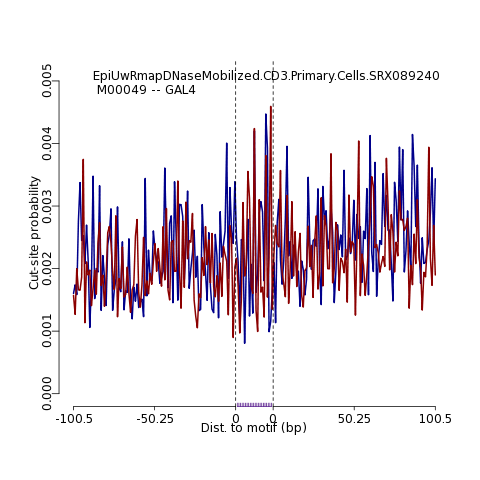

Supplement: S3 File — For each motif, footprint profiles are aggregated across all binding sites in all 653 DNase-seq samples. Color indicates which strand the motif matches, positive (blue) or negative (red). Text in the upper left denotes the tissue with the highest Z-score from the CENTIPEDE mode, the motif ID, and the corresponding transcription factor. (GZ) [file pgen.1005875.s004.tar.gz › recalibratedMotifShape/M00049.lambda.png]

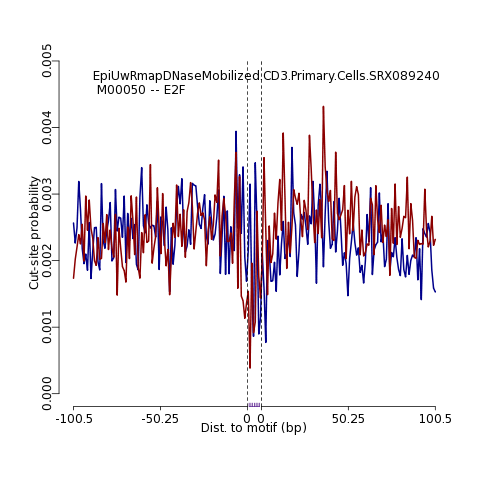

Supplement: S3 File — For each motif, footprint profiles are aggregated across all binding sites in all 653 DNase-seq samples. Color indicates which strand the motif matches, positive (blue) or negative (red). Text in the upper left denotes the tissue with the highest Z-score from the CENTIPEDE mode, the motif ID, and the corresponding transcription factor. (GZ) [file pgen.1005875.s004.tar.gz › recalibratedMotifShape/M00050.lambda.png]

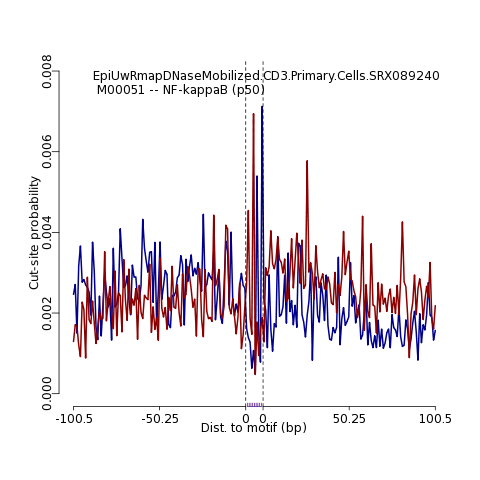

Supplement: S3 File — For each motif, footprint profiles are aggregated across all binding sites in all 653 DNase-seq samples. Color indicates which strand the motif matches, positive (blue) or negative (red). Text in the upper left denotes the tissue with the highest Z-score from the CENTIPEDE mode, the motif ID, and the corresponding transcription factor. (GZ) [file pgen.1005875.s004.tar.gz › recalibratedMotifShape/M00051.lambda.png]

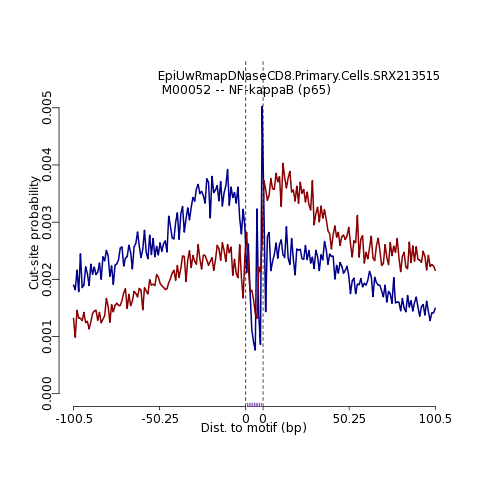

Supplement: S3 File — For each motif, footprint profiles are aggregated across all binding sites in all 653 DNase-seq samples. Color indicates which strand the motif matches, positive (blue) or negative (red). Text in the upper left denotes the tissue with the highest Z-score from the CENTIPEDE mode, the motif ID, and the corresponding transcription factor. (GZ) [file pgen.1005875.s004.tar.gz › recalibratedMotifShape/M00052.lambda.png]

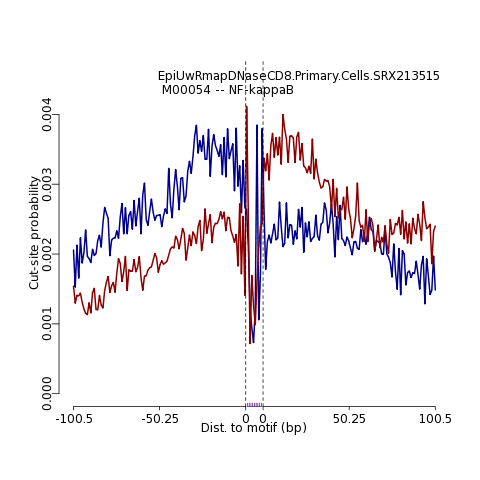

Supplement: S3 File — For each motif, footprint profiles are aggregated across all binding sites in all 653 DNase-seq samples. Color indicates which strand the motif matches, positive (blue) or negative (red). Text in the upper left denotes the tissue with the highest Z-score from the CENTIPEDE mode, the motif ID, and the corresponding transcription factor. (GZ) [file pgen.1005875.s004.tar.gz › recalibratedMotifShape/M00054.lambda.png]

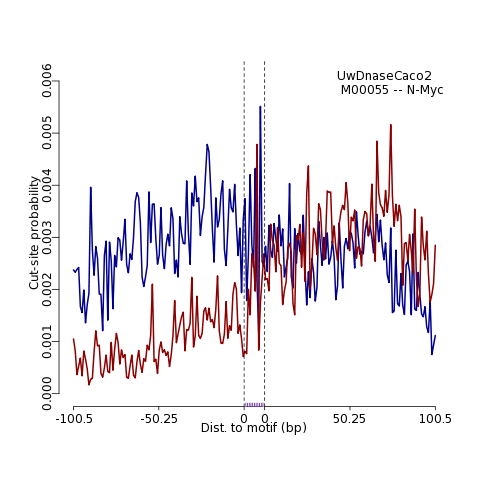

Supplement: S3 File — For each motif, footprint profiles are aggregated across all binding sites in all 653 DNase-seq samples. Color indicates which strand the motif matches, positive (blue) or negative (red). Text in the upper left denotes the tissue with the highest Z-score from the CENTIPEDE mode, the motif ID, and the corresponding transcription factor. (GZ) [file pgen.1005875.s004.tar.gz › recalibratedMotifShape/M00055.lambda.png]

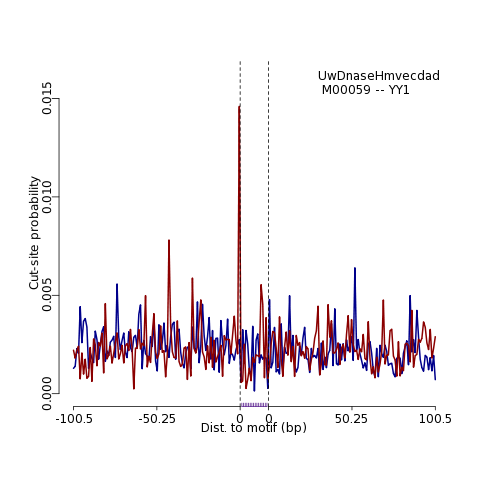

Supplement: S3 File — For each motif, footprint profiles are aggregated across all binding sites in all 653 DNase-seq samples. Color indicates which strand the motif matches, positive (blue) or negative (red). Text in the upper left denotes the tissue with the highest Z-score from the CENTIPEDE mode, the motif ID, and the corresponding transcription factor. (GZ) [file pgen.1005875.s004.tar.gz › recalibratedMotifShape/M00059.lambda.png]

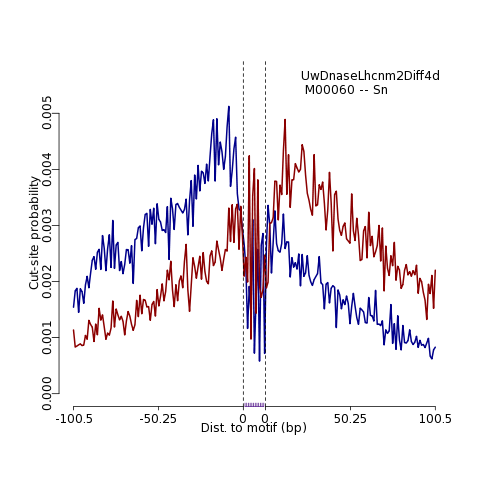

Supplement: S3 File — For each motif, footprint profiles are aggregated across all binding sites in all 653 DNase-seq samples. Color indicates which strand the motif matches, positive (blue) or negative (red). Text in the upper left denotes the tissue with the highest Z-score from the CENTIPEDE mode, the motif ID, and the corresponding transcription factor. (GZ) [file pgen.1005875.s004.tar.gz › recalibratedMotifShape/M00060.lambda.png]

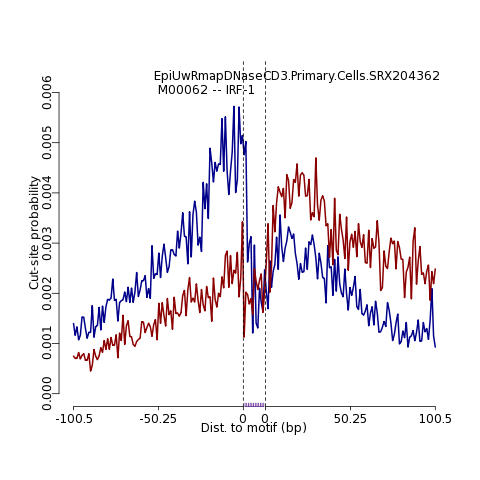

Supplement: S3 File — For each motif, footprint profiles are aggregated across all binding sites in all 653 DNase-seq samples. Color indicates which strand the motif matches, positive (blue) or negative (red). Text in the upper left denotes the tissue with the highest Z-score from the CENTIPEDE mode, the motif ID, and the corresponding transcription factor. (GZ) [file pgen.1005875.s004.tar.gz › recalibratedMotifShape/M00062.lambda.png]

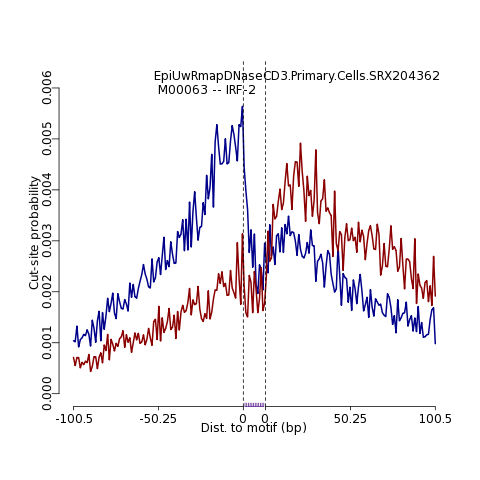

Supplement: S3 File — For each motif, footprint profiles are aggregated across all binding sites in all 653 DNase-seq samples. Color indicates which strand the motif matches, positive (blue) or negative (red). Text in the upper left denotes the tissue with the highest Z-score from the CENTIPEDE mode, the motif ID, and the corresponding transcription factor. (GZ) [file pgen.1005875.s004.tar.gz › recalibratedMotifShape/M00063.lambda.png]

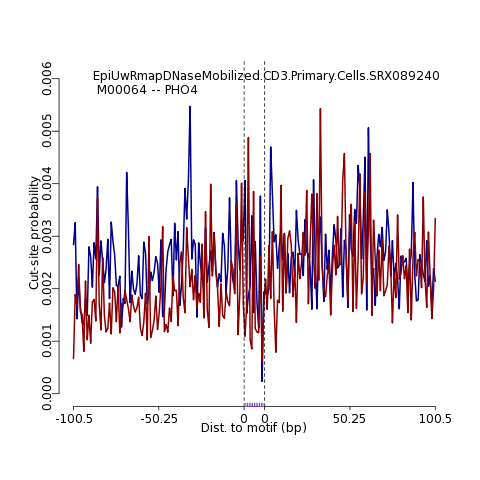

Supplement: S3 File — For each motif, footprint profiles are aggregated across all binding sites in all 653 DNase-seq samples. Color indicates which strand the motif matches, positive (blue) or negative (red). Text in the upper left denotes the tissue with the highest Z-score from the CENTIPEDE mode, the motif ID, and the corresponding transcription factor. (GZ) [file pgen.1005875.s004.tar.gz › recalibratedMotifShape/M00064.lambda.png]

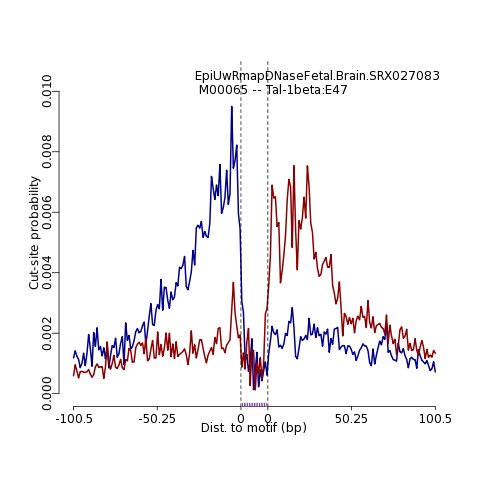

Supplement: S3 File — For each motif, footprint profiles are aggregated across all binding sites in all 653 DNase-seq samples. Color indicates which strand the motif matches, positive (blue) or negative (red). Text in the upper left denotes the tissue with the highest Z-score from the CENTIPEDE mode, the motif ID, and the corresponding transcription factor. (GZ) [file pgen.1005875.s004.tar.gz › recalibratedMotifShape/M00065.lambda.png]

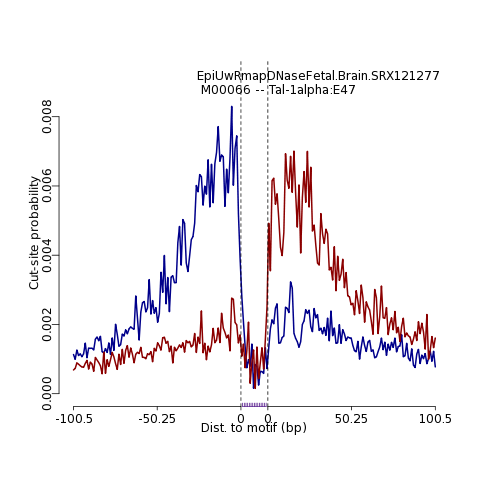

Supplement: S3 File — For each motif, footprint profiles are aggregated across all binding sites in all 653 DNase-seq samples. Color indicates which strand the motif matches, positive (blue) or negative (red). Text in the upper left denotes the tissue with the highest Z-score from the CENTIPEDE mode, the motif ID, and the corresponding transcription factor. (GZ) [file pgen.1005875.s004.tar.gz › recalibratedMotifShape/M00066.lambda.png]

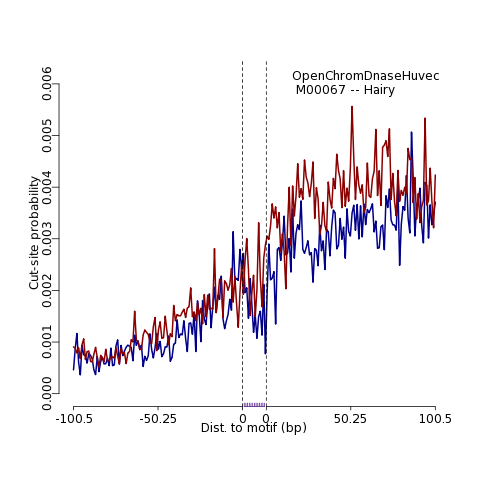

Supplement: S3 File — For each motif, footprint profiles are aggregated across all binding sites in all 653 DNase-seq samples. Color indicates which strand the motif matches, positive (blue) or negative (red). Text in the upper left denotes the tissue with the highest Z-score from the CENTIPEDE mode, the motif ID, and the corresponding transcription factor. (GZ) [file pgen.1005875.s004.tar.gz › recalibratedMotifShape/M00067.lambda.png]

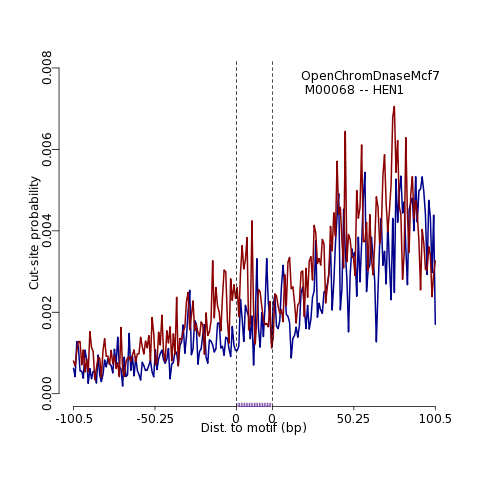

Supplement: S3 File — For each motif, footprint profiles are aggregated across all binding sites in all 653 DNase-seq samples. Color indicates which strand the motif matches, positive (blue) or negative (red). Text in the upper left denotes the tissue with the highest Z-score from the CENTIPEDE mode, the motif ID, and the corresponding transcription factor. (GZ) [file pgen.1005875.s004.tar.gz › recalibratedMotifShape/M00068.lambda.png]

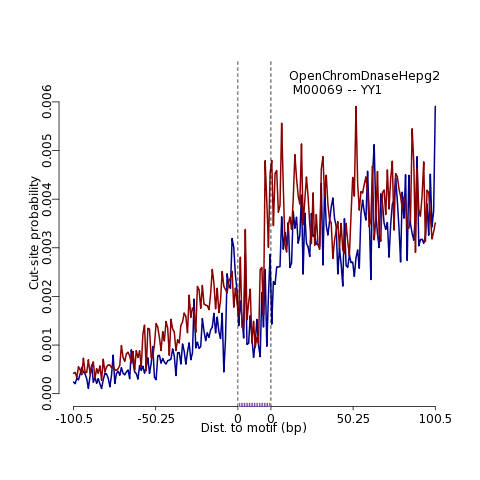

Supplement: S3 File — For each motif, footprint profiles are aggregated across all binding sites in all 653 DNase-seq samples. Color indicates which strand the motif matches, positive (blue) or negative (red). Text in the upper left denotes the tissue with the highest Z-score from the CENTIPEDE mode, the motif ID, and the corresponding transcription factor. (GZ) [file pgen.1005875.s004.tar.gz › recalibratedMotifShape/M00069.lambda.png]

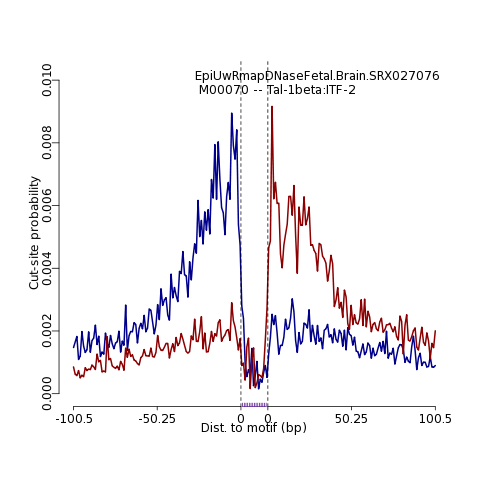

Supplement: S3 File — For each motif, footprint profiles are aggregated across all binding sites in all 653 DNase-seq samples. Color indicates which strand the motif matches, positive (blue) or negative (red). Text in the upper left denotes the tissue with the highest Z-score from the CENTIPEDE mode, the motif ID, and the corresponding transcription factor. (GZ) [file pgen.1005875.s004.tar.gz › recalibratedMotifShape/M00070.lambda.png]

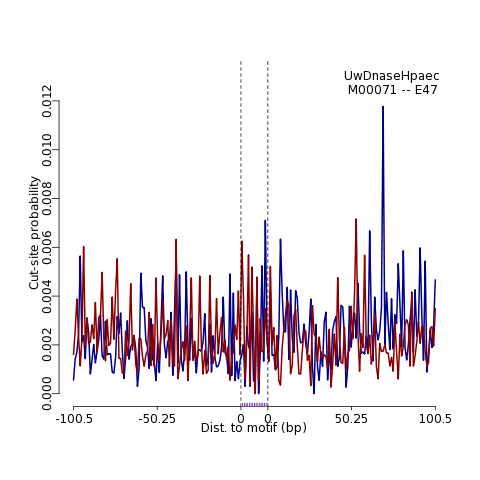

Supplement: S3 File — For each motif, footprint profiles are aggregated across all binding sites in all 653 DNase-seq samples. Color indicates which strand the motif matches, positive (blue) or negative (red). Text in the upper left denotes the tissue with the highest Z-score from the CENTIPEDE mode, the motif ID, and the corresponding transcription factor. (GZ) [file pgen.1005875.s004.tar.gz › recalibratedMotifShape/M00071.lambda.png]

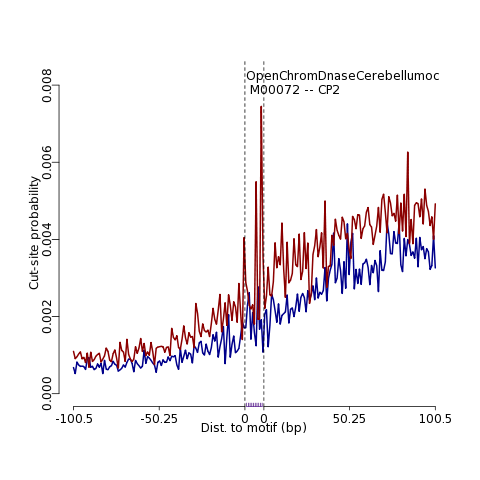

Supplement: S3 File — For each motif, footprint profiles are aggregated across all binding sites in all 653 DNase-seq samples. Color indicates which strand the motif matches, positive (blue) or negative (red). Text in the upper left denotes the tissue with the highest Z-score from the CENTIPEDE mode, the motif ID, and the corresponding transcription factor. (GZ) [file pgen.1005875.s004.tar.gz › recalibratedMotifShape/M00072.lambda.png]

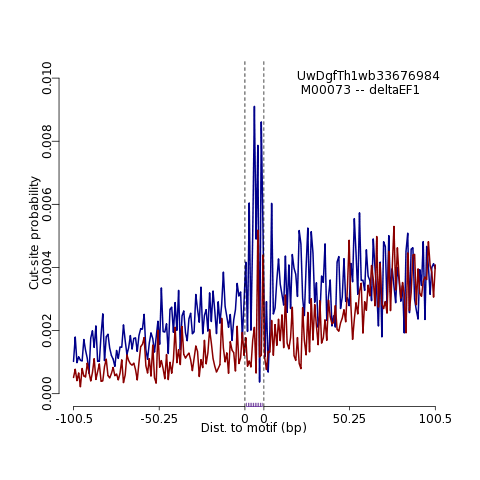

Supplement: S3 File — For each motif, footprint profiles are aggregated across all binding sites in all 653 DNase-seq samples. Color indicates which strand the motif matches, positive (blue) or negative (red). Text in the upper left denotes the tissue with the highest Z-score from the CENTIPEDE mode, the motif ID, and the corresponding transcription factor. (GZ) [file pgen.1005875.s004.tar.gz › recalibratedMotifShape/M00073.lambda.png]

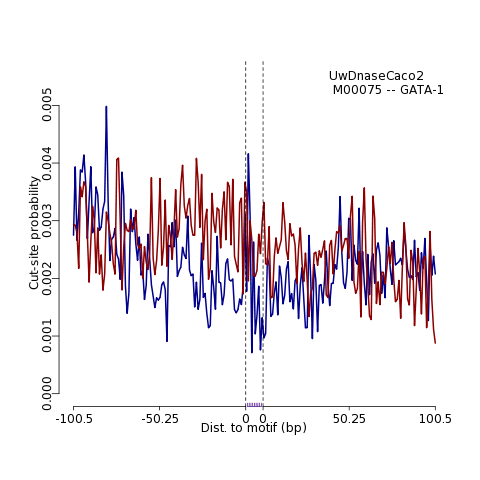

Supplement: S3 File — For each motif, footprint profiles are aggregated across all binding sites in all 653 DNase-seq samples. Color indicates which strand the motif matches, positive (blue) or negative (red). Text in the upper left denotes the tissue with the highest Z-score from the CENTIPEDE mode, the motif ID, and the corresponding transcription factor. (GZ) [file pgen.1005875.s004.tar.gz › recalibratedMotifShape/M00075.lambda.png]

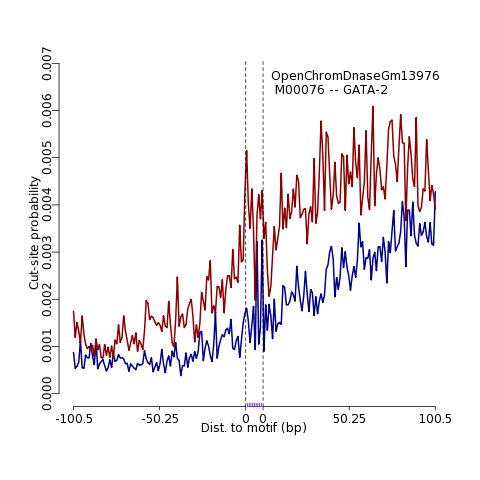

Supplement: S3 File — For each motif, footprint profiles are aggregated across all binding sites in all 653 DNase-seq samples. Color indicates which strand the motif matches, positive (blue) or negative (red). Text in the upper left denotes the tissue with the highest Z-score from the CENTIPEDE mode, the motif ID, and the corresponding transcription factor. (GZ) [file pgen.1005875.s004.tar.gz › recalibratedMotifShape/M00076.lambda.png]

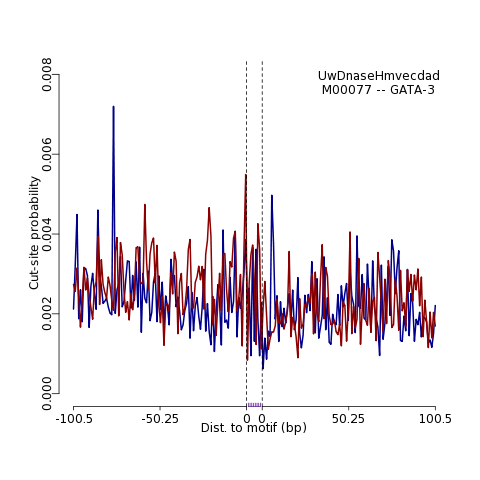

Supplement: S3 File — For each motif, footprint profiles are aggregated across all binding sites in all 653 DNase-seq samples. Color indicates which strand the motif matches, positive (blue) or negative (red). Text in the upper left denotes the tissue with the highest Z-score from the CENTIPEDE mode, the motif ID, and the corresponding transcription factor. (GZ) [file pgen.1005875.s004.tar.gz › recalibratedMotifShape/M00077.lambda.png]

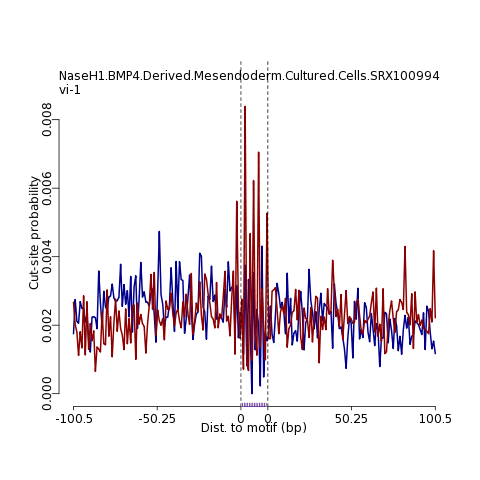

Supplement: S3 File — For each motif, footprint profiles are aggregated across all binding sites in all 653 DNase-seq samples. Color indicates which strand the motif matches, positive (blue) or negative (red). Text in the upper left denotes the tissue with the highest Z-score from the CENTIPEDE mode, the motif ID, and the corresponding transcription factor. (GZ) [file pgen.1005875.s004.tar.gz › recalibratedMotifShape/M00078.lambda.png]

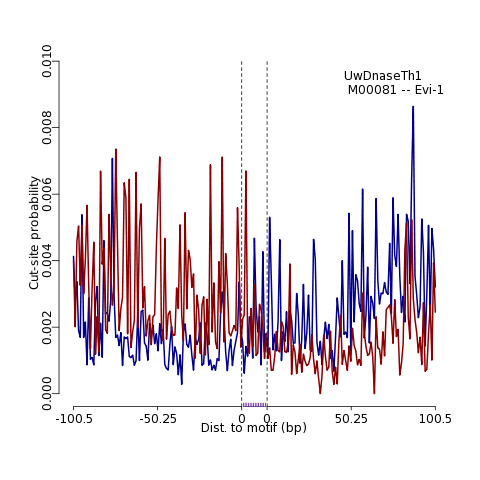

Supplement: S3 File — For each motif, footprint profiles are aggregated across all binding sites in all 653 DNase-seq samples. Color indicates which strand the motif matches, positive (blue) or negative (red). Text in the upper left denotes the tissue with the highest Z-score from the CENTIPEDE mode, the motif ID, and the corresponding transcription factor. (GZ) [file pgen.1005875.s004.tar.gz › recalibratedMotifShape/M00081.lambda.png]

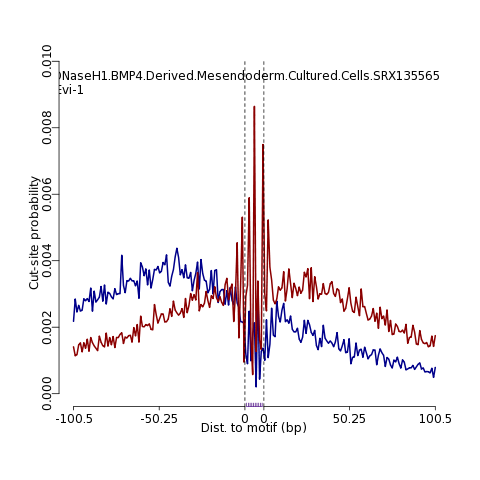

Supplement: S3 File — For each motif, footprint profiles are aggregated across all binding sites in all 653 DNase-seq samples. Color indicates which strand the motif matches, positive (blue) or negative (red). Text in the upper left denotes the tissue with the highest Z-score from the CENTIPEDE mode, the motif ID, and the corresponding transcription factor. (GZ) [file pgen.1005875.s004.tar.gz › recalibratedMotifShape/M00082.lambda.png]

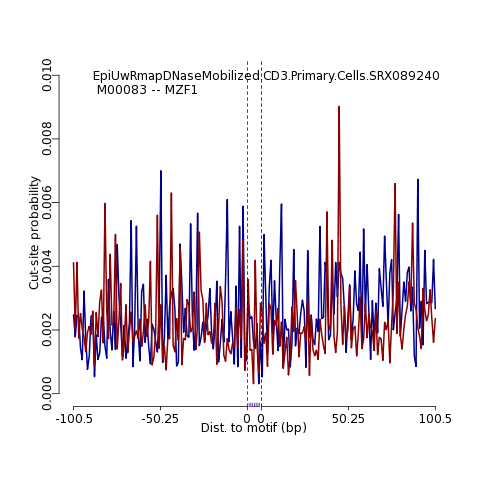

Supplement: S3 File — For each motif, footprint profiles are aggregated across all binding sites in all 653 DNase-seq samples. Color indicates which strand the motif matches, positive (blue) or negative (red). Text in the upper left denotes the tissue with the highest Z-score from the CENTIPEDE mode, the motif ID, and the corresponding transcription factor. (GZ) [file pgen.1005875.s004.tar.gz › recalibratedMotifShape/M00083.lambda.png]

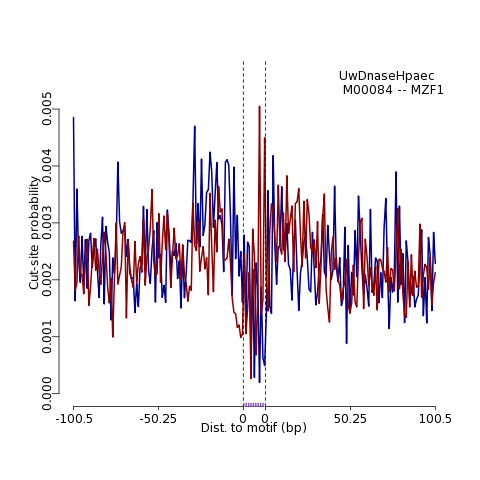

Supplement: S3 File — For each motif, footprint profiles are aggregated across all binding sites in all 653 DNase-seq samples. Color indicates which strand the motif matches, positive (blue) or negative (red). Text in the upper left denotes the tissue with the highest Z-score from the CENTIPEDE mode, the motif ID, and the corresponding transcription factor. (GZ) [file pgen.1005875.s004.tar.gz › recalibratedMotifShape/M00084.lambda.png]

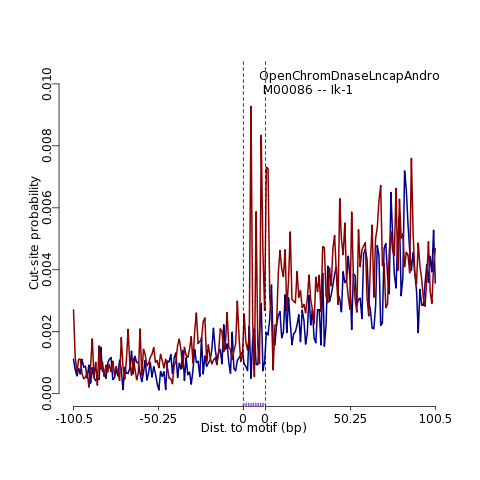

Supplement: S3 File — For each motif, footprint profiles are aggregated across all binding sites in all 653 DNase-seq samples. Color indicates which strand the motif matches, positive (blue) or negative (red). Text in the upper left denotes the tissue with the highest Z-score from the CENTIPEDE mode, the motif ID, and the corresponding transcription factor. (GZ) [file pgen.1005875.s004.tar.gz › recalibratedMotifShape/M00086.lambda.png]

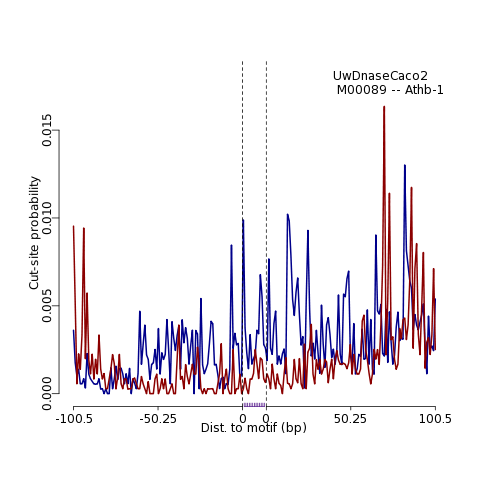

Supplement: S3 File — For each motif, footprint profiles are aggregated across all binding sites in all 653 DNase-seq samples. Color indicates which strand the motif matches, positive (blue) or negative (red). Text in the upper left denotes the tissue with the highest Z-score from the CENTIPEDE mode, the motif ID, and the corresponding transcription factor. (GZ) [file pgen.1005875.s004.tar.gz › recalibratedMotifShape/M00089.lambda.png]

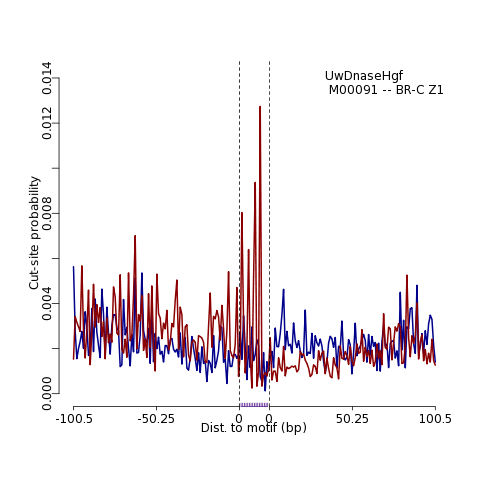

Supplement: S3 File — For each motif, footprint profiles are aggregated across all binding sites in all 653 DNase-seq samples. Color indicates which strand the motif matches, positive (blue) or negative (red). Text in the upper left denotes the tissue with the highest Z-score from the CENTIPEDE mode, the motif ID, and the corresponding transcription factor. (GZ) [file pgen.1005875.s004.tar.gz › recalibratedMotifShape/M00091.lambda.png]

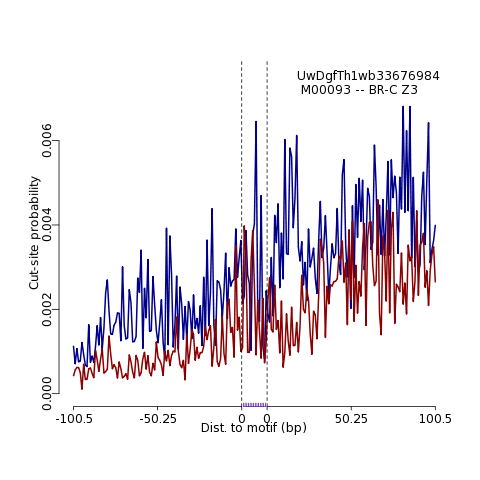

Supplement: S3 File — For each motif, footprint profiles are aggregated across all binding sites in all 653 DNase-seq samples. Color indicates which strand the motif matches, positive (blue) or negative (red). Text in the upper left denotes the tissue with the highest Z-score from the CENTIPEDE mode, the motif ID, and the corresponding transcription factor. (GZ) [file pgen.1005875.s004.tar.gz › recalibratedMotifShape/M00093.lambda.png]

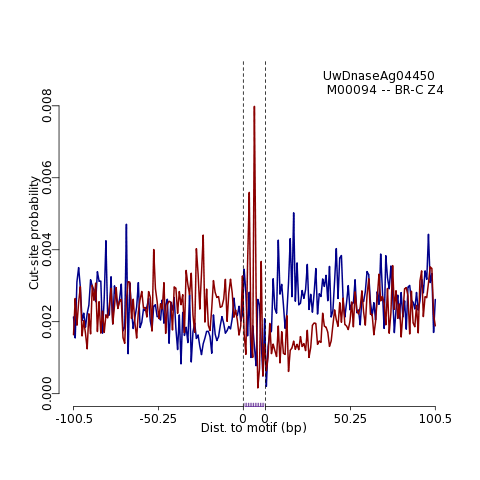

Supplement: S3 File — For each motif, footprint profiles are aggregated across all binding sites in all 653 DNase-seq samples. Color indicates which strand the motif matches, positive (blue) or negative (red). Text in the upper left denotes the tissue with the highest Z-score from the CENTIPEDE mode, the motif ID, and the corresponding transcription factor. (GZ) [file pgen.1005875.s004.tar.gz › recalibratedMotifShape/M00094.lambda.png]

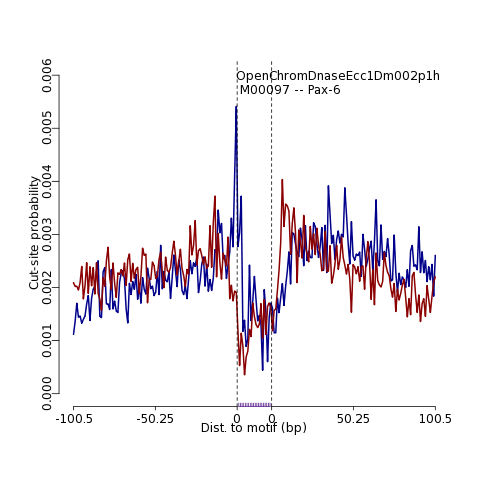

Supplement: S3 File — For each motif, footprint profiles are aggregated across all binding sites in all 653 DNase-seq samples. Color indicates which strand the motif matches, positive (blue) or negative (red). Text in the upper left denotes the tissue with the highest Z-score from the CENTIPEDE mode, the motif ID, and the corresponding transcription factor. (GZ) [file pgen.1005875.s004.tar.gz › recalibratedMotifShape/M00097.lambda.png]

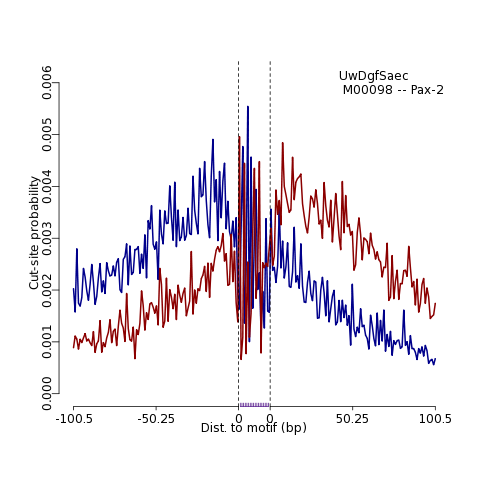

Supplement: S3 File — For each motif, footprint profiles are aggregated across all binding sites in all 653 DNase-seq samples. Color indicates which strand the motif matches, positive (blue) or negative (red). Text in the upper left denotes the tissue with the highest Z-score from the CENTIPEDE mode, the motif ID, and the corresponding transcription factor. (GZ) [file pgen.1005875.s004.tar.gz › recalibratedMotifShape/M00098.lambda.png]

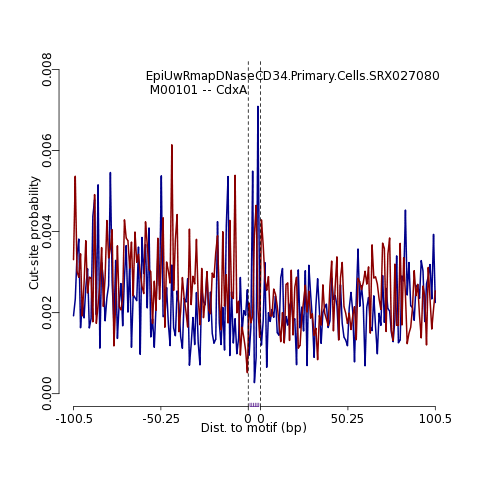

Supplement: S3 File — For each motif, footprint profiles are aggregated across all binding sites in all 653 DNase-seq samples. Color indicates which strand the motif matches, positive (blue) or negative (red). Text in the upper left denotes the tissue with the highest Z-score from the CENTIPEDE mode, the motif ID, and the corresponding transcription factor. (GZ) [file pgen.1005875.s004.tar.gz › recalibratedMotifShape/M00101.lambda.png]

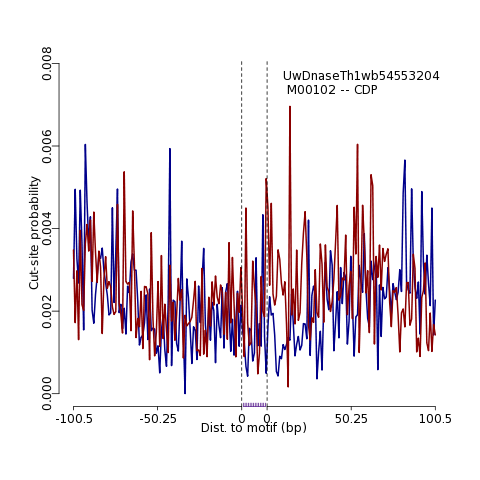

Supplement: S3 File — For each motif, footprint profiles are aggregated across all binding sites in all 653 DNase-seq samples. Color indicates which strand the motif matches, positive (blue) or negative (red). Text in the upper left denotes the tissue with the highest Z-score from the CENTIPEDE mode, the motif ID, and the corresponding transcription factor. (GZ) [file pgen.1005875.s004.tar.gz › recalibratedMotifShape/M00102.lambda.png]

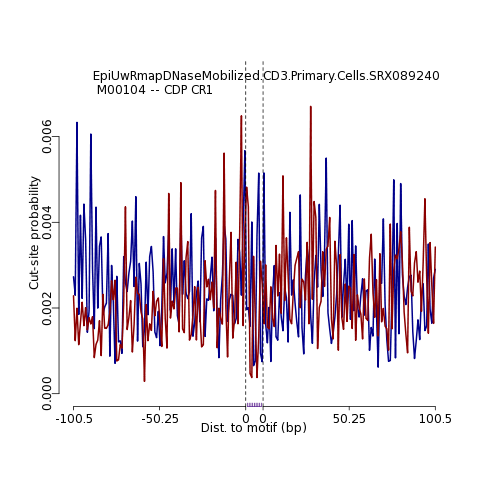

Supplement: S3 File — For each motif, footprint profiles are aggregated across all binding sites in all 653 DNase-seq samples. Color indicates which strand the motif matches, positive (blue) or negative (red). Text in the upper left denotes the tissue with the highest Z-score from the CENTIPEDE mode, the motif ID, and the corresponding transcription factor. (GZ) [file pgen.1005875.s004.tar.gz › recalibratedMotifShape/M00104.lambda.png]

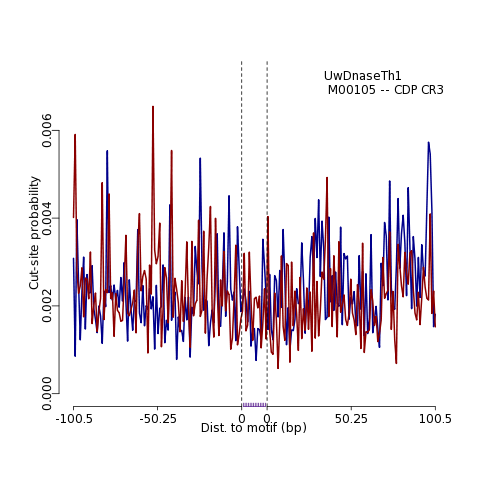

Supplement: S3 File — For each motif, footprint profiles are aggregated across all binding sites in all 653 DNase-seq samples. Color indicates which strand the motif matches, positive (blue) or negative (red). Text in the upper left denotes the tissue with the highest Z-score from the CENTIPEDE mode, the motif ID, and the corresponding transcription factor. (GZ) [file pgen.1005875.s004.tar.gz › recalibratedMotifShape/M00105.lambda.png]

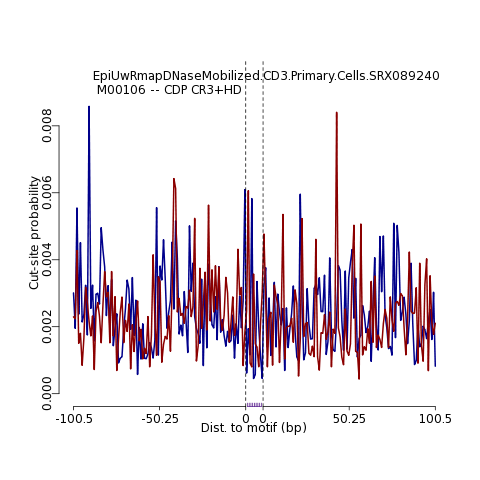

Supplement: S3 File — For each motif, footprint profiles are aggregated across all binding sites in all 653 DNase-seq samples. Color indicates which strand the motif matches, positive (blue) or negative (red). Text in the upper left denotes the tissue with the highest Z-score from the CENTIPEDE mode, the motif ID, and the corresponding transcription factor. (GZ) [file pgen.1005875.s004.tar.gz › recalibratedMotifShape/M00106.lambda.png]

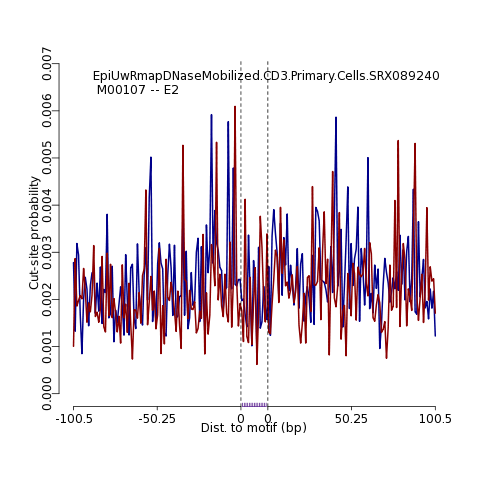

Supplement: S3 File — For each motif, footprint profiles are aggregated across all binding sites in all 653 DNase-seq samples. Color indicates which strand the motif matches, positive (blue) or negative (red). Text in the upper left denotes the tissue with the highest Z-score from the CENTIPEDE mode, the motif ID, and the corresponding transcription factor. (GZ) [file pgen.1005875.s004.tar.gz › recalibratedMotifShape/M00107.lambda.png]

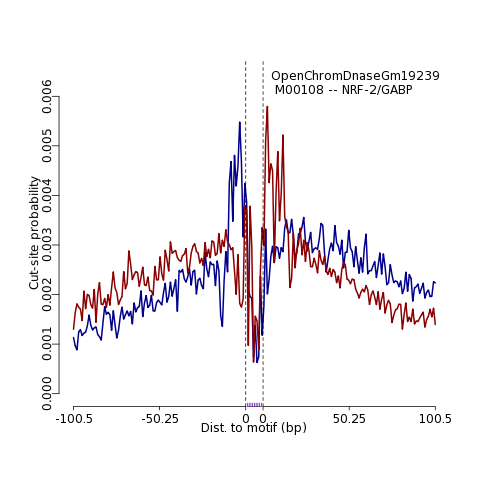

Supplement: S3 File — For each motif, footprint profiles are aggregated across all binding sites in all 653 DNase-seq samples. Color indicates which strand the motif matches, positive (blue) or negative (red). Text in the upper left denotes the tissue with the highest Z-score from the CENTIPEDE mode, the motif ID, and the corresponding transcription factor. (GZ) [file pgen.1005875.s004.tar.gz › recalibratedMotifShape/M00108.lambda.png]

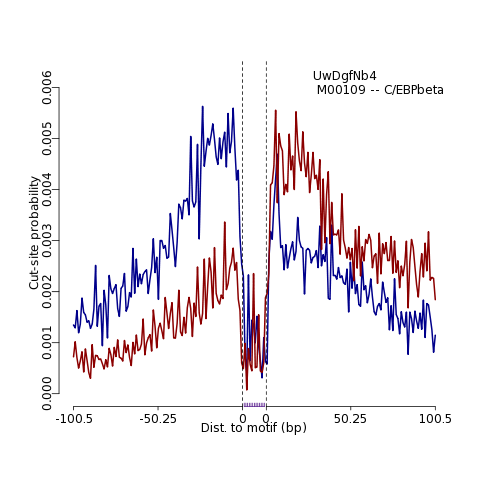

Supplement: S3 File — For each motif, footprint profiles are aggregated across all binding sites in all 653 DNase-seq samples. Color indicates which strand the motif matches, positive (blue) or negative (red). Text in the upper left denotes the tissue with the highest Z-score from the CENTIPEDE mode, the motif ID, and the corresponding transcription factor. (GZ) [file pgen.1005875.s004.tar.gz › recalibratedMotifShape/M00109.lambda.png]

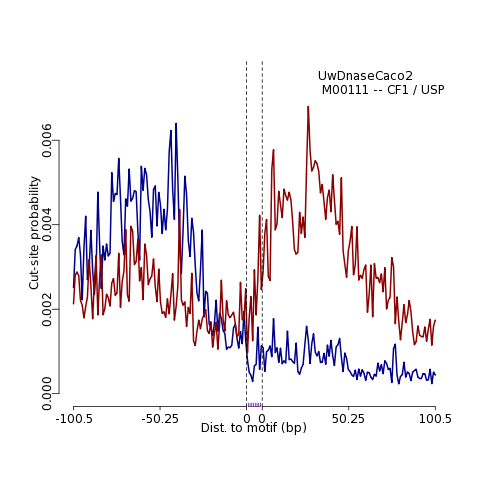

Supplement: S3 File — For each motif, footprint profiles are aggregated across all binding sites in all 653 DNase-seq samples. Color indicates which strand the motif matches, positive (blue) or negative (red). Text in the upper left denotes the tissue with the highest Z-score from the CENTIPEDE mode, the motif ID, and the corresponding transcription factor. (GZ) [file pgen.1005875.s004.tar.gz › recalibratedMotifShape/M00111.lambda.png]

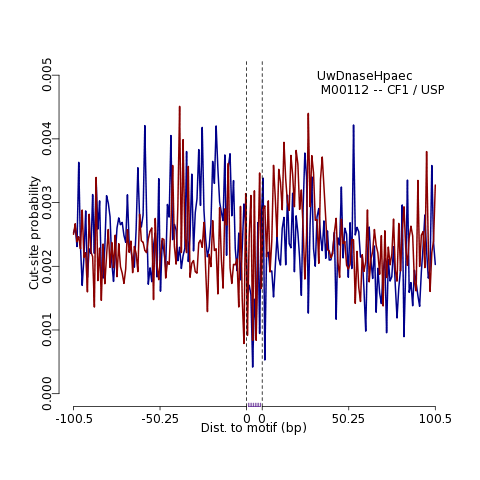

Supplement: S3 File — For each motif, footprint profiles are aggregated across all binding sites in all 653 DNase-seq samples. Color indicates which strand the motif matches, positive (blue) or negative (red). Text in the upper left denotes the tissue with the highest Z-score from the CENTIPEDE mode, the motif ID, and the corresponding transcription factor. (GZ) [file pgen.1005875.s004.tar.gz › recalibratedMotifShape/M00112.lambda.png]

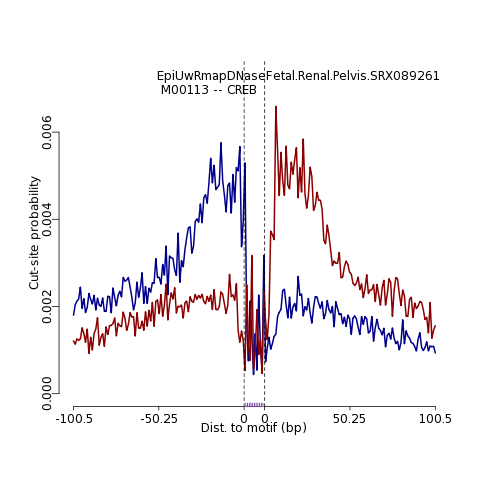

Supplement: S3 File — For each motif, footprint profiles are aggregated across all binding sites in all 653 DNase-seq samples. Color indicates which strand the motif matches, positive (blue) or negative (red). Text in the upper left denotes the tissue with the highest Z-score from the CENTIPEDE mode, the motif ID, and the corresponding transcription factor. (GZ) [file pgen.1005875.s004.tar.gz › recalibratedMotifShape/M00113.lambda.png]

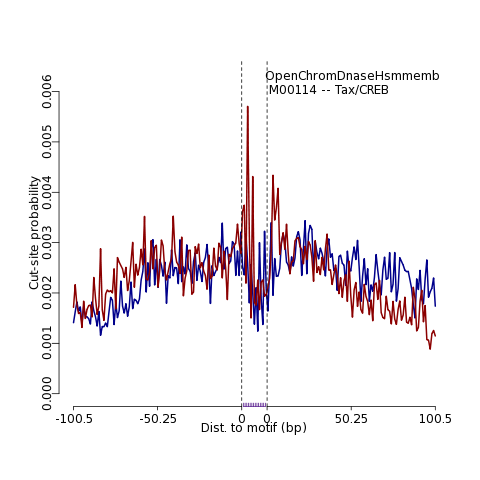

Supplement: S3 File — For each motif, footprint profiles are aggregated across all binding sites in all 653 DNase-seq samples. Color indicates which strand the motif matches, positive (blue) or negative (red). Text in the upper left denotes the tissue with the highest Z-score from the CENTIPEDE mode, the motif ID, and the corresponding transcription factor. (GZ) [file pgen.1005875.s004.tar.gz › recalibratedMotifShape/M00114.lambda.png]

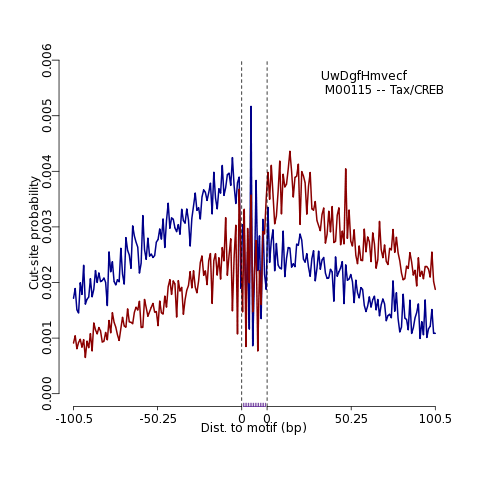

Supplement: S3 File — For each motif, footprint profiles are aggregated across all binding sites in all 653 DNase-seq samples. Color indicates which strand the motif matches, positive (blue) or negative (red). Text in the upper left denotes the tissue with the highest Z-score from the CENTIPEDE mode, the motif ID, and the corresponding transcription factor. (GZ) [file pgen.1005875.s004.tar.gz › recalibratedMotifShape/M00115.lambda.png]

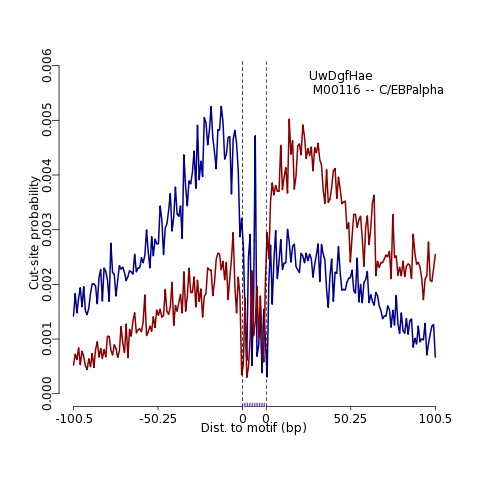

Supplement: S3 File — For each motif, footprint profiles are aggregated across all binding sites in all 653 DNase-seq samples. Color indicates which strand the motif matches, positive (blue) or negative (red). Text in the upper left denotes the tissue with the highest Z-score from the CENTIPEDE mode, the motif ID, and the corresponding transcription factor. (GZ) [file pgen.1005875.s004.tar.gz › recalibratedMotifShape/M00116.lambda.png]

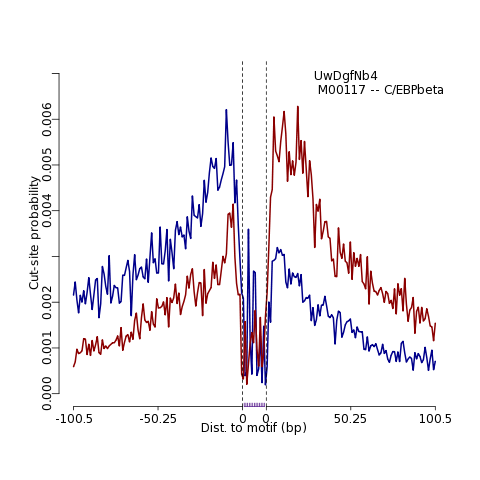

Supplement: S3 File — For each motif, footprint profiles are aggregated across all binding sites in all 653 DNase-seq samples. Color indicates which strand the motif matches, positive (blue) or negative (red). Text in the upper left denotes the tissue with the highest Z-score from the CENTIPEDE mode, the motif ID, and the corresponding transcription factor. (GZ) [file pgen.1005875.s004.tar.gz › recalibratedMotifShape/M00117.lambda.png]

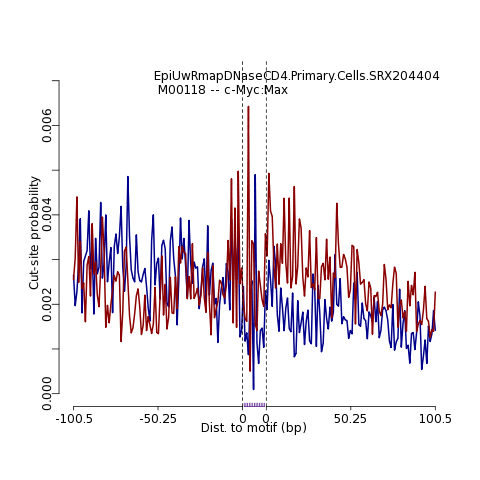

Supplement: S3 File — For each motif, footprint profiles are aggregated across all binding sites in all 653 DNase-seq samples. Color indicates which strand the motif matches, positive (blue) or negative (red). Text in the upper left denotes the tissue with the highest Z-score from the CENTIPEDE mode, the motif ID, and the corresponding transcription factor. (GZ) [file pgen.1005875.s004.tar.gz › recalibratedMotifShape/M00118.lambda.png]

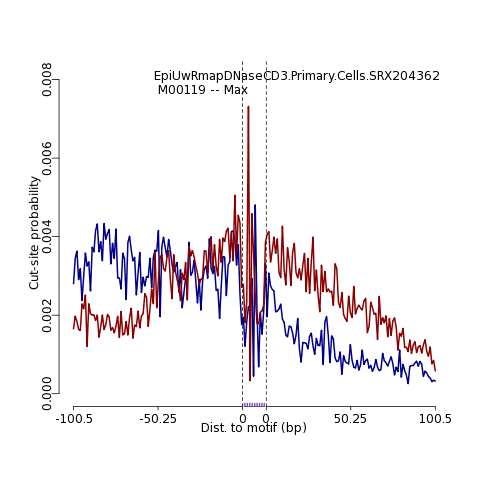

Supplement: S3 File — For each motif, footprint profiles are aggregated across all binding sites in all 653 DNase-seq samples. Color indicates which strand the motif matches, positive (blue) or negative (red). Text in the upper left denotes the tissue with the highest Z-score from the CENTIPEDE mode, the motif ID, and the corresponding transcription factor. (GZ) [file pgen.1005875.s004.tar.gz › recalibratedMotifShape/M00119.lambda.png]

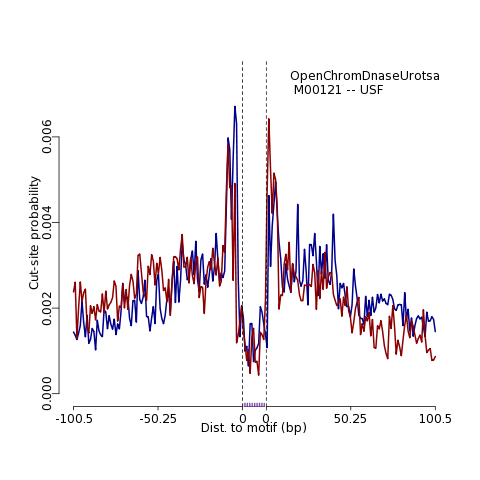

Supplement: S3 File — For each motif, footprint profiles are aggregated across all binding sites in all 653 DNase-seq samples. Color indicates which strand the motif matches, positive (blue) or negative (red). Text in the upper left denotes the tissue with the highest Z-score from the CENTIPEDE mode, the motif ID, and the corresponding transcription factor. (GZ) [file pgen.1005875.s004.tar.gz › recalibratedMotifShape/M00121.lambda.png]

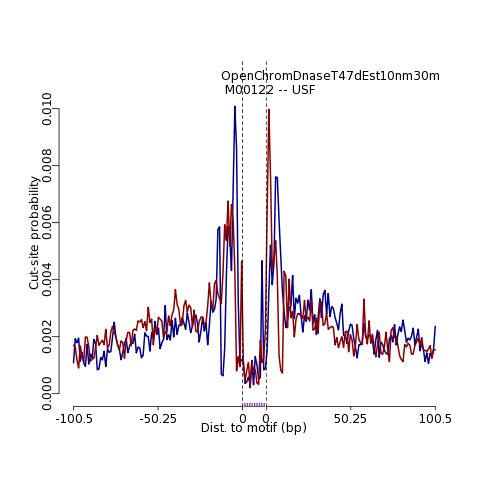

Supplement: S3 File — For each motif, footprint profiles are aggregated across all binding sites in all 653 DNase-seq samples. Color indicates which strand the motif matches, positive (blue) or negative (red). Text in the upper left denotes the tissue with the highest Z-score from the CENTIPEDE mode, the motif ID, and the corresponding transcription factor. (GZ) [file pgen.1005875.s004.tar.gz › recalibratedMotifShape/M00122.lambda.png]

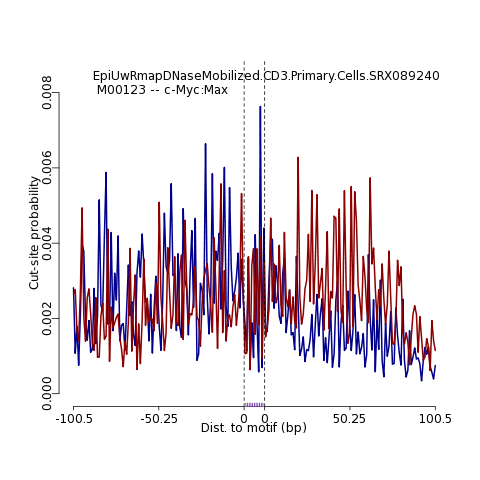

Supplement: S3 File — For each motif, footprint profiles are aggregated across all binding sites in all 653 DNase-seq samples. Color indicates which strand the motif matches, positive (blue) or negative (red). Text in the upper left denotes the tissue with the highest Z-score from the CENTIPEDE mode, the motif ID, and the corresponding transcription factor. (GZ) [file pgen.1005875.s004.tar.gz › recalibratedMotifShape/M00123.lambda.png]

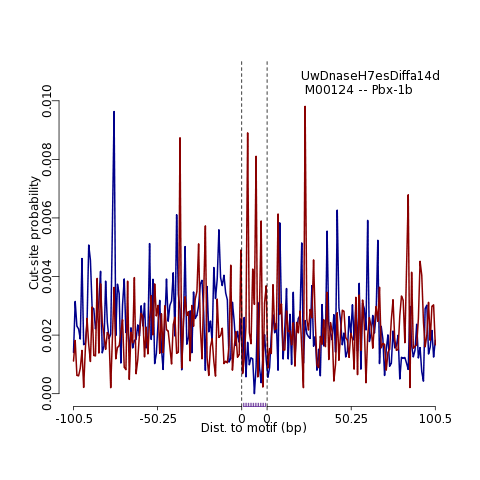

Supplement: S3 File — For each motif, footprint profiles are aggregated across all binding sites in all 653 DNase-seq samples. Color indicates which strand the motif matches, positive (blue) or negative (red). Text in the upper left denotes the tissue with the highest Z-score from the CENTIPEDE mode, the motif ID, and the corresponding transcription factor. (GZ) [file pgen.1005875.s004.tar.gz › recalibratedMotifShape/M00124.lambda.png]

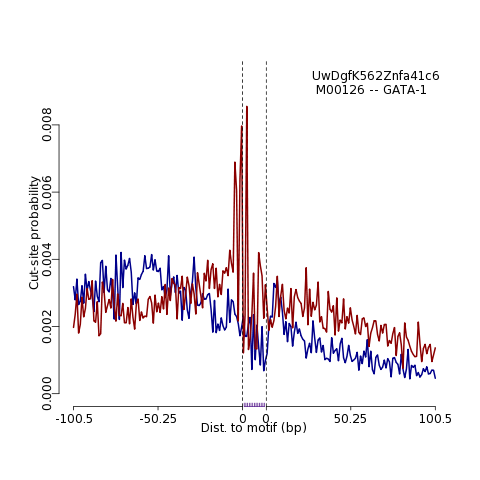

Supplement: S3 File — For each motif, footprint profiles are aggregated across all binding sites in all 653 DNase-seq samples. Color indicates which strand the motif matches, positive (blue) or negative (red). Text in the upper left denotes the tissue with the highest Z-score from the CENTIPEDE mode, the motif ID, and the corresponding transcription factor. (GZ) [file pgen.1005875.s004.tar.gz › recalibratedMotifShape/M00126.lambda.png]

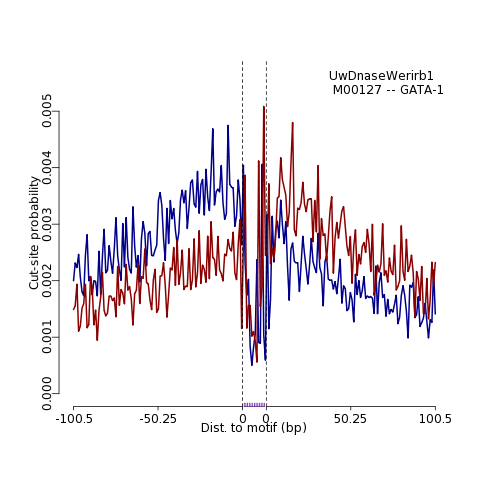

Supplement: S3 File — For each motif, footprint profiles are aggregated across all binding sites in all 653 DNase-seq samples. Color indicates which strand the motif matches, positive (blue) or negative (red). Text in the upper left denotes the tissue with the highest Z-score from the CENTIPEDE mode, the motif ID, and the corresponding transcription factor. (GZ) [file pgen.1005875.s004.tar.gz › recalibratedMotifShape/M00127.lambda.png]

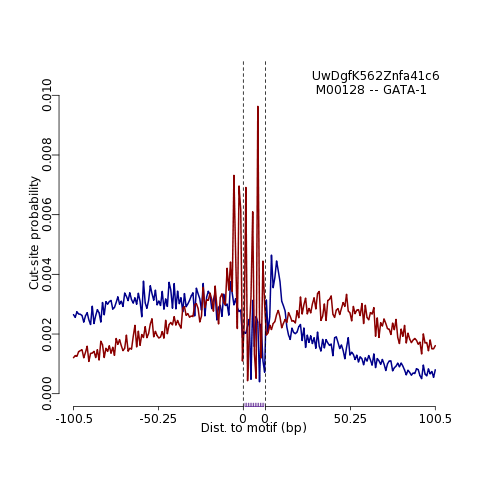

Supplement: S3 File — For each motif, footprint profiles are aggregated across all binding sites in all 653 DNase-seq samples. Color indicates which strand the motif matches, positive (blue) or negative (red). Text in the upper left denotes the tissue with the highest Z-score from the CENTIPEDE mode, the motif ID, and the corresponding transcription factor. (GZ) [file pgen.1005875.s004.tar.gz › recalibratedMotifShape/M00128.lambda.png]

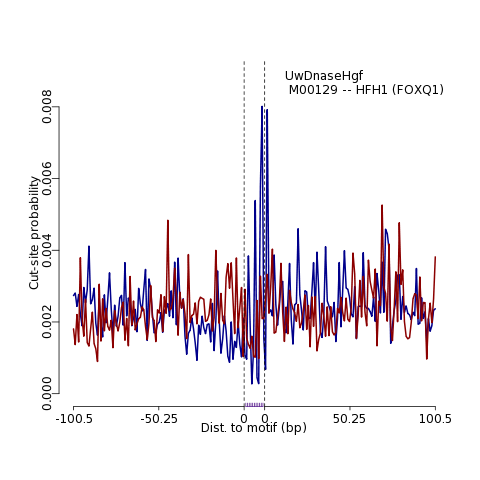

Supplement: S3 File — For each motif, footprint profiles are aggregated across all binding sites in all 653 DNase-seq samples. Color indicates which strand the motif matches, positive (blue) or negative (red). Text in the upper left denotes the tissue with the highest Z-score from the CENTIPEDE mode, the motif ID, and the corresponding transcription factor. (GZ) [file pgen.1005875.s004.tar.gz › recalibratedMotifShape/M00129.lambda.png]

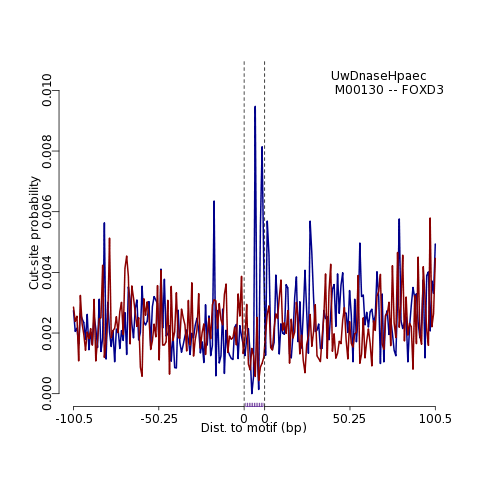

Supplement: S3 File — For each motif, footprint profiles are aggregated across all binding sites in all 653 DNase-seq samples. Color indicates which strand the motif matches, positive (blue) or negative (red). Text in the upper left denotes the tissue with the highest Z-score from the CENTIPEDE mode, the motif ID, and the corresponding transcription factor. (GZ) [file pgen.1005875.s004.tar.gz › recalibratedMotifShape/M00130.lambda.png]

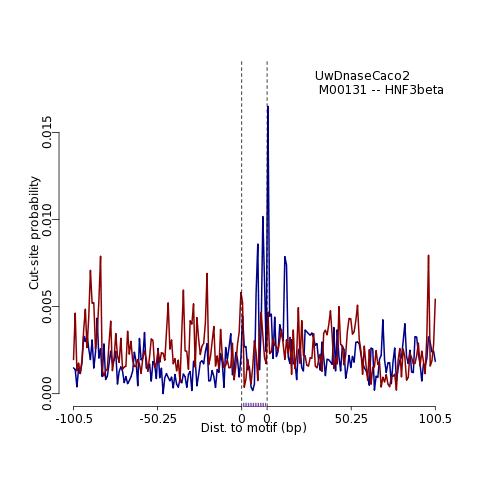

Supplement: S3 File — For each motif, footprint profiles are aggregated across all binding sites in all 653 DNase-seq samples. Color indicates which strand the motif matches, positive (blue) or negative (red). Text in the upper left denotes the tissue with the highest Z-score from the CENTIPEDE mode, the motif ID, and the corresponding transcription factor. (GZ) [file pgen.1005875.s004.tar.gz › recalibratedMotifShape/M00131.lambda.png]

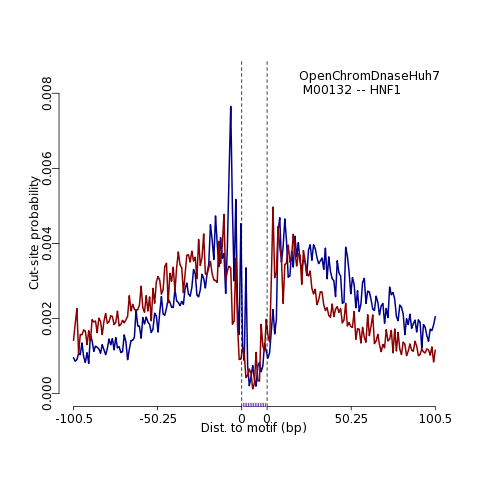

Supplement: S3 File — For each motif, footprint profiles are aggregated across all binding sites in all 653 DNase-seq samples. Color indicates which strand the motif matches, positive (blue) or negative (red). Text in the upper left denotes the tissue with the highest Z-score from the CENTIPEDE mode, the motif ID, and the corresponding transcription factor. (GZ) [file pgen.1005875.s004.tar.gz › recalibratedMotifShape/M00132.lambda.png]

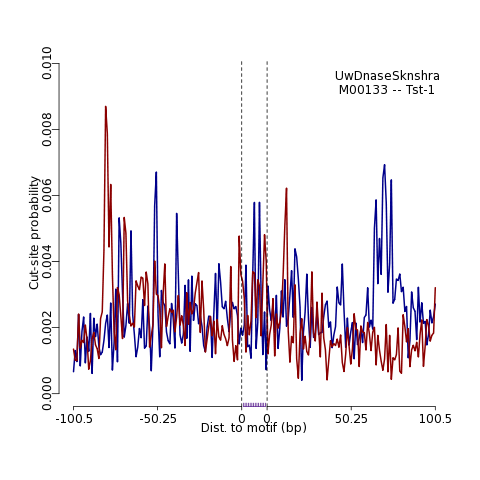

Supplement: S3 File — For each motif, footprint profiles are aggregated across all binding sites in all 653 DNase-seq samples. Color indicates which strand the motif matches, positive (blue) or negative (red). Text in the upper left denotes the tissue with the highest Z-score from the CENTIPEDE mode, the motif ID, and the corresponding transcription factor. (GZ) [file pgen.1005875.s004.tar.gz › recalibratedMotifShape/M00133.lambda.png]

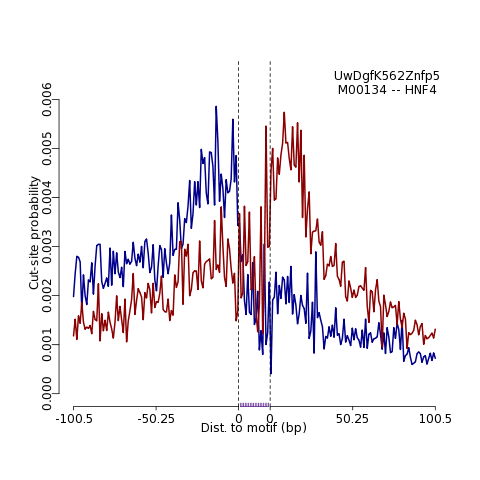

Supplement: S3 File — For each motif, footprint profiles are aggregated across all binding sites in all 653 DNase-seq samples. Color indicates which strand the motif matches, positive (blue) or negative (red). Text in the upper left denotes the tissue with the highest Z-score from the CENTIPEDE mode, the motif ID, and the corresponding transcription factor. (GZ) [file pgen.1005875.s004.tar.gz › recalibratedMotifShape/M00134.lambda.png]

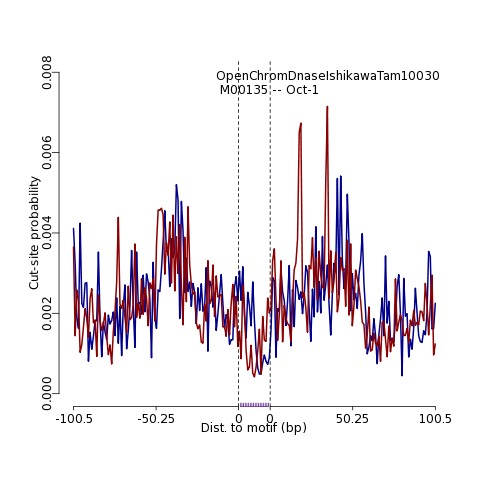

Supplement: S3 File — For each motif, footprint profiles are aggregated across all binding sites in all 653 DNase-seq samples. Color indicates which strand the motif matches, positive (blue) or negative (red). Text in the upper left denotes the tissue with the highest Z-score from the CENTIPEDE mode, the motif ID, and the corresponding transcription factor. (GZ) [file pgen.1005875.s004.tar.gz › recalibratedMotifShape/M00135.lambda.png]

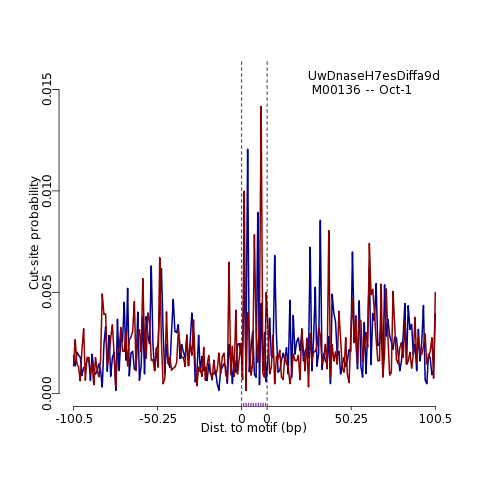

Supplement: S3 File — For each motif, footprint profiles are aggregated across all binding sites in all 653 DNase-seq samples. Color indicates which strand the motif matches, positive (blue) or negative (red). Text in the upper left denotes the tissue with the highest Z-score from the CENTIPEDE mode, the motif ID, and the corresponding transcription factor. (GZ) [file pgen.1005875.s004.tar.gz › recalibratedMotifShape/M00136.lambda.png]

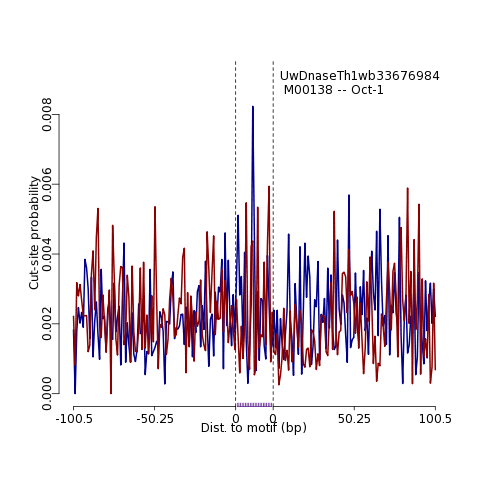

Supplement: S3 File — For each motif, footprint profiles are aggregated across all binding sites in all 653 DNase-seq samples. Color indicates which strand the motif matches, positive (blue) or negative (red). Text in the upper left denotes the tissue with the highest Z-score from the CENTIPEDE mode, the motif ID, and the corresponding transcription factor. (GZ) [file pgen.1005875.s004.tar.gz › recalibratedMotifShape/M00138.lambda.png]
