# Supplementary material for: Which Genetics Variants in DNase-Seq Footprints Are More Likely to Alter Binding?
Source: PLoS Genet. 2016 Feb 22;12(2):e1005875. doi: 10.1371/journal.pgen.1005875 (PMC4764260; doi:10.1371/journal.pgen.1005875)
Supplement: S1 Fig — Numbers next to boxes refer to the corresponding section in the Supplement. (PDF) [file pgen.1005875.s022.pdf]

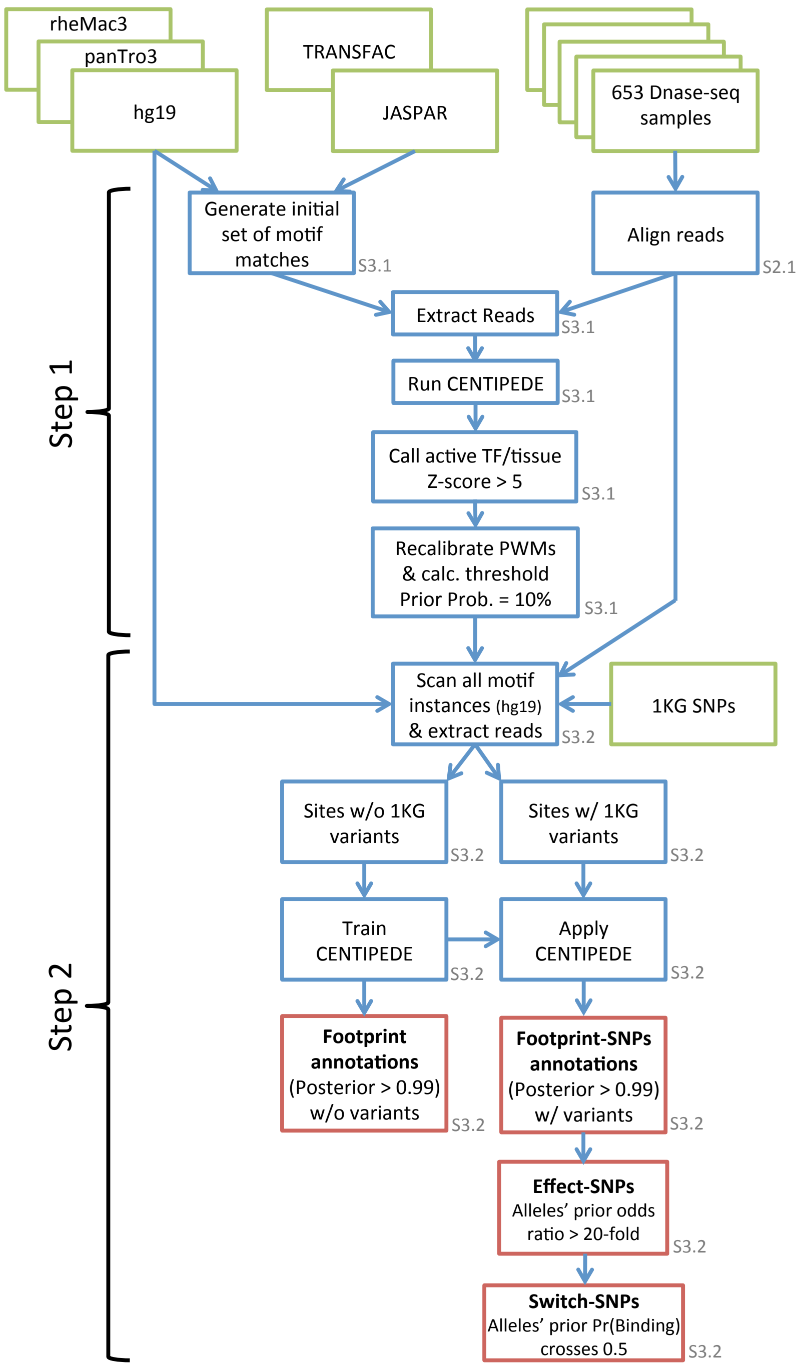

Figure S1: **Flowchart detailing steps of the CENTIPEDE-based annotation of regulatory regions and variants.** Numbers next to boxes refer to the corresponding section in the Supplement.
