# Supplementary material for: Which Genetics Variants in DNase-Seq Footprints Are More Likely to Alter Binding?
Source: PLoS Genet. 2016 Feb 22;12(2):e1005875. doi: 10.1371/journal.pgen.1005875 (PMC4764260; doi:10.1371/journal.pgen.1005875)
Supplement: S3 Fig — “Prior Odds Ratio > 20” is the same criteria as the one used to define effect-SNPs. Numbers next to boxes refer to the corresponding section in the Supplement. (PDF) [file pgen.1005875.s024.pdf]

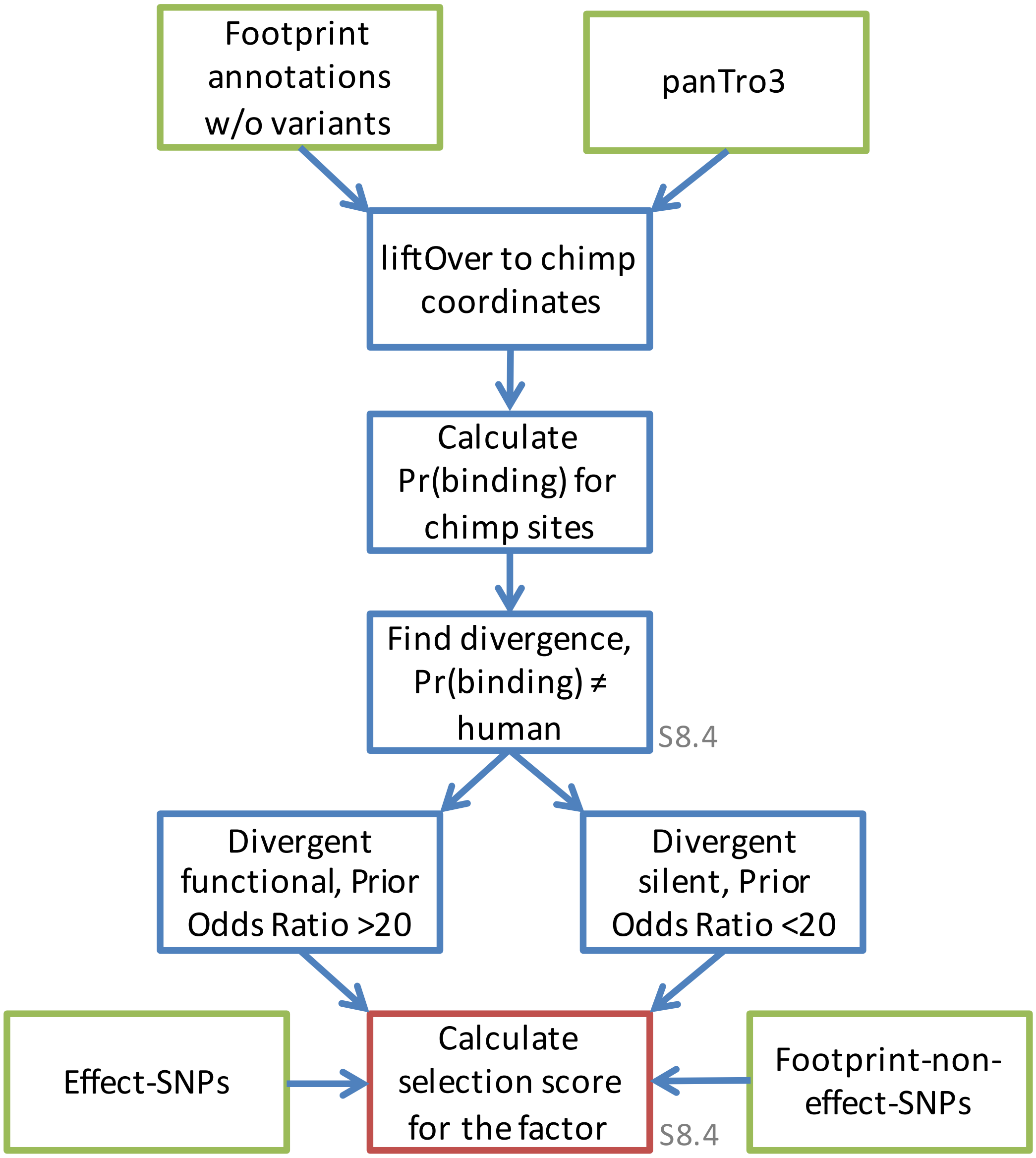

Figure S3: **Flowchart detailing analysis pipeline for identifying selection across TFBS.** "Prior Odds Ratio $>$ 20" is the same criteria as the one used to define effect-SNPs. Numbers next to boxes refer to the corresponding section in the Supplement.
