# Supplementary material for: Which Genetics Variants in DNase-Seq Footprints Are More Likely to Alter Binding?
Source: PLoS Genet. 2016 Feb 22;12(2):e1005875. doi: 10.1371/journal.pgen.1005875 (PMC4764260; doi:10.1371/journal.pgen.1005875)
Supplement: S4 Fig — Footprint profiles are aggregated across all binding sites in all 653 DNase-seq samples, and stratified by Z-score (color). The higher the Z-score, the more likely a factor is bound as predicted by the CENTIPEDE model. (PDF) [file pgen.1005875.s025.pdf]

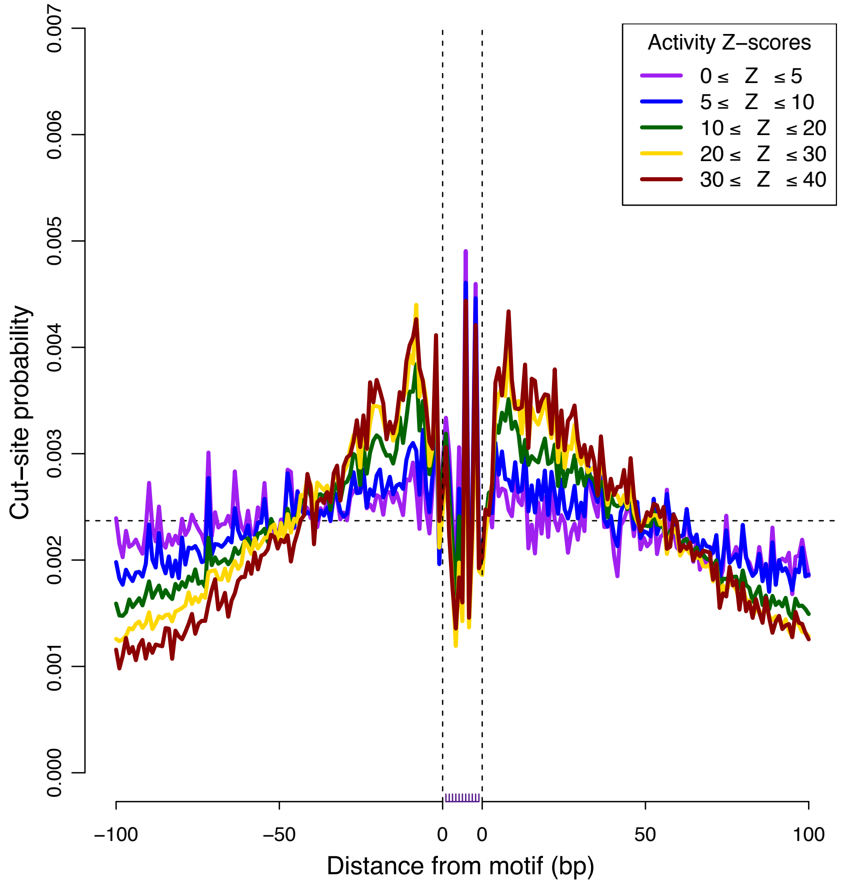

Figure S4: **Binding profiles of AP-1 motif M00172.** Footprint profiles are aggregated across all binding sites in all 653 DNase-seq samples, and stratified by Z-score (color). The higher the Z-score, the more likely a factor is bound as predicted by the CENTIPEDE model.
