# Supplementary material for: Which Genetics Variants in DNase-Seq Footprints Are More Likely to Alter Binding?
Source: PLoS Genet. 2016 Feb 22;12(2):e1005875. doi: 10.1371/journal.pgen.1005875 (PMC4764260; doi:10.1371/journal.pgen.1005875)
Supplement: S5 Fig — Shown is the full distribution of Z-scores (calculated with Equation 2 in S1 Text) across every sample-motif pair. The dotted vertical line at Z = 5 shows the selected threshold for factor activity. (PDF) [file pgen.1005875.s026.pdf]

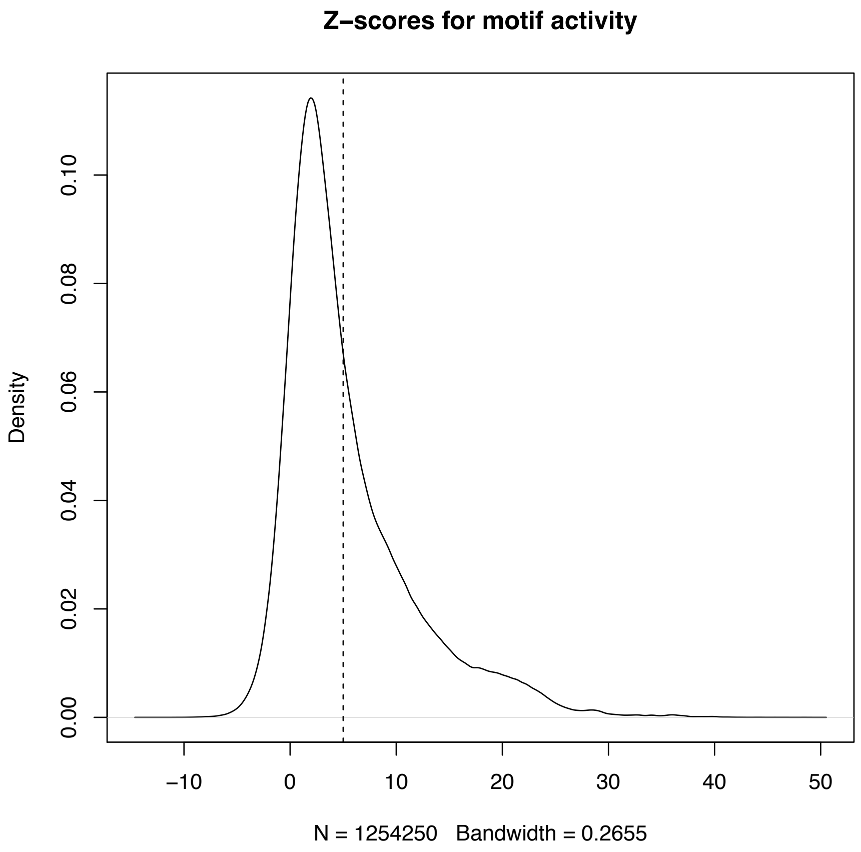

Figure S5: **Distribution of Z-scores across samples and motifs.** Shown is the full distribution of Z-scores (calculated with eq. 2) across every sample-motif pair. The dotted vertical line at $Z = 5$ shows the selected threshold for factor activity.
