# Supplementary material for: Which Genetics Variants in DNase-Seq Footprints Are More Likely to Alter Binding?
Source: PLoS Genet. 2016 Feb 22;12(2):e1005875. doi: 10.1371/journal.pgen.1005875 (PMC4764260; doi:10.1371/journal.pgen.1005875)
Supplement: S6 Fig — For each factor motif, shown is the original seed sequence model (left) and the revised model (right). x-axis: position within motif, y-axis: information content. (A) NRSF (B) CTCF (C) PU.1 (D) AP-1. (PDF) [file pgen.1005875.s027.pdf]

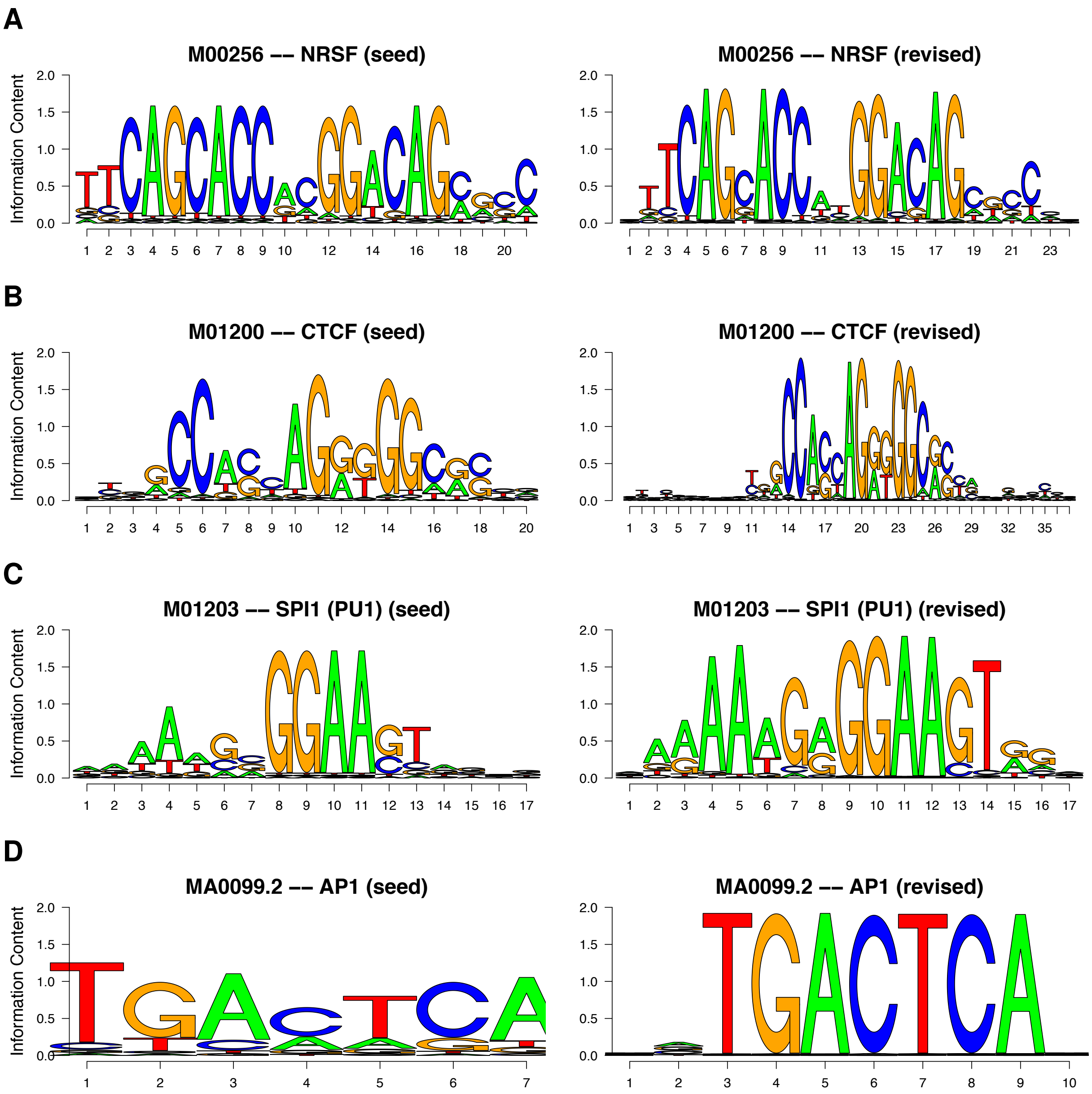

Figure S6: **Comparison between seed and revised sequence model.** For each factor motif, shown is the original seed sequence model (left) and the revised model (right). x-axis: position within motif, y-axis: information content. (A) NRSF (B) CTCF (C) PU.1 (D) AP-1
