# Supplementary material for: Which Genetics Variants in DNase-Seq Footprints Are More Likely to Alter Binding?
Source: PLoS Genet. 2016 Feb 22;12(2):e1005875. doi: 10.1371/journal.pgen.1005875 (PMC4764260; doi:10.1371/journal.pgen.1005875)
Supplement: S7 Fig — For each TF binding motif, CENTIPEDE-predicted footprints in GM12878 cells were compared using ENCODE ChIP-seq data as a gold standard. (A & B) CTCF (C & D) GABP (E & F) NRSF (G & H) PU.1. (PDF) [file pgen.1005875.s028.pdf]

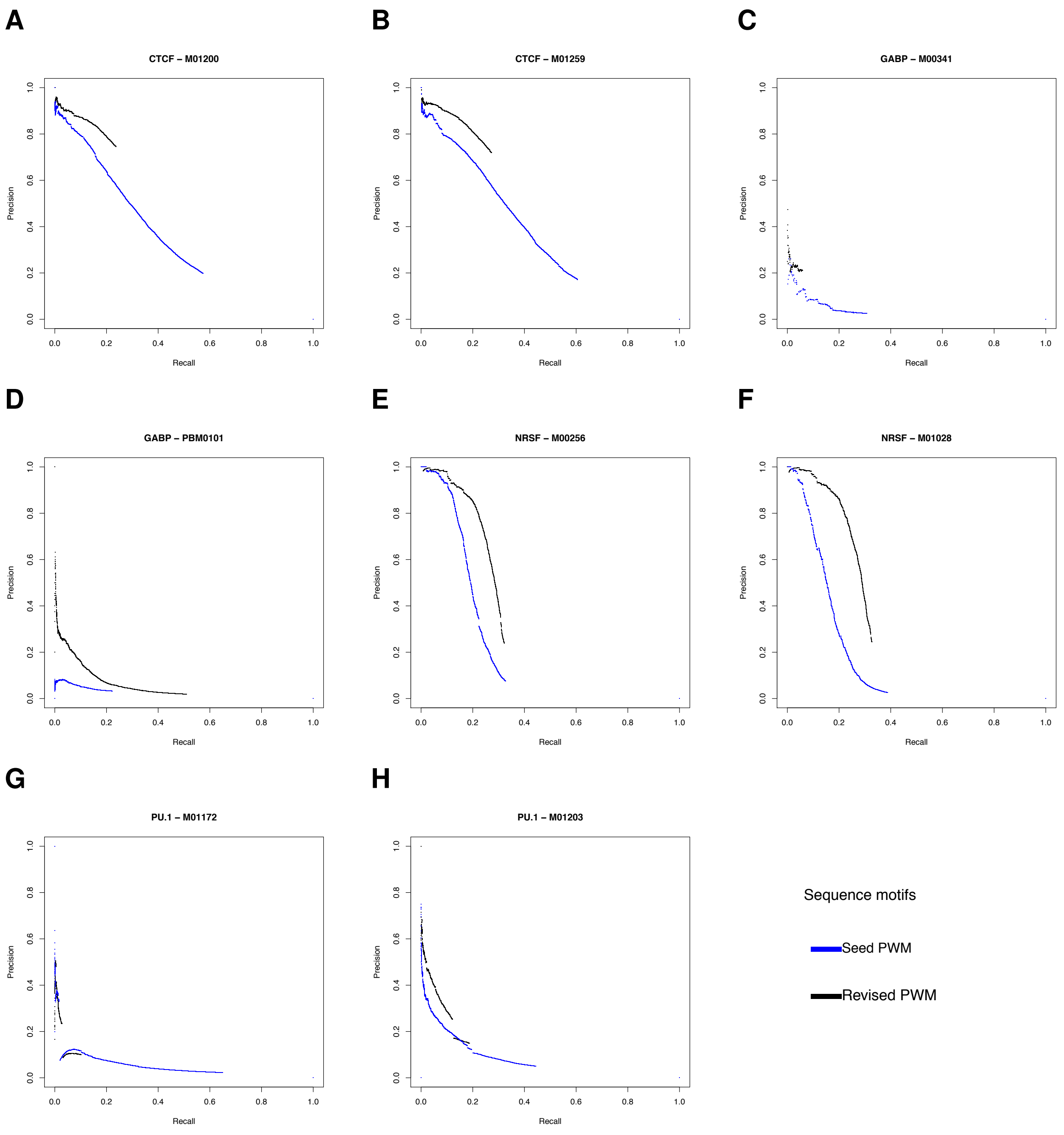

Figure S7: **Precision-recall curves for seed (blue) and revised (black) sequence models.** For each TF binding motif, CENTIPEDE-predicted footprints in GM12878 cells were compared using ENCODE ChIP-seq data as a gold standard. (A & B) CTCF (C & D) GABP (E & F) NRSF (G & H) PU.1
