# Supplementary material for: Which Genetics Variants in DNase-Seq Footprints Are More Likely to Alter Binding?
Source: PLoS Genet. 2016 Feb 22;12(2):e1005875. doi: 10.1371/journal.pgen.1005875 (PMC4764260; doi:10.1371/journal.pgen.1005875)
Supplement: S8 Fig — Due to thresholds on the match score (see Section 3.2 in S1 Text), few models have data Pr(binding) < 0.2. For ease of display data is binned in 10% increments. Points represent the average number of ChIP-seq reads within that bin and vertical lines represent the 95% confidence interval. Spearman correlation (legend) is calculated using the full data set without binning. (A & B) CTCF (C & D) NRSF (E & F) PU.1. (PDF) [file pgen.1005875.s029.pdf]

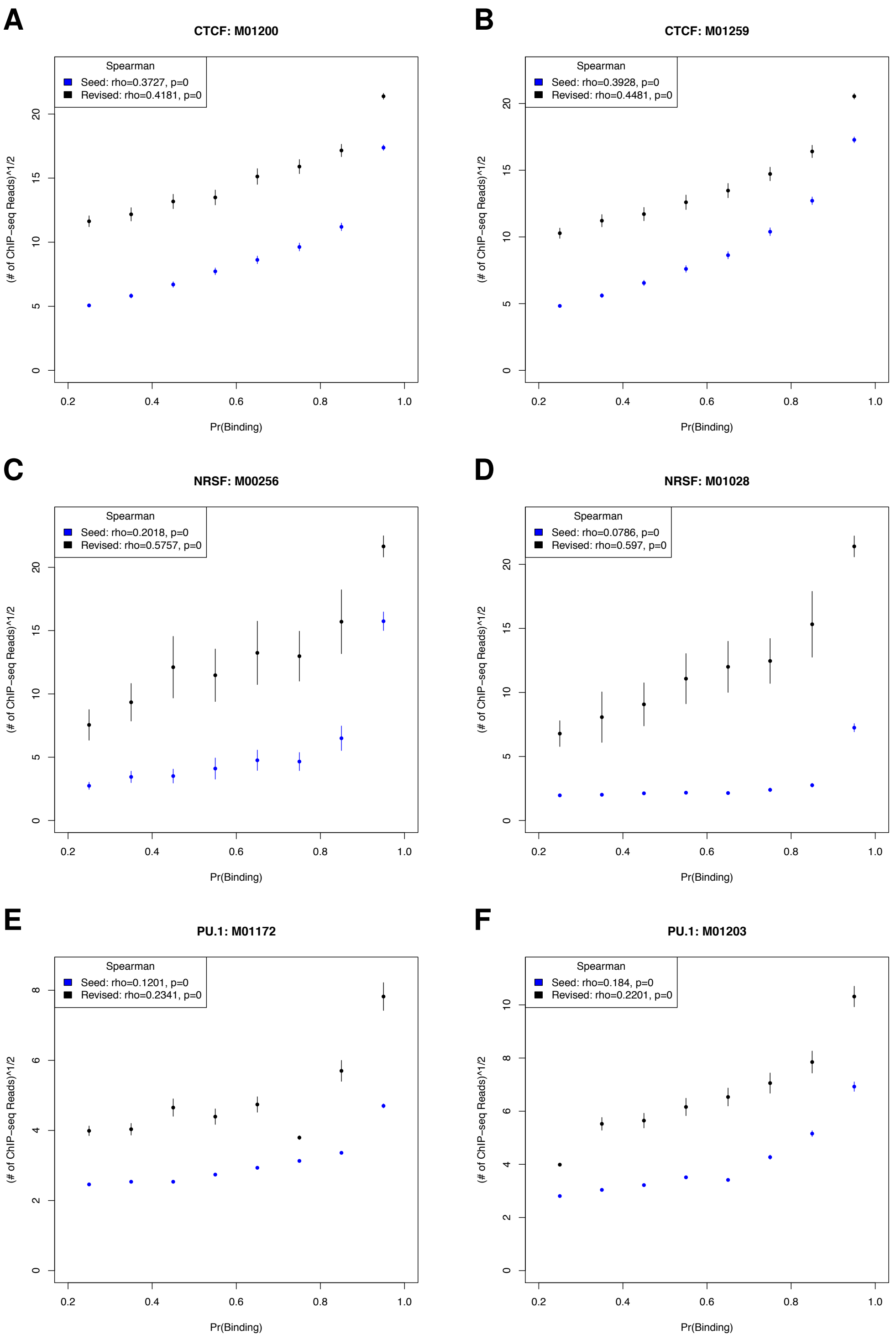

Figure S8: **Comparison of prior Pr(binding) derived from PWM scores to ChIP-seq read data across all motif matches using seed (blue) and revised (black) sequence models.** Due to thresholds on the match score (see Section S3.2), few models have data Pr(binding) < 0.2. For ease of display data is binned in 10% increments. Points represent the average number of ChIP-seq reads within that bin and vertical lines represent the 95% confidence interval. Spearman correlation (legend) is calculated using the full data set without binning. (A & B) CTCF, (C & D) NRSF, (E & F) PU.1
