# Supplementary material for: Which Genetics Variants in DNase-Seq Footprints Are More Likely to Alter Binding?
Source: PLoS Genet. 2016 Feb 22;12(2):e1005875. doi: 10.1371/journal.pgen.1005875 (PMC4764260; doi:10.1371/journal.pgen.1005875)
Supplement: S9 Fig — (A) Plot showing ρ allele ratios for SNPs interrogated for CD34 primary cells (used for ASH analysis). Three peaks on the histogram (right) correspond to homozygous reference (top), heterozygous (middle), and homozygous alternate (bottom) SNPs. (B) Plot showing ρ allele ratios for SNPs interrogated for the cancer line K562 (discarded for ASH analysis). Signatures of chromosomal abnormalities are evident from the scatterplot, such as copy number variation and loss of heterozygosity. (PDF) [file pgen.1005875.s030.pdf]

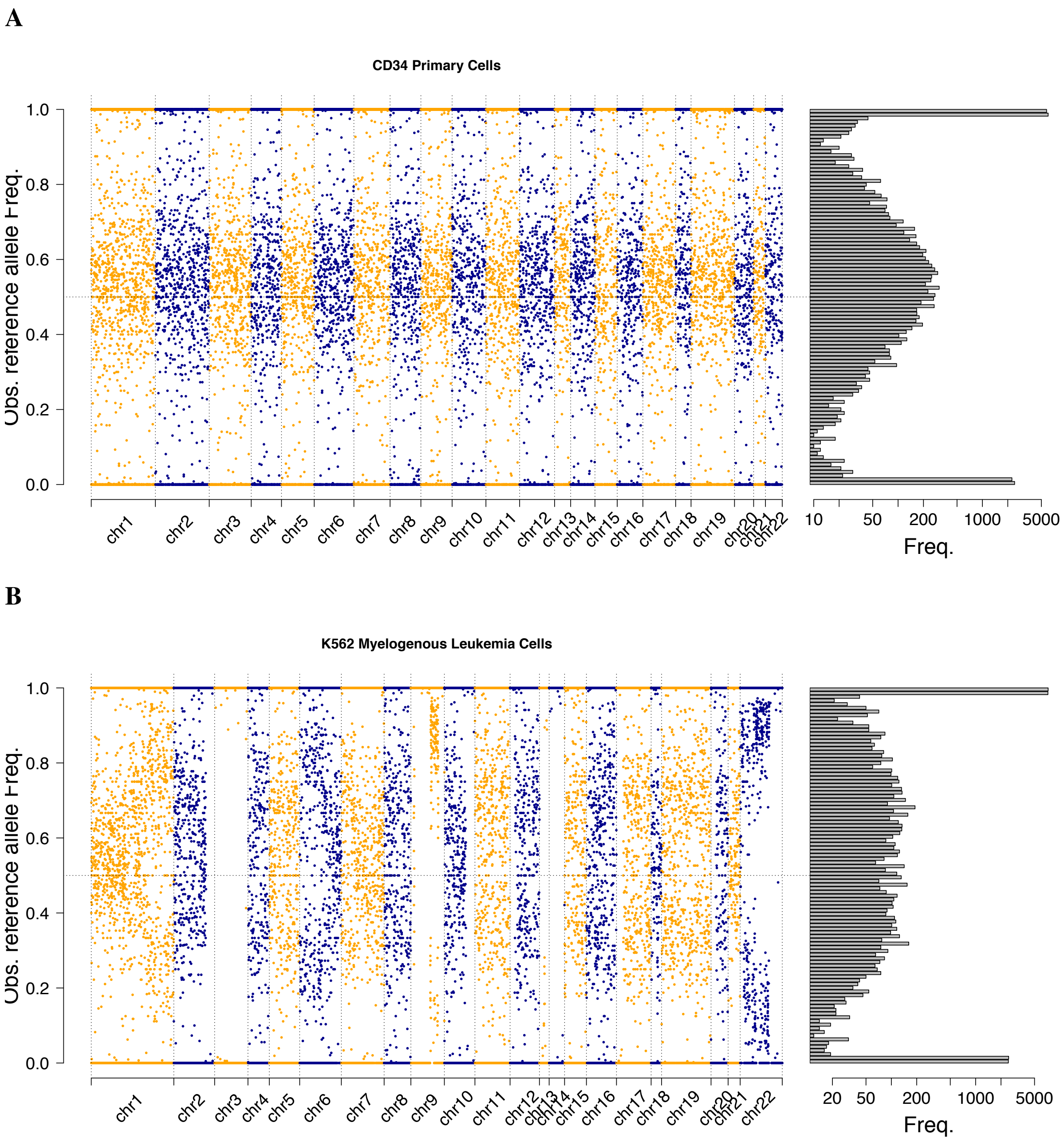

Figure S9: **Reference allele ratio ρ at 1KG variants.** (A) Plot showing ρ allele ratios for SNPs interrogated for CD34 primary cells (used for ASH analysis). Three peaks on the histogram (right) correspond to homozygous reference (top), heterozygous (middle), and homozygous alternate (bottom) SNPs. (B) Plot showing ρ allele ratios for SNPs interrogated for the cancer line K562 (discarded for ASH analysis). Signatures of chromosomal abnormalities are evident from the scatterplot, such as copy number variation and loss of heterozygosity.
