# Supplementary material for: Which Genetics Variants in DNase-Seq Footprints Are More Likely to Alter Binding?
Source: PLoS Genet. 2016 Feb 22;12(2):e1005875. doi: 10.1371/journal.pgen.1005875 (PMC4764260; doi:10.1371/journal.pgen.1005875)
Supplement: S10 Fig — On all four panels y-axis represents the parameter M that is reciprocally related to the dispersion of rho in the QuASAR model. Dotted lines represent values used to filter samples. (A) Dispersion and correlation between ρ and ϕ (B) Dispersion and ρ estimation. Bottom plots show zoomed view of samples with M < 100. (PDF) [file pgen.1005875.s031.pdf]

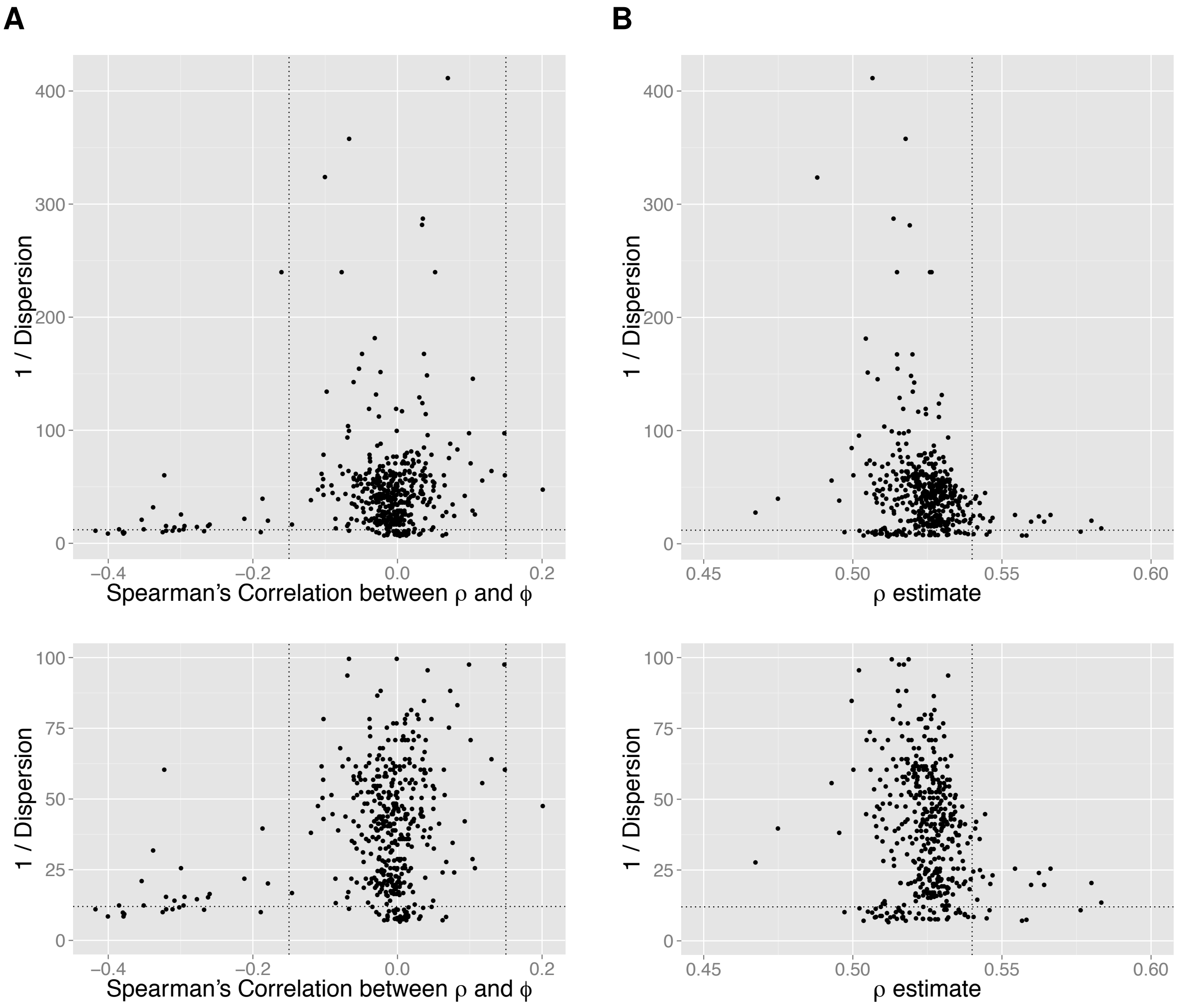

Figure S10: **Distribution of values used for post-ASH analysis filter criteria.** On all four panels y-axis represents the parameter M that is reciprocally related to the dispersion of rho in the QuASAR model. Dotted lines represent values used to filter samples. (A) Dispersion and correlation between ρ and ϕ (B) Dispersion and ρ estimation. Bottom plots show zoomed view of samples with $M < 100$.
