# Supplementary material for: Which Genetics Variants in DNase-Seq Footprints Are More Likely to Alter Binding?
Source: PLoS Genet. 2016 Feb 22;12(2):e1005875. doi: 10.1371/journal.pgen.1005875 (PMC4764260; doi:10.1371/journal.pgen.1005875)
Supplement: S11 Fig — SNPs identified in both the CENTIPEDE and ASH analysis are shown, shaded by p-value of allelic imbalance from QuASAR. Points circled in red display significant ASH at 20% FDR. The blue line is a logistic curve fit using points with a p < 0.1. (PDF) [file pgen.1005875.s032.pdf]

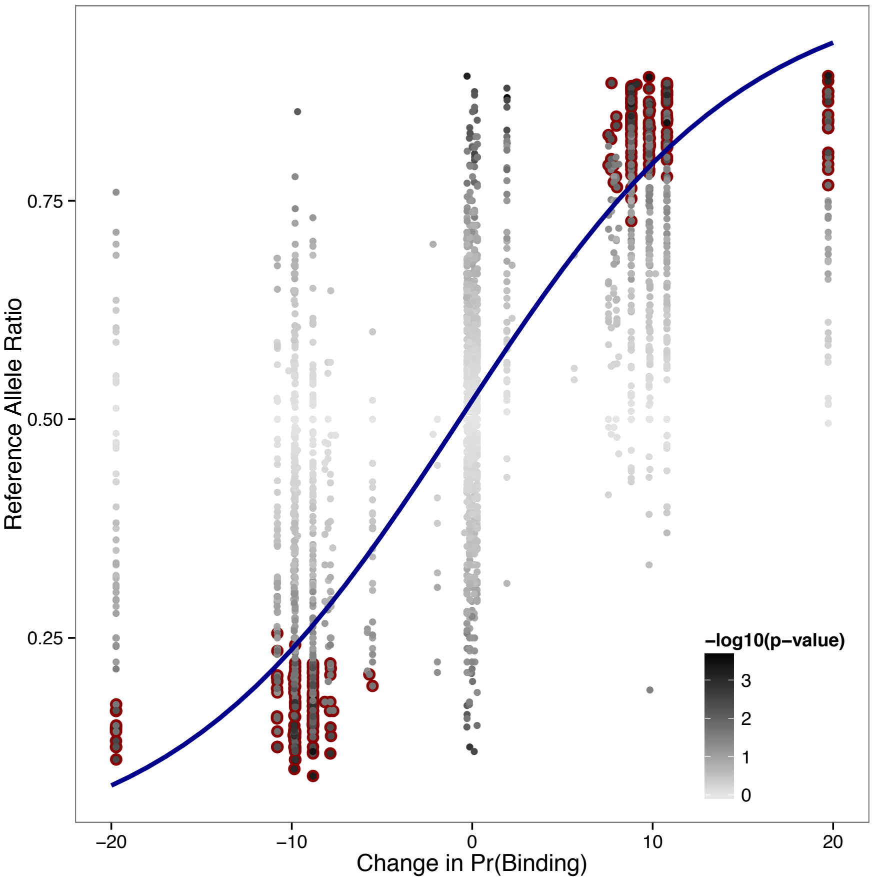

Figure S11: **Correlation between CENTIPEDE predictions and observed ASH.** SNPs identified in both the CENTIPEDE and ASH analysis are shown, shaded by p-value of allelic imbalance from QuASAR. Points circled in red display significant ASH at 20% FDR. The blue line is a logistic curve fit using points with a $p < 0.1$.
