# Supplementary material for: Which Genetics Variants in DNase-Seq Footprints Are More Likely to Alter Binding?
Source: PLoS Genet. 2016 Feb 22;12(2):e1005875. doi: 10.1371/journal.pgen.1005875 (PMC4764260; doi:10.1371/journal.pgen.1005875)
Supplement: S12 Fig — Each line represents a density plot of the magnitude of allelic imbalance ∣(allele ratio—0.5)∣ for SNPs within each functional annotation. (PDF) [file pgen.1005875.s033.pdf]

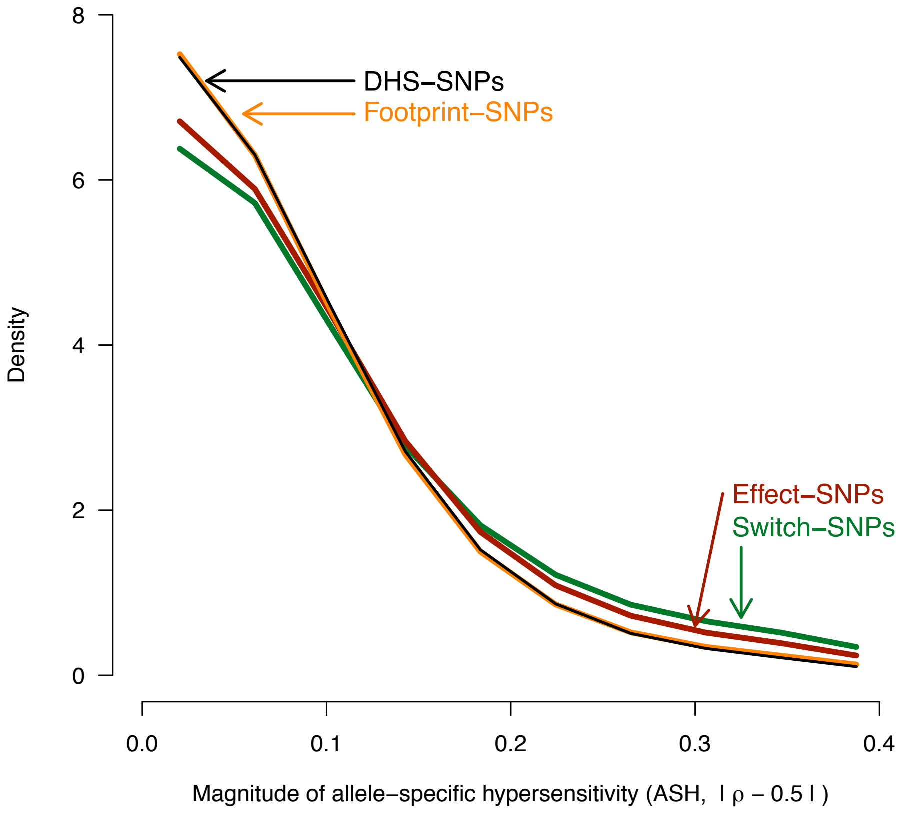

Figure S12: **Magnitude of allelic imbalance within predicted functional annotations**. Each line represents a density plot of the magnitude of allelic imbalance |(allele ratio - 0.5)| for SNPs within each functional annotation.
