# Supplementary material for: Which Genetics Variants in DNase-Seq Footprints Are More Likely to Alter Binding?
Source: PLoS Genet. 2016 Feb 22;12(2):e1005875. doi: 10.1371/journal.pgen.1005875 (PMC4764260; doi:10.1371/journal.pgen.1005875)
Supplement: S13 Fig — Shown are three additional categories of SNPs from recent studies of functional variation within TF binding sites. (PDF) [file pgen.1005875.s034.pdf]

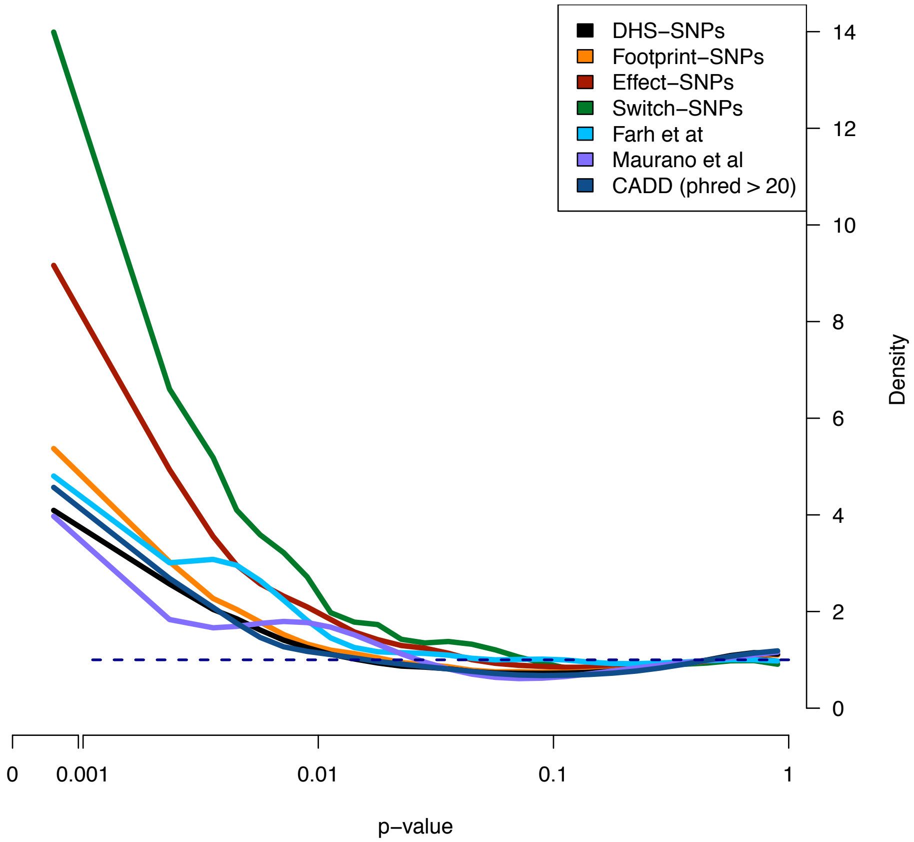

Figure S13: **ASH p-value densities for different SNP categories.** Shown are three additional categories of SNPs from recent studies of functional variation within TF binding sites.
