# Supplementary material for: Which Genetics Variants in DNase-Seq Footprints Are More Likely to Alter Binding?
Source: PLoS Genet. 2016 Feb 22;12(2):e1005875. doi: 10.1371/journal.pgen.1005875 (PMC4764260; doi:10.1371/journal.pgen.1005875)
Supplement: S14 Fig — ASH p-value densities for different SNP categories. For effect-SNPs and switch-SNPs, shown are different thresholds used for defining the category (20x is the threshold used throughout this analysis). The dotted blue line represents the null distribution. (PDF) [file pgen.1005875.s035.pdf]

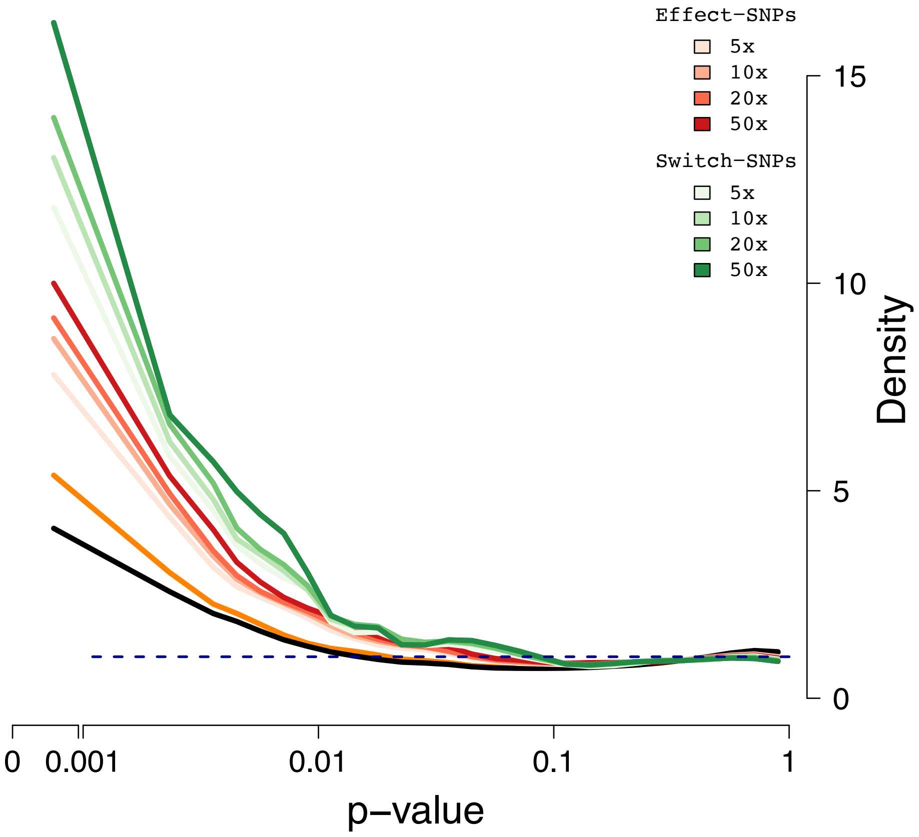

Figure S14: **Comparison of thresholds for functional annotation categories**. ASH p-value densities for different SNP categories. For effect-SNPs and switch-SNPs, shown are different thresholds used for defining the category (20x is the threshold used throughout this analysis). The dotted blue line represents the null distribution.
