# Supplementary material for: Which Genetics Variants in DNase-Seq Footprints Are More Likely to Alter Binding?
Source: PLoS Genet. 2016 Feb 22;12(2):e1005875. doi: 10.1371/journal.pgen.1005875 (PMC4764260; doi:10.1371/journal.pgen.1005875)
Supplement: S15 Fig — ASH p-value densities for different SNP categories and PWM (sequence match) match scores. Numbers in parentheses are the number of SNPs in those categories. The dotted blue line represents the null distribution. (PDF) [file pgen.1005875.s036.pdf]

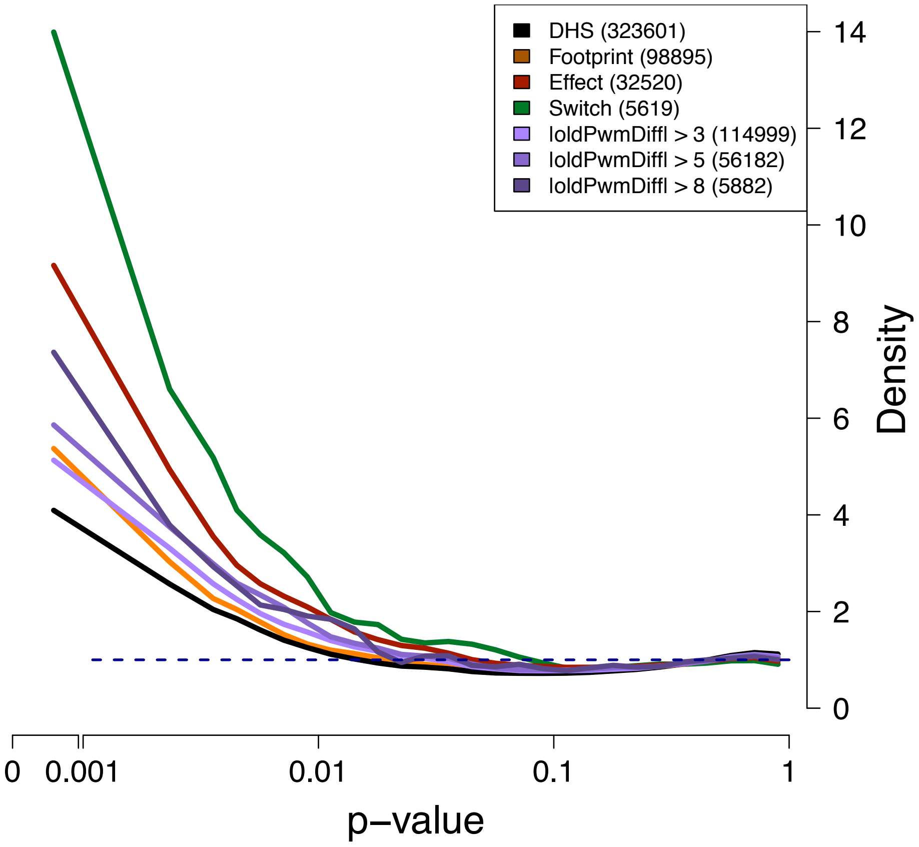

Figure S15: **Identification of ASH using only PWM score**. ASH p-value densities for different SNP categories and PWM (sequence match) match scores. Numbers in parentheses are the number of SNPs in those categories. The dotted blue line represents the null distribution.
