# Supplementary material for: Which Genetics Variants in DNase-Seq Footprints Are More Likely to Alter Binding?
Source: PLoS Genet. 2016 Feb 22;12(2):e1005875. doi: 10.1371/journal.pgen.1005875 (PMC4764260; doi:10.1371/journal.pgen.1005875)
Supplement: S16 Fig — ASH p-value densities for different SNP categories and SNPs with indicated phyloP conservation scores. Numbers in parentheses are the number of SNPs in those categories. The dotted blue line represents the null distribution. (PDF) [file pgen.1005875.s037.pdf]

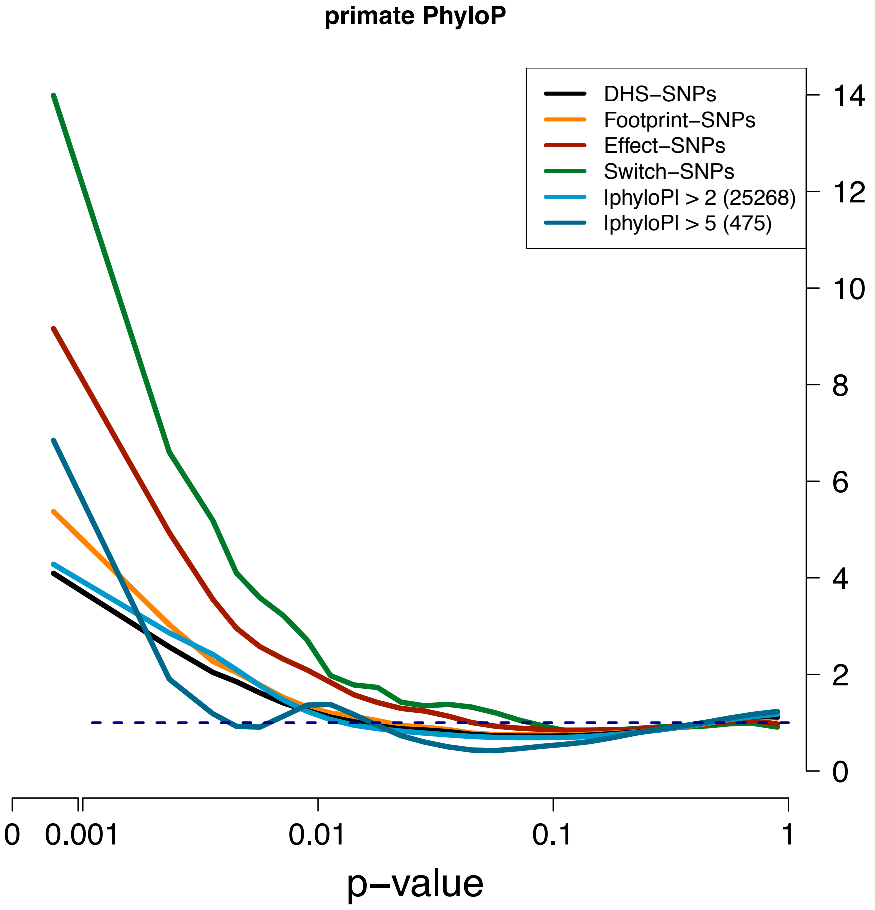

Figure S16: **Identification of ASH using phyloP conservation score**. ASH p-value densities for different SNP categories and SNPs with indicated phyloP conservation scores. Numbers in parentheses are the number of SNPs in those categories. The dotted blue line represents the null distribution.
