# Supplementary material for: Which Genetics Variants in DNase-Seq Footprints Are More Likely to Alter Binding?
Source: PLoS Genet. 2016 Feb 22;12(2):e1005875. doi: 10.1371/journal.pgen.1005875 (PMC4764260; doi:10.1371/journal.pgen.1005875)
Supplement: S17 Fig — For all motifs with > 100 hSNPs, an ASH enrichment ratio was calculated as # ASH-hSNPs (20% FDR) / # hSNPs across all binding sites genome-wide. The black line shows the average ratio across all motifs. Several factors whose binding sites are highly enriched or depleted for ASH-hSNPs are labeled. (PDF) [file pgen.1005875.s038.pdf]

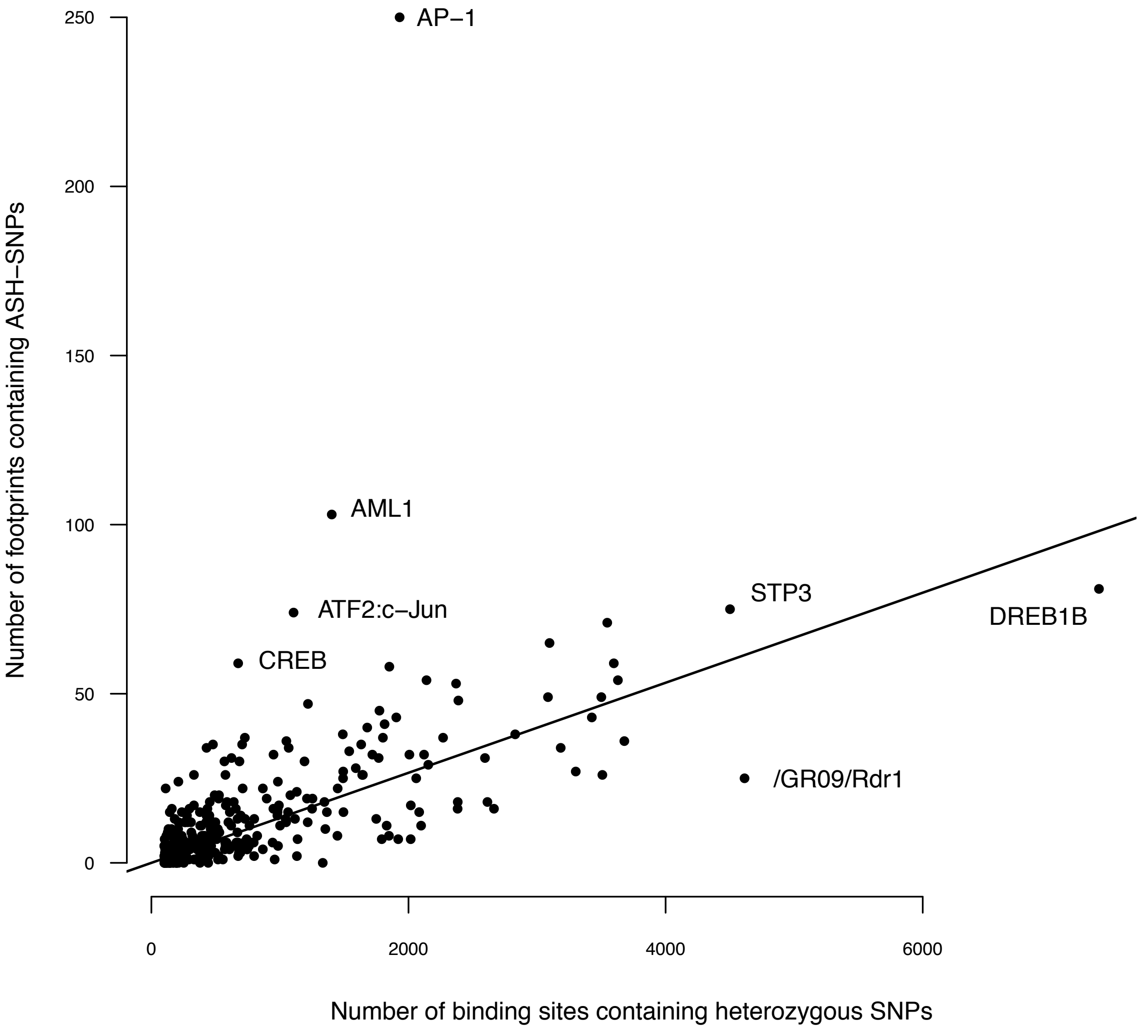

Figure S17: **Distribution of ASH enrichment ratios.** For all motifs with >100 hSNPs, an ASH enrichment ratio was calculated as # ASH-hSNPs (20% FDR) / # hSNPs across all binding sites genome-wide. The black line shows the average ratio across all motifs. Several factors whose binding sites are highly enriched or depleted for ASH-hSNPs are labeled.
