# Supplementary material for: Which Genetics Variants in DNase-Seq Footprints Are More Likely to Alter Binding?
Source: PLoS Genet. 2016 Feb 22;12(2):e1005875. doi: 10.1371/journal.pgen.1005875 (PMC4764260; doi:10.1371/journal.pgen.1005875)
Supplement: S18 Fig — (A) Density plot showing the distribution of selection scores from the modified MK test. (B) Comparison of selection scores to the number of tissues each factor is predicted to be active in. (C) Comparison of selection scores to the median distance to the TSS across all sites for a given factor. (PDF) [file pgen.1005875.s039.pdf]

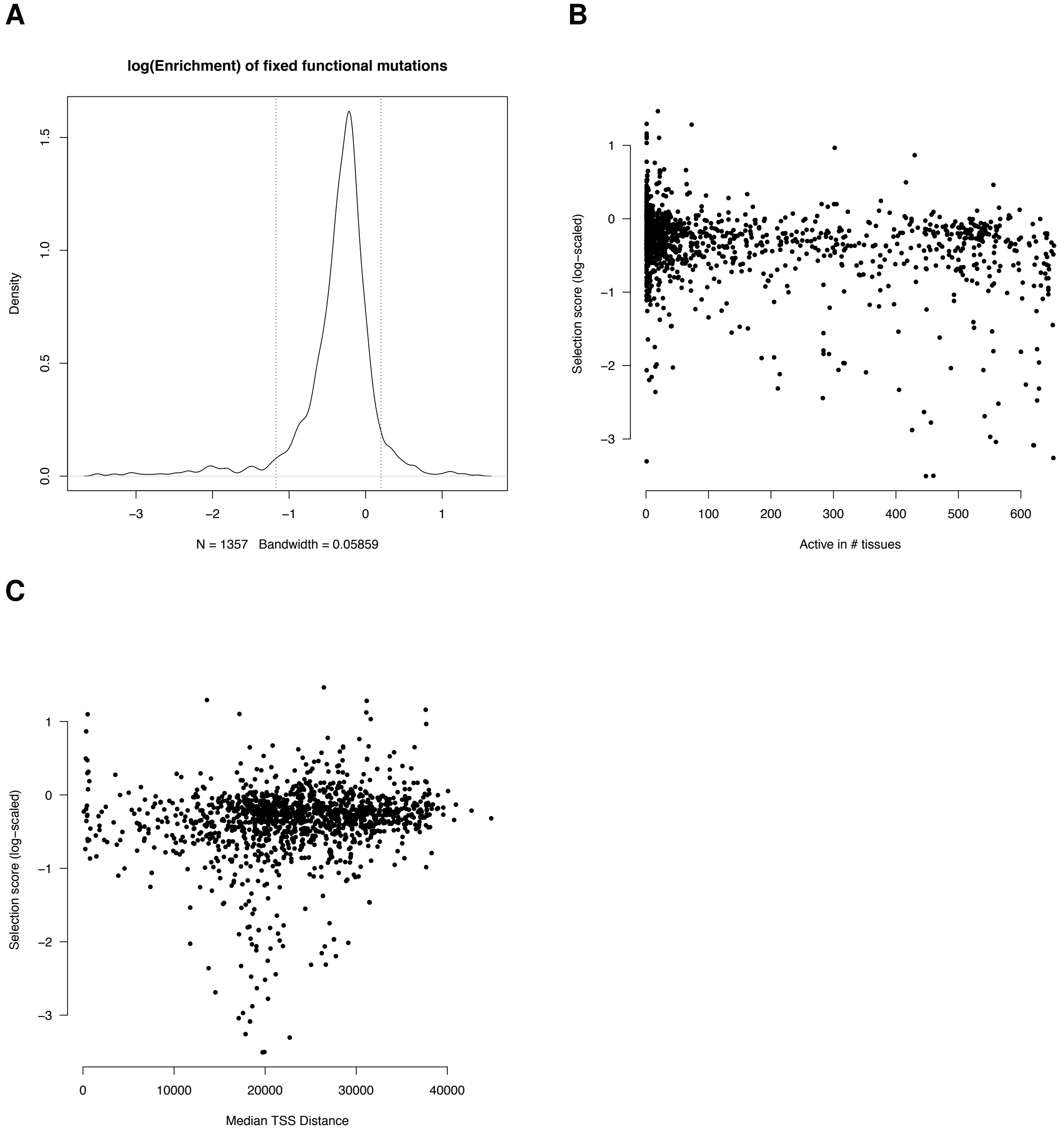


Figure S18: **Identifying selection signals in TF binding sites**. (A) Density plot showing the distribution of selection scores from the modified MK test. (B) Comparison of selection scores to the number of tissues each factor is predicted to be active in. (C) Comparison of selection scores to the median distance to the TSS across all sites for a given factor.
