# Supplementary material for: Which Genetics Variants in DNase-Seq Footprints Are More Likely to Alter Binding?
Source: PLoS Genet. 2016 Feb 22;12(2):e1005875. doi: 10.1371/journal.pgen.1005875 (PMC4764260; doi:10.1371/journal.pgen.1005875)
Supplement: S19 Fig — Shown are the relative enrichments for each DAF/selection score bin, for all variants (A) and for singletons and doubletons (B). (PDF) [file pgen.1005875.s040.pdf]

A

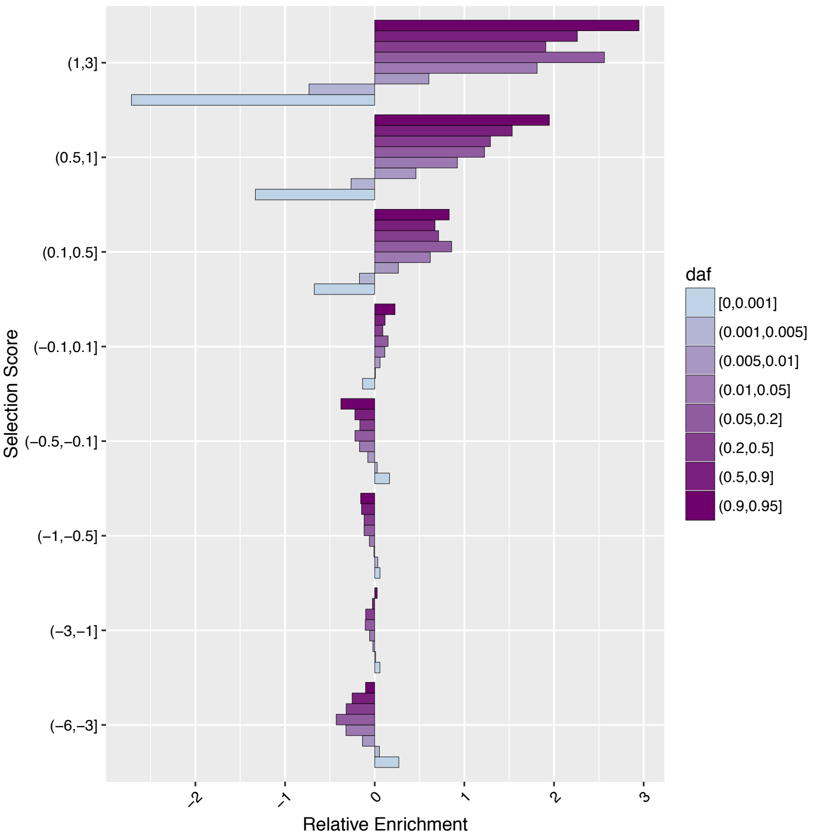

B

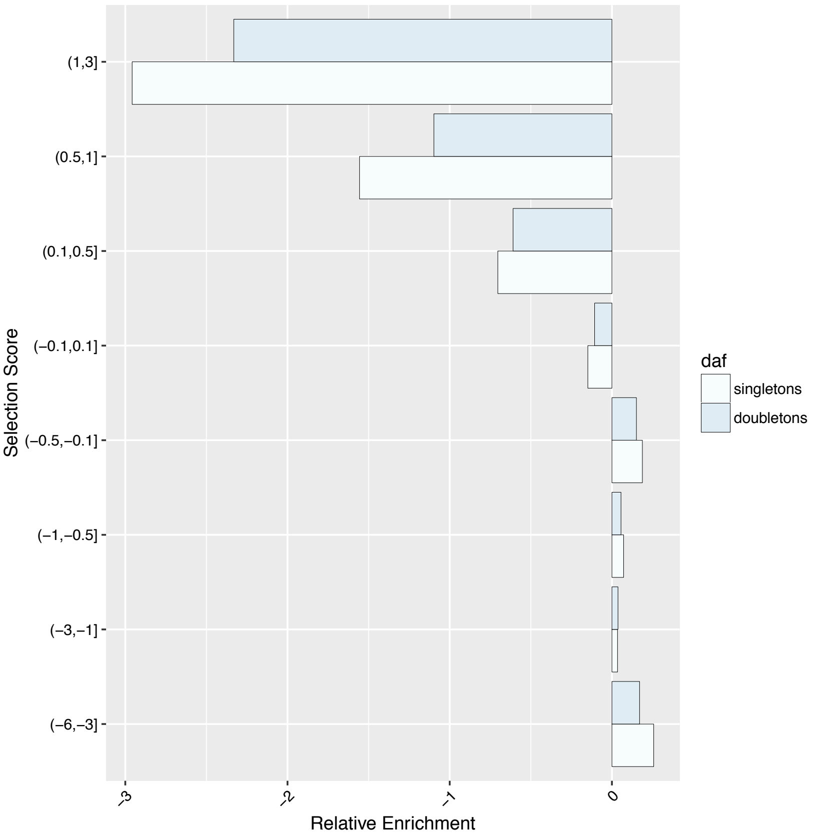

Figure S19: **Derived allele frequency and selection score**. Shown are the relative enrichments for each DAF/selection score bin, for all variants (A) and for singletons and doubletons (B).
