# Supplementary material for: Which Genetics Variants in DNase-Seq Footprints Are More Likely to Alter Binding?
Source: PLoS Genet. 2016 Feb 22;12(2):e1005875. doi: 10.1371/journal.pgen.1005875 (PMC4764260; doi:10.1371/journal.pgen.1005875)
Supplement: S20 Fig — Shown are the log2(enrichment) values with 95% confidence intervals for each factor whose binding sites are enriched for SNPs associated with the traits in S14 Table. x-axis is truncated at 10 for ease of display. (PDF) [file pgen.1005875.s041.pdf]

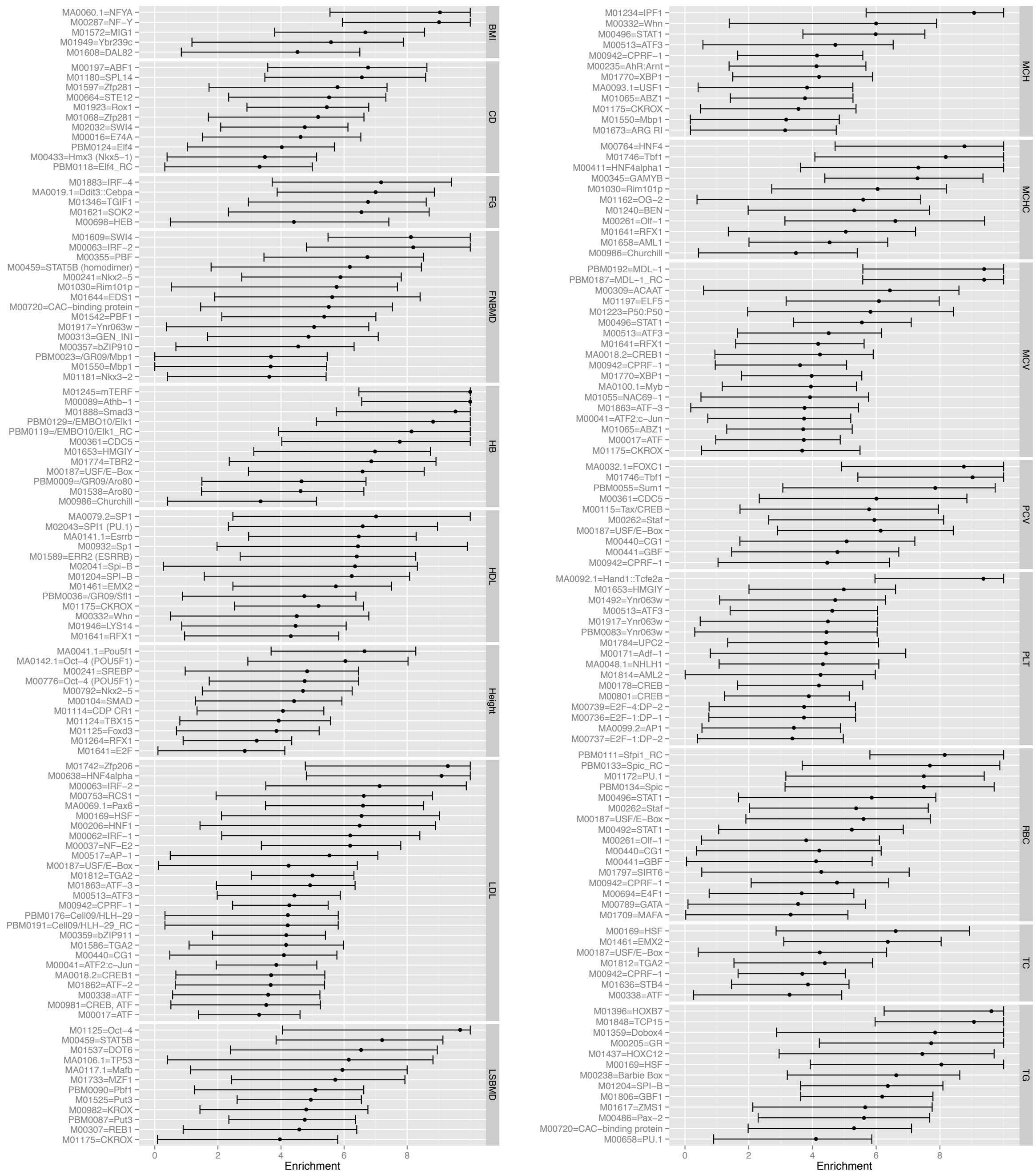

Figure S20: **Enrichment of transcription factors motifs from fgwas**. Shown are the log_2(enrichment) values with 95% confidence intervals for each factor whose binding sites are enriched for SNPs associated with the traits in Table 14. x-axis is truncated at 10 for ease of display.
