# Supplementary material for: Which Genetics Variants in DNase-Seq Footprints Are More Likely to Alter Binding?
Source: PLoS Genet. 2016 Feb 22;12(2):e1005875. doi: 10.1371/journal.pgen.1005875 (PMC4764260; doi:10.1371/journal.pgen.1005875)
Supplement: S21 Fig — Log Bayes factor (top) and posterior probabilities (bottom) of association to the indicated trait for all genetic variants in the regions containing rs4519508 and rs532436. (PDF) [file pgen.1005875.s042.pdf]

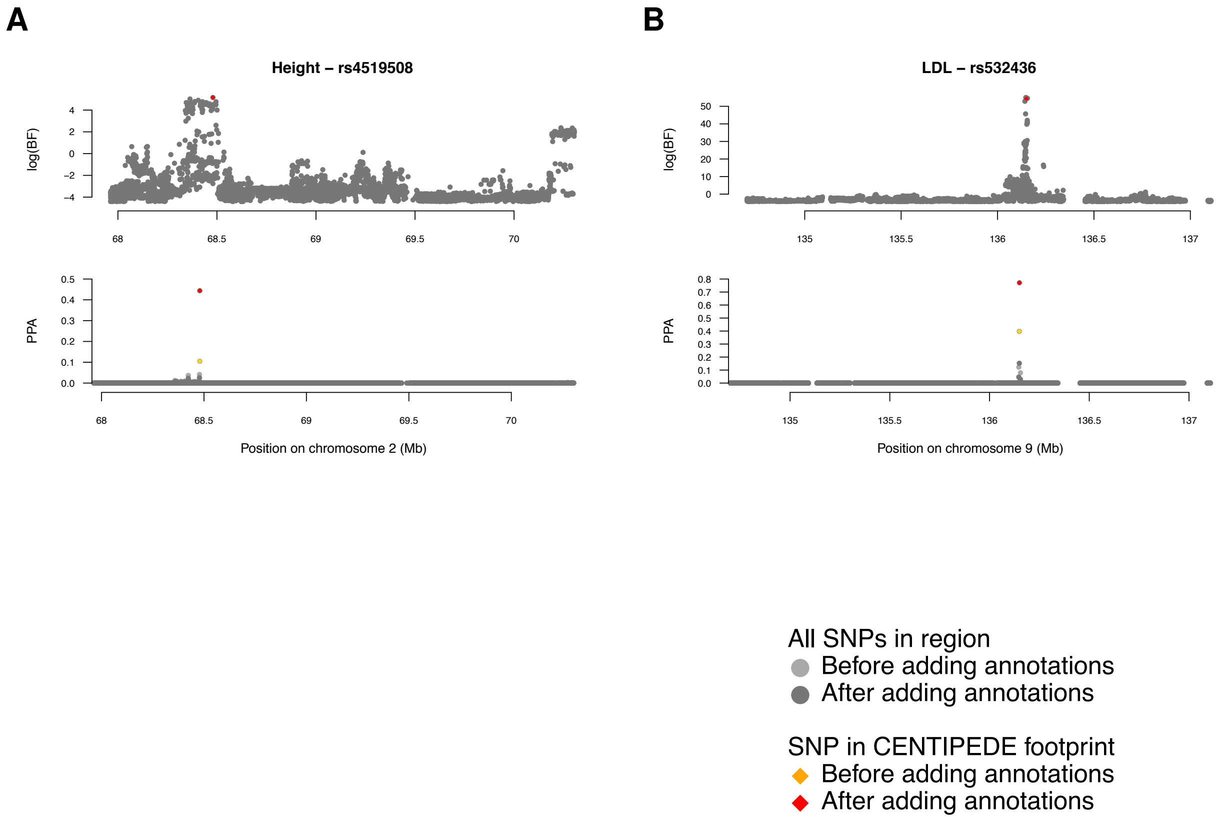

Figure S21: **Association plots identifying SNPs in footprints.** Log Bayes factor (top) and posterior probabilities (bottom) of association to the indicated trait for all genetic variants in the regions containing rs4519508 and rs532436.
