# Supplementary material for: Which Genetics Variants in DNase-Seq Footprints Are More Likely to Alter Binding?
Source: PLoS Genet. 2016 Feb 22;12(2):e1005875. doi: 10.1371/journal.pgen.1005875 (PMC4764260; doi:10.1371/journal.pgen.1005875)
Supplement: S22 Fig — For each point, plotted is the difference in the change in probability of binding (mutated prior log ratio—reference prior log ratio, x-axis) versus the log2(fold change) between mutated and wild type reporter constructs (y-axis). The black line represents the best-fit line from a linear model fit on all 22 points. (PDF) [file pgen.1005875.s043.pdf]

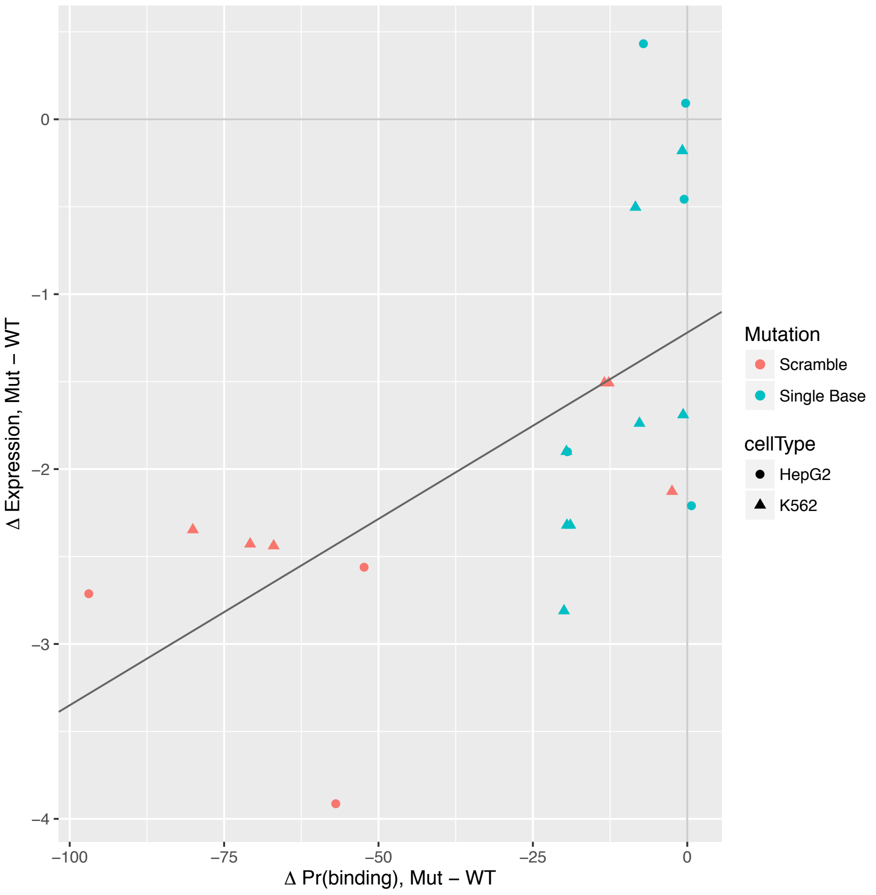

Figure S22: **Correlation of CENTIPEDE predictions and mutated enhancers in HepG2 and K562 cells.** For each point, plotted is the difference in the change in probability of binding (mutated prior log ratio - reference prior log ratio, x-axis) versus the log_2(fold change) between mutated and wild type reporter constructs expression (y-axis). The black line represents the best-fit line from a linear model fit on all 22 points.
