# Supplementary material for: Which Genetics Variants in DNase-Seq Footprints Are More Likely to Alter Binding?
Source: PLoS Genet. 2016 Feb 22;12(2):e1005875. doi: 10.1371/journal.pgen.1005875 (PMC4764260; doi:10.1371/journal.pgen.1005875)
Supplement: S23 Fig — For each SNP in S13 Table, two plots show the log Bayes factor (top) and posterior probabilities (bottom) of association to the indicated trait for all genetic variants in the region containing the SNP. (PDF) [file pgen.1005875.s044.pdf]

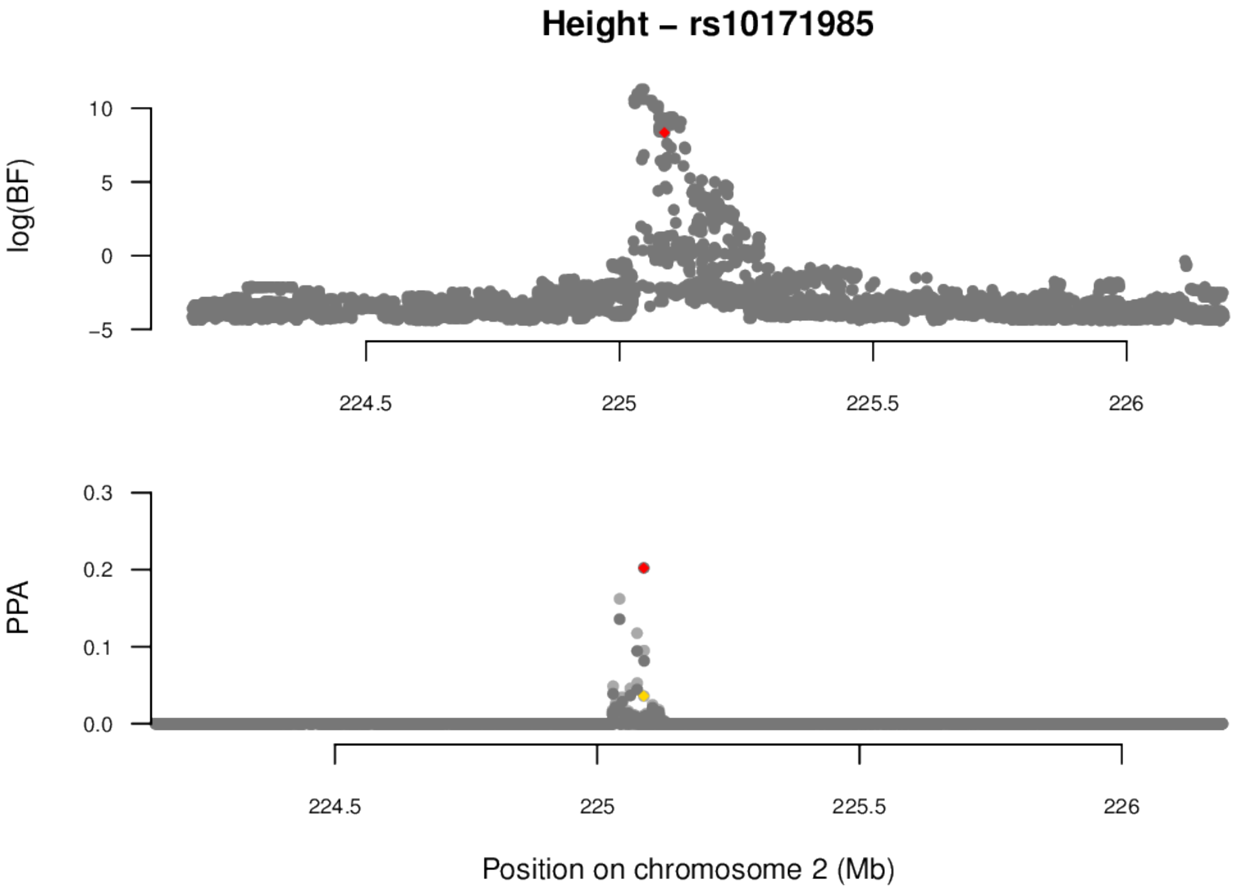

Height – rs10171985
log(BF)
10
5
0
−5
224.5
225
225.5
226
PPA
0.3
0.2
0.1
0.0
224.5
225
225.5
226
Position on chromosome 2 (Mb)

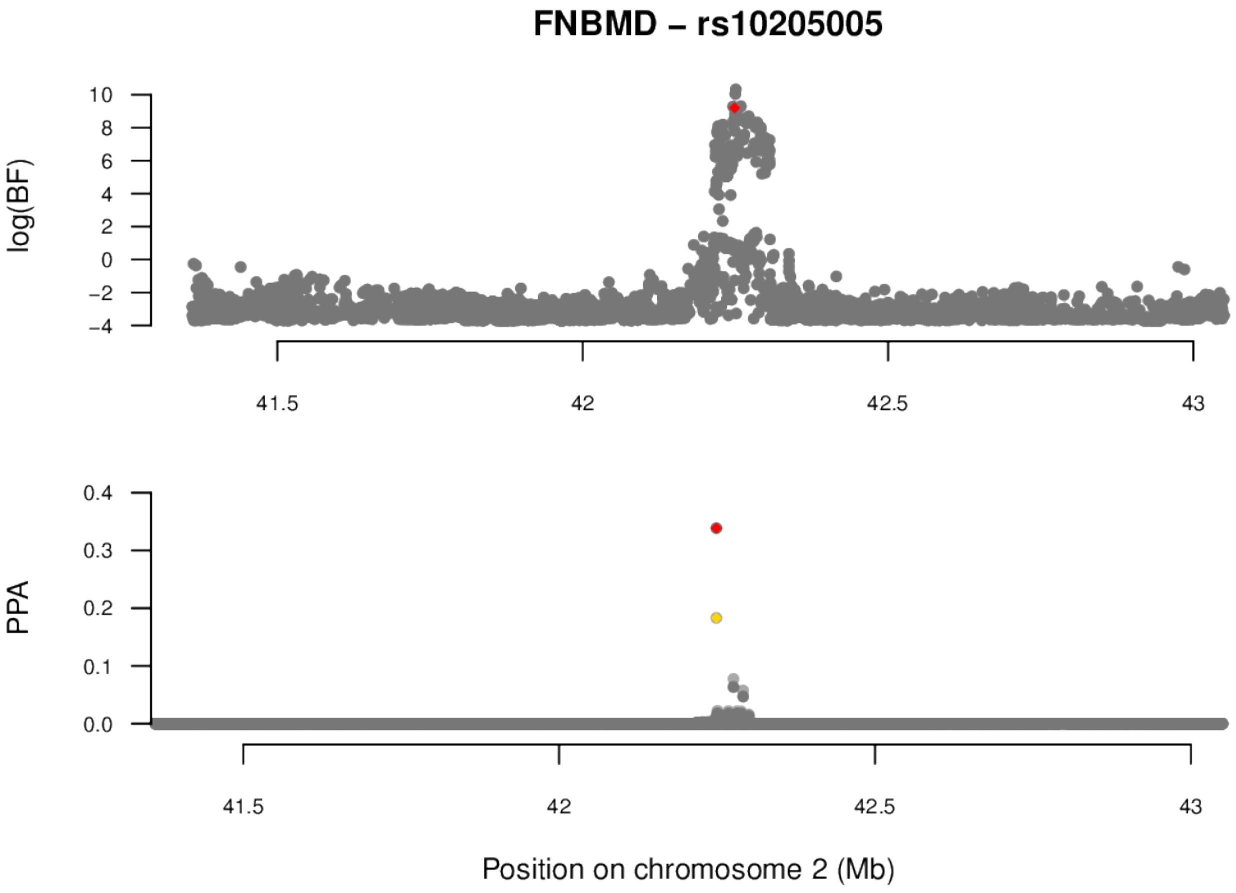
FNBMD – rs10205005
log(BF)
10
8
6
4
2
0
−2
−4
41.5
42
42.5
43
PPA
0.4
0.3
0.2
0.1
0.0
41.5
42
42.5
43
Position on chromosome 2 (Mb)

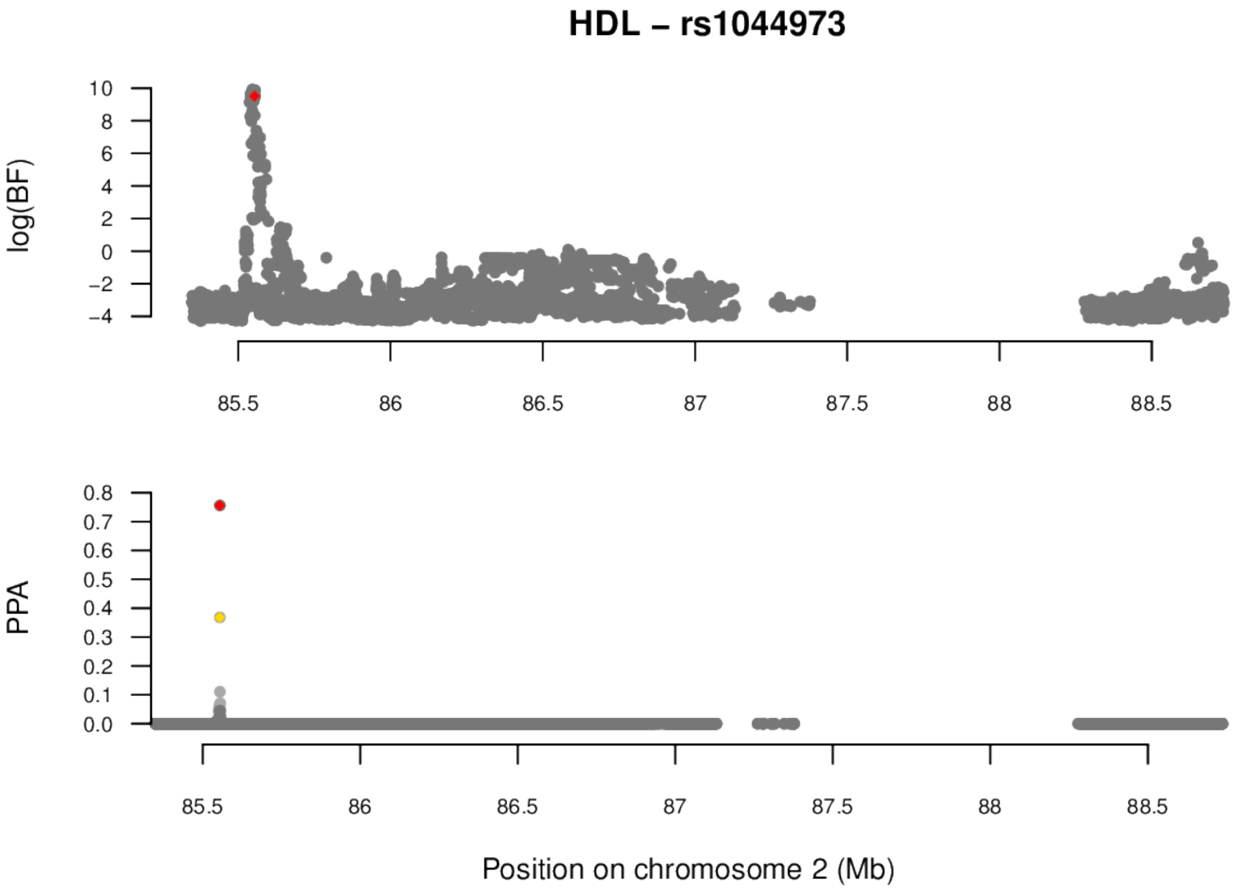
HDL – rs1044973
log(BF)
10
8
6
4
2
0
−2
−4
85.5
86
86.5
87
87.5
88
88.5
PPA
0.8
0.7
0.6
0.5
0.4
0.3
0.2
0.1
0.0
85.5
86
86.5
87
87.5
88
88.5
Position on chromosome 2 (Mb)

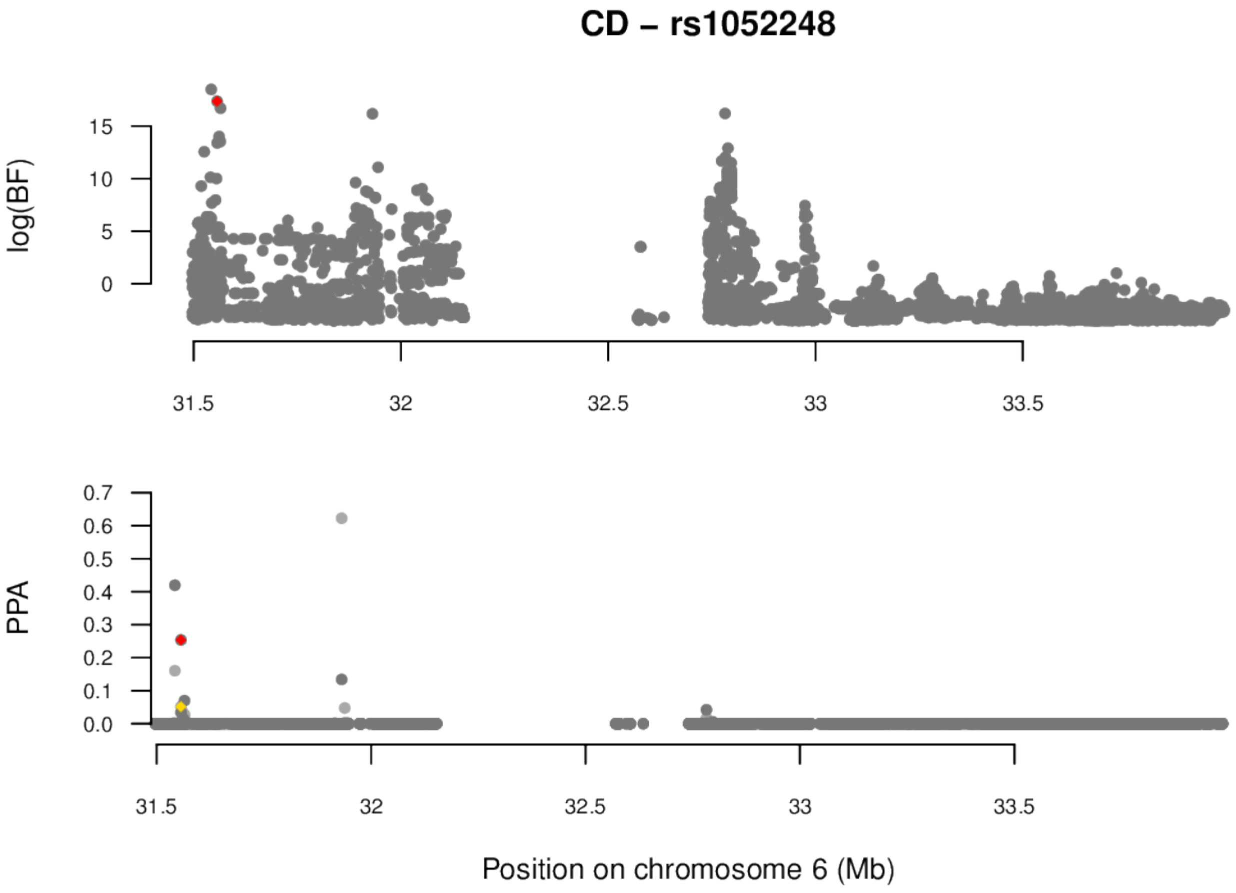
CD – rs1052248
log(BF)
15
10
5
0
31.5
32
32.5
33
33.5
PPA
0.7
0.6
0.5
0.4
0.3
0.2
0.1
0.0
31.5
32
32.5
33
33.5
Position on chromosome 6 (Mb)

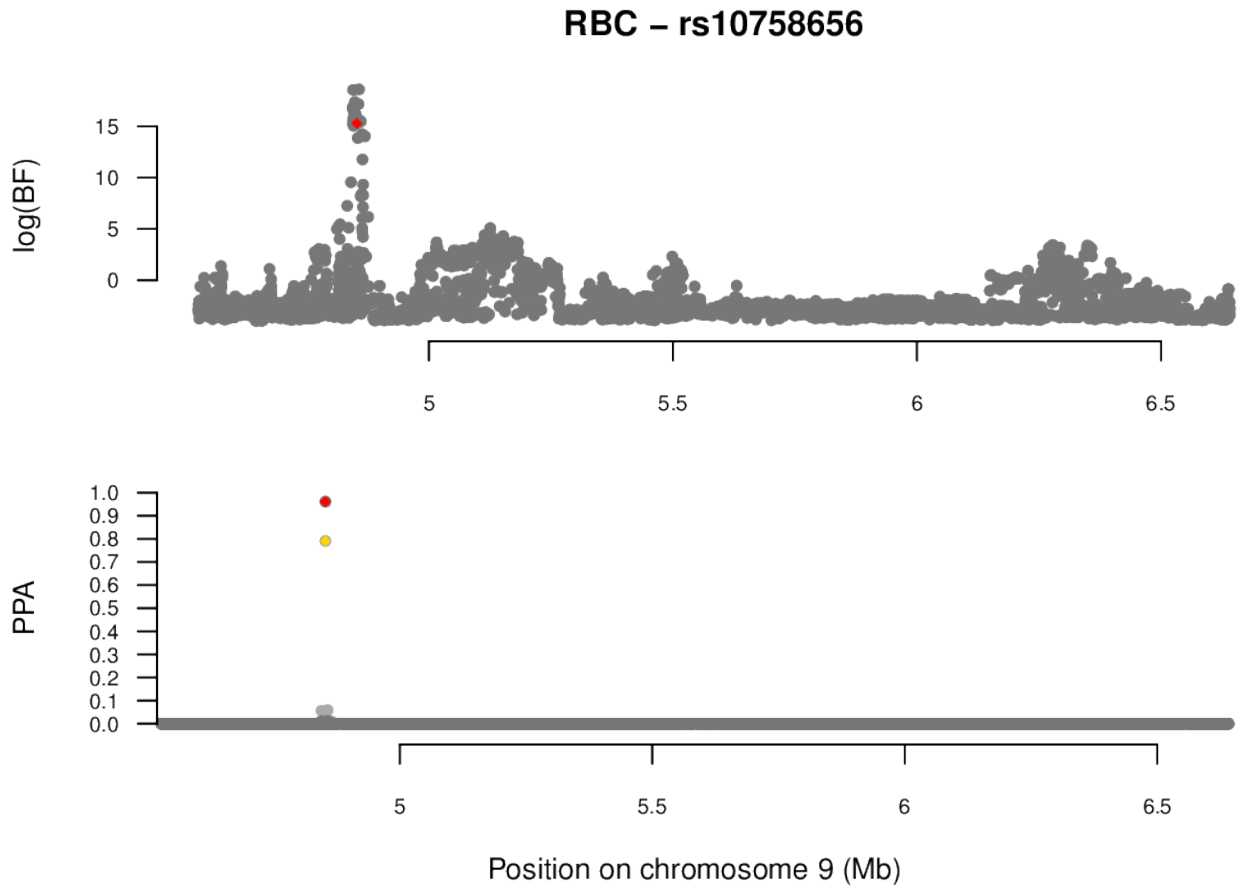
RBC – rs10758656
log(BF)
15
10
5
0
5
5.5
6
6.5
PPA
1.0
0.9
0.8
0.7
0.6
0.5
0.4
0.3
0.2
0.1
0.0
5
5.5
6
6.5
Position on chromosome 9 (Mb)

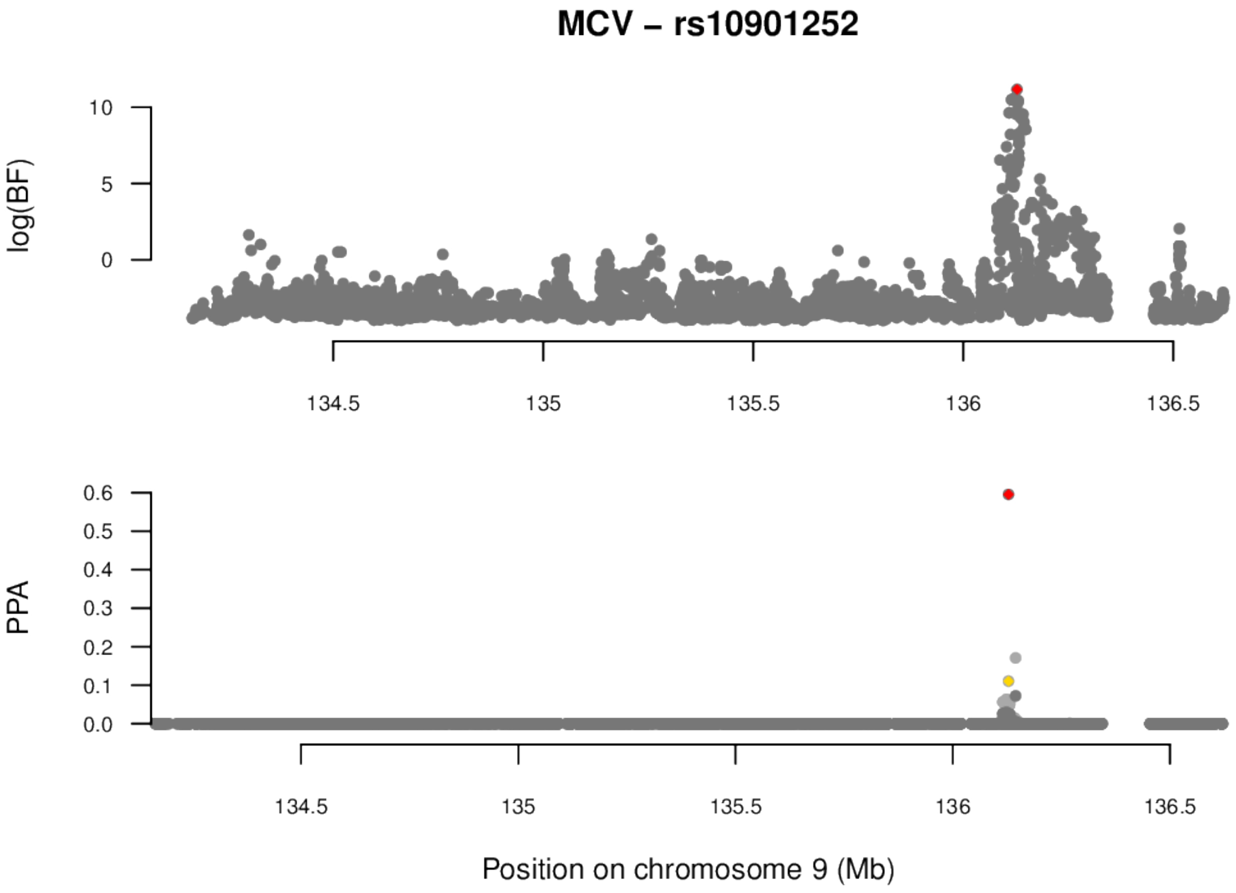

MCV – rs10901252
log(BF)
10
5
0
134.5
135
135.5
136
136.5
PPA
0.6
0.5
0.4
0.3
0.2
0.1
0.0
134.5
135
135.5
136
136.5
Position on chromosome 9 (Mb)

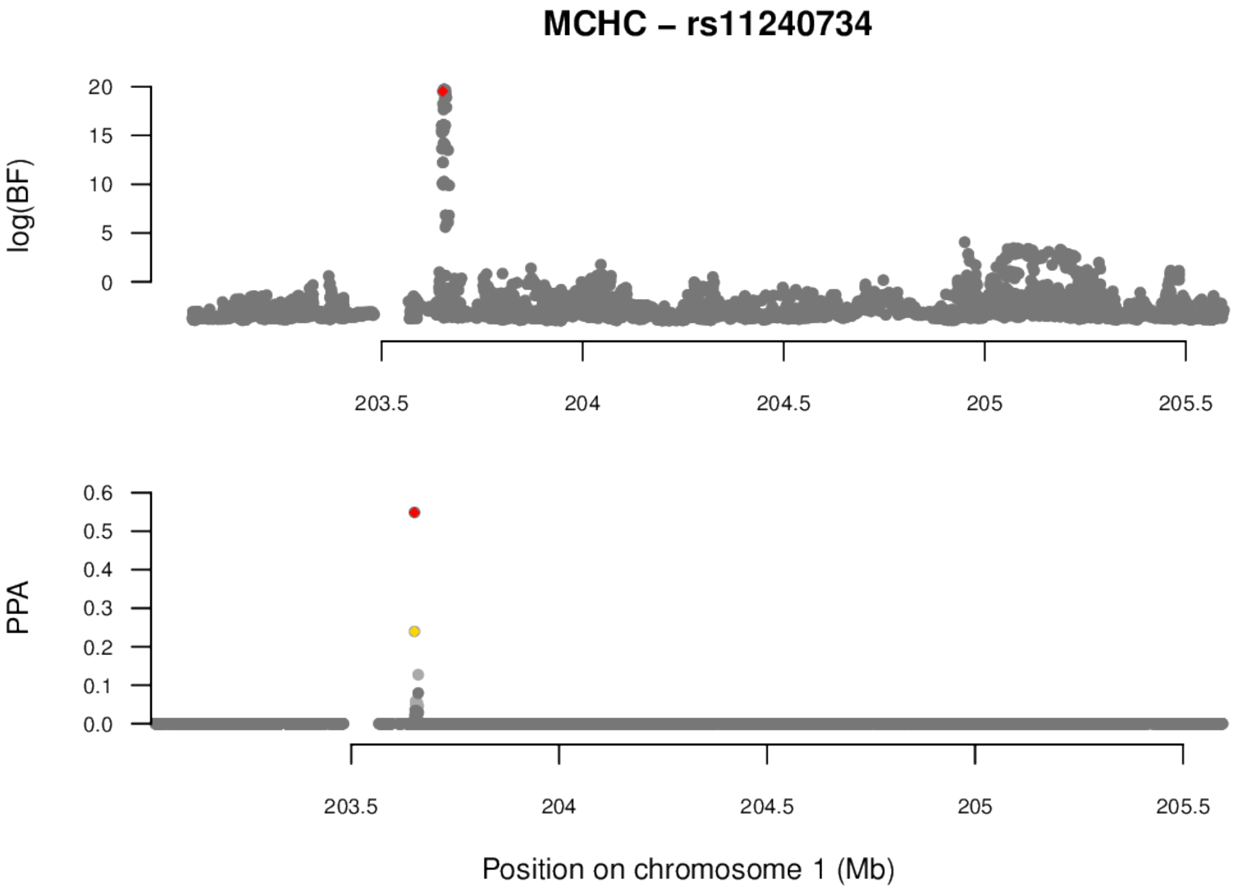
MCHC – rs11240734
log(BF)
20
15
10
5
0
203.5
204
204.5
205
205.5
PPA
0.6
0.5
0.4
0.3
0.2
0.1
0.0
203.5
204
204.5
205
205.5
Position on chromosome 1 (Mb)

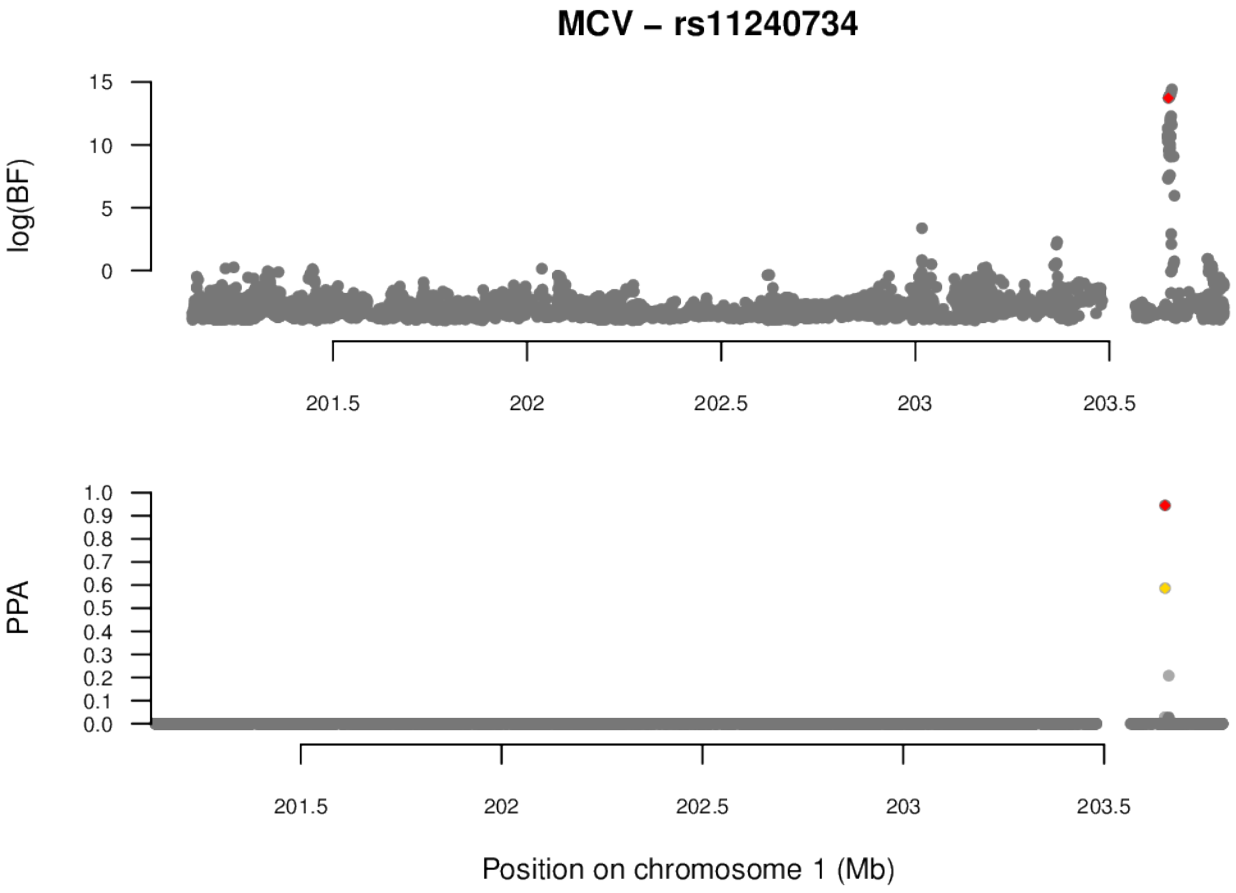

MCV – rs11240734
log(BF)
15
10
5
0
201.5
202
202.5
203
203.5
PPA
1.0
0.9
0.8
0.7
0.6
0.5
0.4
0.3
0.2
0.1
0.0
201.5
202
202.5
203
203.5
Position on chromosome 1 (Mb)

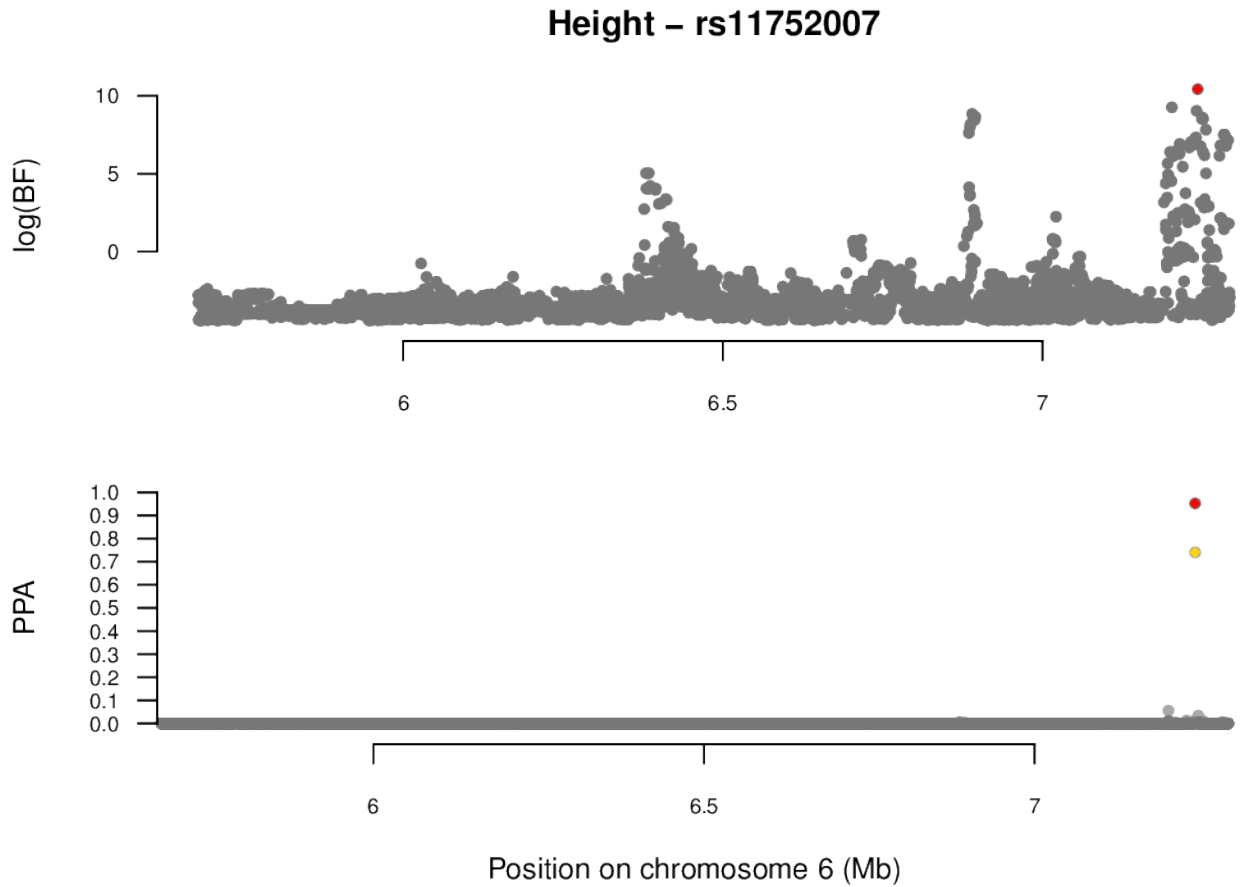

Height – rs11752007
log(BF)
10
5
0
6
6.5
7
PPA
1.0
0.9
0.8
0.7
0.6
0.5
0.4
0.3
0.2
0.1
0.0
6
6.5
7
Position on chromosome 6 (Mb)

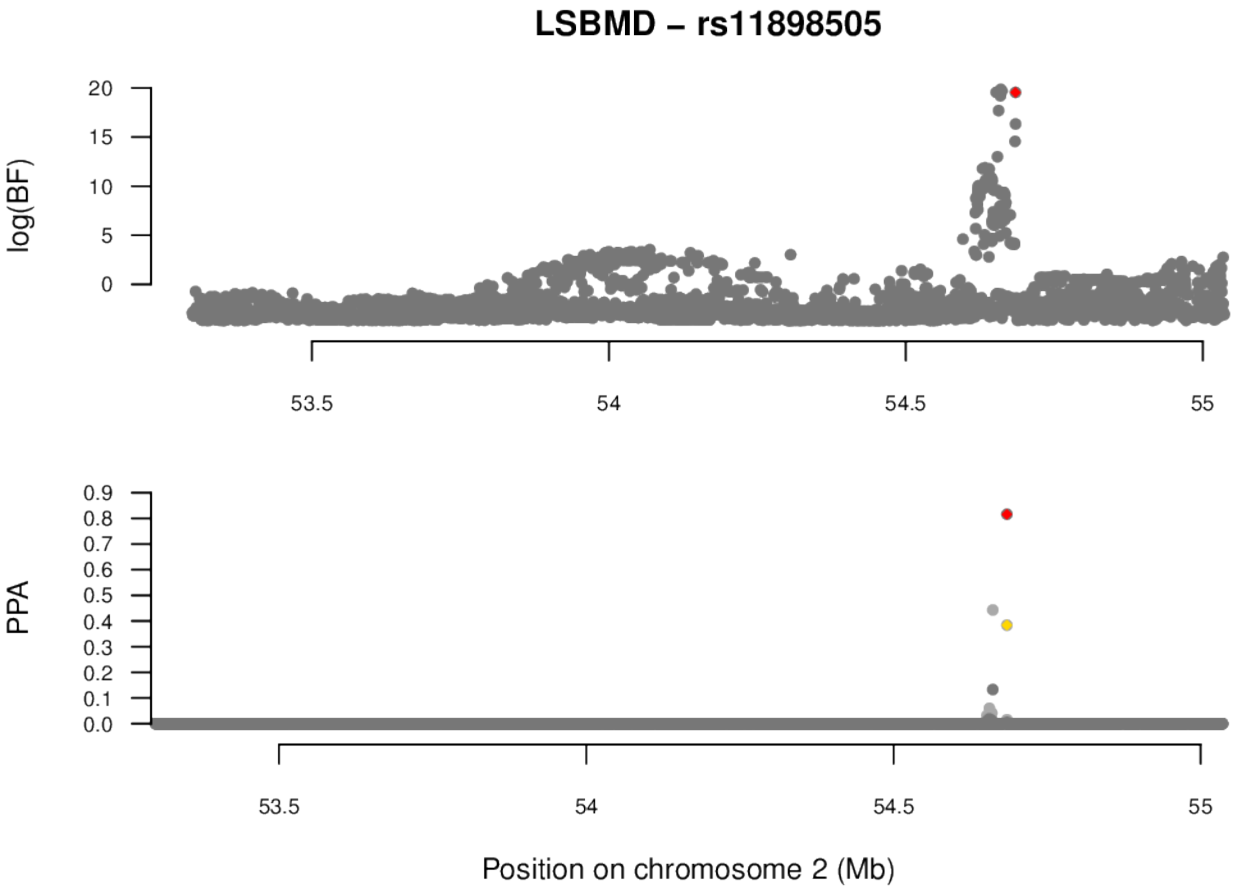
LSBMD – rs11898505
log(BF)
20
15
10
5
0
53.5
54
54.5
55
PPA
0.9
0.8
0.7
0.6
0.5
0.4
0.3
0.2
0.1
0.0
53.5
54
54.5
55
Position on chromosome 2 (Mb)

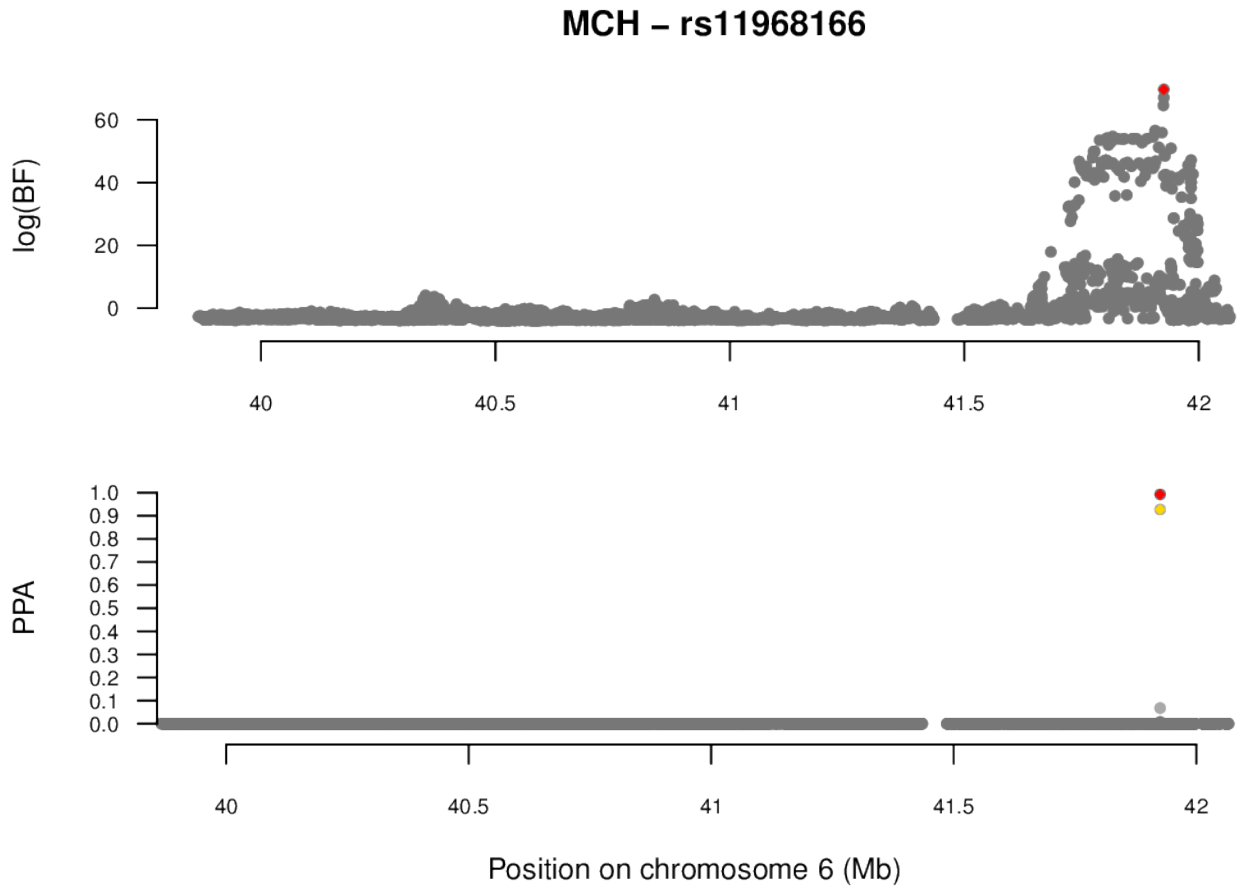
MCH – rs11968166
log(BF)
60
40
20
0
40
40.5
41
41.5
42
PPA
1.0
0.9
0.8
0.7
0.6
0.5
0.4
0.3
0.2
0.1
0.0
40
40.5
41
41.5
42
Position on chromosome 6 (Mb)

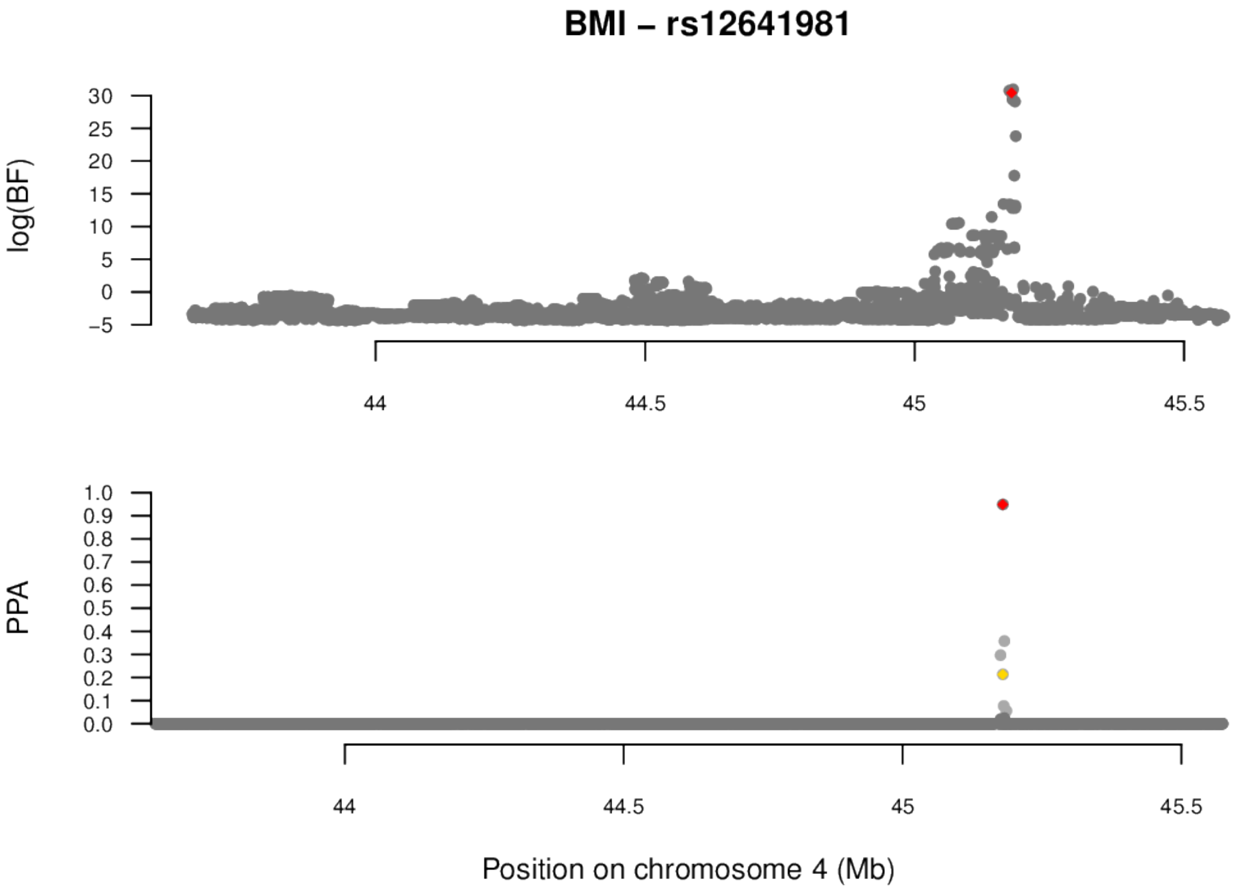

BMI – rs12641981
log(BF)
30
25
20
15
10
5
0
−5
44
44.5
45
45.5
PPA
1.0
0.9
0.8
0.7
0.6
0.5
0.4
0.3
0.2
0.1
0.0
44
44.5
45
45.5
Position on chromosome 4 (Mb)

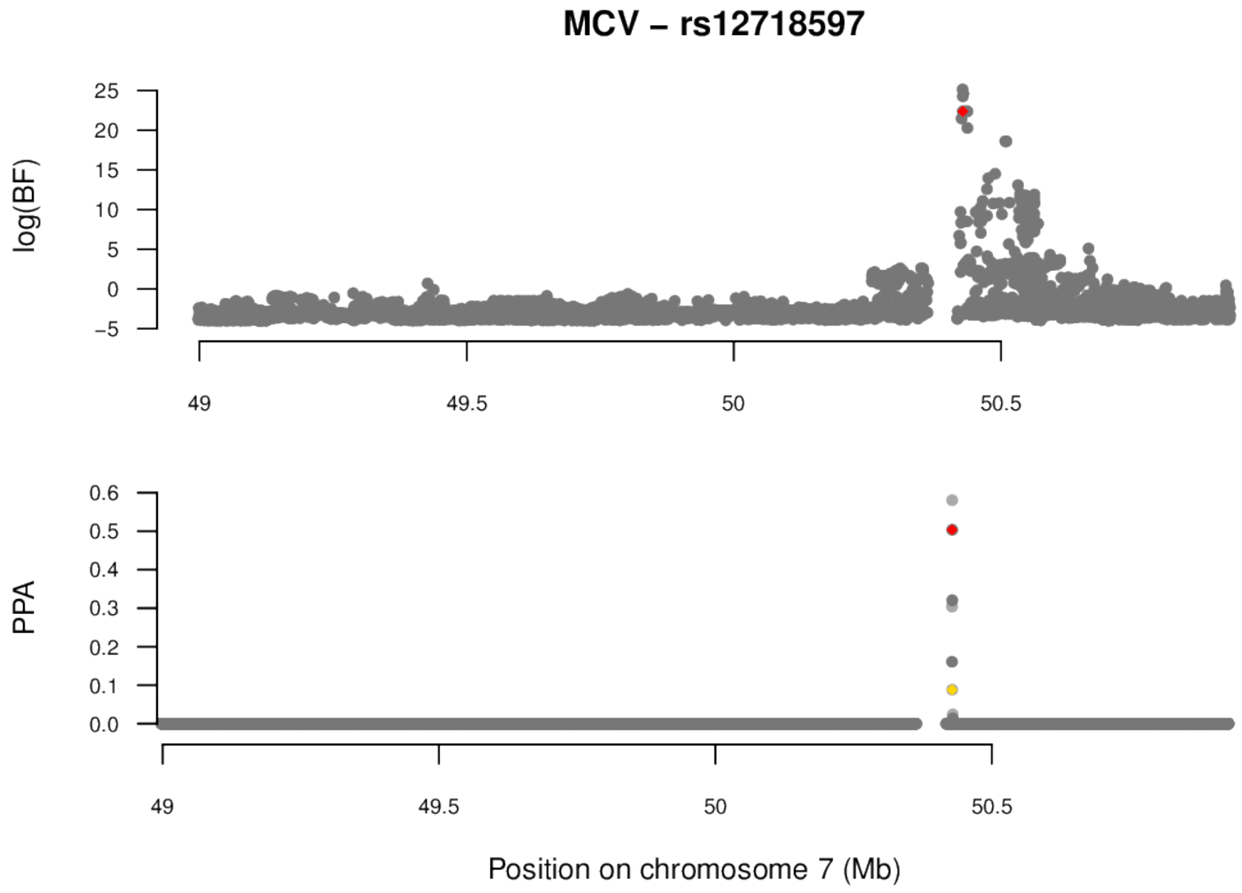
MCV – rs12718597
log(BF)
25
20
15
10
5
0
−5
49
49.5
50
50.5
PPA
0.6
0.5
0.4
0.3
0.2
0.1
0.0
49
49.5
50
50.5
Position on chromosome 7 (Mb)

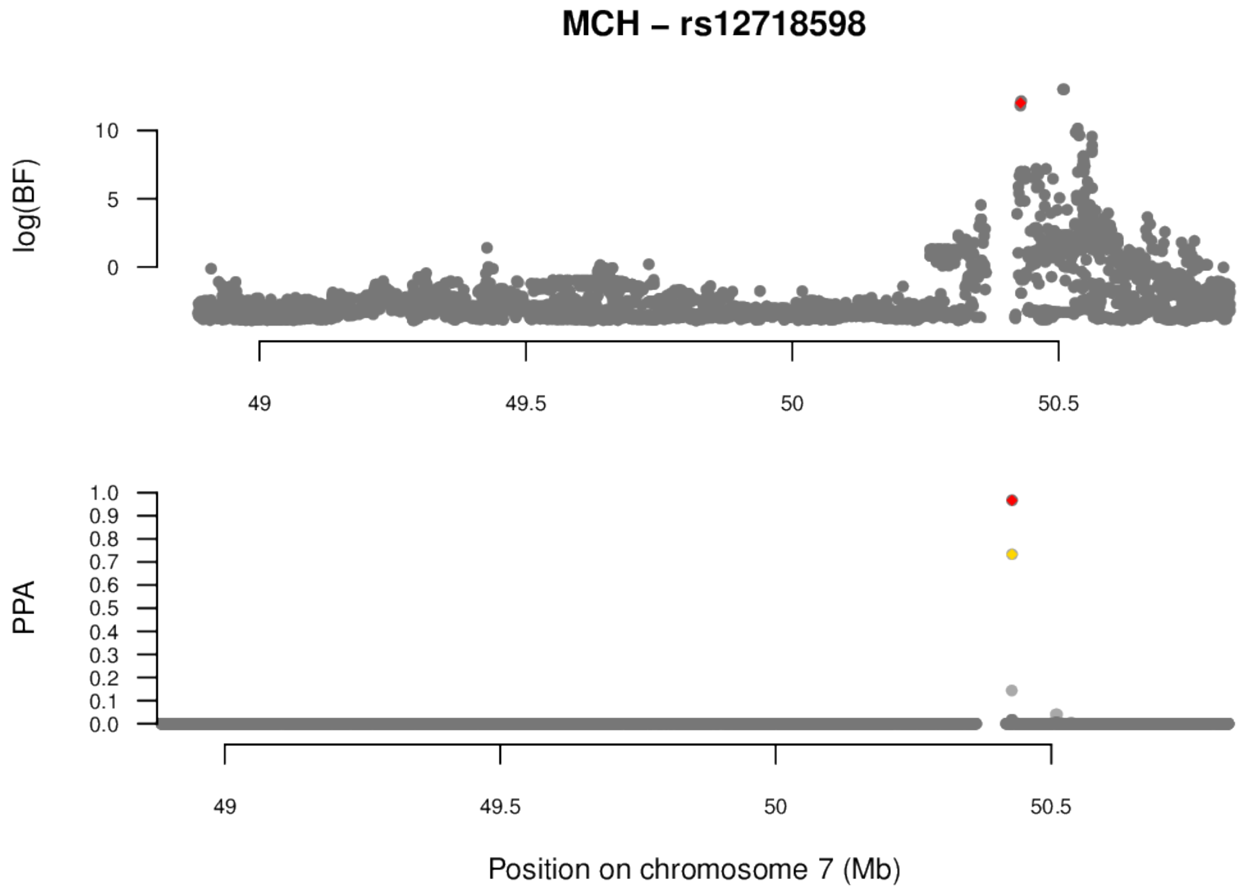

MCH – rs12718598
log(BF)
10
5
0
49
49.5
50
50.5
PPA
1.0
0.9
0.8
0.7
0.6
0.5
0.4
0.3
0.2
0.1
0.0
49
49.5
50
50.5
Position on chromosome 7 (Mb)

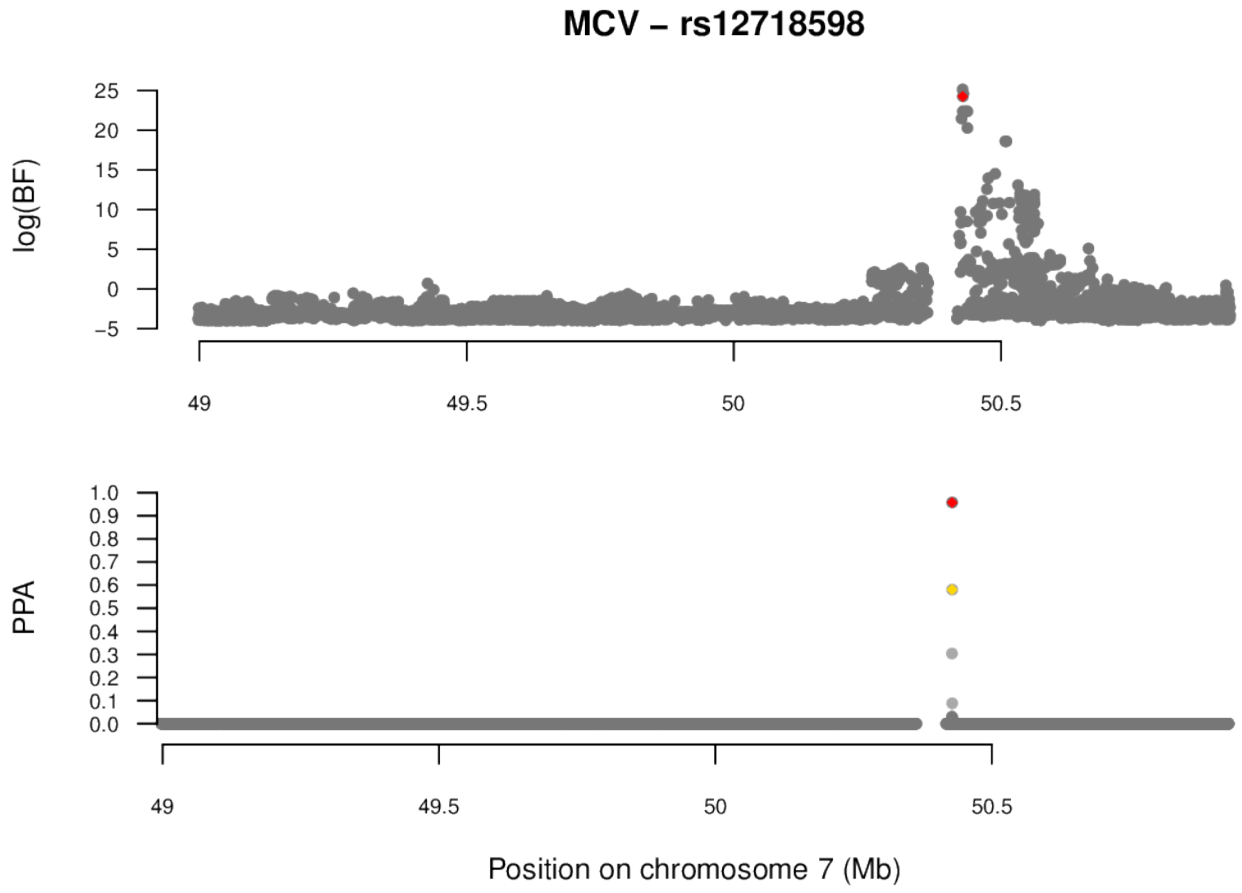

MCV – rs12718598
log(BF)
25
20
15
10
5
0
−5
49
49.5
50
50.5
PPA
1.0
0.9
0.8
0.7
0.6
0.5
0.4
0.3
0.2
0.1
0.0
49
49.5
50
50.5
Position on chromosome 7 (Mb)

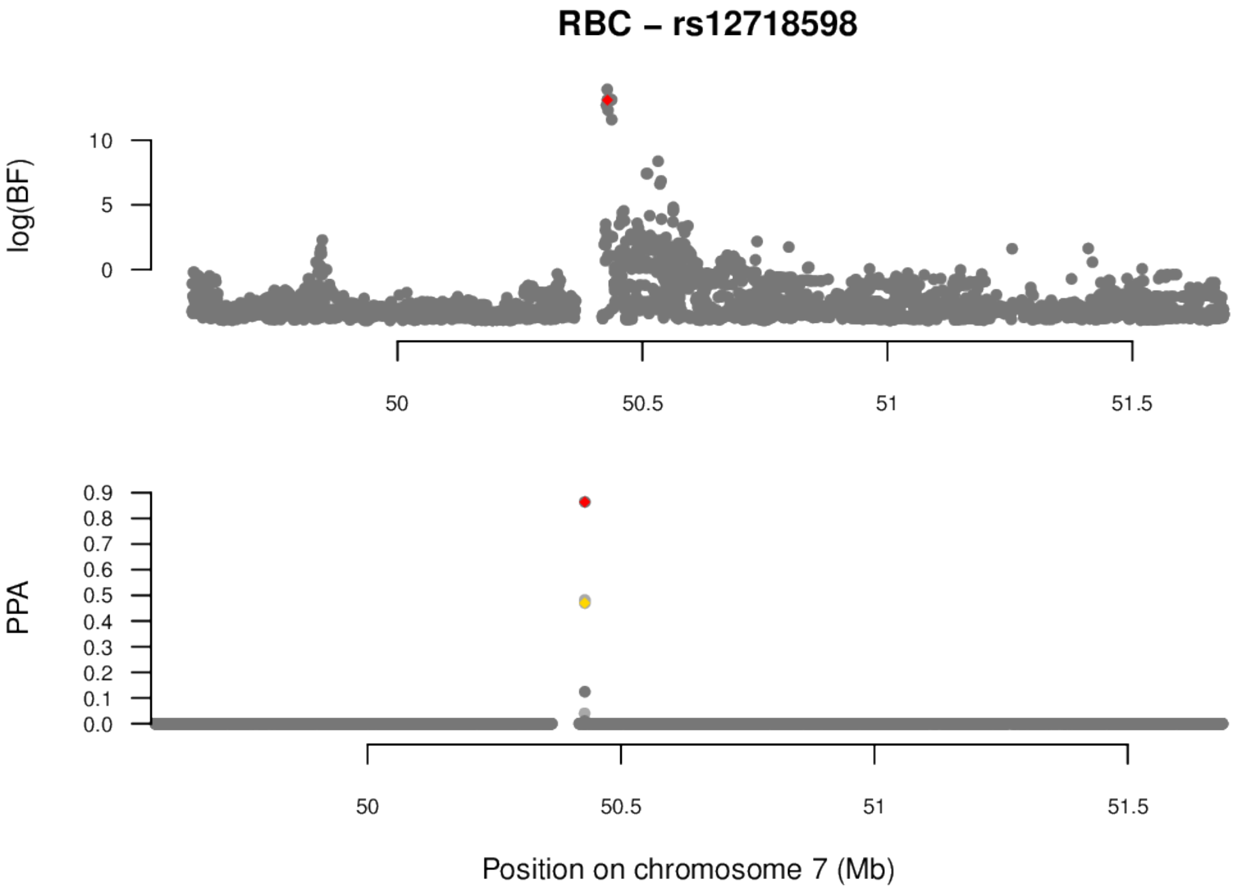

RBC – rs12718598
log(BF)
10
5
0
50
50.5
51
51.5
PPA
0.9
0.8
0.7
0.6
0.5
0.4
0.3
0.2
0.1
0.0
50
50.5
51
51.5
Position on chromosome 7 (Mb)

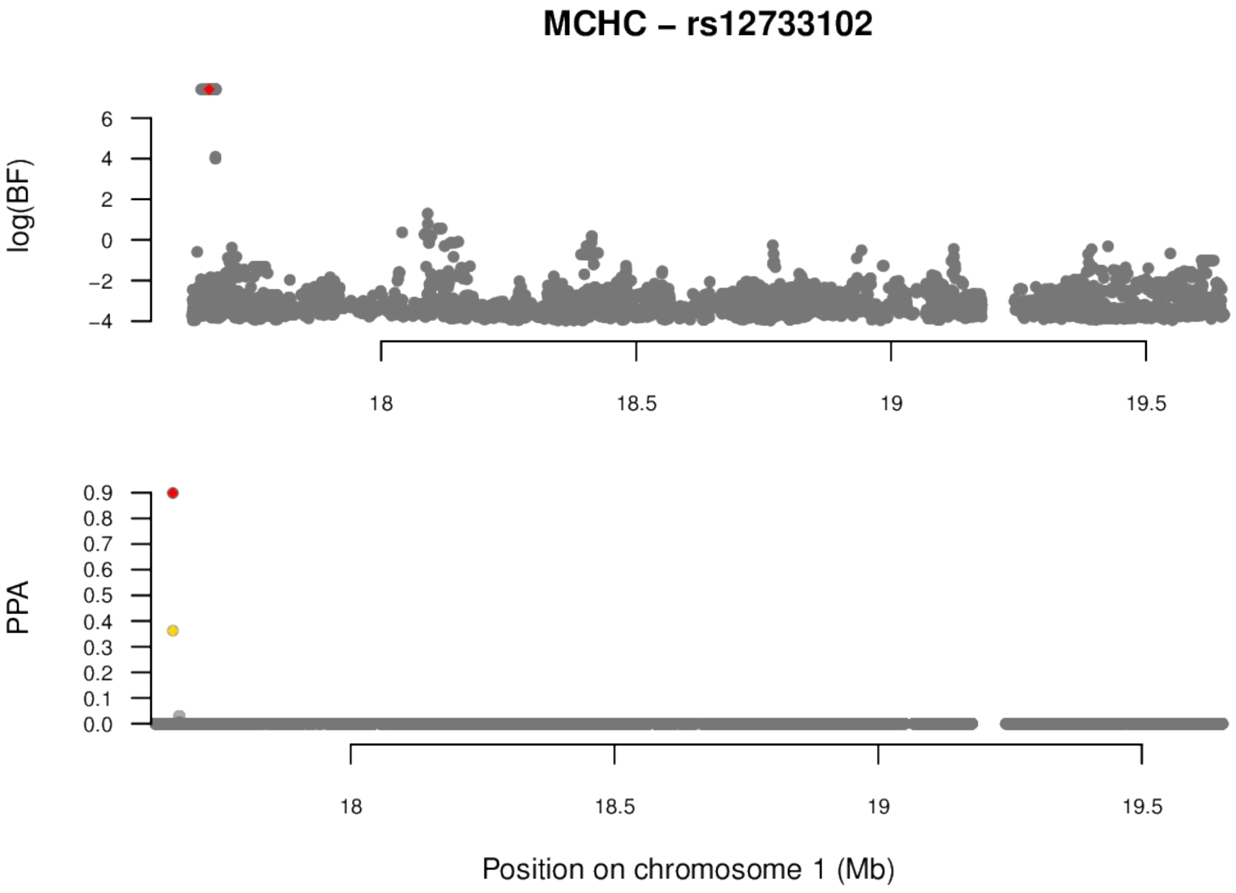

MCHC – rs12733102
log(BF)
6
4
2
0
−2
−4
18
18.5
19
19.5
PPA
0.9
0.8
0.7
0.6
0.5
0.4
0.3
0.2
0.1
0.0
18
18.5
19
19.5
Position on chromosome 1 (Mb)

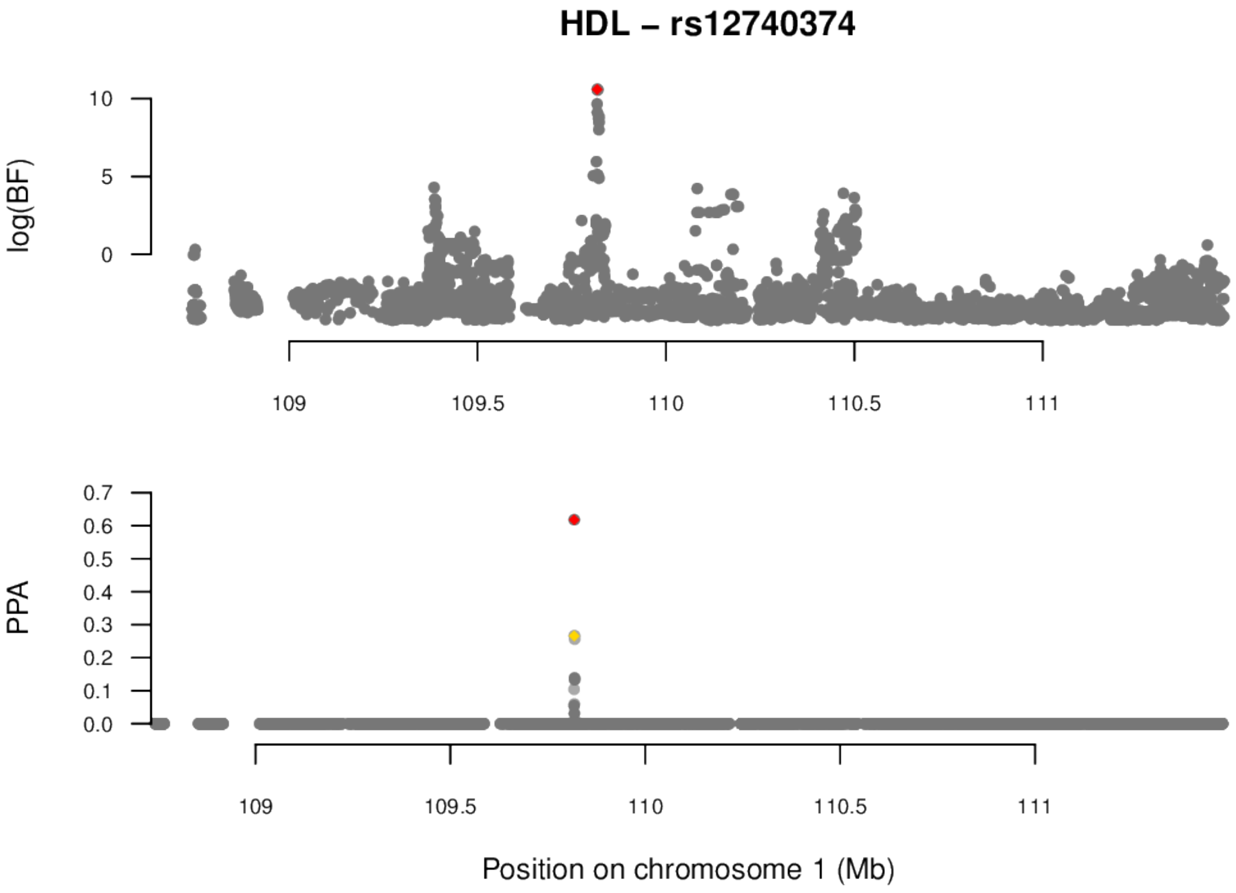
HDL – rs12740374
log(BF)
10
5
0
109
109.5
110
110.5
111
PPA
0.7
0.6
0.5
0.4
0.3
0.2
0.1
0.0
109
109.5
110
110.5
111
Position on chromosome 1 (Mb)

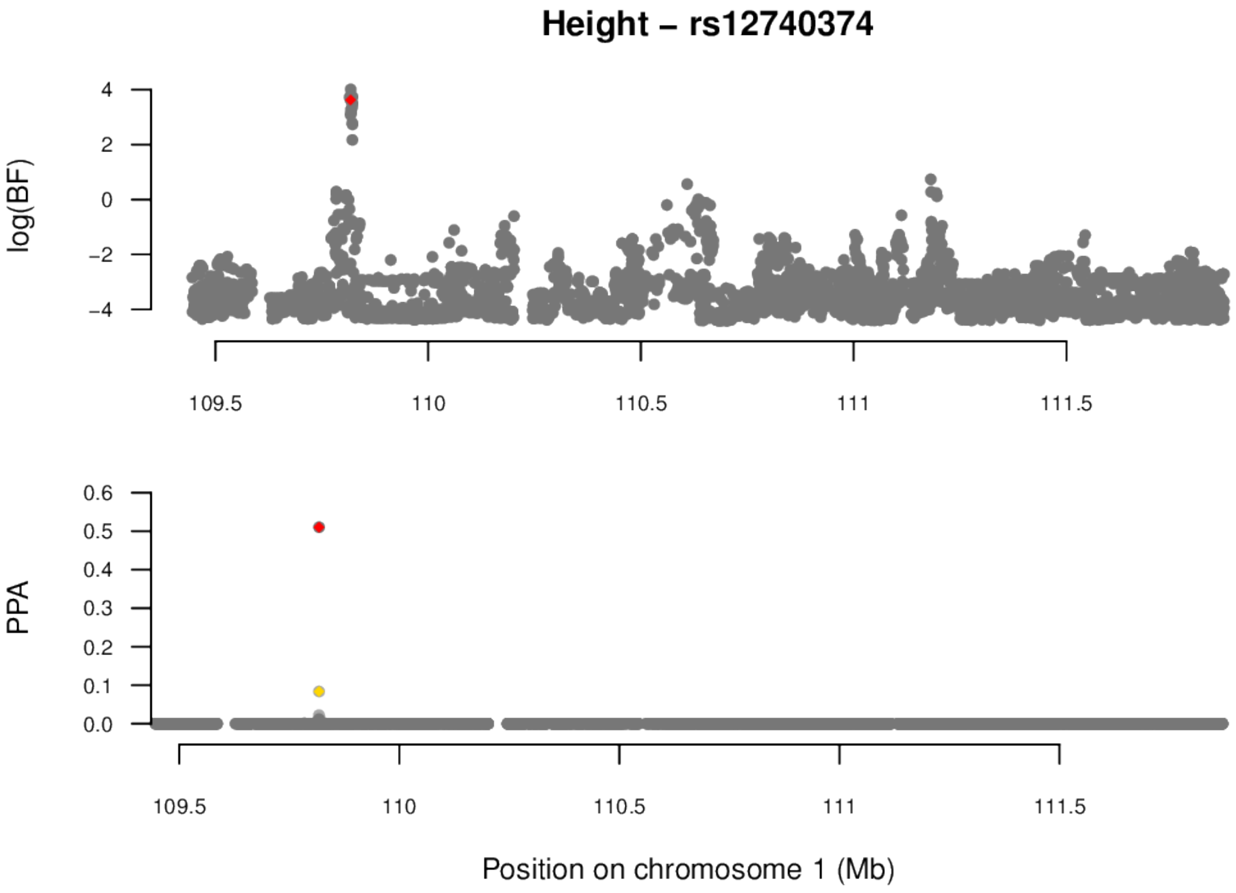
Height – rs12740374
log(BF)
4
2
0
−2
−4
109.5
110
110.5
111
111.5
PPA
0.6
0.5
0.4
0.3
0.2
0.1
0.0
109.5
110
110.5
111
111.5
Position on chromosome 1 (Mb)

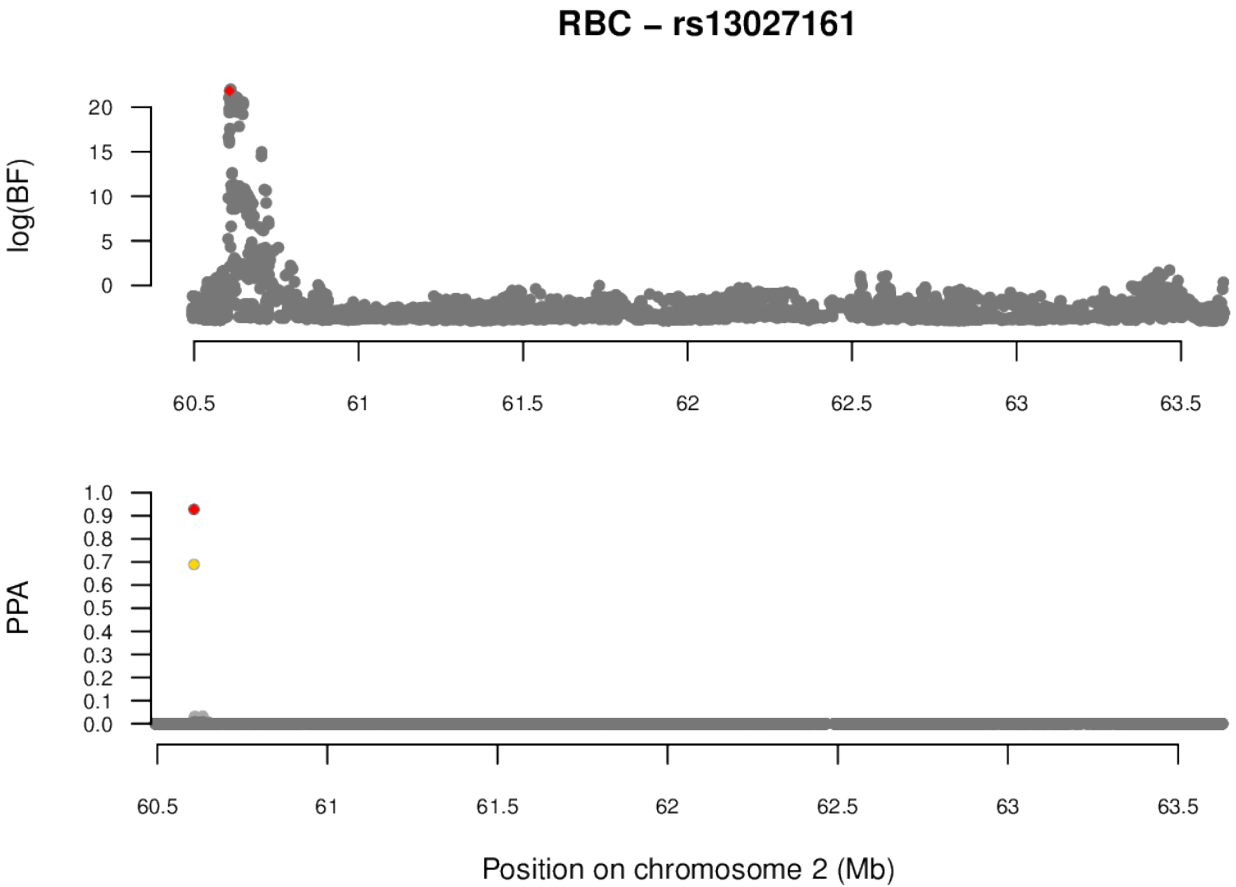
RBC – rs13027161
log(BF)
20
15
10
5
0
60.5
61
61.5
62
62.5
63
63.5
PPA
1.0
0.9
0.8
0.7
0.6
0.5
0.4
0.3
0.2
0.1
0.0
60.5
61
61.5
62
62.5
63
63.5
Position on chromosome 2 (Mb)

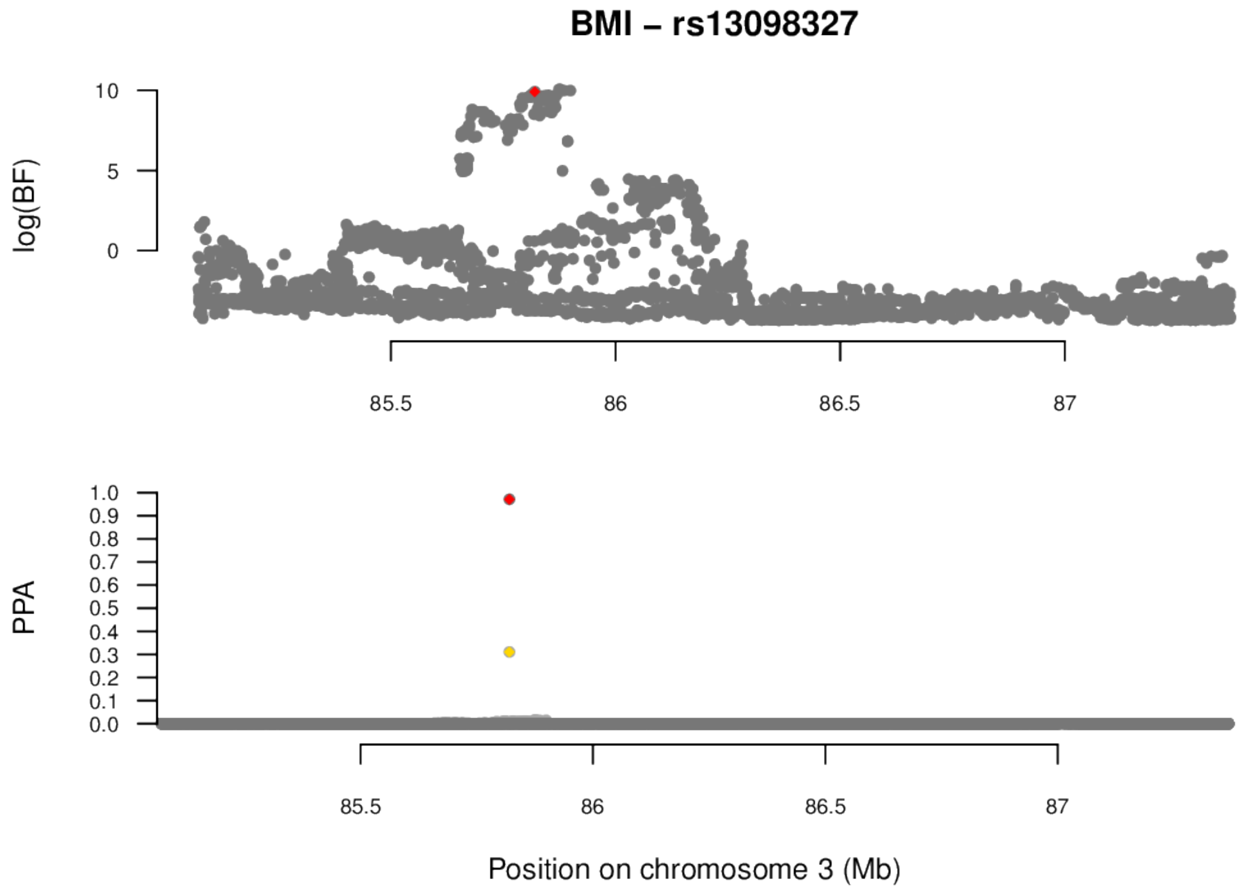

BMI – rs13098327
log(BF)
10
5
0
85.5
86
86.5
87
PPA
1.0
0.9
0.8
0.7
0.6
0.5
0.4
0.3
0.2
0.1
0.0
85.5
86
86.5
87
Position on chromosome 3 (Mb)

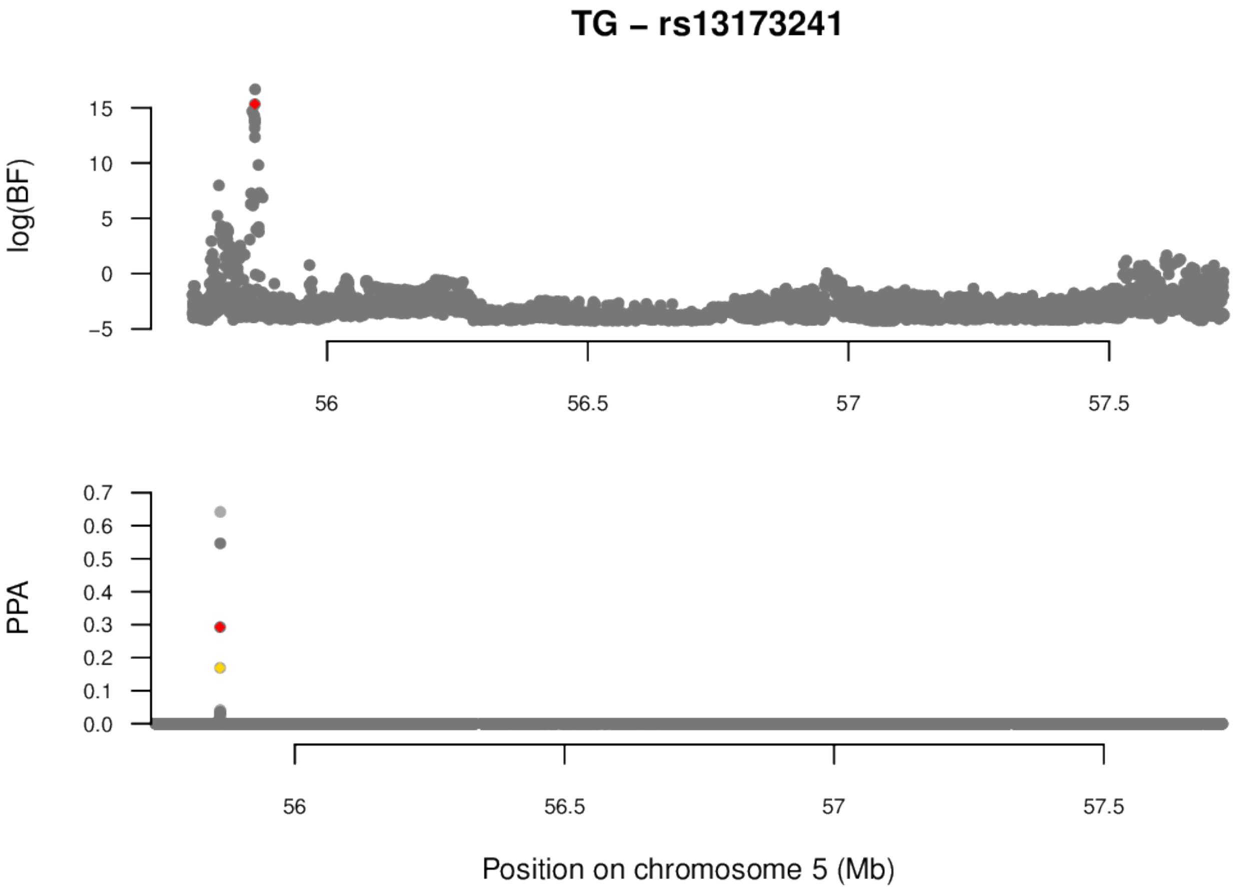
TG – rs13173241
log(BF)
15
10
5
0
−5
56
56.5
57
57.5
PPA
0.7
0.6
0.5
0.4
0.3
0.2
0.1
0.0
56
56.5
57
57.5
Position on chromosome 5 (Mb)

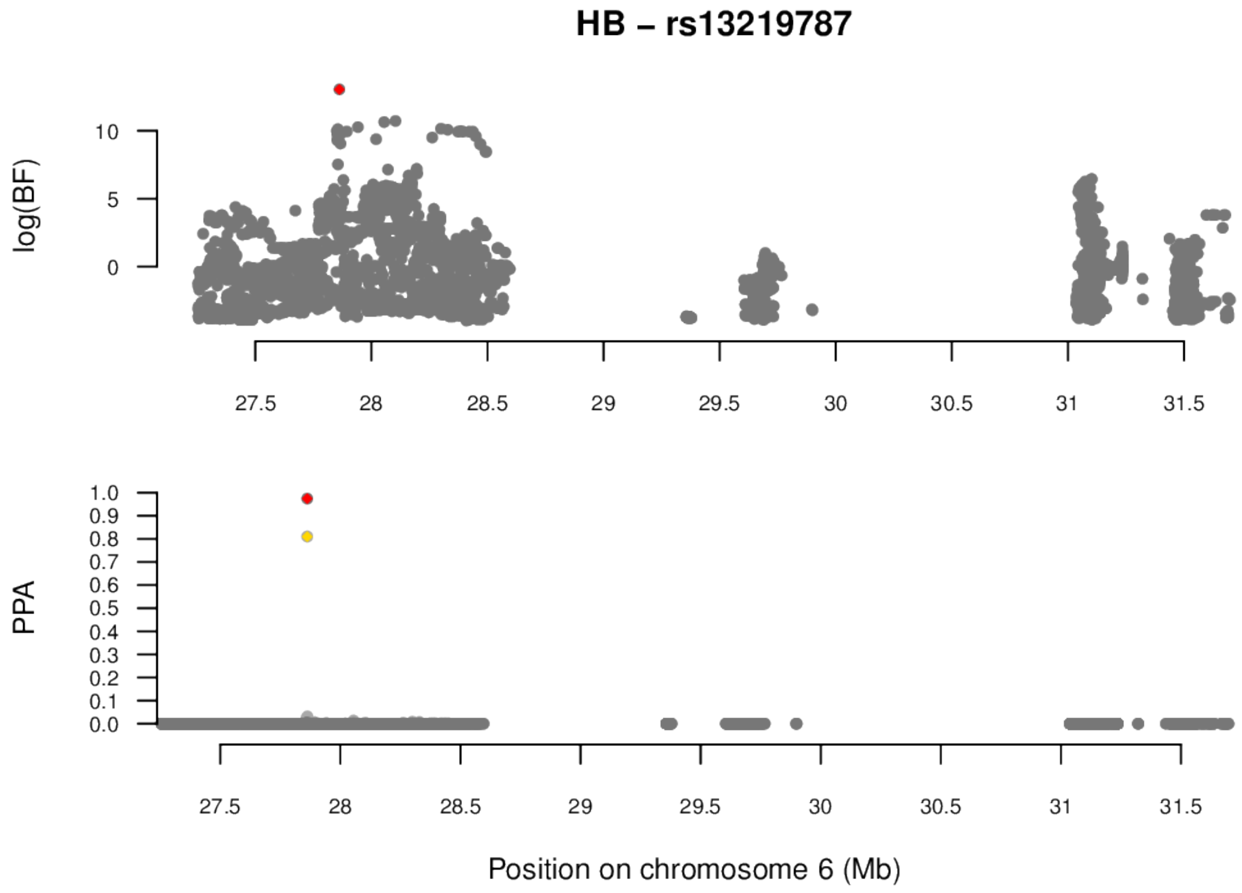

HB – rs13219787
log(BF)
10
5
0
27.5
28
28.5
29
29.5
30
30.5
31
31.5
PPA
1.0
0.9
0.8
0.7
0.6
0.5
0.4
0.3
0.2
0.1
0.0
27.5
28
28.5
29
29.5
30
30.5
31
31.5
Position on chromosome 6 (Mb)

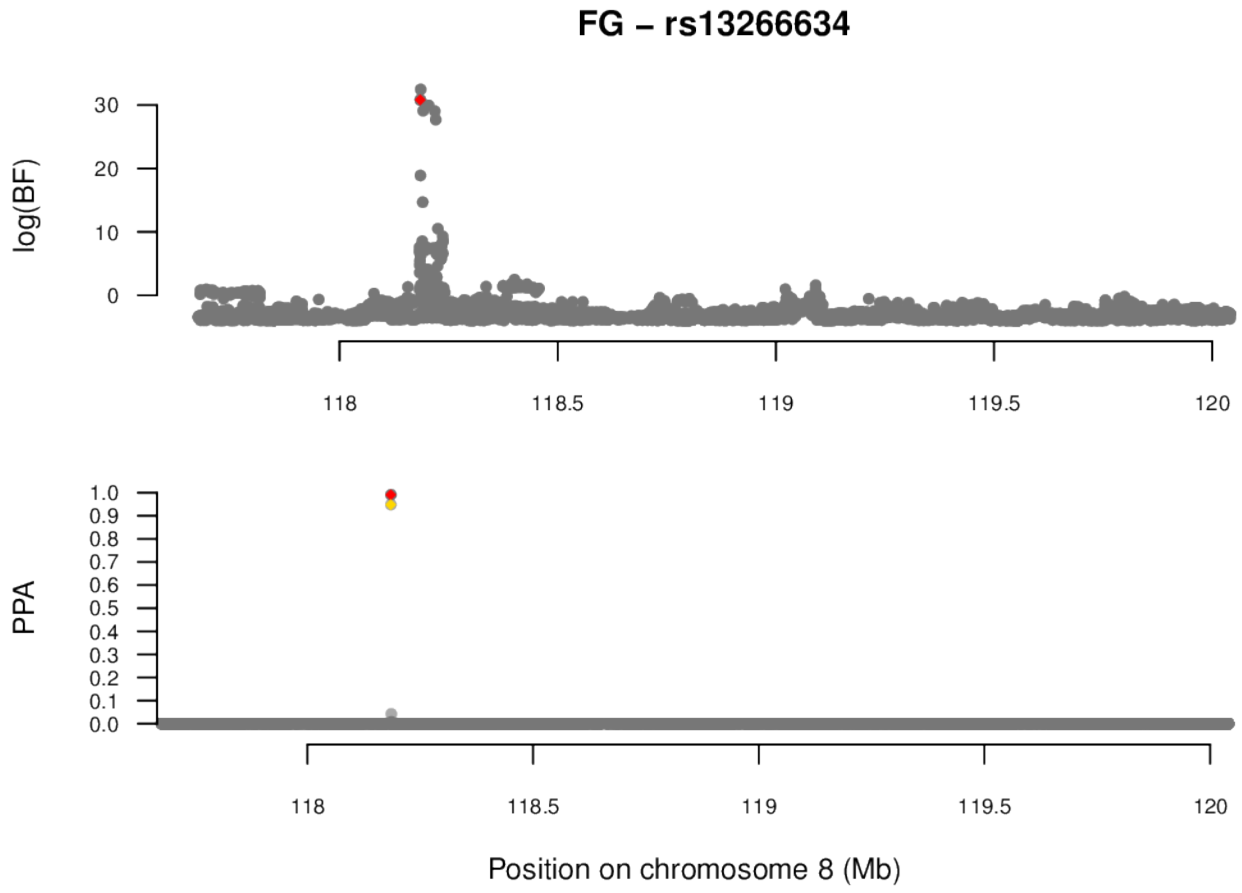

FG – rs13266634
log(BF)
30
20
10
0
118
118.5
119
119.5
120
PPA
1.0
0.9
0.8
0.7
0.6
0.5
0.4
0.3
0.2
0.1
0.0
118
118.5
119
119.5
120
Position on chromosome 8 (Mb)

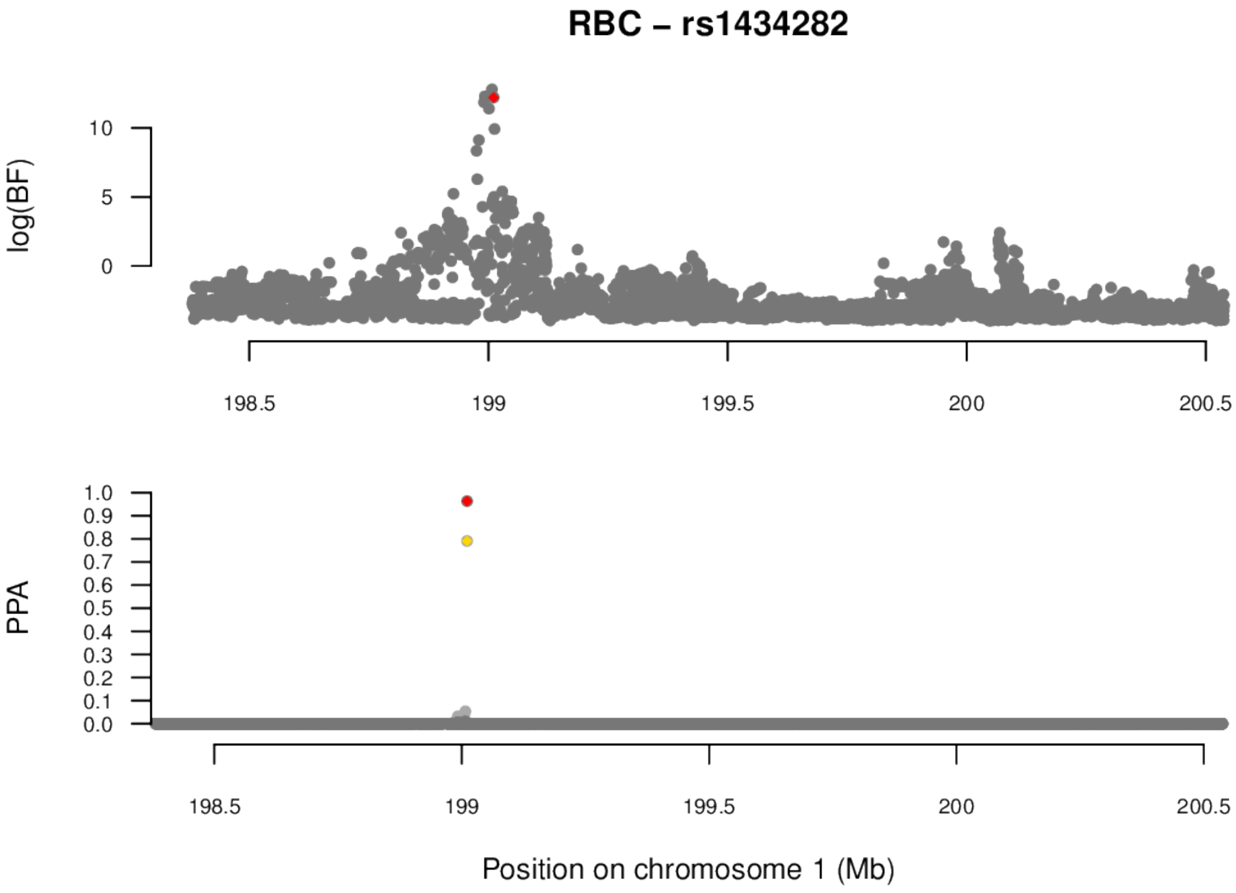

RBC – rs1434282
log(BF)
10
5
0
198.5
199
199.5
200
200.5
PPA
1.0
0.9
0.8
0.7
0.6
0.5
0.4
0.3
0.2
0.1
0.0
198.5
199
199.5
200
200.5
Position on chromosome 1 (Mb)

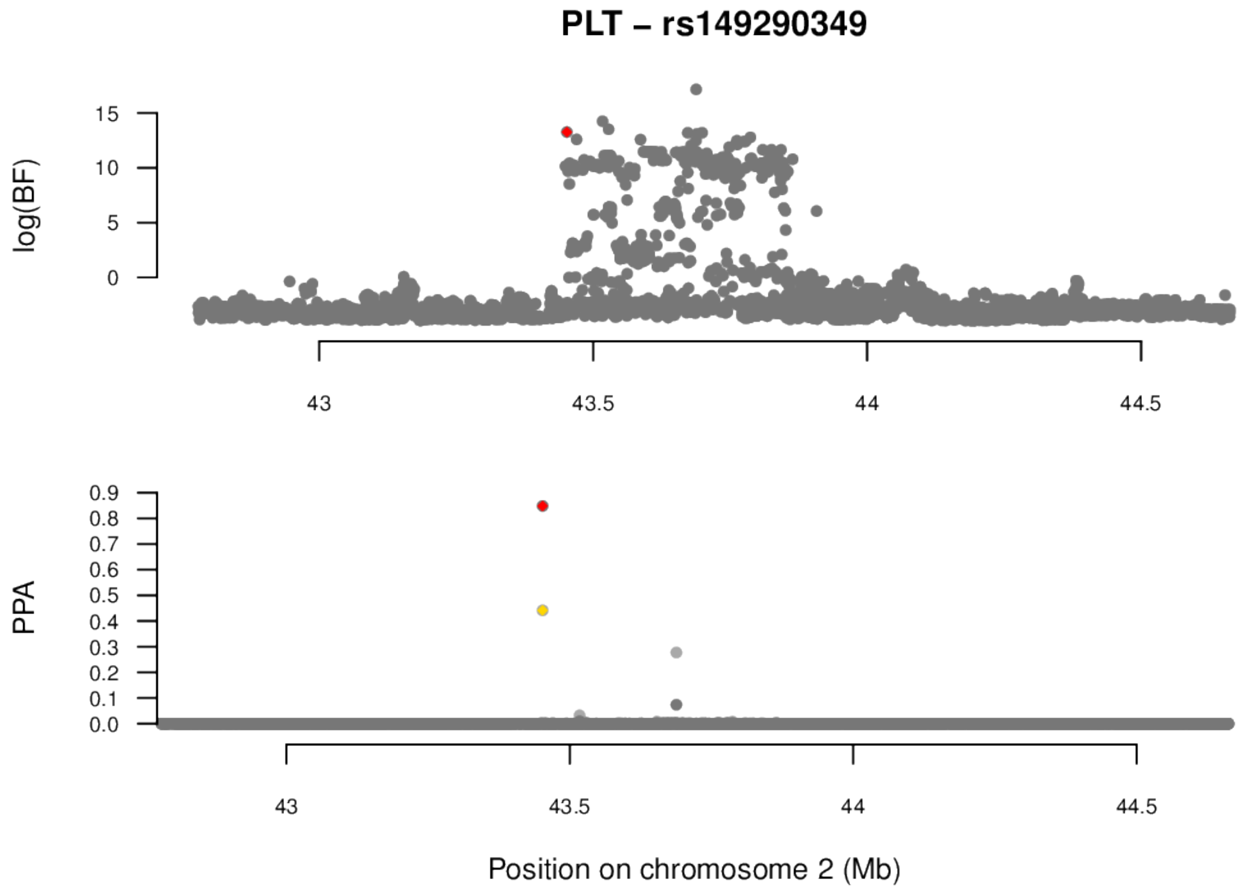
PLT – rs149290349
log(BF)
15
10
5
0
43
43.5
44
44.5
PPA
0.9
0.8
0.7
0.6
0.5
0.4
0.3
0.2
0.1
0.0
43
43.5
44
44.5
Position on chromosome 2 (Mb)

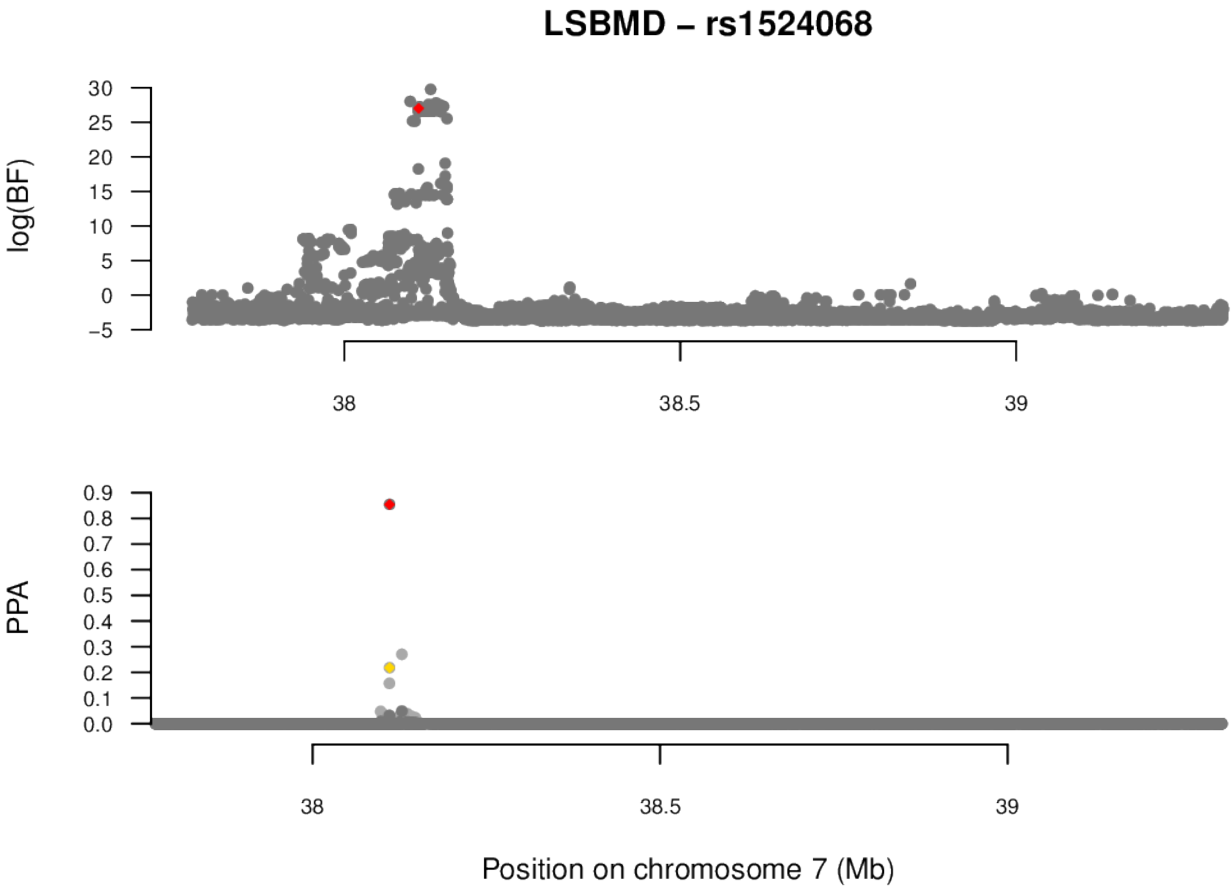

LSBMD – rs1524068
log(BF)
30
25
20
15
10
5
0
−5
38
38.5
39
PPA
0.9
0.8
0.7
0.6
0.5
0.4
0.3
0.2
0.1
0.0
38
38.5
39
Position on chromosome 7 (Mb)

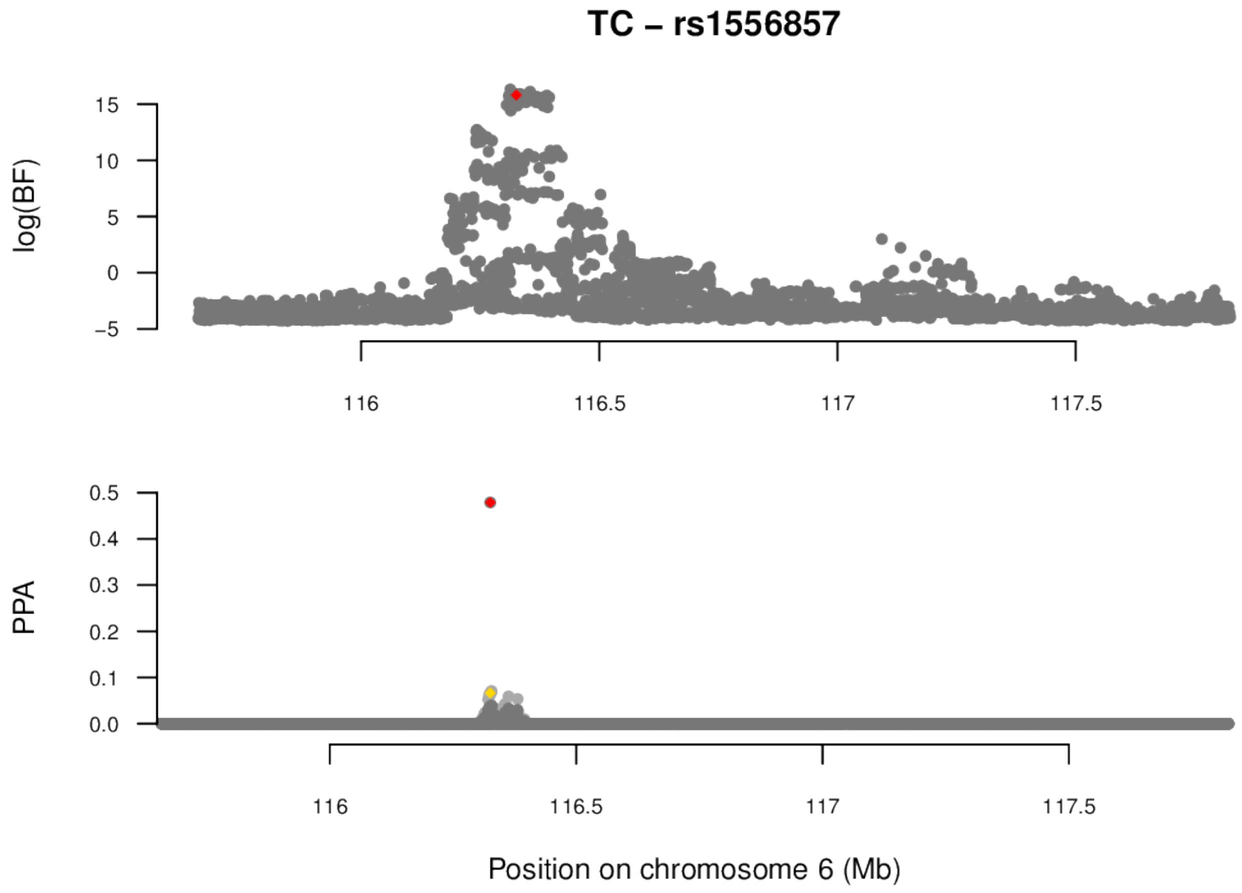
TC – rs1556857
log(BF)
15
10
5
0
−5
116
116.5
117
117.5
PPA
0.5
0.4
0.3
0.2
0.1
0.0
116
116.5
117
117.5
Position on chromosome 6 (Mb)

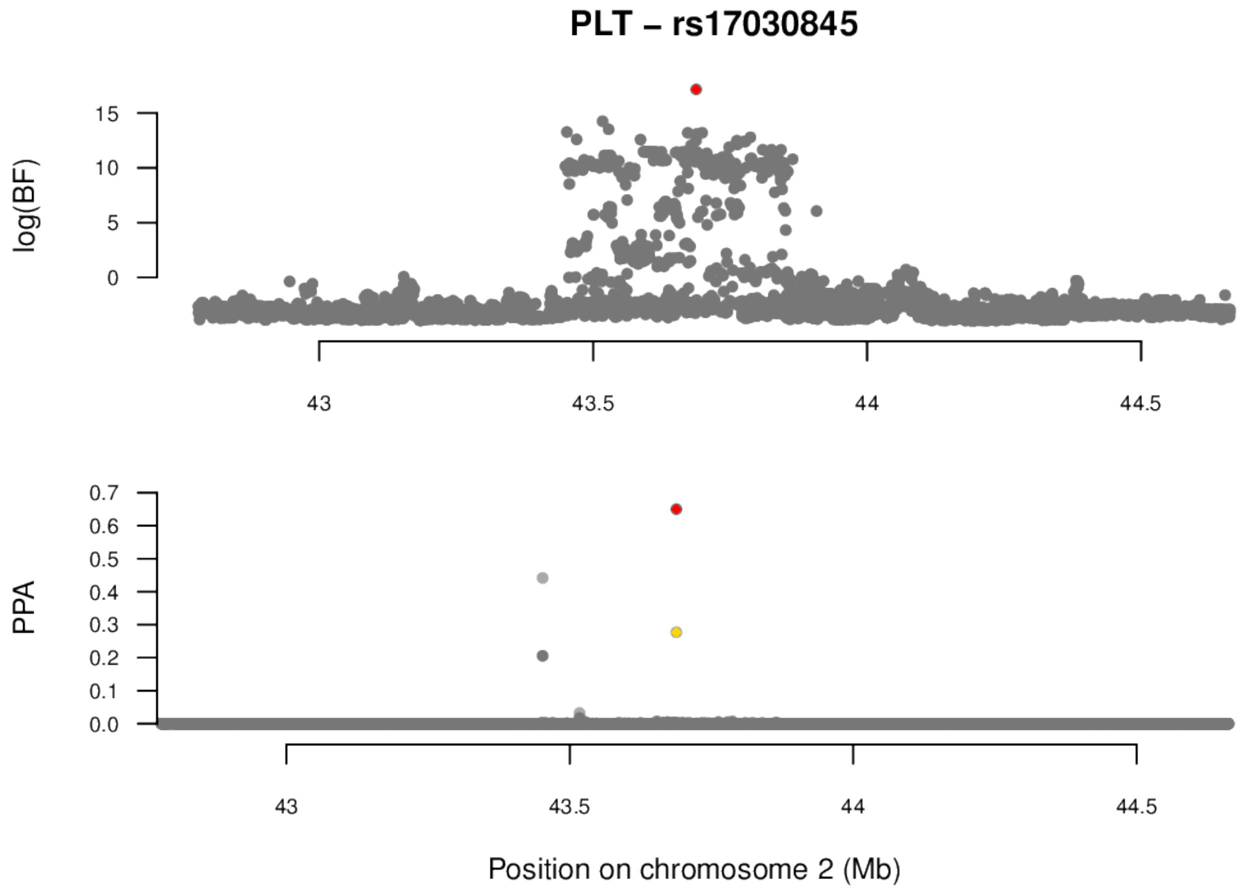
PLT – rs17030845
log(BF)
15
10
5
0
43
43.5
44
44.5
PPA
0.7
0.6
0.5
0.4
0.3
0.2
0.1
0.0
43
43.5
44
44.5
Position on chromosome 2 (Mb)

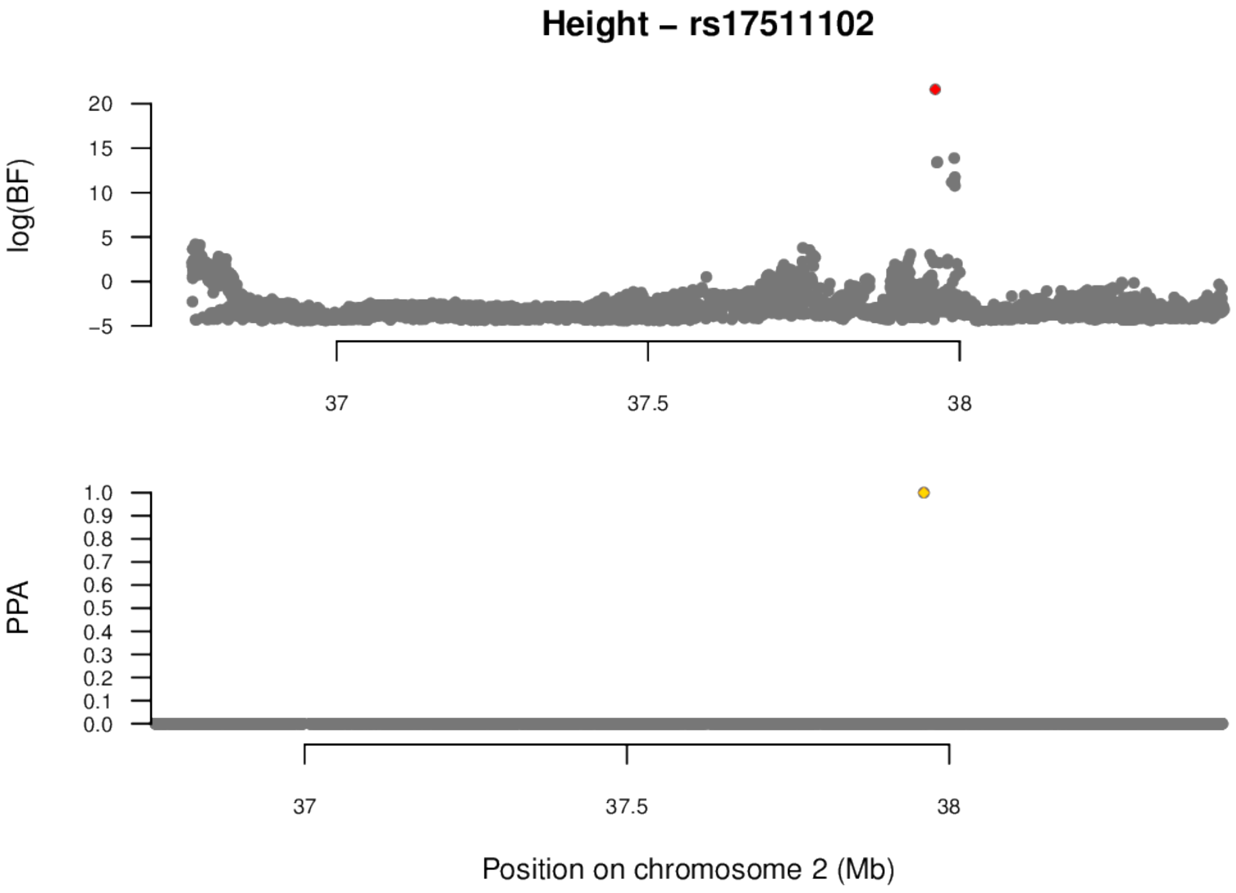
Height – rs17511102
log(BF)
20
15
10
5
0
−5
37
37.5
38
PPA
1.0
0.9
0.8
0.7
0.6
0.5
0.4
0.3
0.2
0.1
0.0
37
37.5
38
Position on chromosome 2 (Mb)

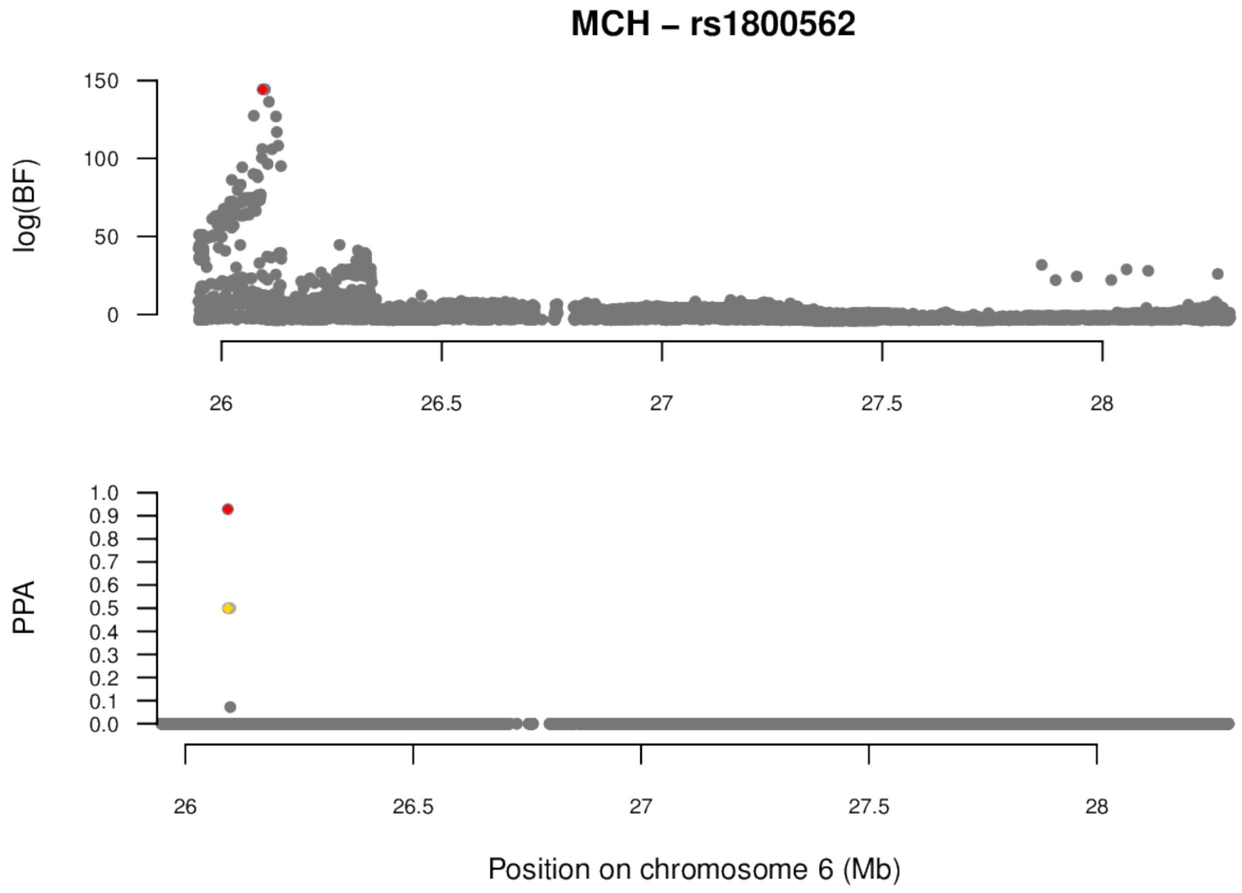

MCH – rs1800562
log(BF)
150
100
50
0
26
26.5
27
27.5
28
PPA
1.0
0.9
0.8
0.7
0.6
0.5
0.4
0.3
0.2
0.1
0.0
26
26.5
27
27.5
28
Position on chromosome 6 (Mb)

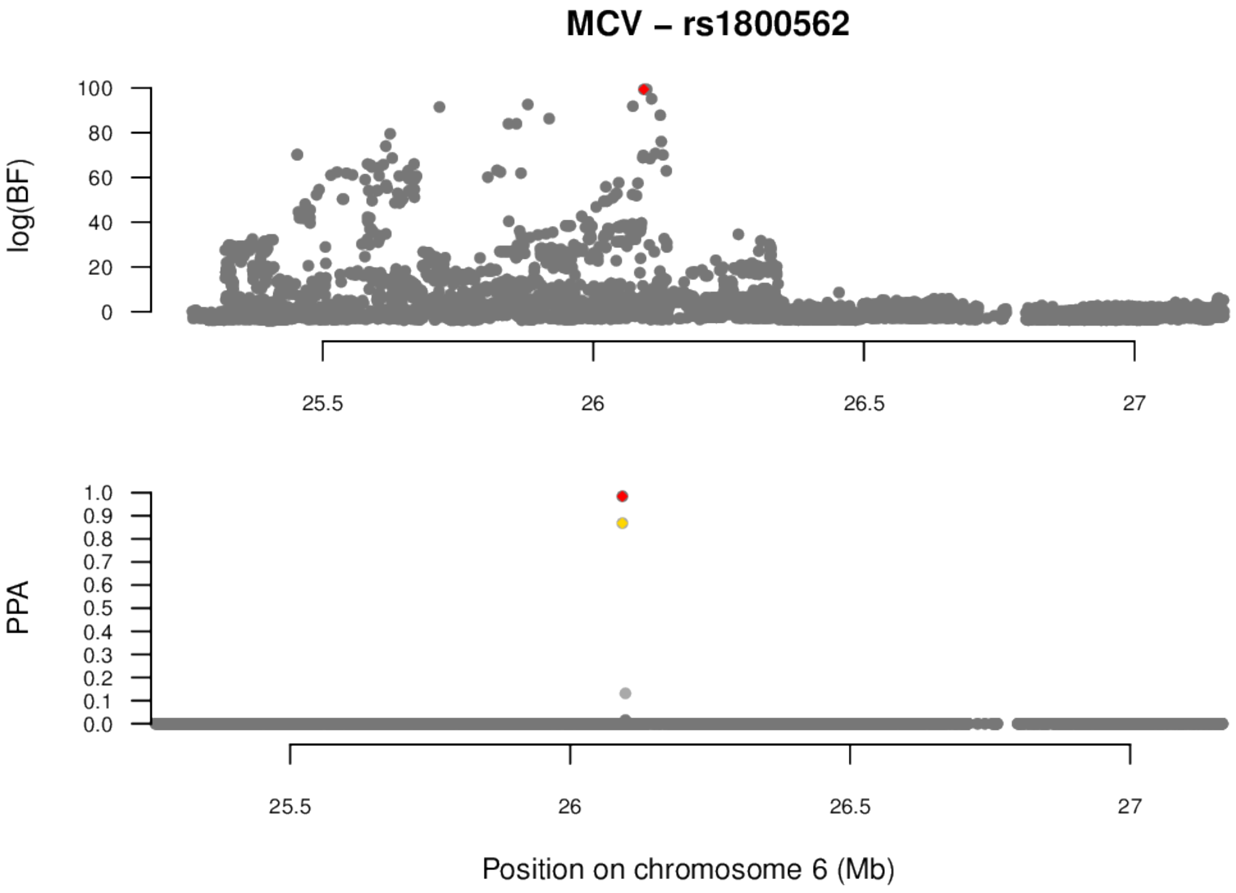
MCV – rs1800562
log(BF)
100
80
60
40
20
0
25.5
26
26.5
27
PPA
1.0
0.9
0.8
0.7
0.6
0.5
0.4
0.3
0.2
0.1
0.0
25.5
26
26.5
27
Position on chromosome 6 (Mb)

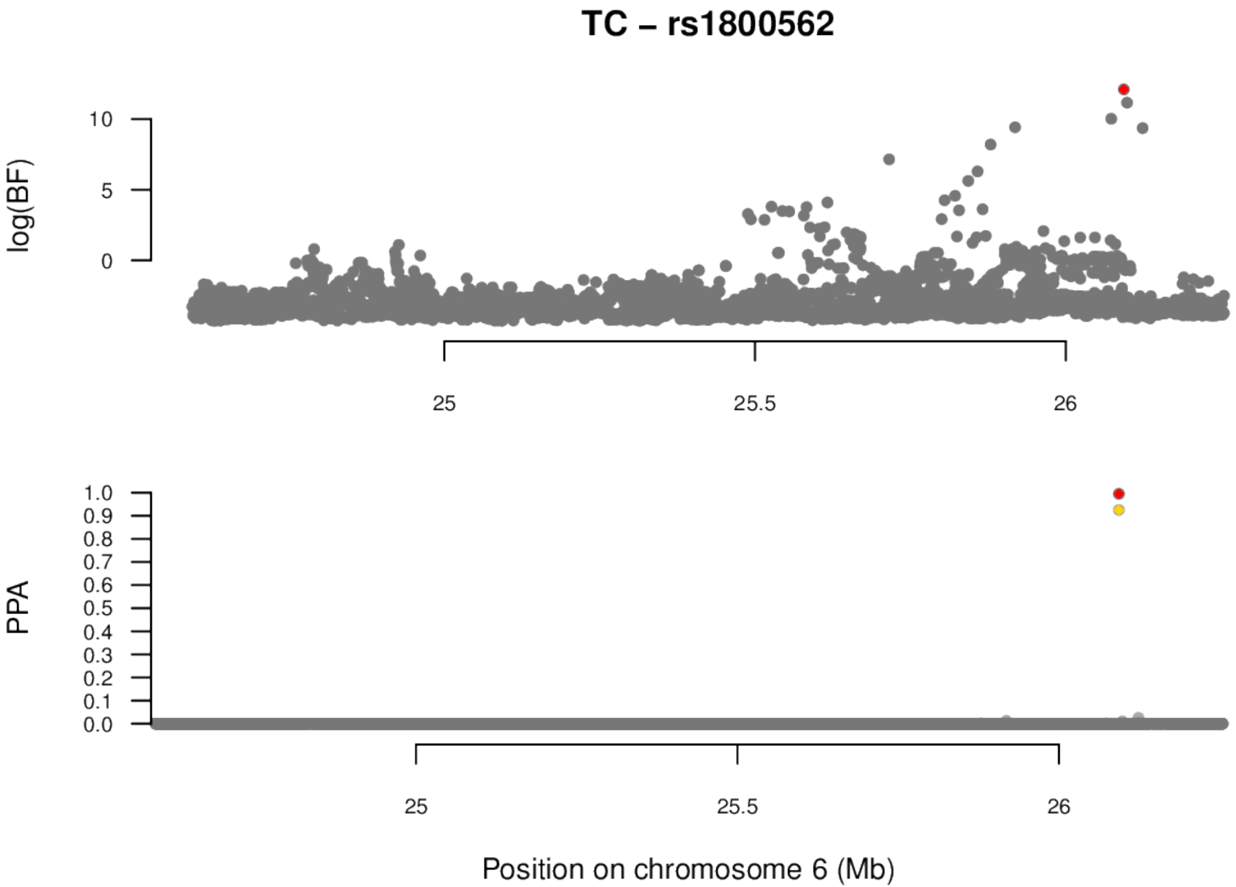

TC – rs1800562
log(BF)
10
5
0
25
25.5
26
PPA
1.0
0.9
0.8
0.7
0.6
0.5
0.4
0.3
0.2
0.1
0.0
25
25.5
26
Position on chromosome 6 (Mb)

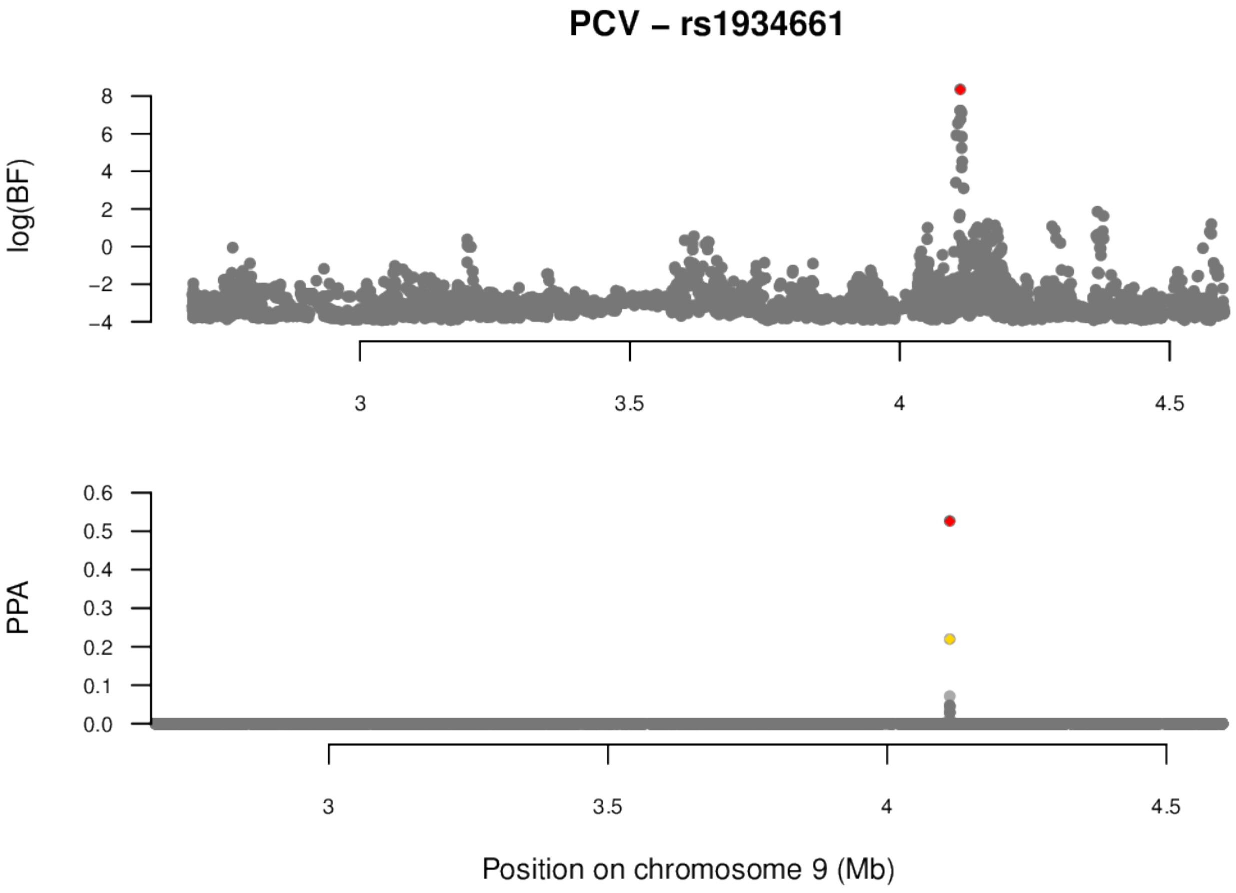

PCV – rs1934661
log(BF)
8
6
4
2
0
−2
−4
3
3.5
4
4.5
PPA
0.6
0.5
0.4
0.3
0.2
0.1
0.0
3
3.5
4
4.5
Position on chromosome 9 (Mb)

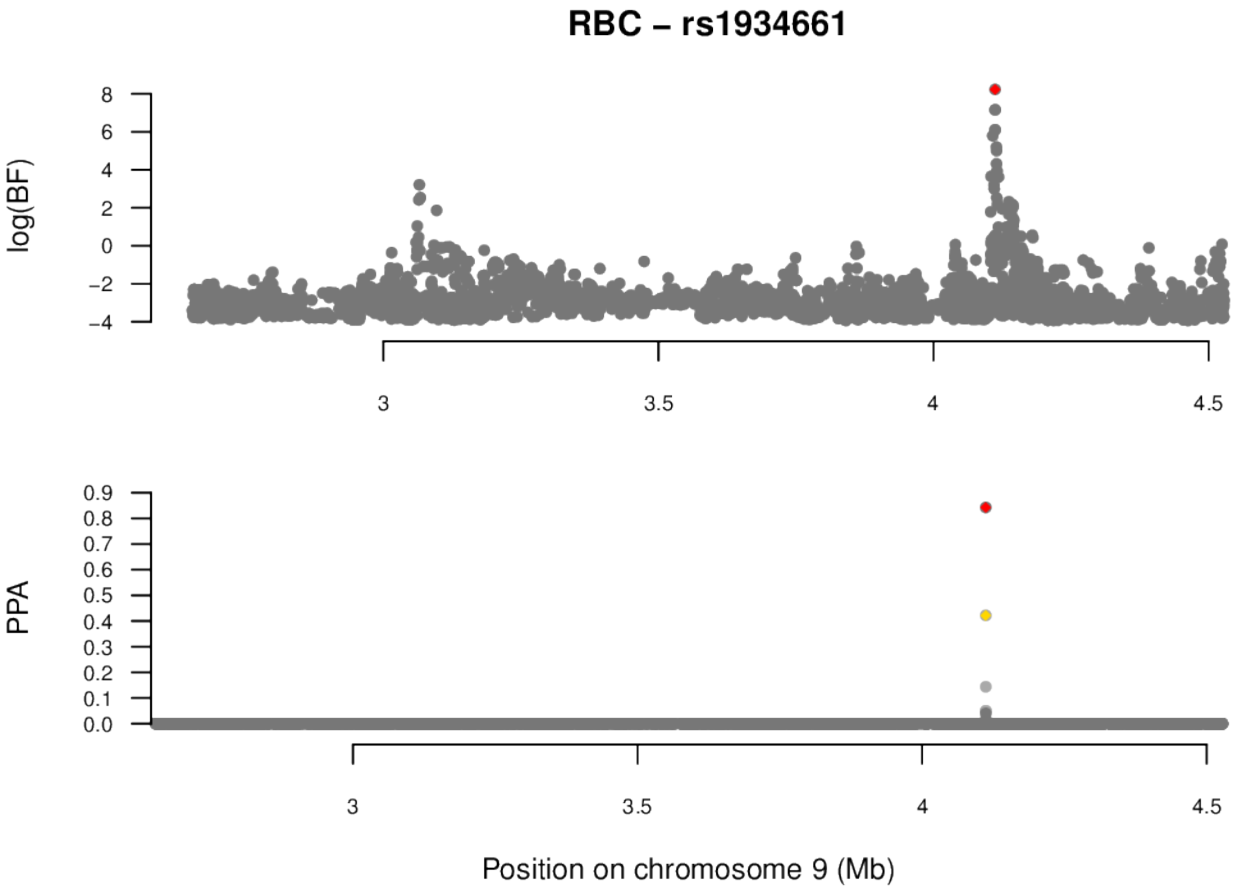

RBC – rs1934661
log(BF)
8
6
4
2
0
−2
−4
3
3.5
4
4.5
PPA
0.9
0.8
0.7
0.6
0.5
0.4
0.3
0.2
0.1
0.0
3
3.5
4
4.5
Position on chromosome 9 (Mb)

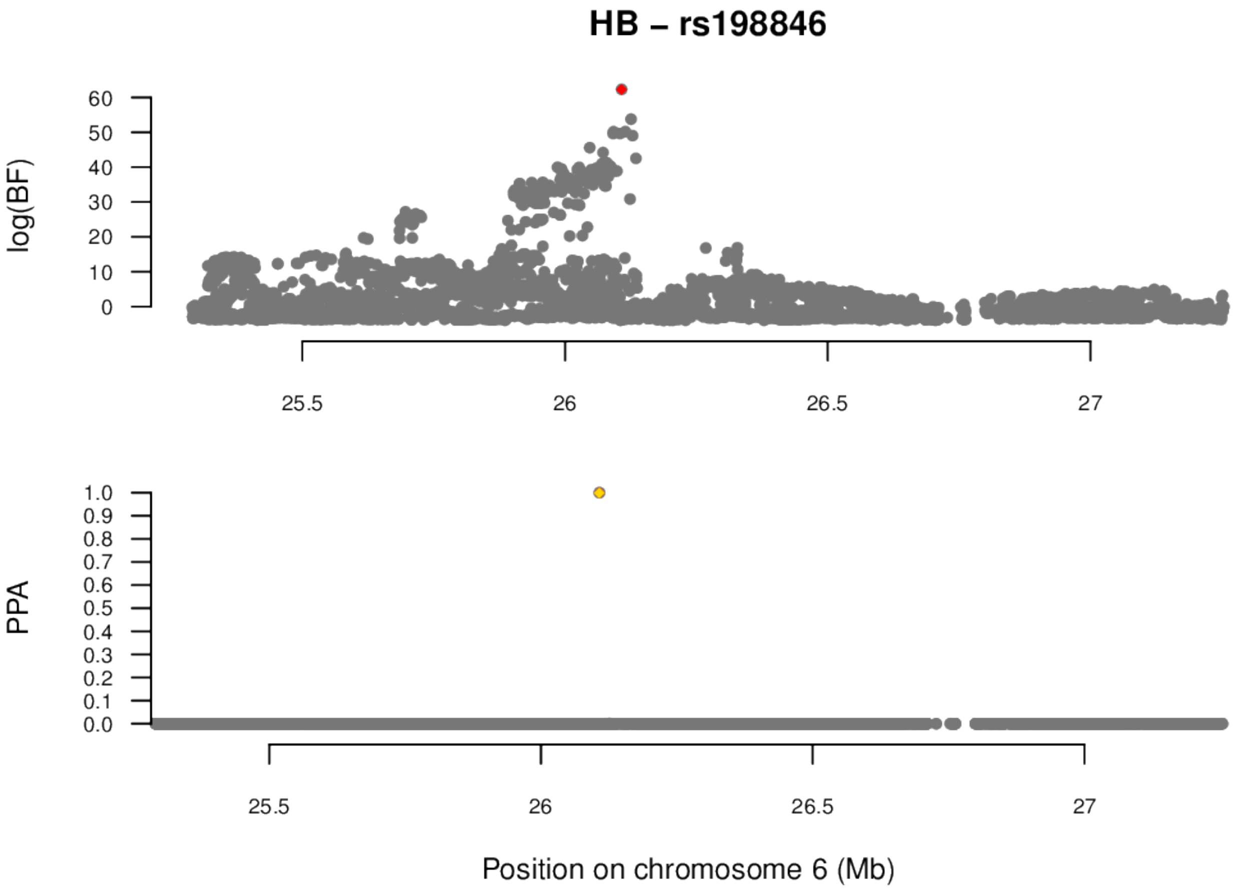
HB – rs198846
log(BF)
60
50
40
30
20
10
0
25.5
26
26.5
27
PPA
1.0
0.9
0.8
0.7
0.6
0.5
0.4
0.3
0.2
0.1
0.0
25.5
26
26.5
27
Position on chromosome 6 (Mb)

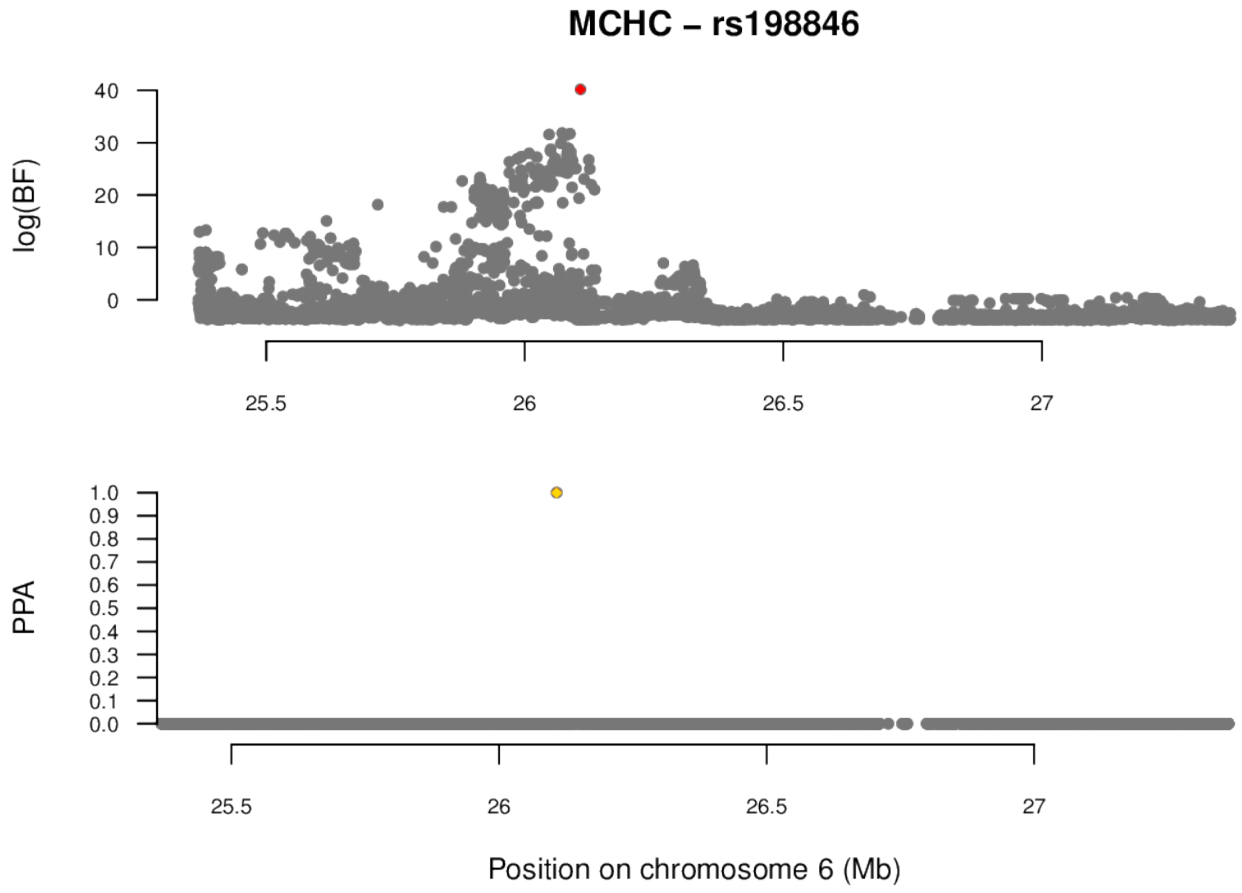

MCHC – rs198846
log(BF)
40
30
20
10
0
25.5
26
26.5
27
PPA
1.0
0.9
0.8
0.7
0.6
0.5
0.4
0.3
0.2
0.1
0.0
25.5
26
26.5
27
Position on chromosome 6 (Mb)

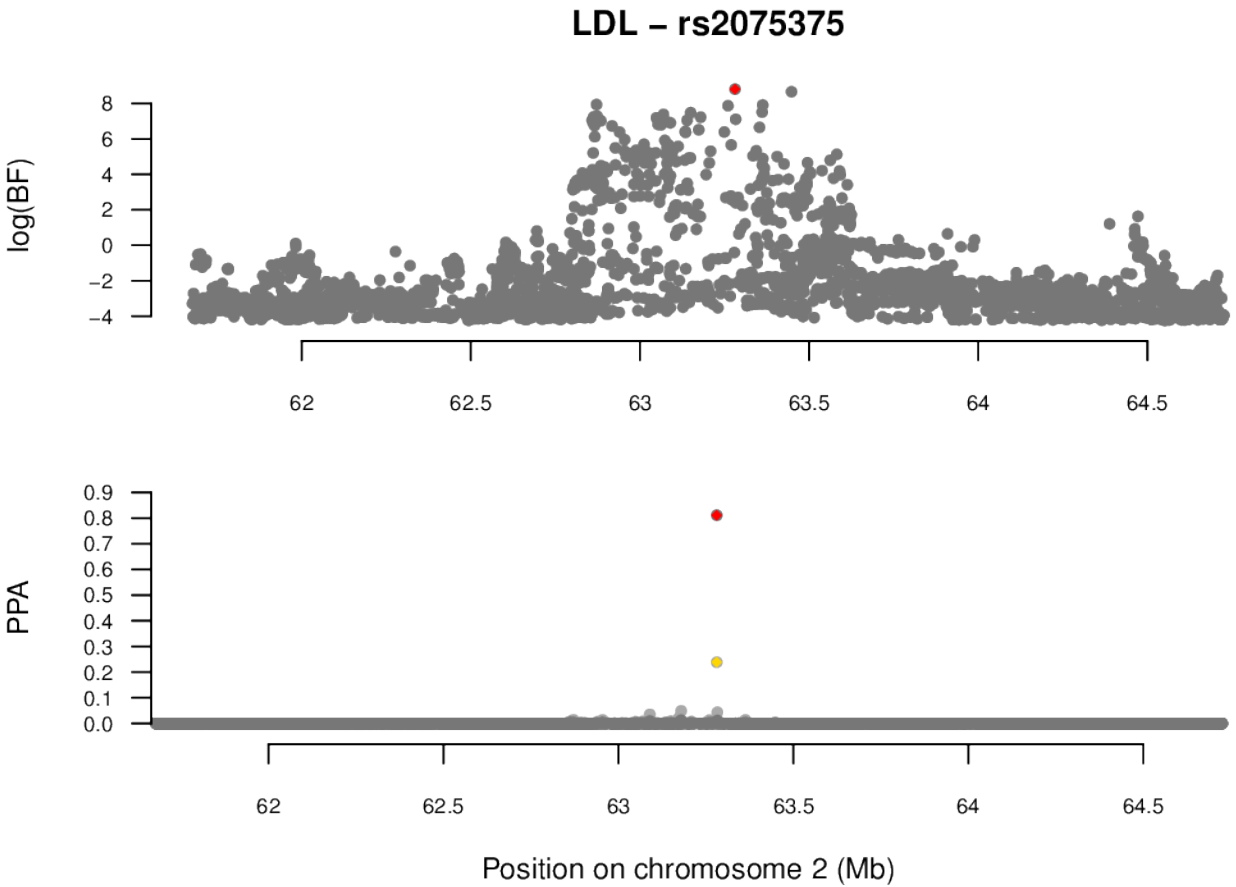
LDL – rs2075375
log(BF)
8
6
4
2
0
−2
−4
62
62.5
63
63.5
64
64.5
PPA
0.9
0.8
0.7
0.6
0.5
0.4
0.3
0.2
0.1
0.0
62
62.5
63
63.5
64
64.5
Position on chromosome 2 (Mb)

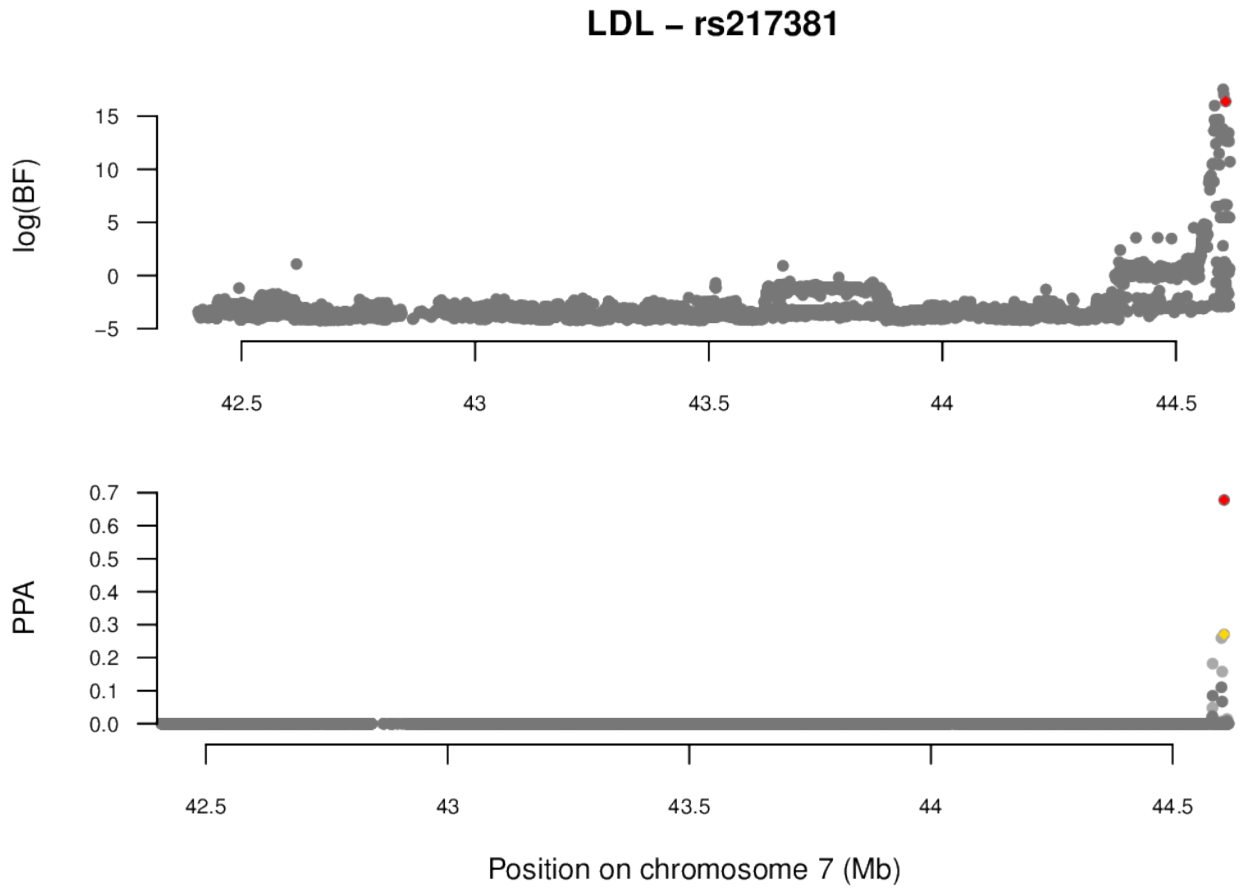

LDL – rs217381
log(BF)
15
10
5
0
−5
42.5
43
43.5
44
44.5
PPA
0.7
0.6
0.5
0.4
0.3
0.2
0.1
0.0
42.5
43
43.5
44
44.5
Position on chromosome 7 (Mb)

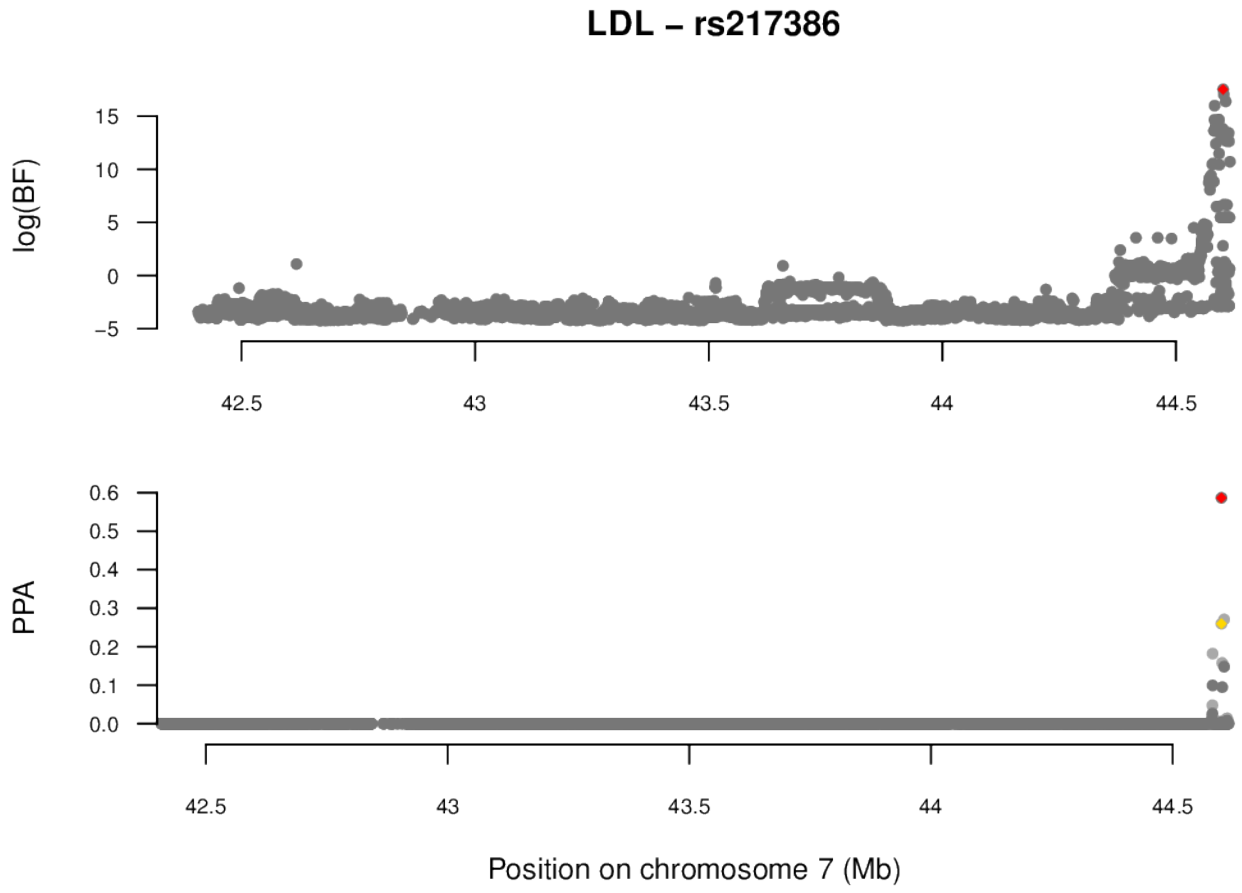
LDL – rs217386
log(BF)
15
10
5
0
−5
42.5
43
43.5
44
44.5
PPA
0.6
0.5
0.4
0.3
0.2
0.1
0.0
42.5
43
43.5
44
44.5
Position on chromosome 7 (Mb)

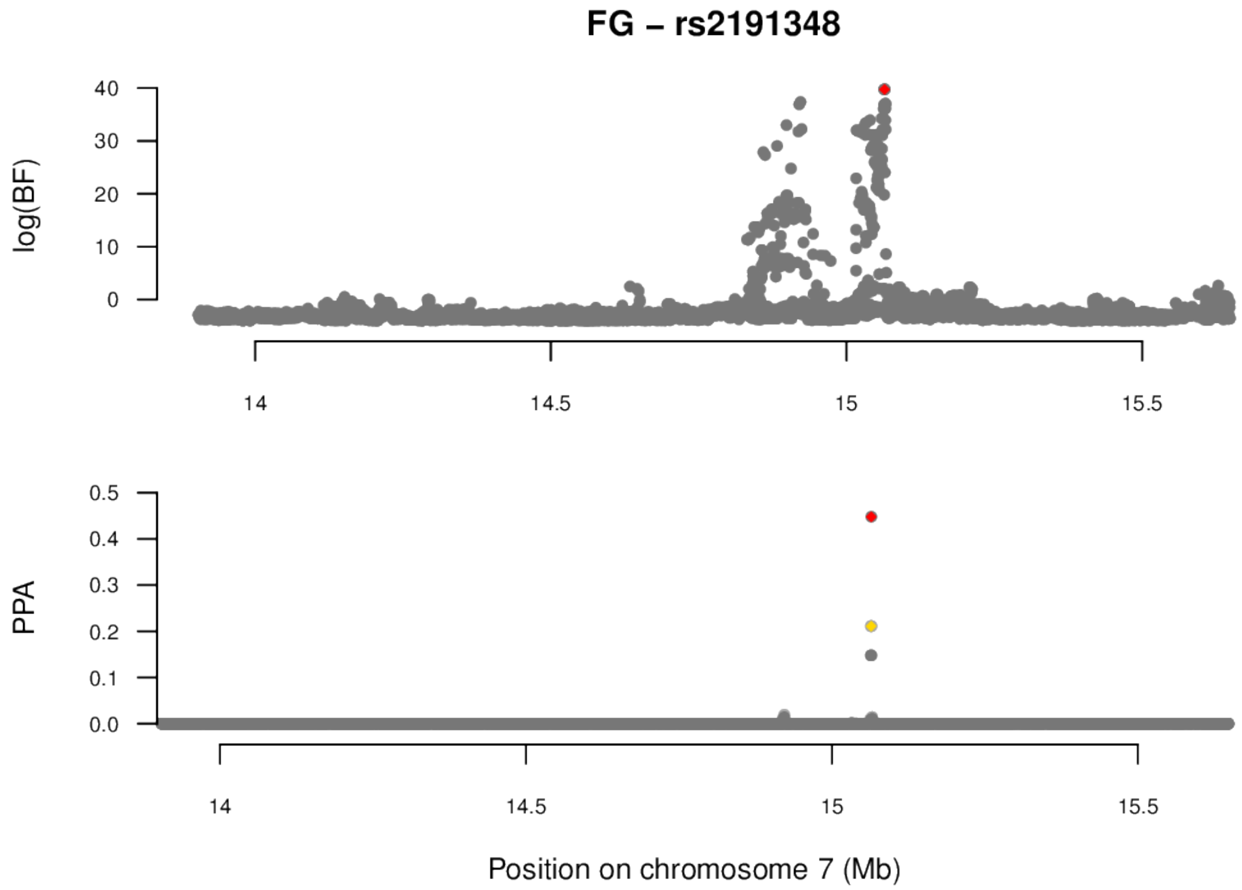

FG – rs2191348
log(BF)
40
30
20
10
0
14
14.5
15
15.5
PPA
0.5
0.4
0.3
0.2
0.1
0.0
14
14.5
15
15.5
Position on chromosome 7 (Mb)

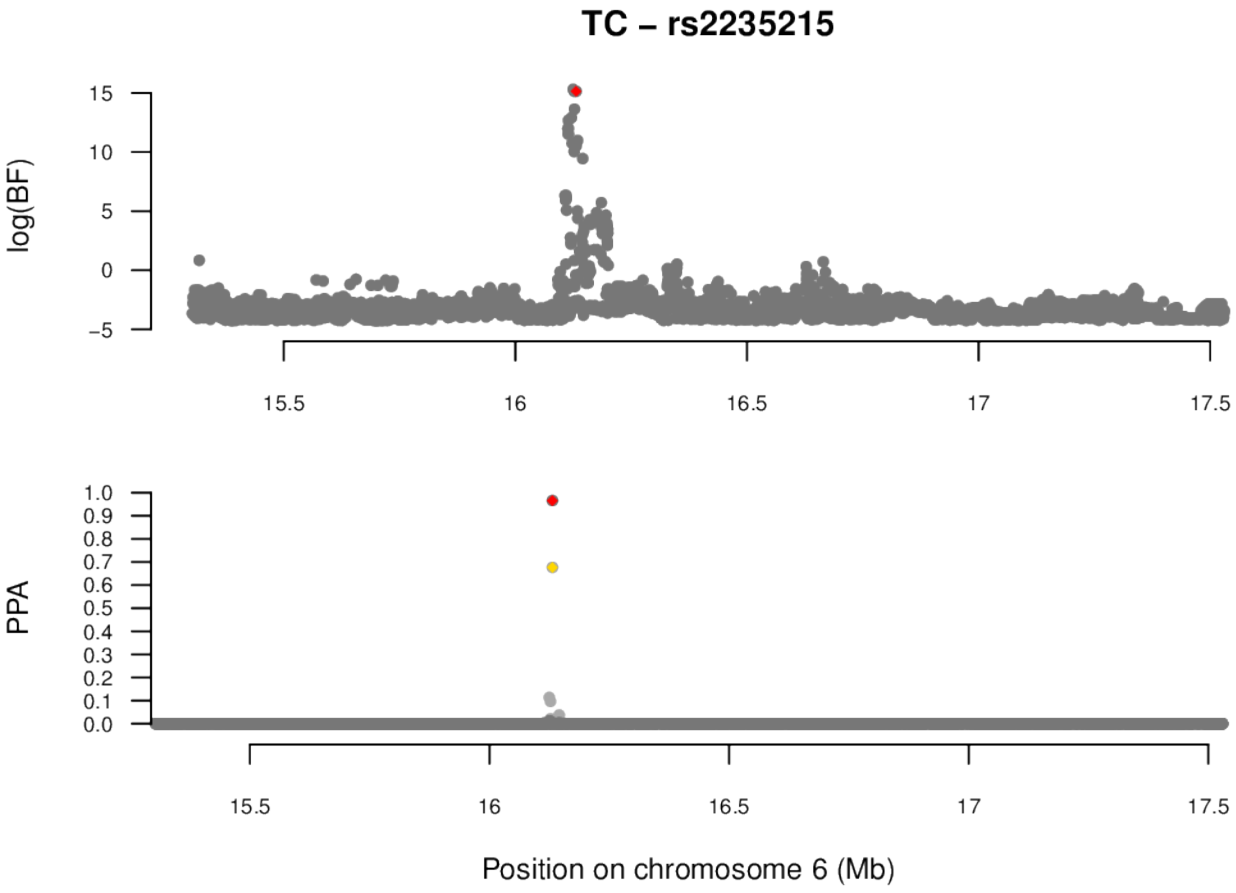

TC – rs2235215
log(BF)
15
10
5
0
−5
15.5
16
16.5
17
17.5
PPA
1.0
0.9
0.8
0.7
0.6
0.5
0.4
0.3
0.2
0.1
0.0
15.5
16
16.5
17
17.5
Position on chromosome 6 (Mb)

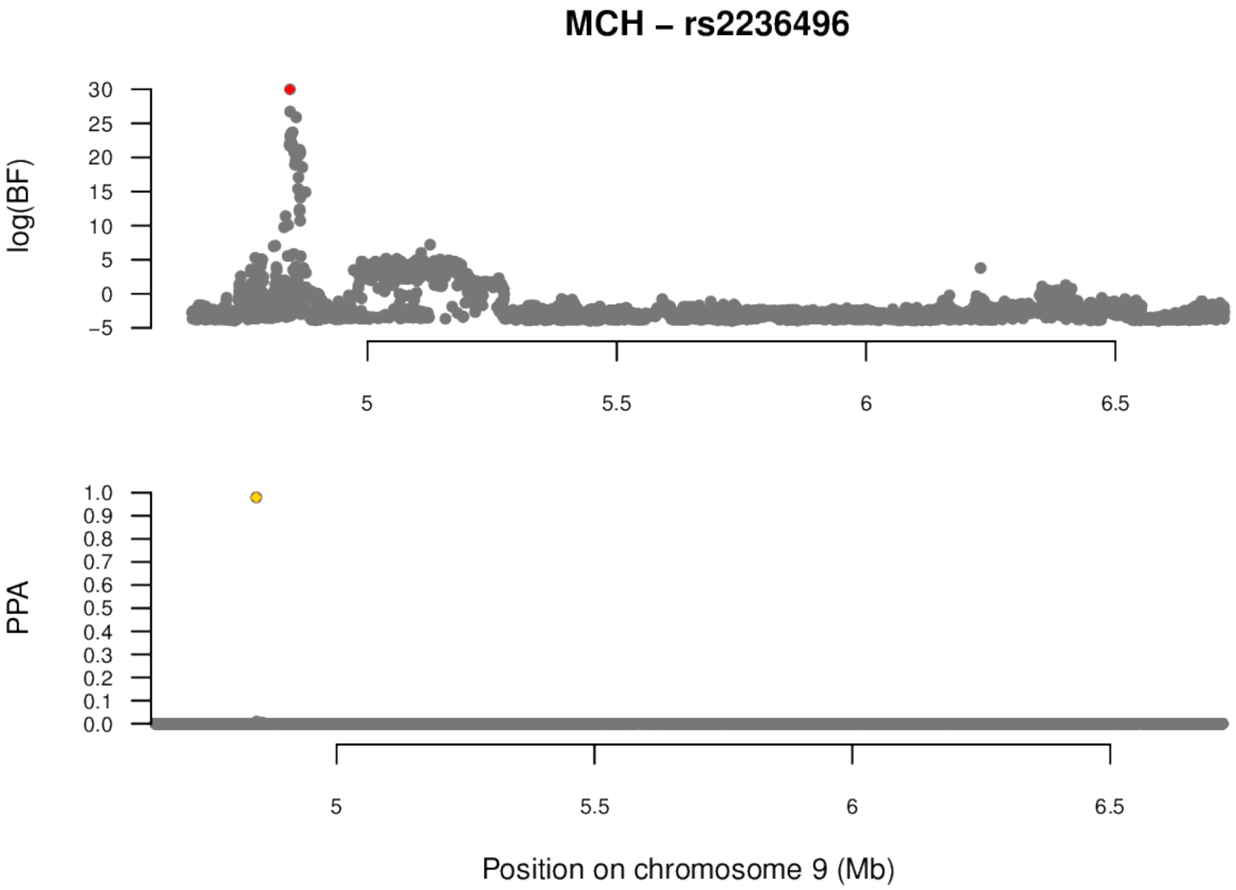

MCH – rs2236496
log(BF)
30
25
20
15
10
5
0
−5
5
5.5
6
6.5
PPA
1.0
0.9
0.8
0.7
0.6
0.5
0.4
0.3
0.2
0.1
0.0
5
5.5
6
6.5
Position on chromosome 9 (Mb)

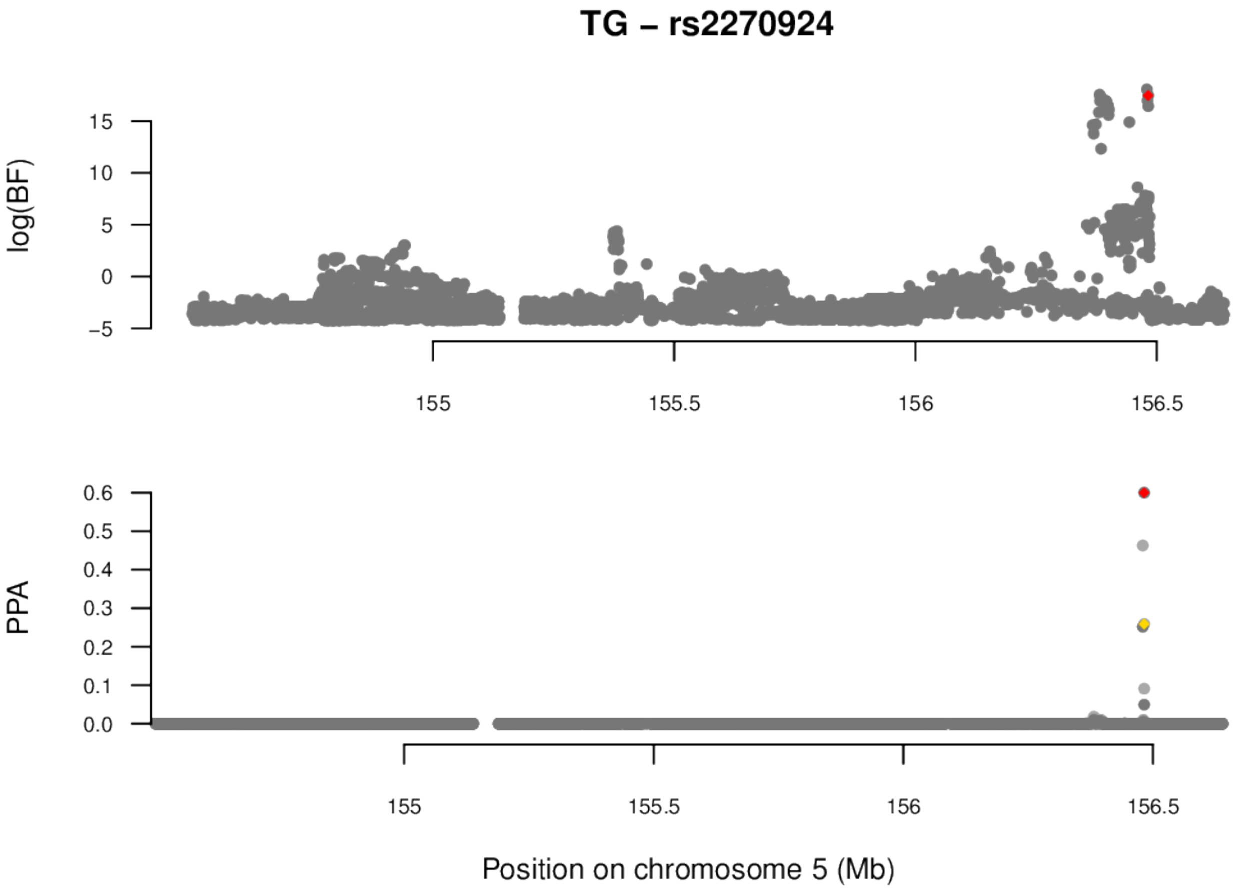

TG – rs2270924
log(BF)
15
10
5
0
−5
155
155.5
156
156.5
PPA
0.6
0.5
0.4
0.3
0.2
0.1
0.0
155
155.5
156
156.5
Position on chromosome 5 (Mb)

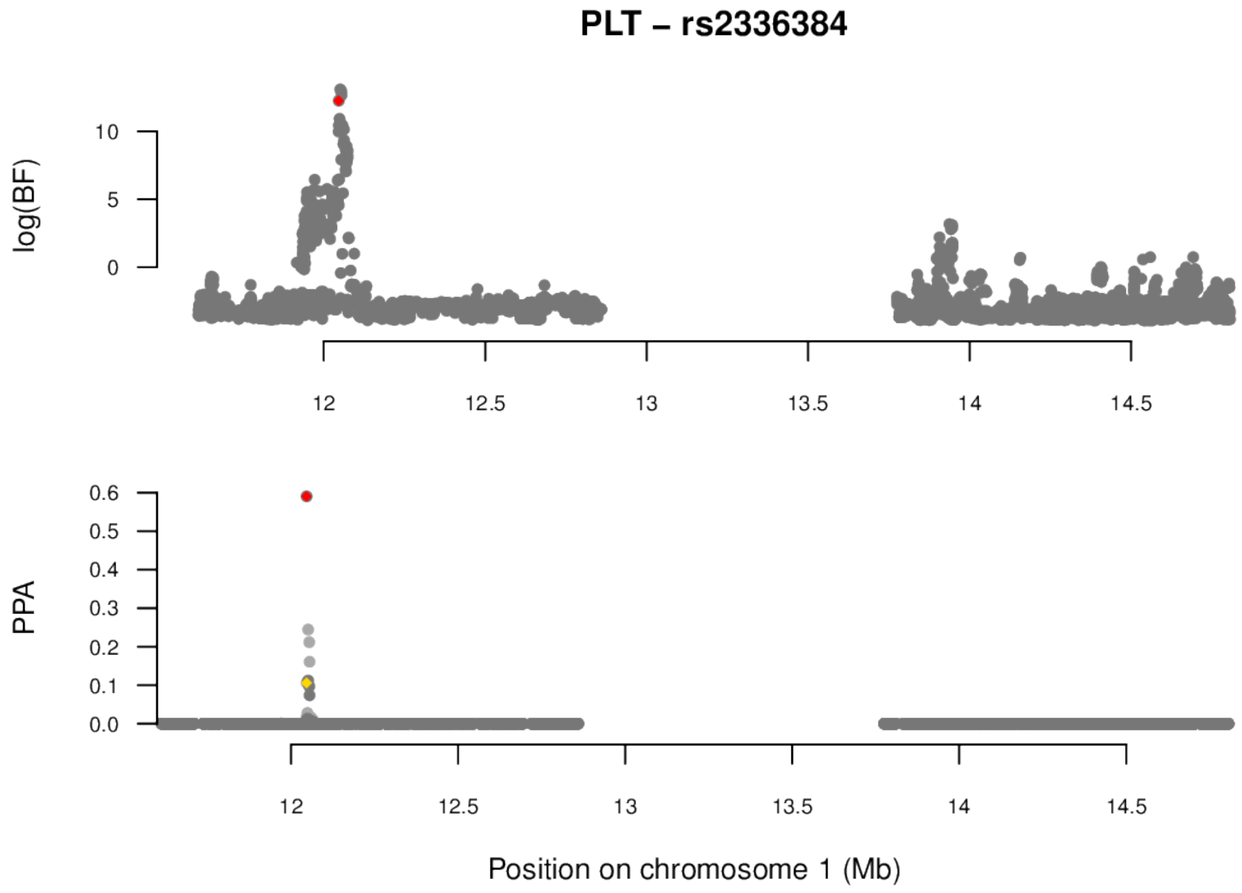

PLT – rs2336384
log(BF)
10
5
0
12
12.5
13
13.5
14
14.5
PPA
0.6
0.5
0.4
0.3
0.2
0.1
0.0
12
12.5
13
13.5
14
14.5
Position on chromosome 1 (Mb)

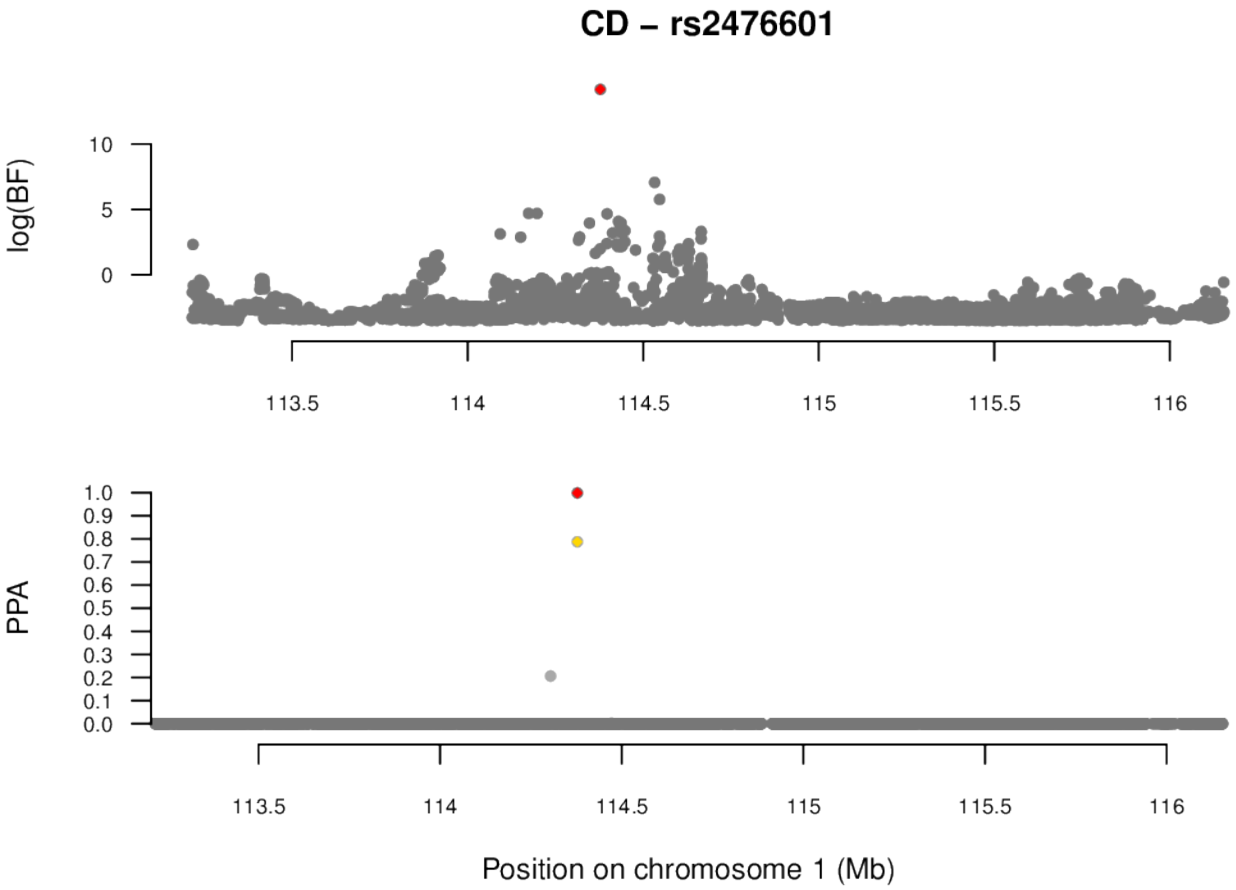
CD – rs2476601
log(BF)
10
5
0
113.5
114
114.5
115
115.5
116
PPA
1.0
0.9
0.8
0.7
0.6
0.5
0.4
0.3
0.2
0.1
0.0
113.5
114
114.5
115
115.5
116
Position on chromosome 1 (Mb)

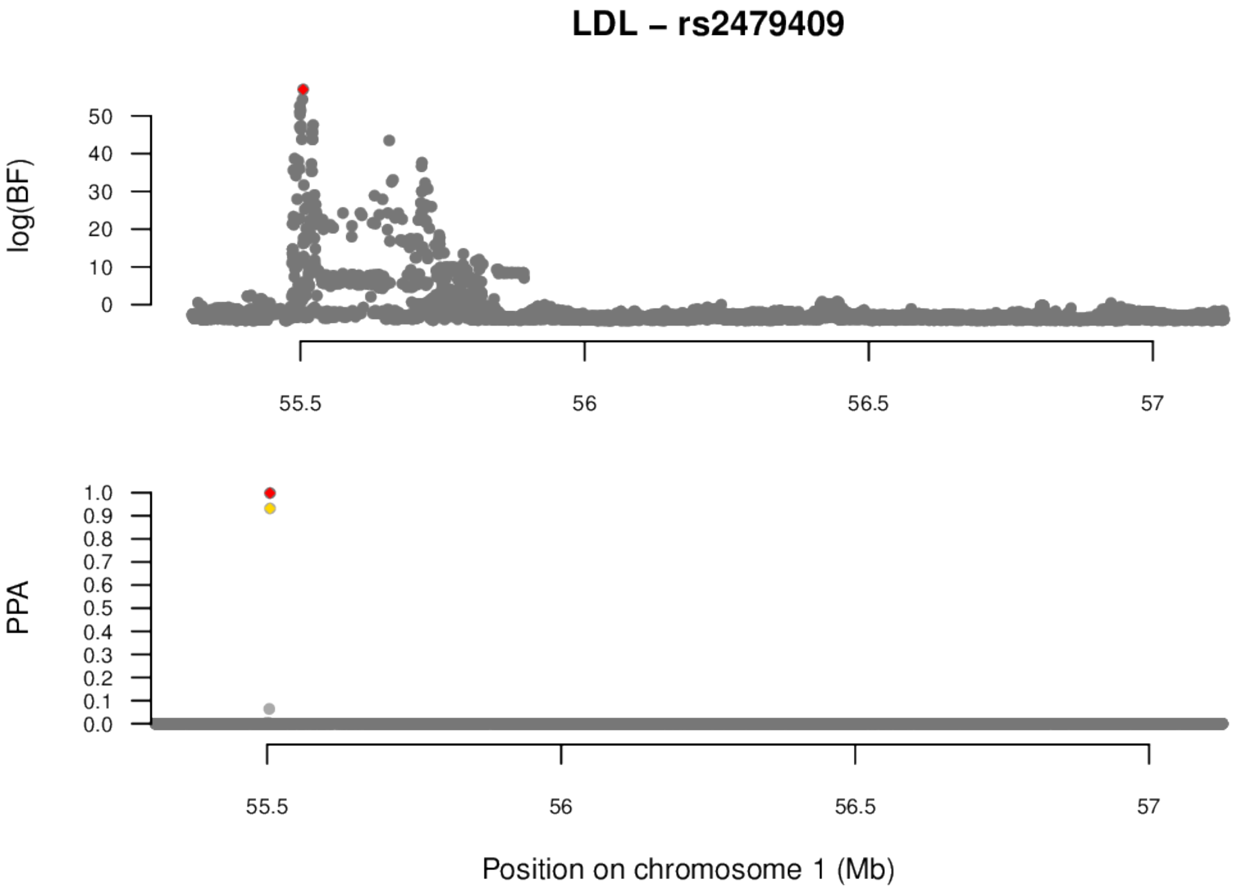

LDL – rs2479409
log(BF)
50
40
30
20
10
0
55.5
56
56.5
57
PPA
1.0
0.9
0.8
0.7
0.6
0.5
0.4
0.3
0.2
0.1
0.0
55.5
56
56.5
57
Position on chromosome 1 (Mb)

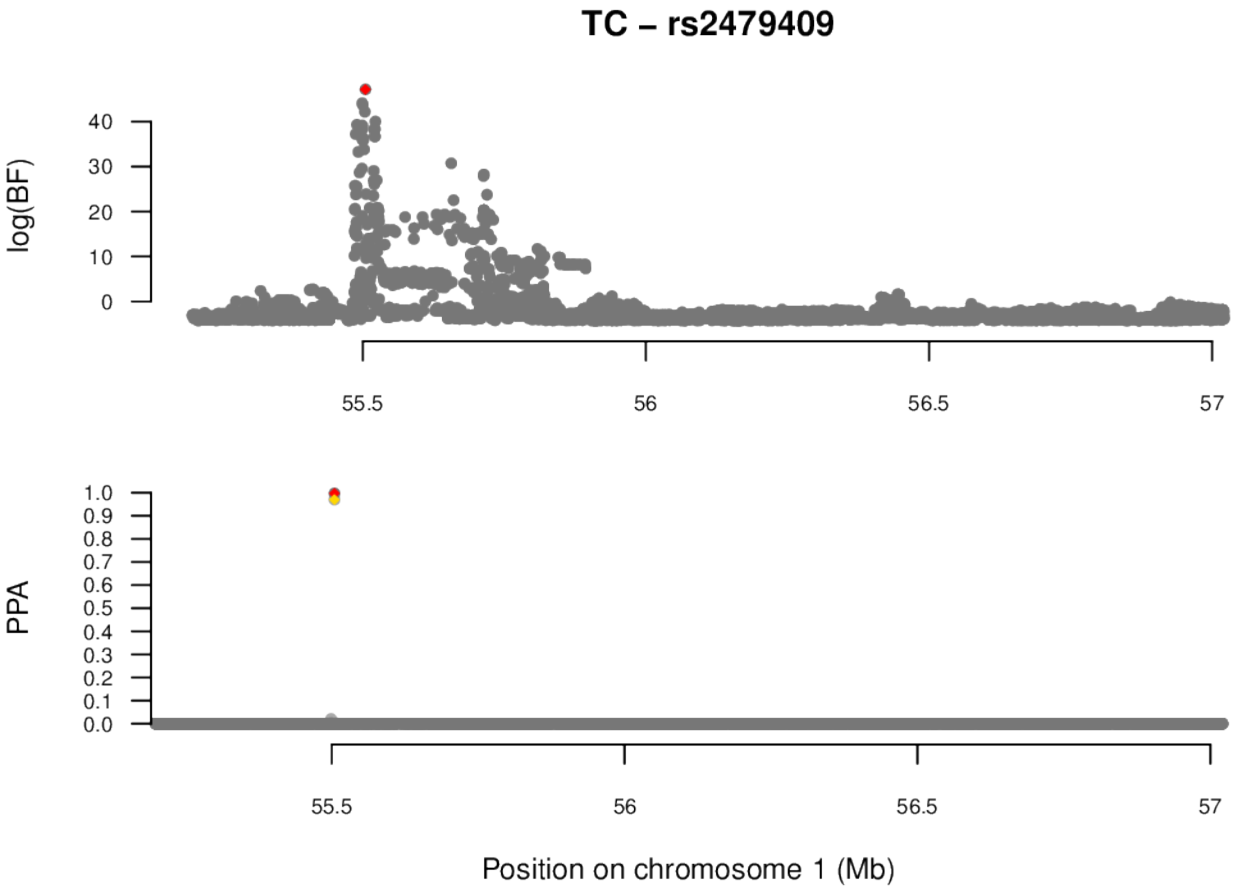
TC – rs2479409
log(BF)
40
30
20
10
0
55.5
56
56.5
57
PPA
1.0
0.9
0.8
0.7
0.6
0.5
0.4
0.3
0.2
0.1
0.0
55.5
56
56.5
57
Position on chromosome 1 (Mb)

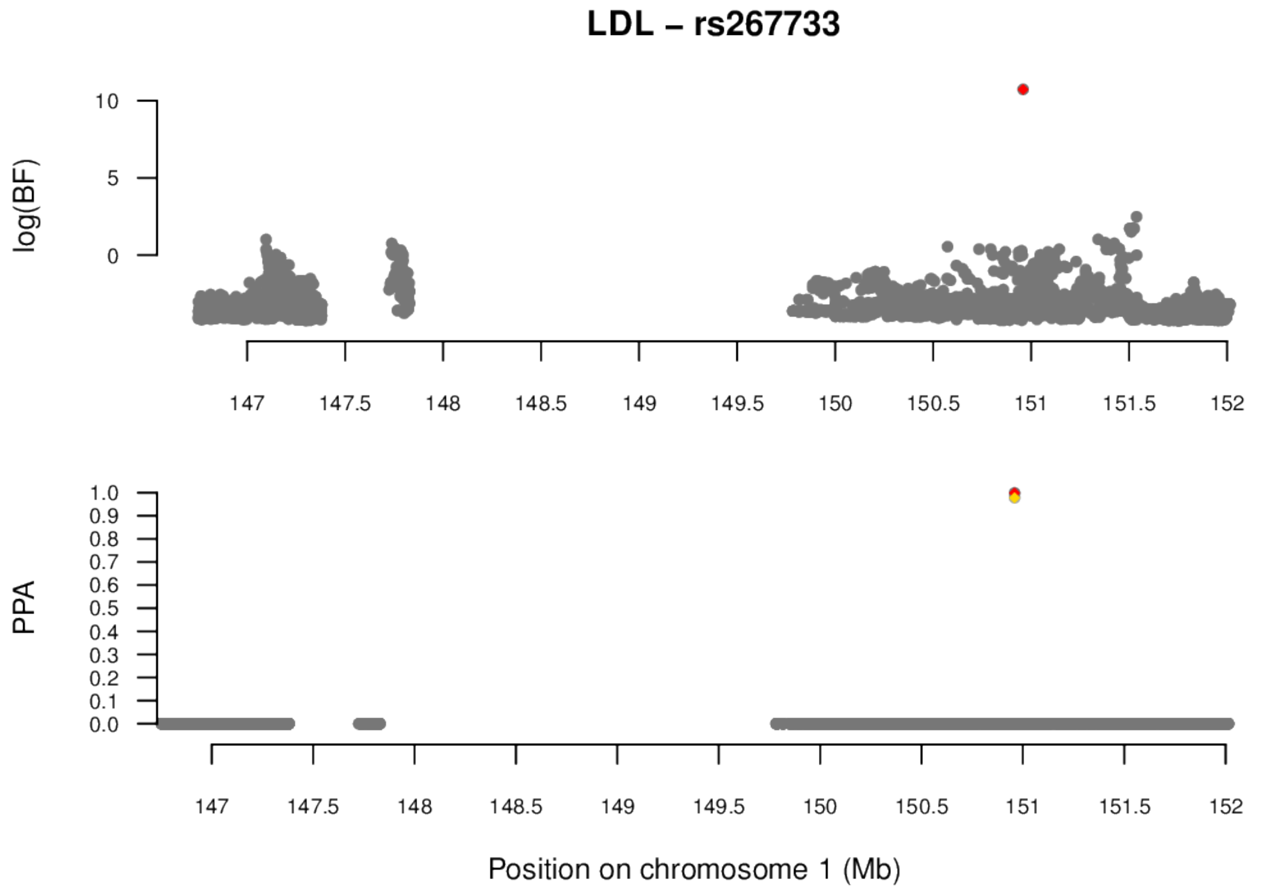

LDL – rs267733
log(BF)
10
5
0
147
147.5
148
148.5
149
149.5
150
150.5
151
151.5
152
PPA
1.0
0.9
0.8
0.7
0.6
0.5
0.4
0.3
0.2
0.1
0.0
147
147.5
148
148.5
149
149.5
150
150.5
151
151.5
152
Position on chromosome 1 (Mb)

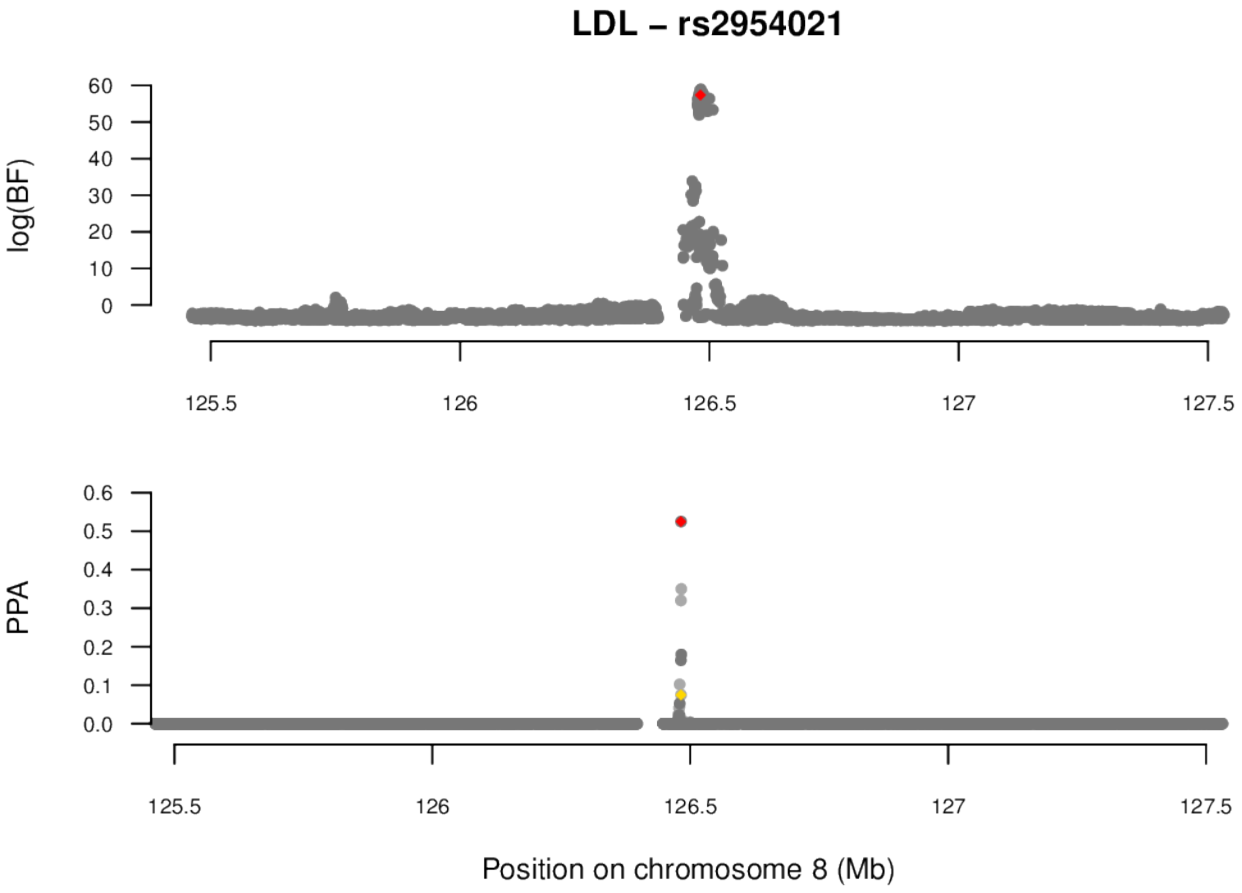

LDL – rs2954021
log(BF)
60
50
40
30
20
10
0
125.5
126
126.5
127
127.5
PPA
0.6
0.5
0.4
0.3
0.2
0.1
0.0
125.5
126
126.5
127
127.5
Position on chromosome 8 (Mb)

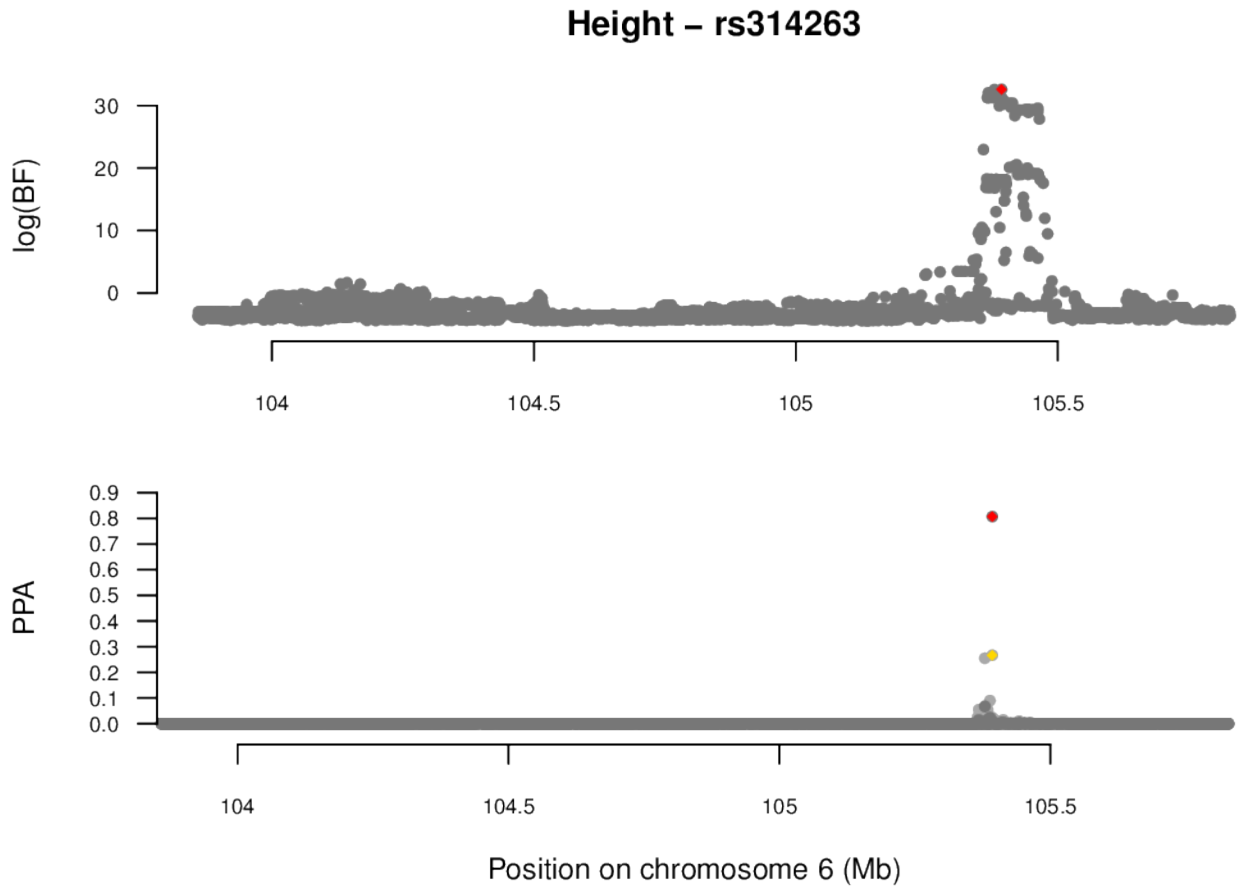

Height – rs314263
log(BF)
30
20
10
0
104
104.5
105
105.5
PPA
0.9
0.8
0.7
0.6
0.5
0.4
0.3
0.2
0.1
0.0
104
104.5
105
105.5
Position on chromosome 6 (Mb)

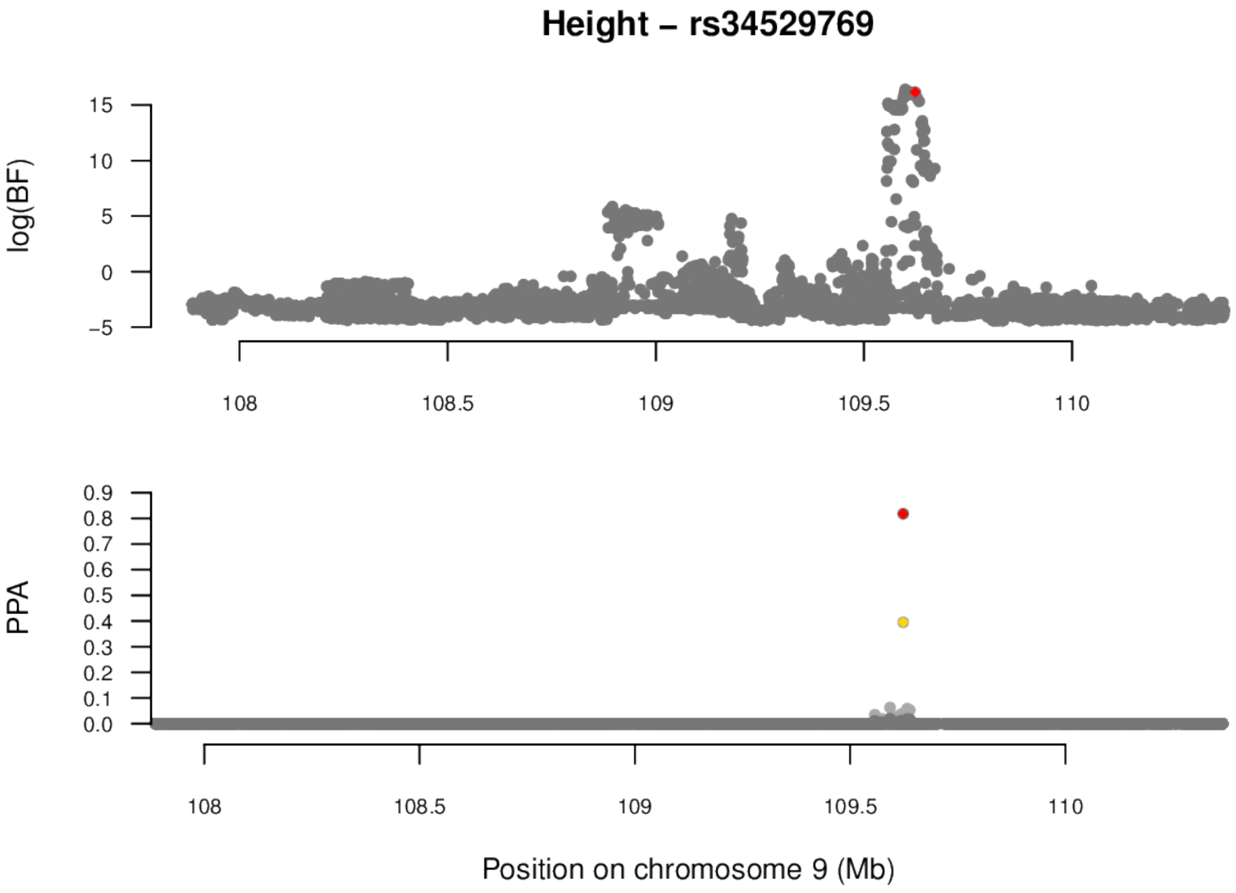
Height – rs34529769
log(BF)
15
10
5
0
−5
108
108.5
109
109.5
110
PPA
0.9
0.8
0.7
0.6
0.5
0.4
0.3
0.2
0.1
0.0
108
108.5
109
109.5
110
Position on chromosome 9 (Mb)

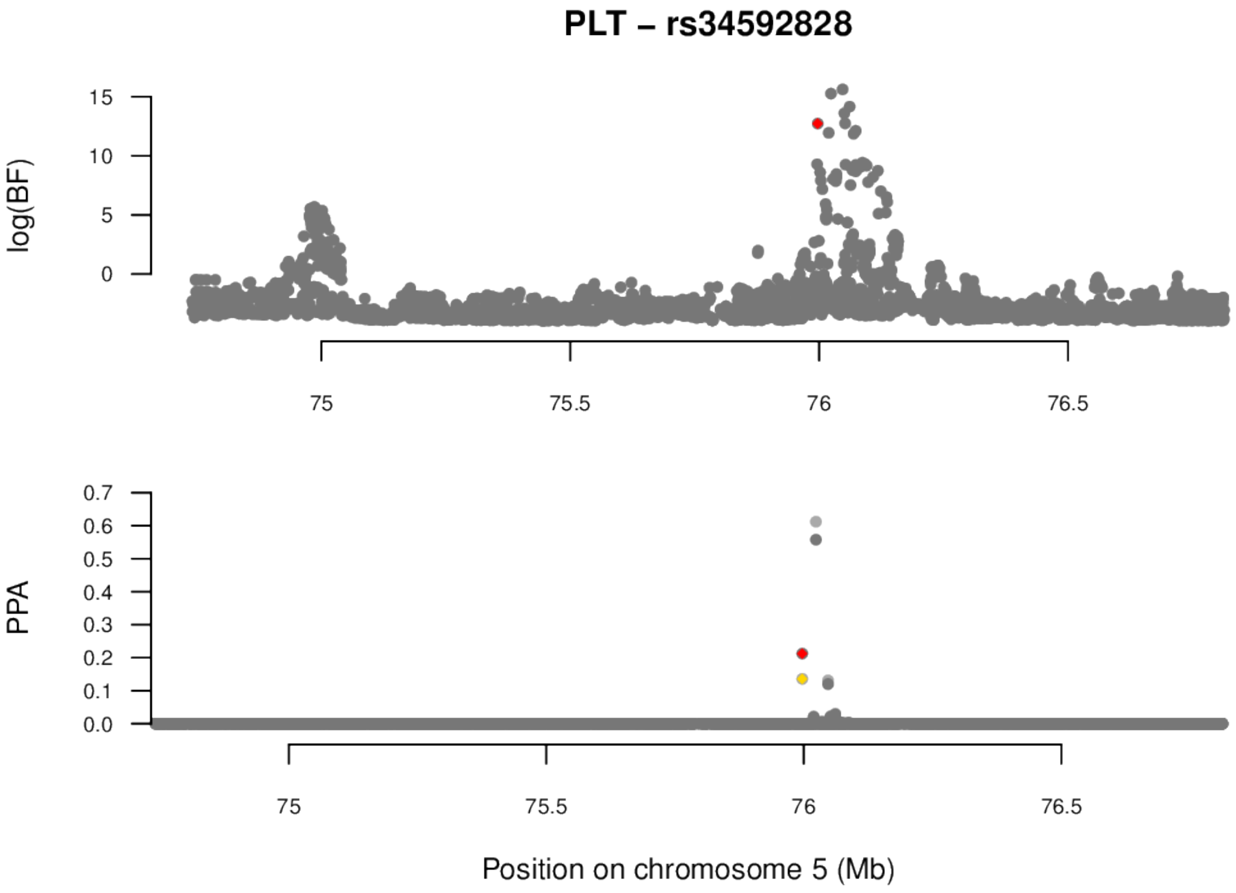
PLT – rs34592828
log(BF)
15
10
5
0
75
75.5
76
76.5
PPA
0.7
0.6
0.5
0.4
0.3
0.2
0.1
0.0
75
75.5
76
76.5
Position on chromosome 5 (Mb)

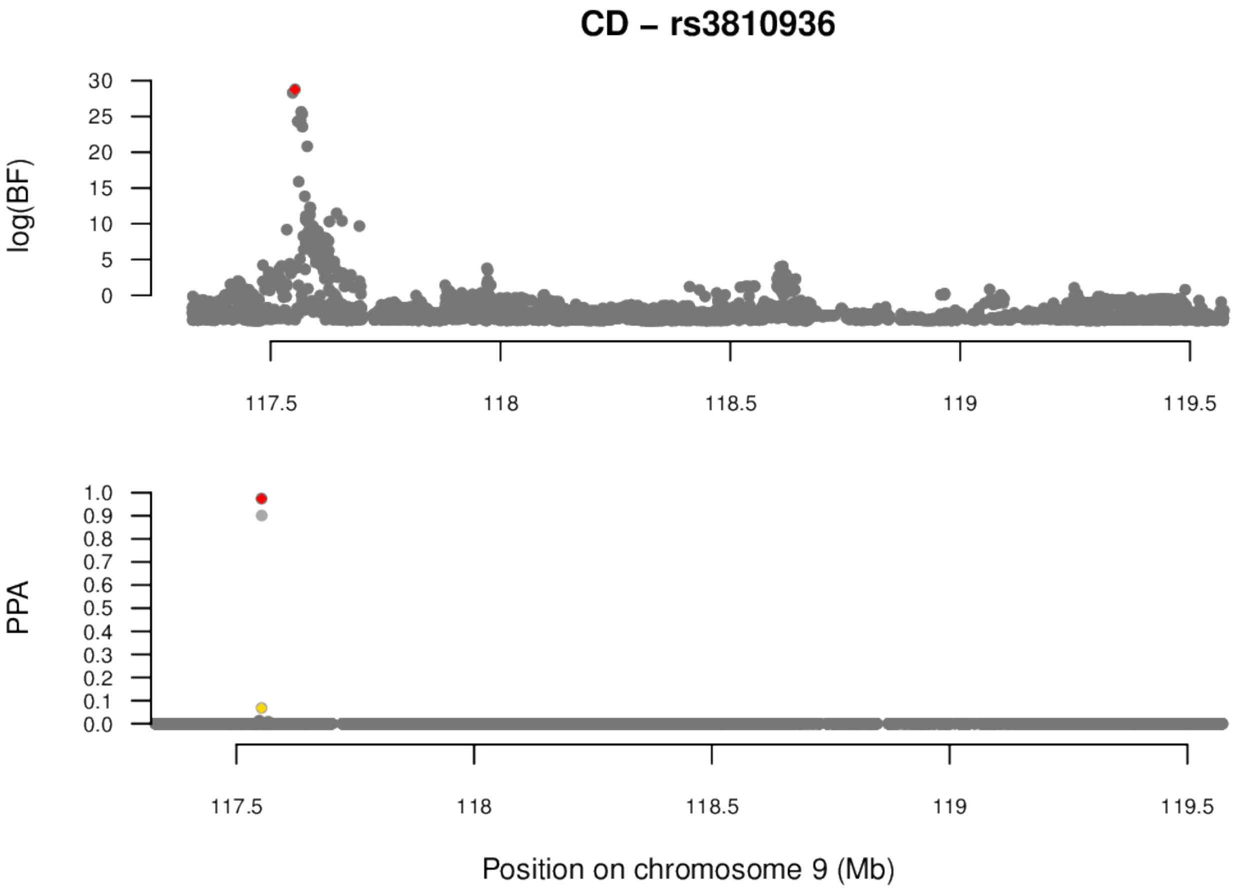

CD – rs3810936
log(BF)
30
25
20
15
10
5
0
117.5
118
118.5
119
119.5
PPA
1.0
0.9
0.8
0.7
0.6
0.5
0.4
0.3
0.2
0.1
0.0
117.5
118
118.5
119
119.5
Position on chromosome 9 (Mb)

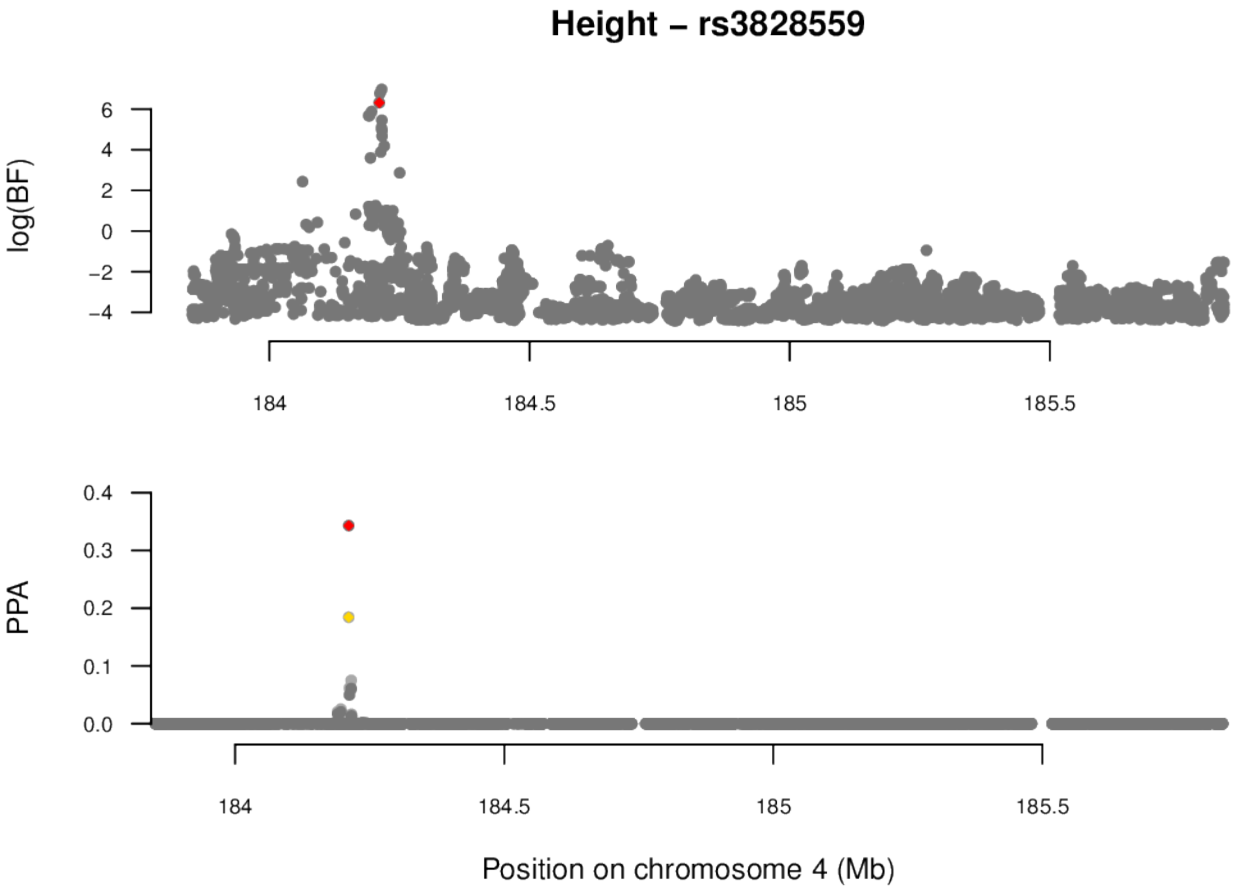
Height – rs3828559
log(BF)
6
4
2
0
−2
−4
184
184.5
185
185.5
PPA
0.4
0.3
0.2
0.1
0.0
184
184.5
185
185.5
Position on chromosome 4 (Mb)

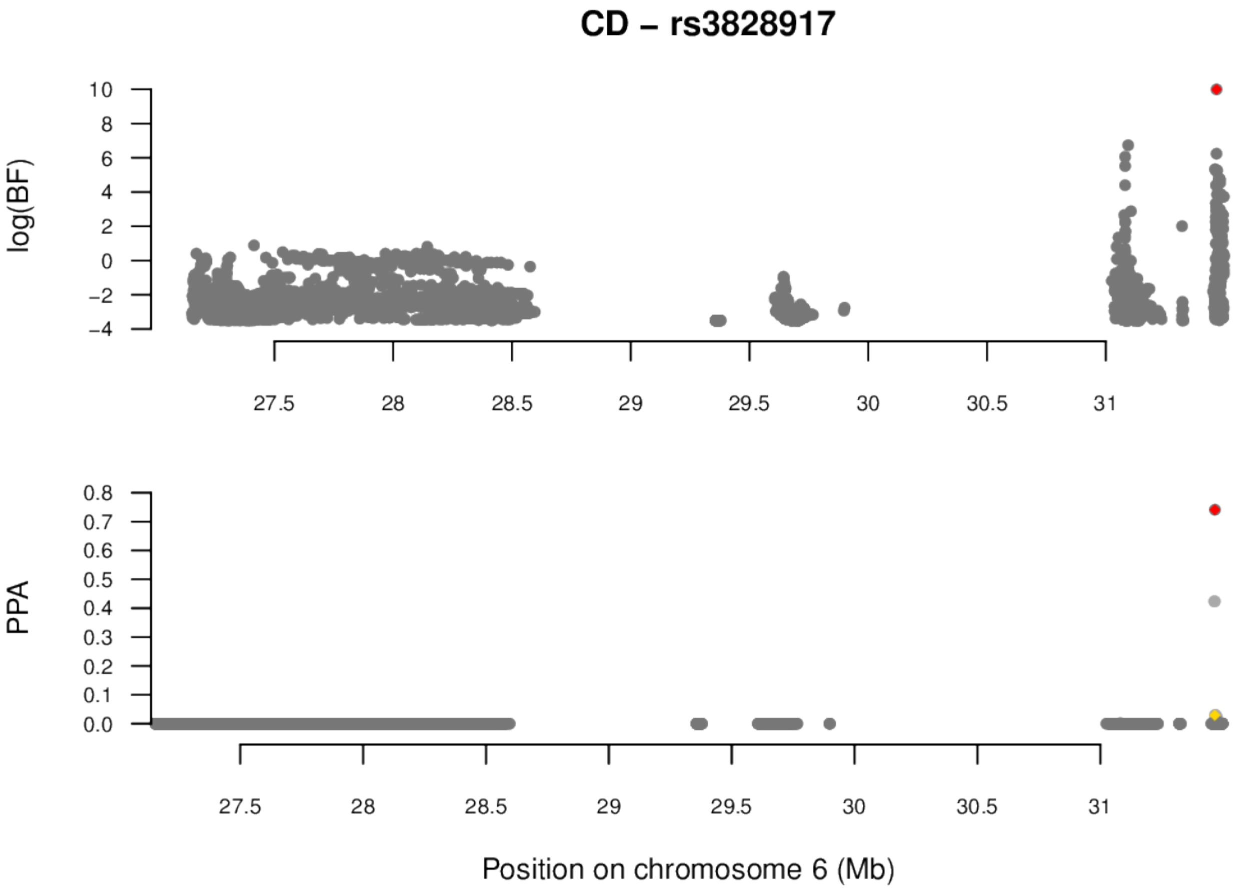
CD – rs3828917
log(BF)
10
8
6
4
2
0
−2
−4
27.5
28
28.5
29
29.5
30
30.5
31
PPA
0.8
0.7
0.6
0.5
0.4
0.3
0.2
0.1
0.0
27.5
28
28.5
29
29.5
30
30.5
31
Position on chromosome 6 (Mb)

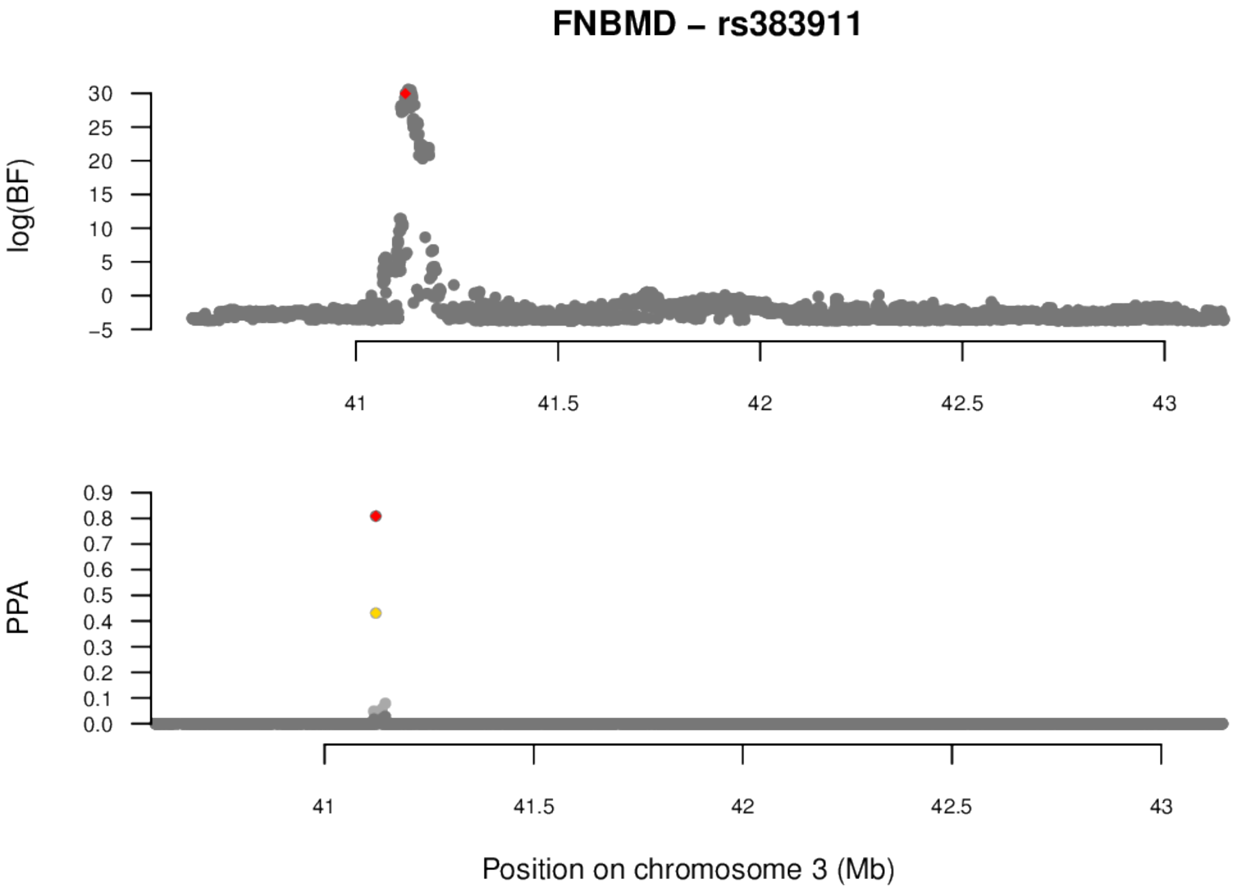
FNBMD – rs383911
log(BF)
30
25
20
15
10
5
0
–5
41
41.5
42
42.5
43
PPA
0.9
0.8
0.7
0.6
0.5
0.4
0.3
0.2
0.1
0.0
41
41.5
42
42.5
43
Position on chromosome 3 (Mb)

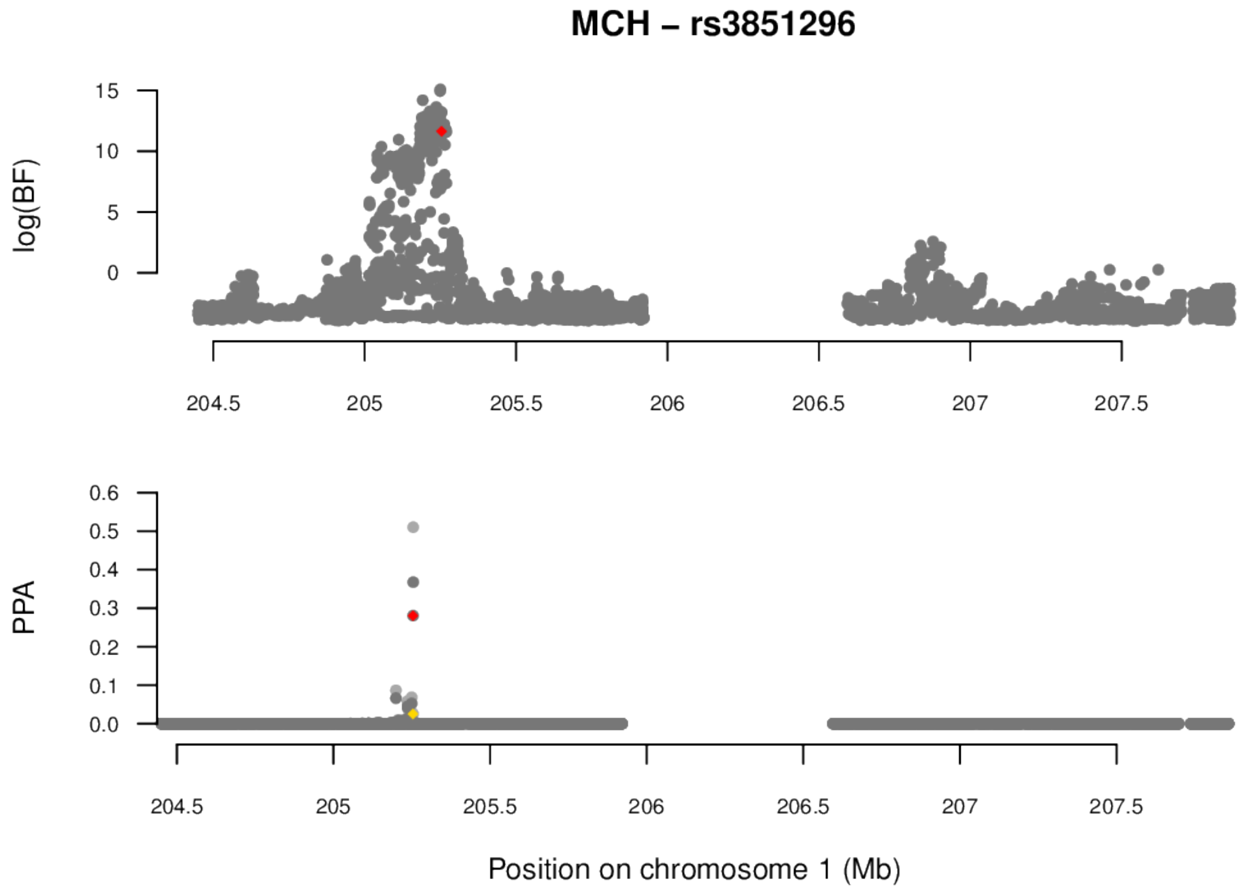

MCH – rs3851296
log(BF)
15
10
5
0
204.5
205
205.5
206
206.5
207
207.5
PPA
0.6
0.5
0.4
0.3
0.2
0.1
0.0
204.5
205
205.5
206
206.5
207
207.5
Position on chromosome 1 (Mb)

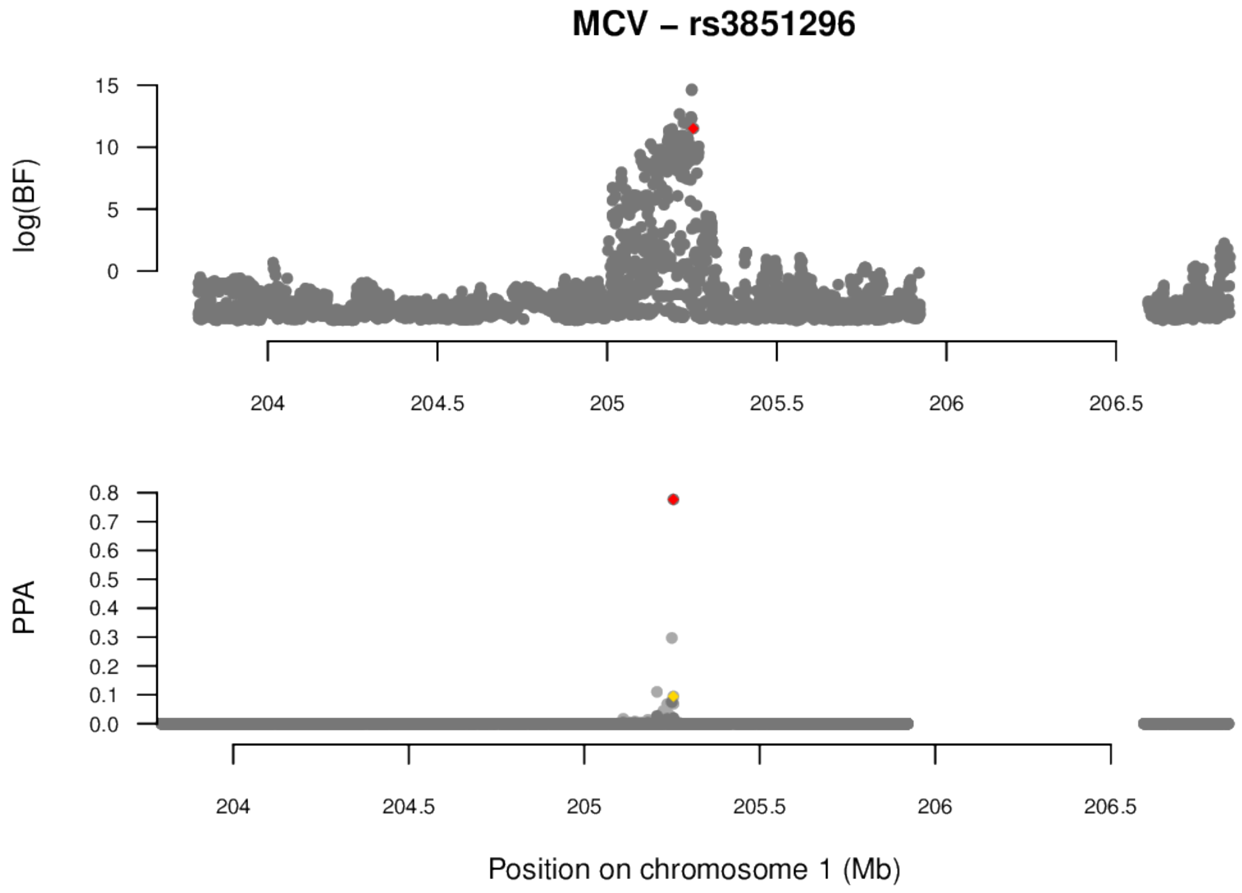

MCV – rs3851296
log(BF)
15
10
5
0
204
204.5
205
205.5
206
206.5
PPA
0.8
0.7
0.6
0.5
0.4
0.3
0.2
0.1
0.0
204
204.5
205
205.5
206
206.5
Position on chromosome 1 (Mb)

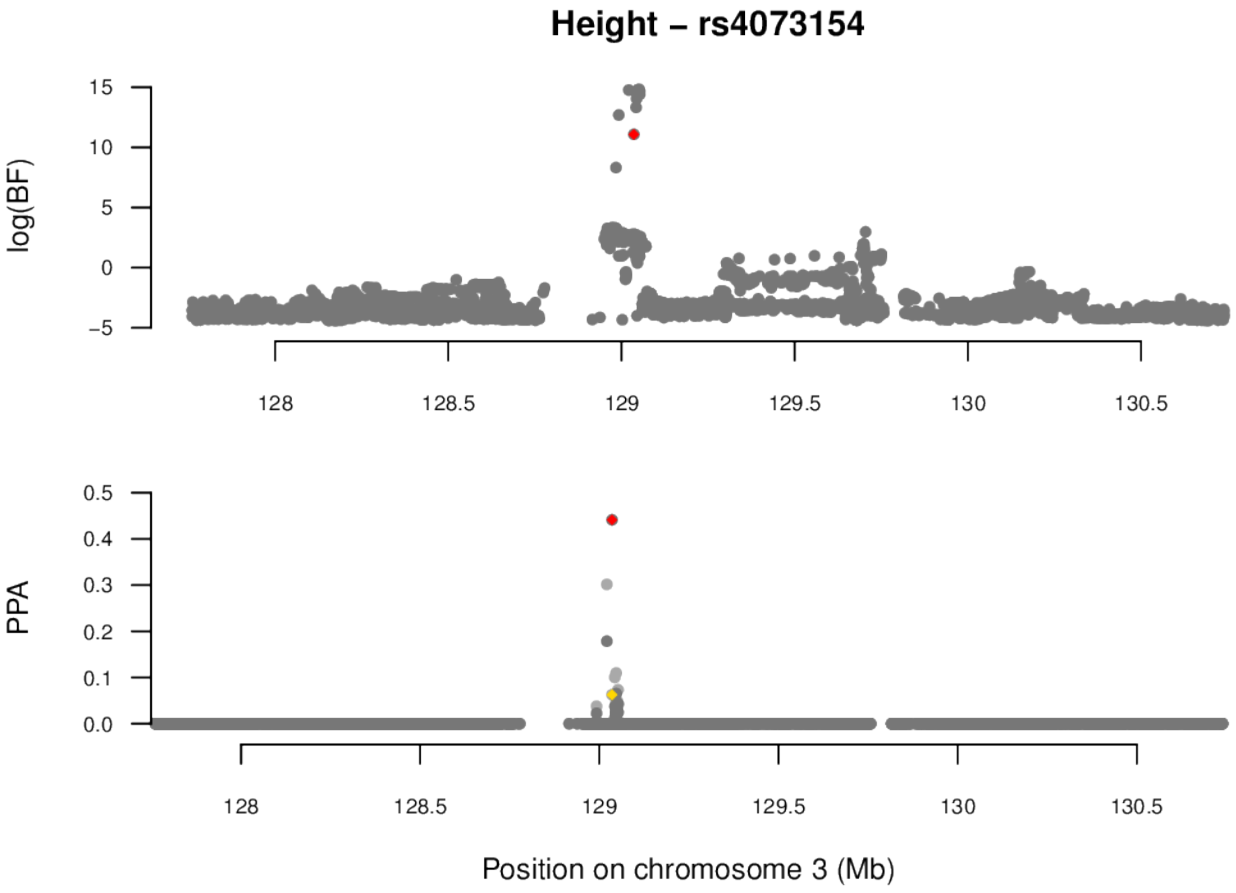

Height – rs4073154
log(BF)
15
10
5
0
−5
128
128.5
129
129.5
130
130.5
PPA
0.5
0.4
0.3
0.2
0.1
0.0
128
128.5
129
129.5
130
130.5
Position on chromosome 3 (Mb)

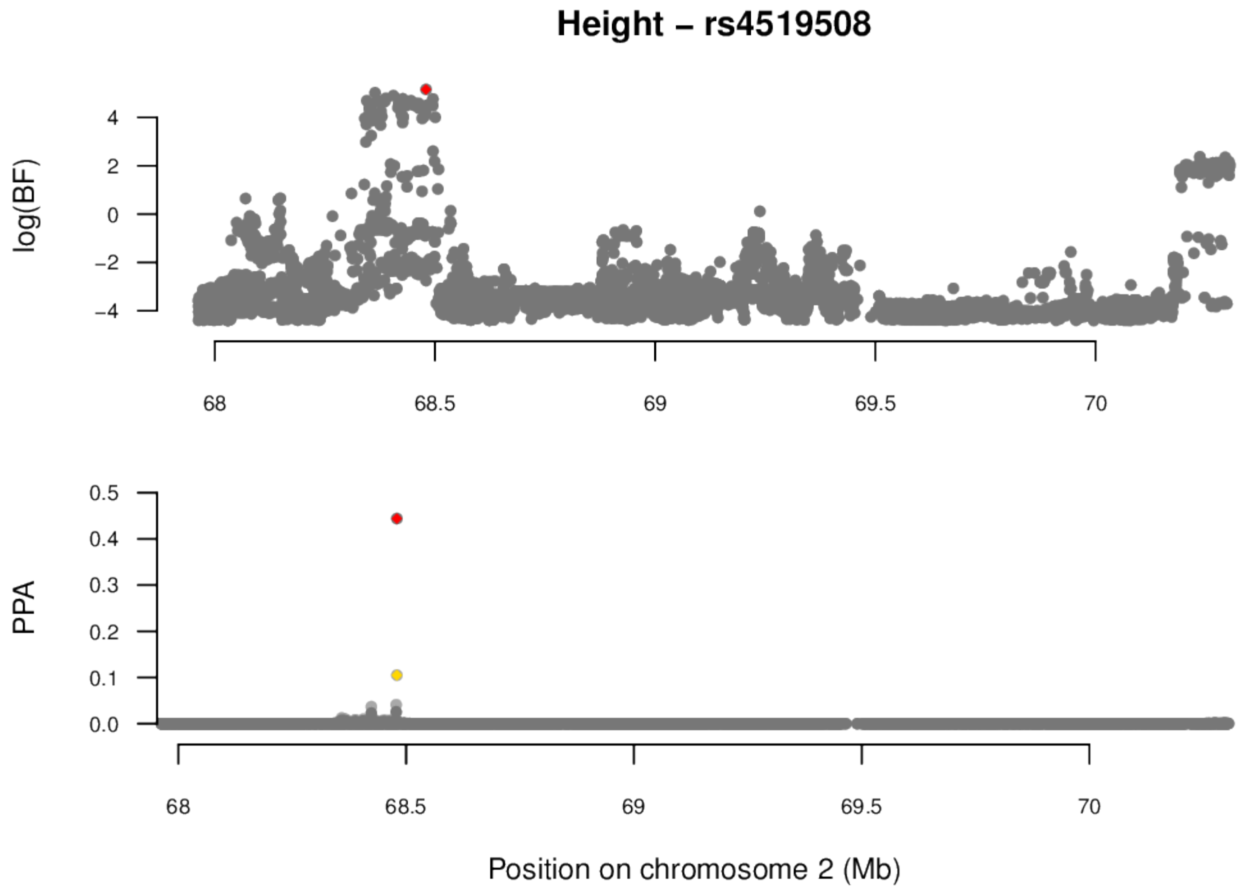

Height – rs4519508
log(BF)
4
2
0
−2
−4
68
68.5
69
69.5
70
PPA
0.5
0.4
0.3
0.2
0.1
0.0
68
68.5
69
69.5
70
Position on chromosome 2 (Mb)

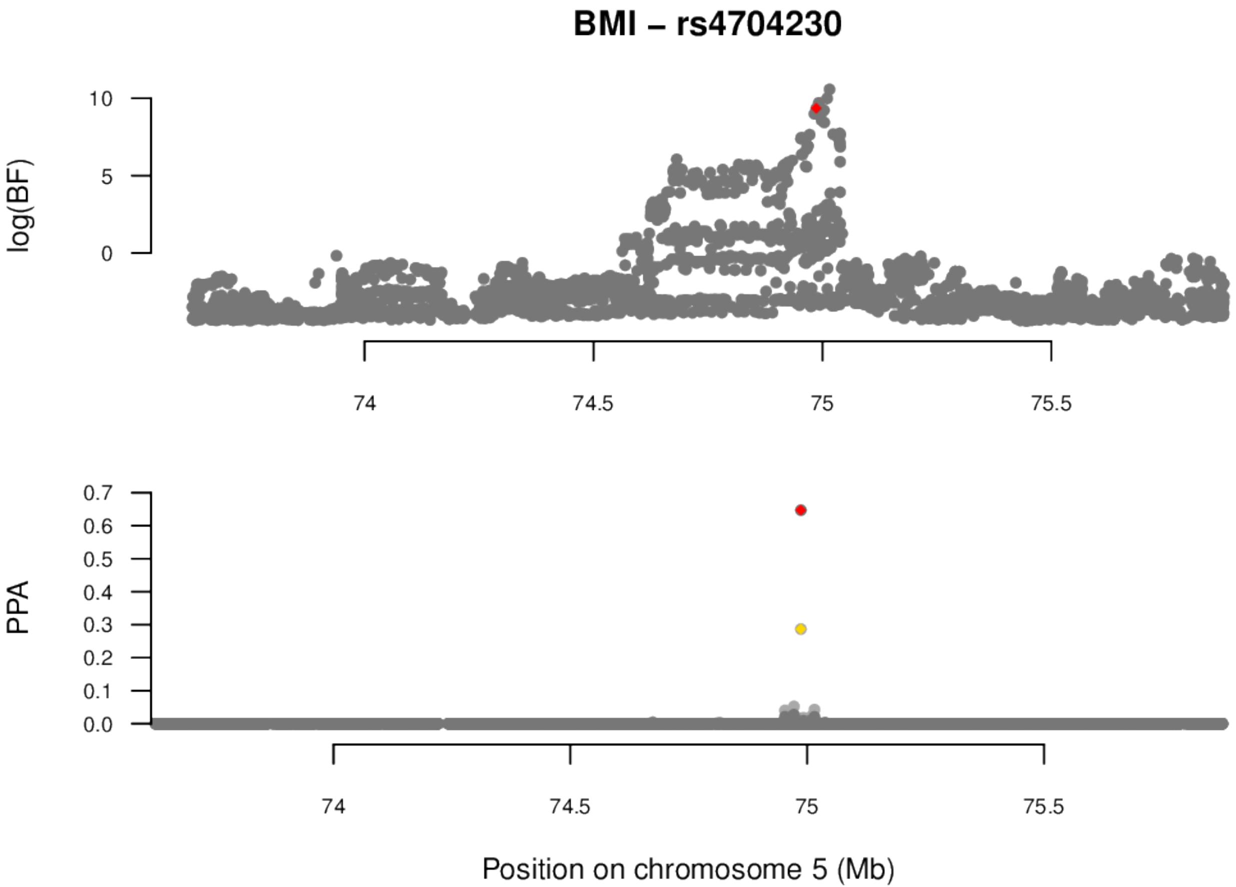

BMI – rs4704230
log(BF)
10
5
0
74
74.5
75
75.5
PPA
0.7
0.6
0.5
0.4
0.3
0.2
0.1
0.0
74
74.5
75
75.5
Position on chromosome 5 (Mb)

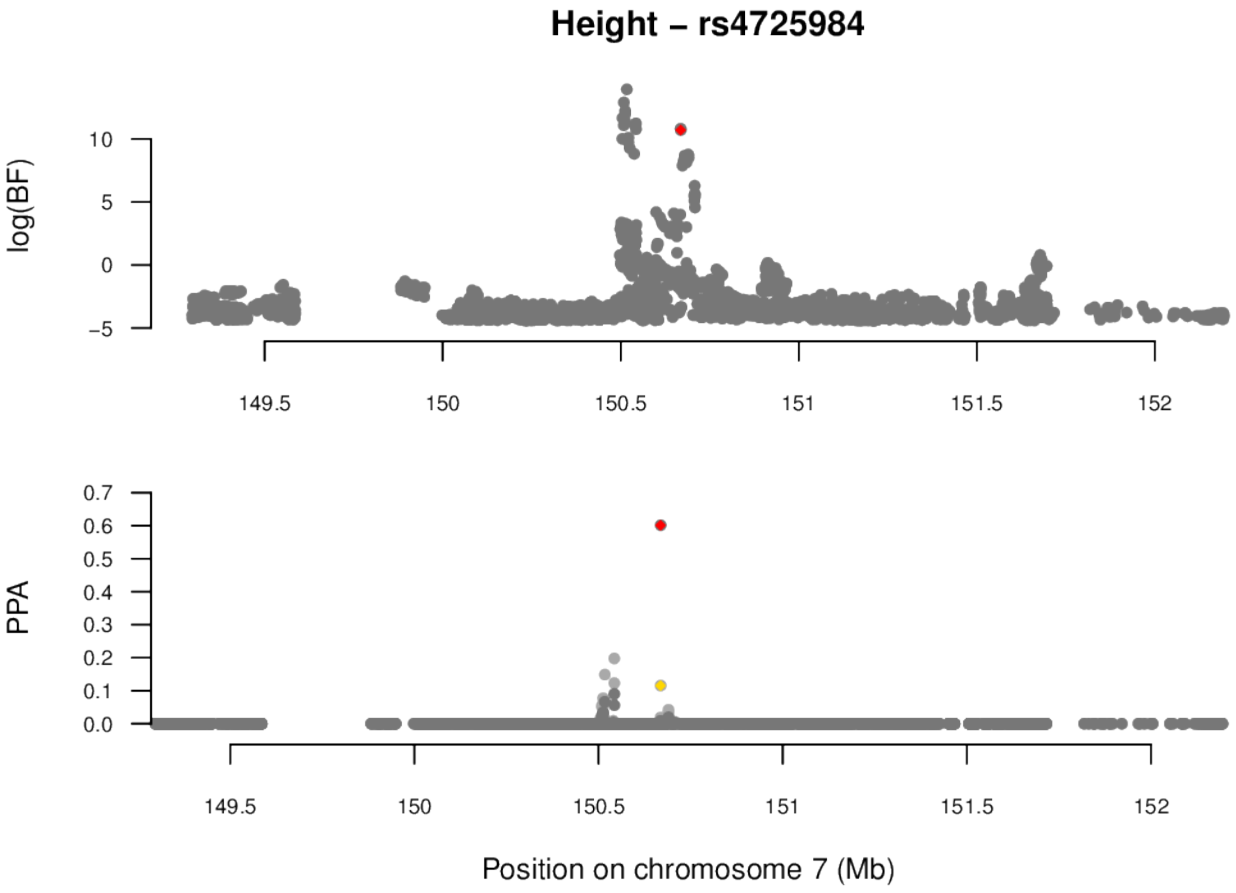
Height – rs4725984
log(BF)
10
5
0
−5
149.5
150
150.5
151
151.5
152
PPA
0.7
0.6
0.5
0.4
0.3
0.2
0.1
0.0
149.5
150
150.5
151
151.5
152
Position on chromosome 7 (Mb)

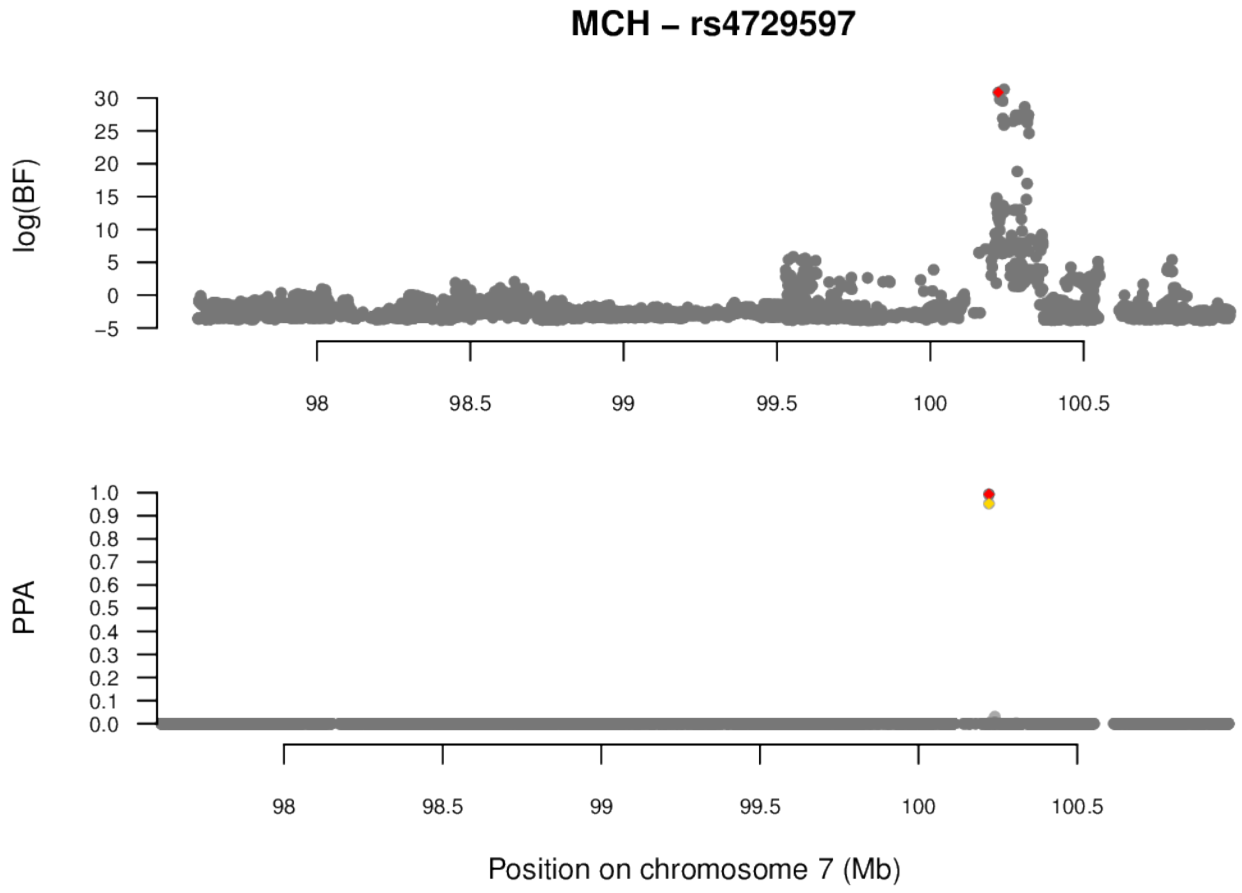

MCH – rs4729597
log(BF)
30
25
20
15
10
5
0
−5
98
98.5
99
99.5
100
100.5
PPA
1.0
0.9
0.8
0.7
0.6
0.5
0.4
0.3
0.2
0.1
0.0
98
98.5
99
99.5
100
100.5
Position on chromosome 7 (Mb)

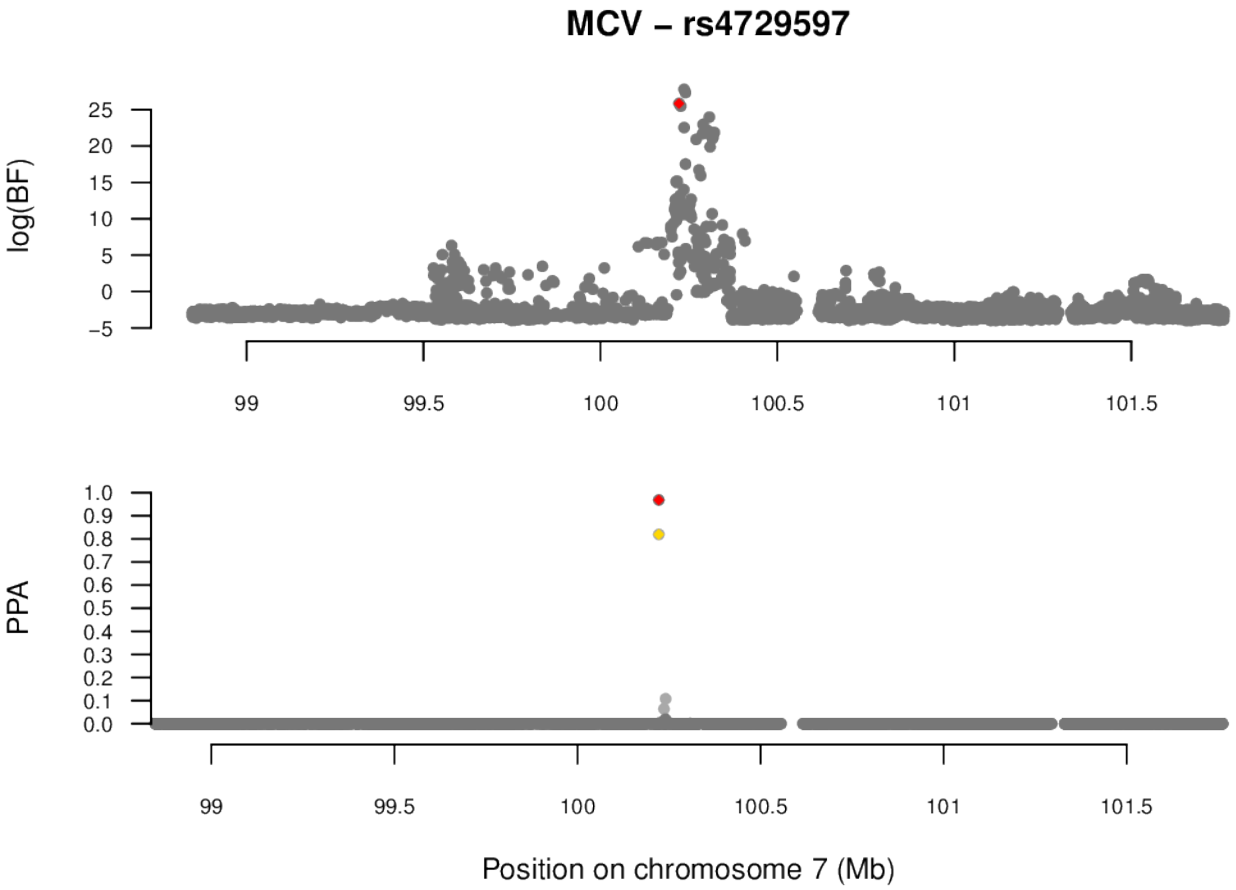

MCV – rs4729597
log(BF)
25
20
15
10
5
0
−5
99
99.5
100
100.5
101
101.5
PPA
1.0
0.9
0.8
0.7
0.6
0.5
0.4
0.3
0.2
0.1
0.0
99
99.5
100
100.5
101
101.5
Position on chromosome 7 (Mb)

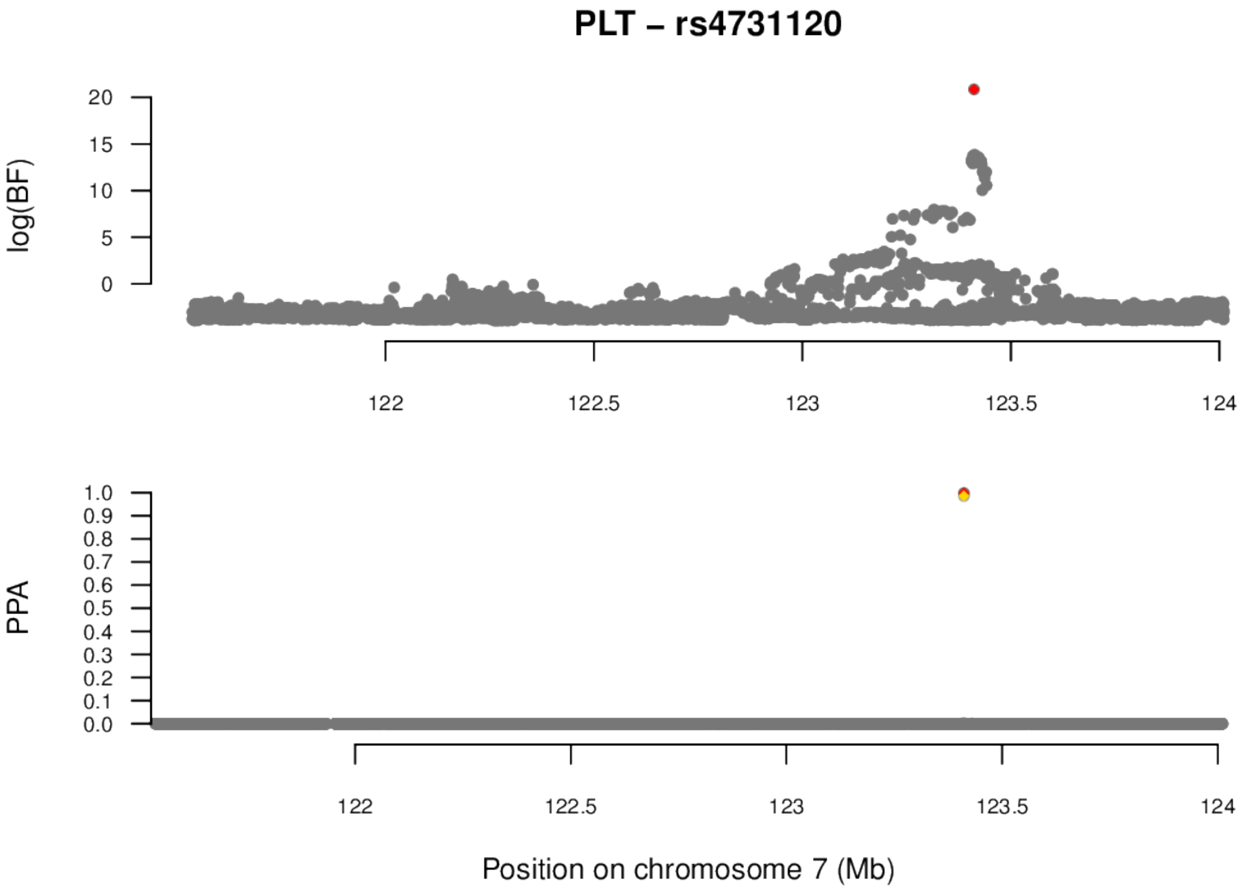
PLT – rs4731120
log(BF)
20
15
10
5
0
122
122.5
123
123.5
124
PPA
1.0
0.9
0.8
0.7
0.6
0.5
0.4
0.3
0.2
0.1
0.0
122
122.5
123
123.5
124
Position on chromosome 7 (Mb)

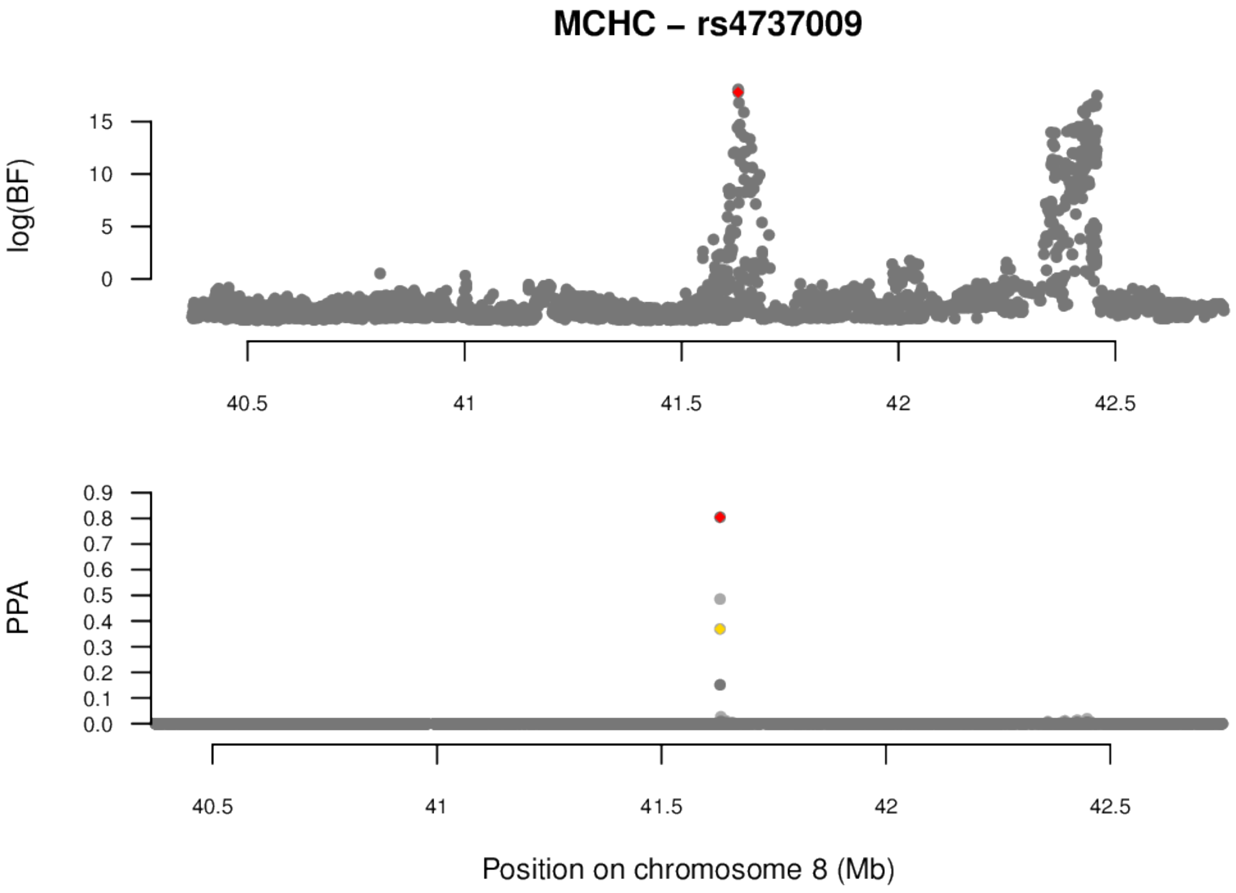
MCHC – rs4737009
log(BF)
15
10
5
0
40.5
41
41.5
42
42.5
PPA
0.9
0.8
0.7
0.6
0.5
0.4
0.3
0.2
0.1
0.0
40.5
41
41.5
42
42.5
Position on chromosome 8 (Mb)

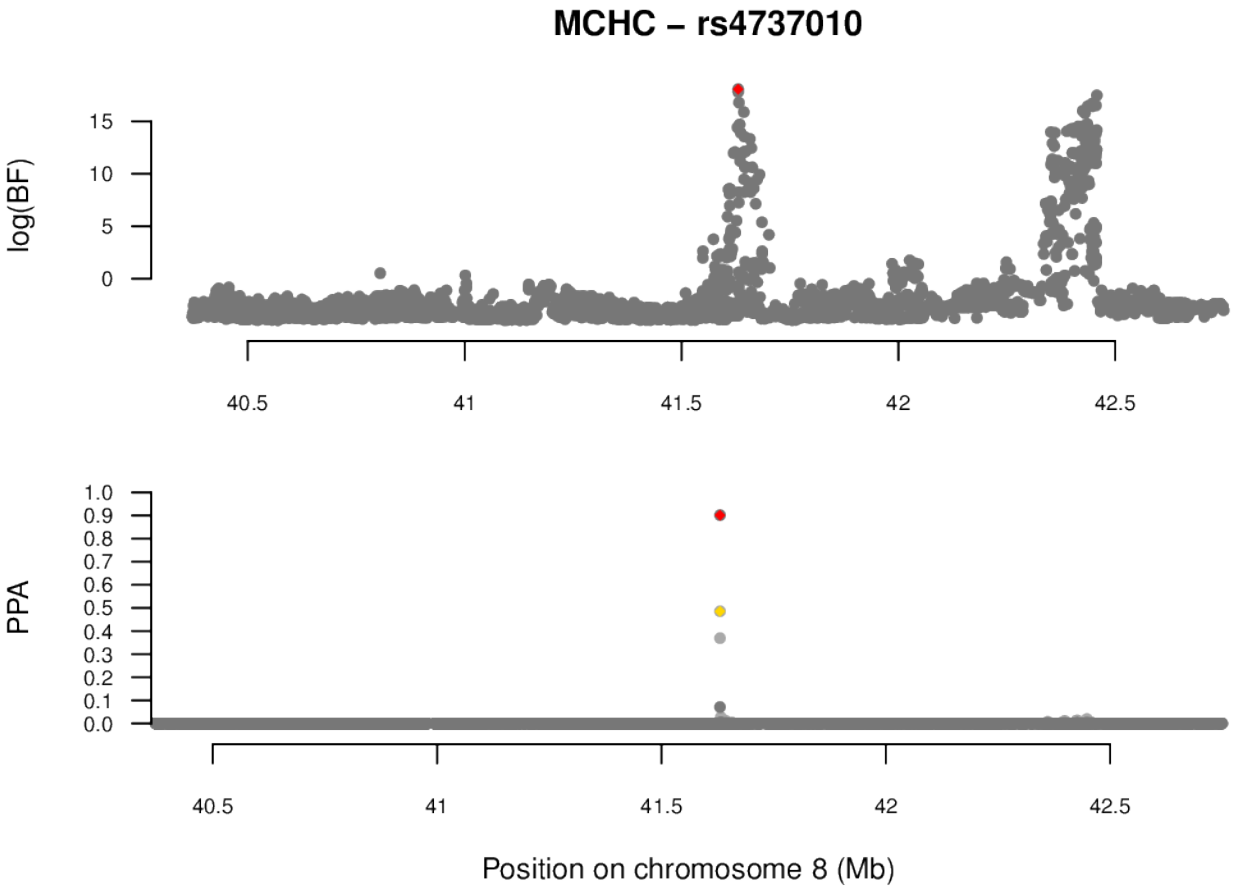

MCHC – rs4737010
log(BF)
15
10
5
0
40.5
41
41.5
42
42.5
PPA
1.0
0.9
0.8
0.7
0.6
0.5
0.4
0.3
0.2
0.1
0.0
40.5
41
41.5
42
42.5
Position on chromosome 8 (Mb)

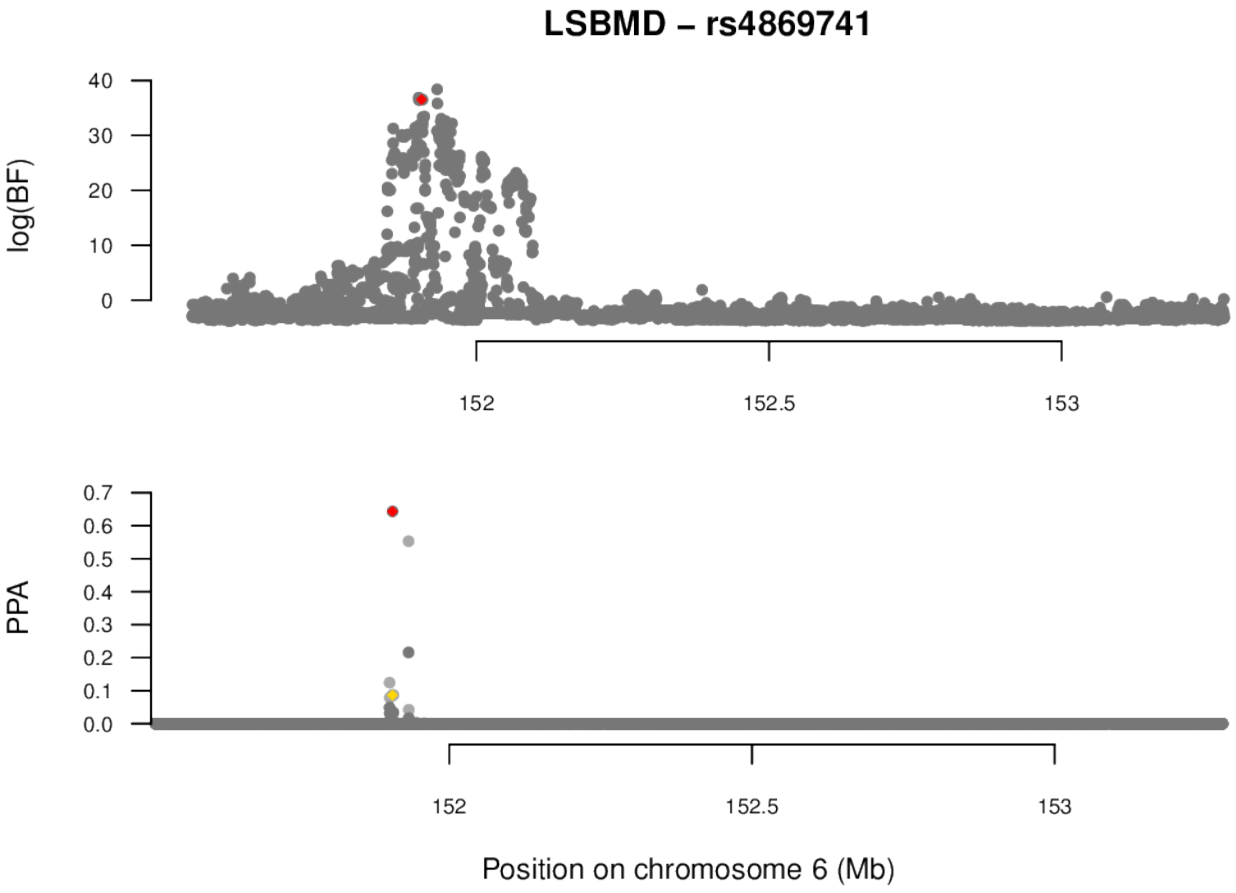
LSBMD – rs4869741
log(BF)
40
30
20
10
0
152
152.5
153
PPA
0.7
0.6
0.5
0.4
0.3
0.2
0.1
0.0
152
152.5
153
Position on chromosome 6 (Mb)

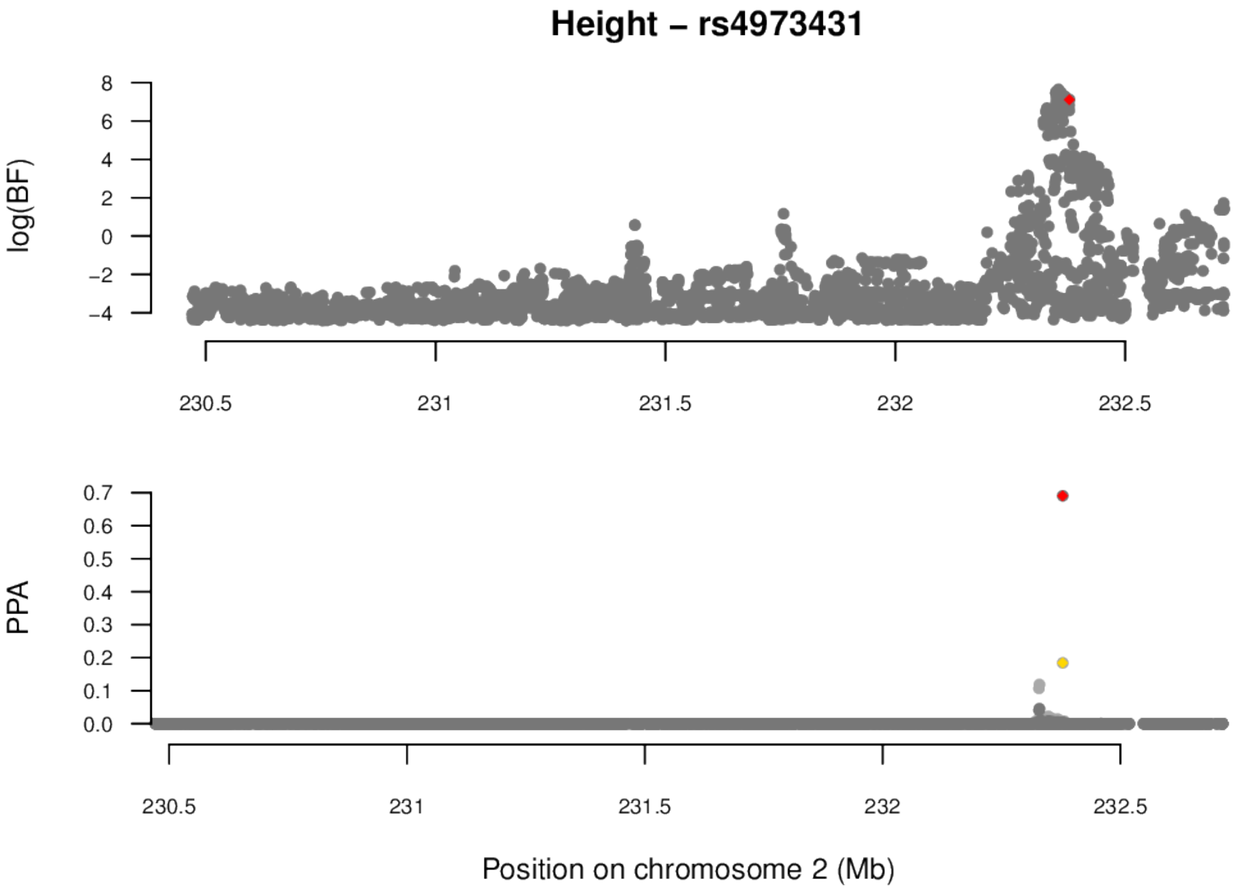
Height – rs4973431
log(BF)
8
6
4
2
0
−2
−4
230.5
231
231.5
232
232.5
PPA
0.7
0.6
0.5
0.4
0.3
0.2
0.1
0.0
230.5
231
231.5
232
232.5
Position on chromosome 2 (Mb)

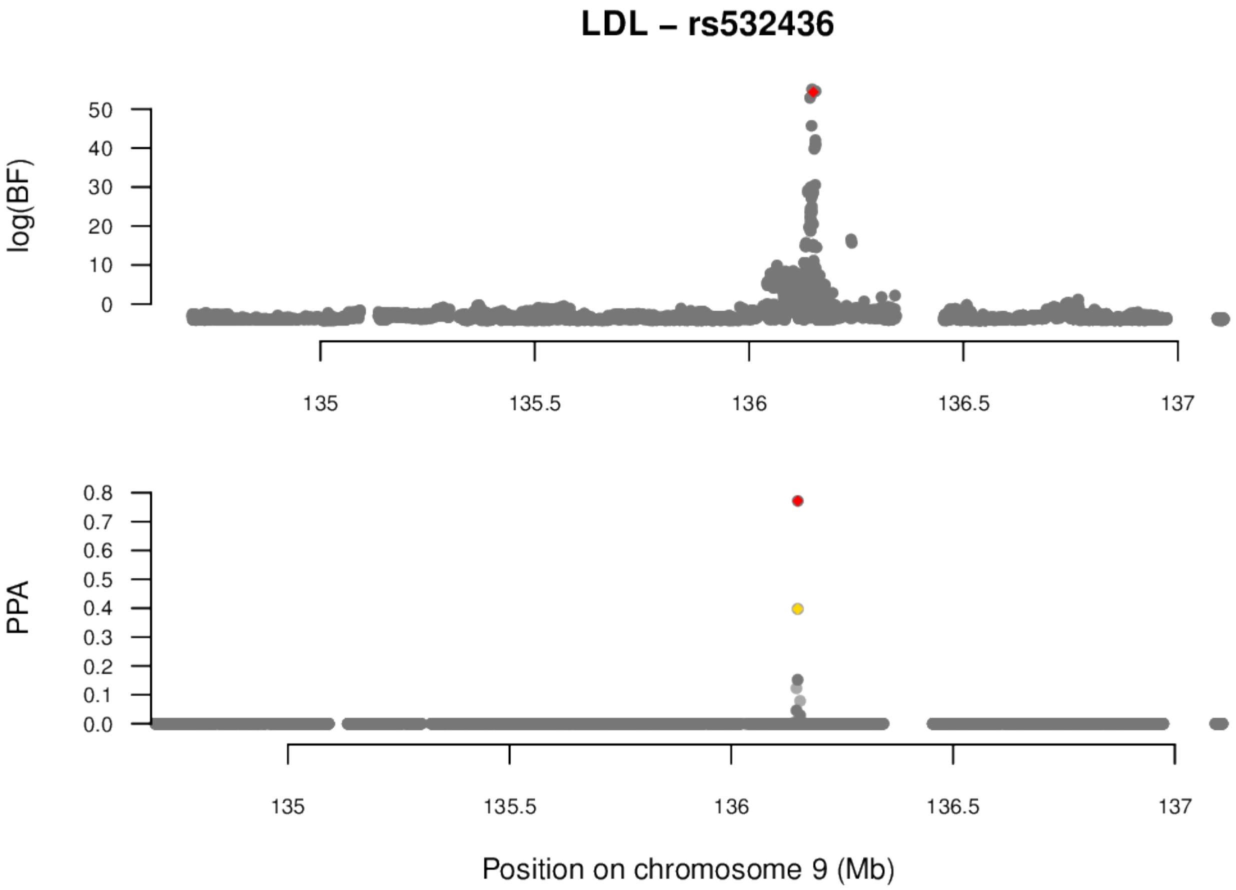

LDL – rs532436
log(BF)
50
40
30
20
10
0
135
135.5
136
136.5
137
PPA
0.8
0.7
0.6
0.5
0.4
0.3
0.2
0.1
0.0
135
135.5
136
136.5
137
Position on chromosome 9 (Mb)

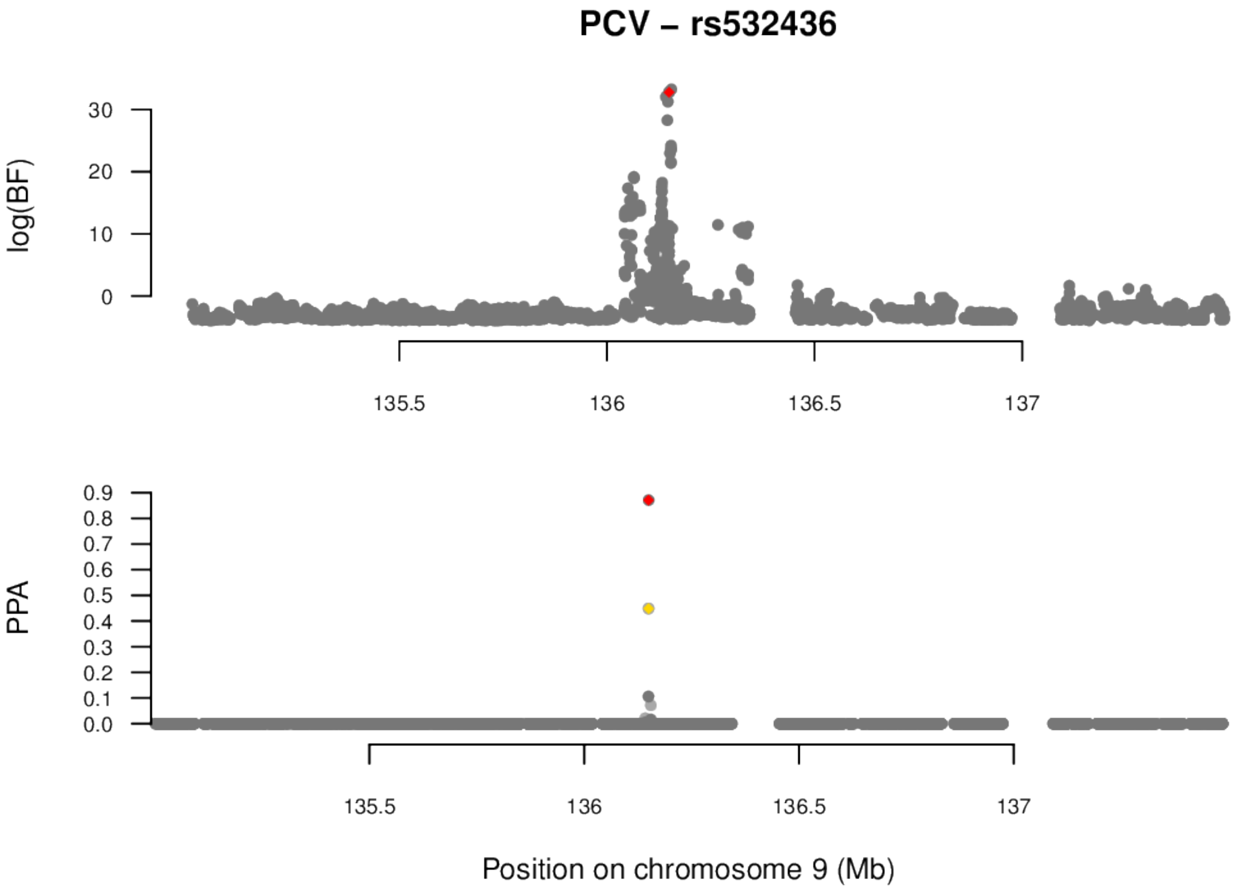

PCV – rs532436
log(BF)
30
20
10
0
135.5
136
136.5
137
PPA
0.9
0.8
0.7
0.6
0.5
0.4
0.3
0.2
0.1
0.0
135.5
136
136.5
137
Position on chromosome 9 (Mb)

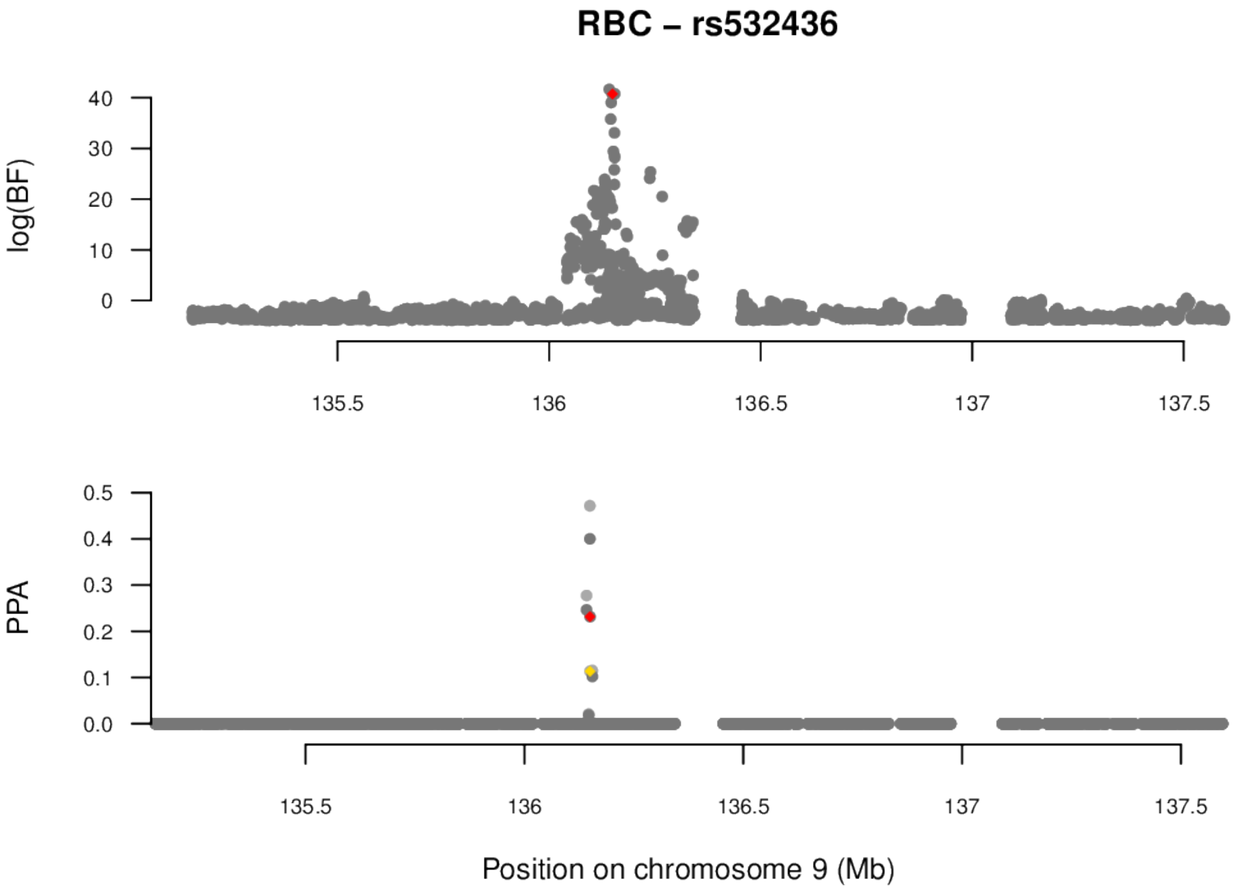
RBC – rs532436
log(BF)
40
30
20
10
0
135.5
136
136.5
137
137.5
PPA
0.5
0.4
0.3
0.2
0.1
0.0
135.5
136
136.5
137
137.5
Position on chromosome 9 (Mb)

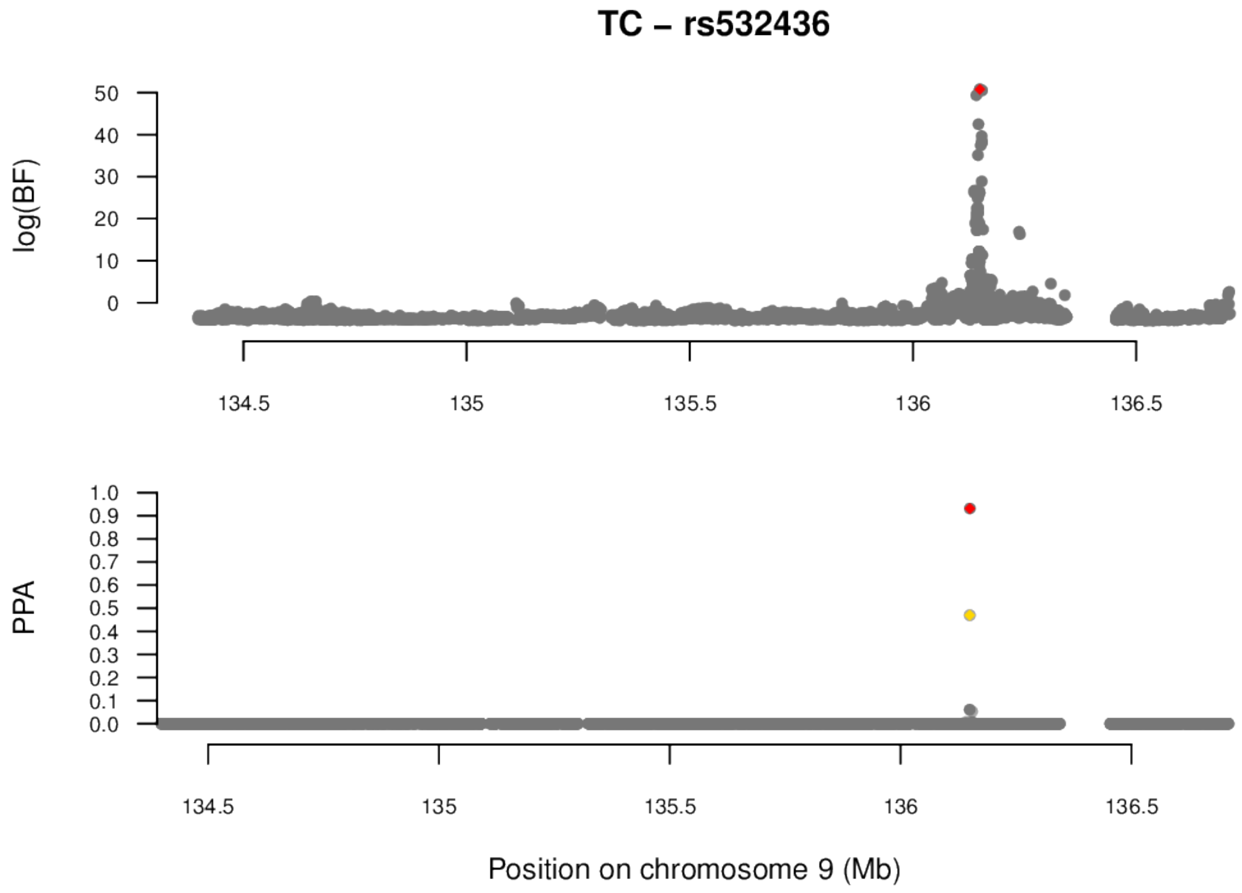
TC – rs532436
log(BF)
50
40
30
20
10
0
134.5
135
135.5
136
136.5
PPA
1.0
0.9
0.8
0.7
0.6
0.5
0.4
0.3
0.2
0.1
0.0
134.5
135
135.5
136
136.5
Position on chromosome 9 (Mb)

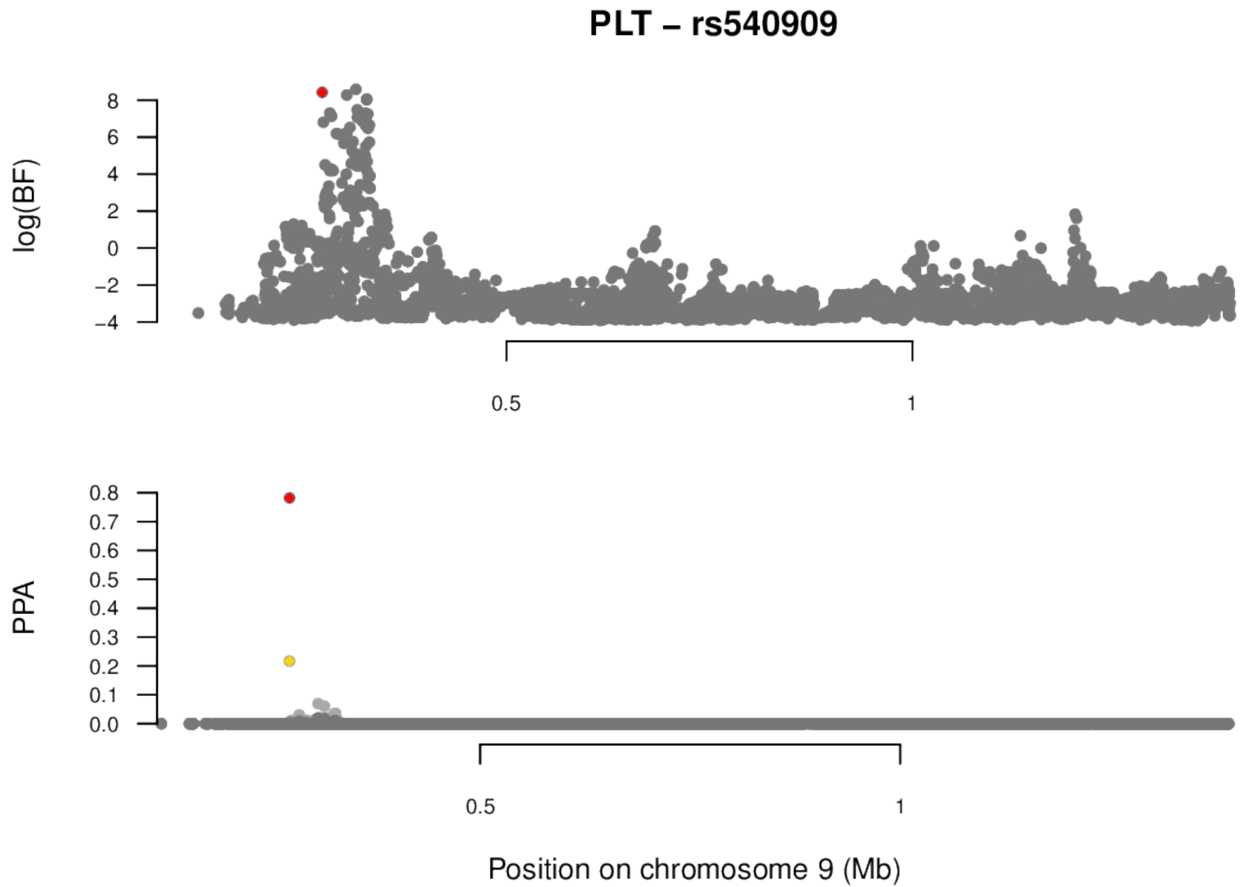
PLT – rs540909
log(BF)
8
6
4
2
0
−2
−4
0.5
1
PPA
0.8
0.7
0.6
0.5
0.4
0.3
0.2
0.1
0.0
0.5
1
Position on chromosome 9 (Mb)

TC – rs553427

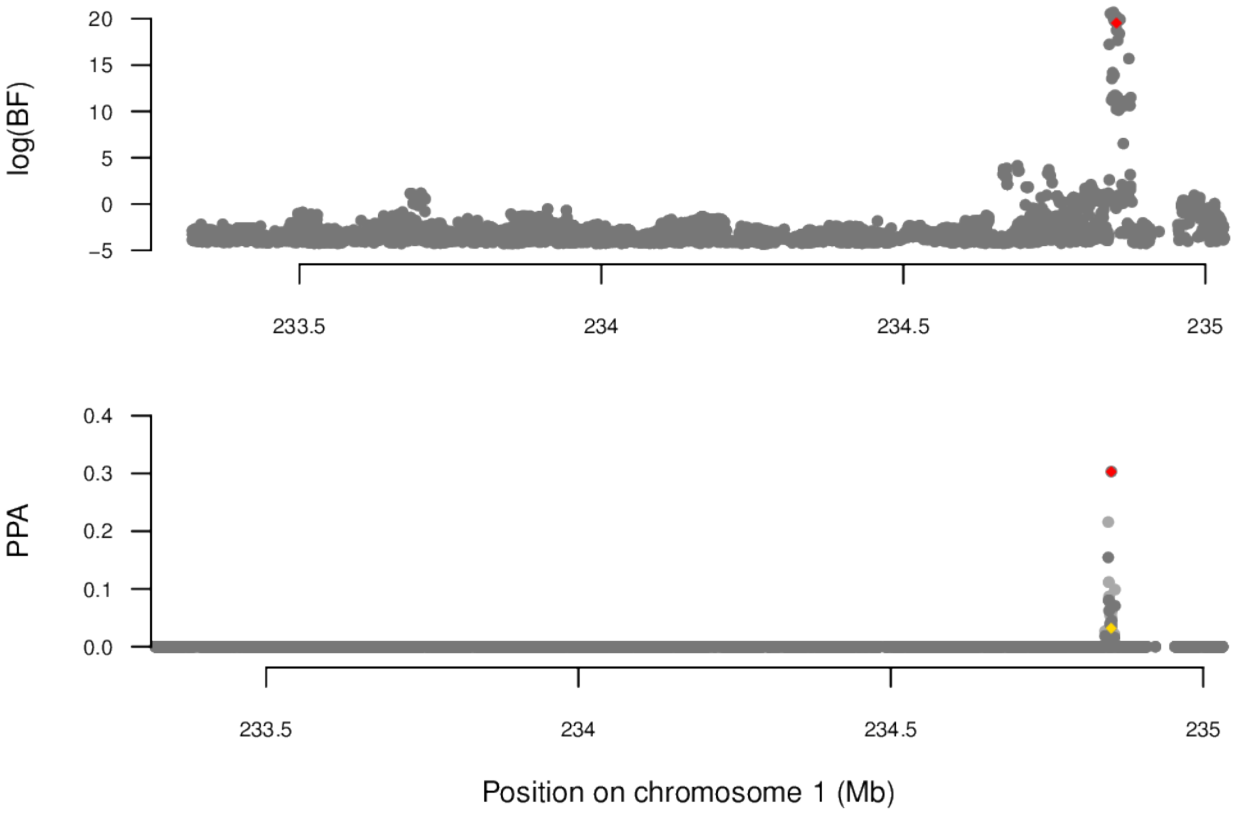

log(BF)
20
15
10
5
0
−5
233.5
234
234.5
235
PPA
0.4
0.3
0.2
0.1
0.0
233.5
234
234.5
235
Position on chromosome 1 (Mb)

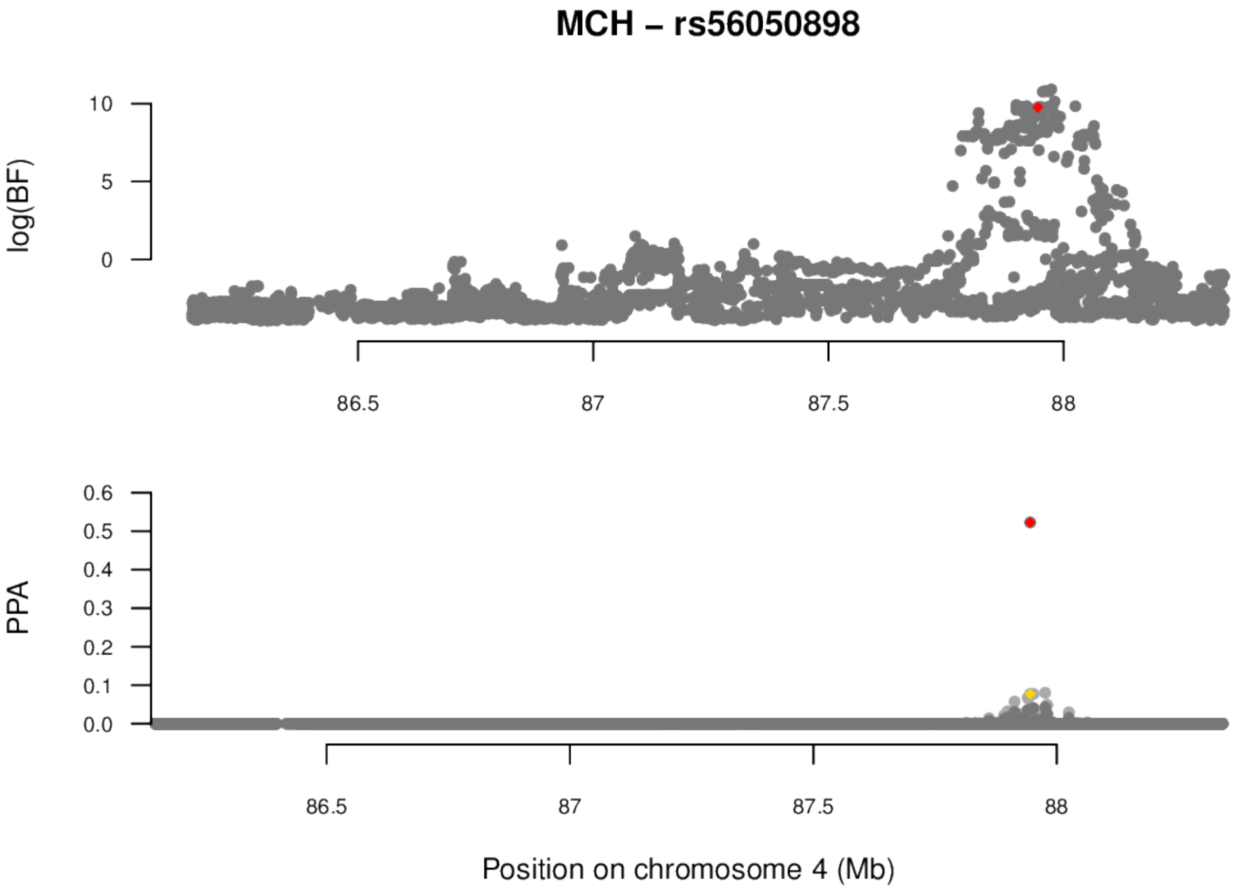
MCH – rs56050898
log(BF)
10
5
0
86.5
87
87.5
88
PPA
0.6
0.5
0.4
0.3
0.2
0.1
0.0
86.5
87
87.5
88
Position on chromosome 4 (Mb)

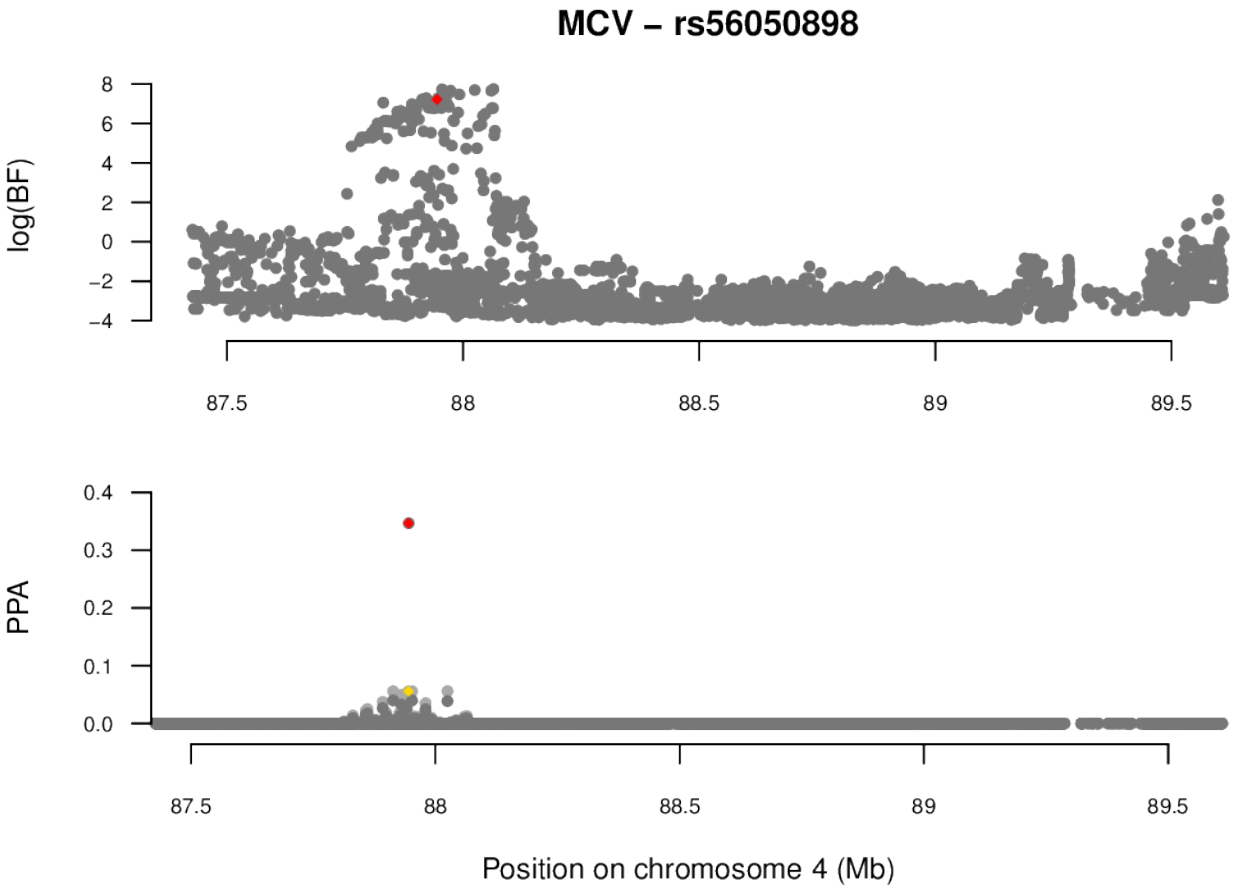
MCV – rs56050898
log(BF)
8
6
4
2
0
−2
−4
87.5
88
88.5
89
89.5
PPA
0.4
0.3
0.2
0.1
0.0
87.5
88
88.5
89
89.5
Position on chromosome 4 (Mb)

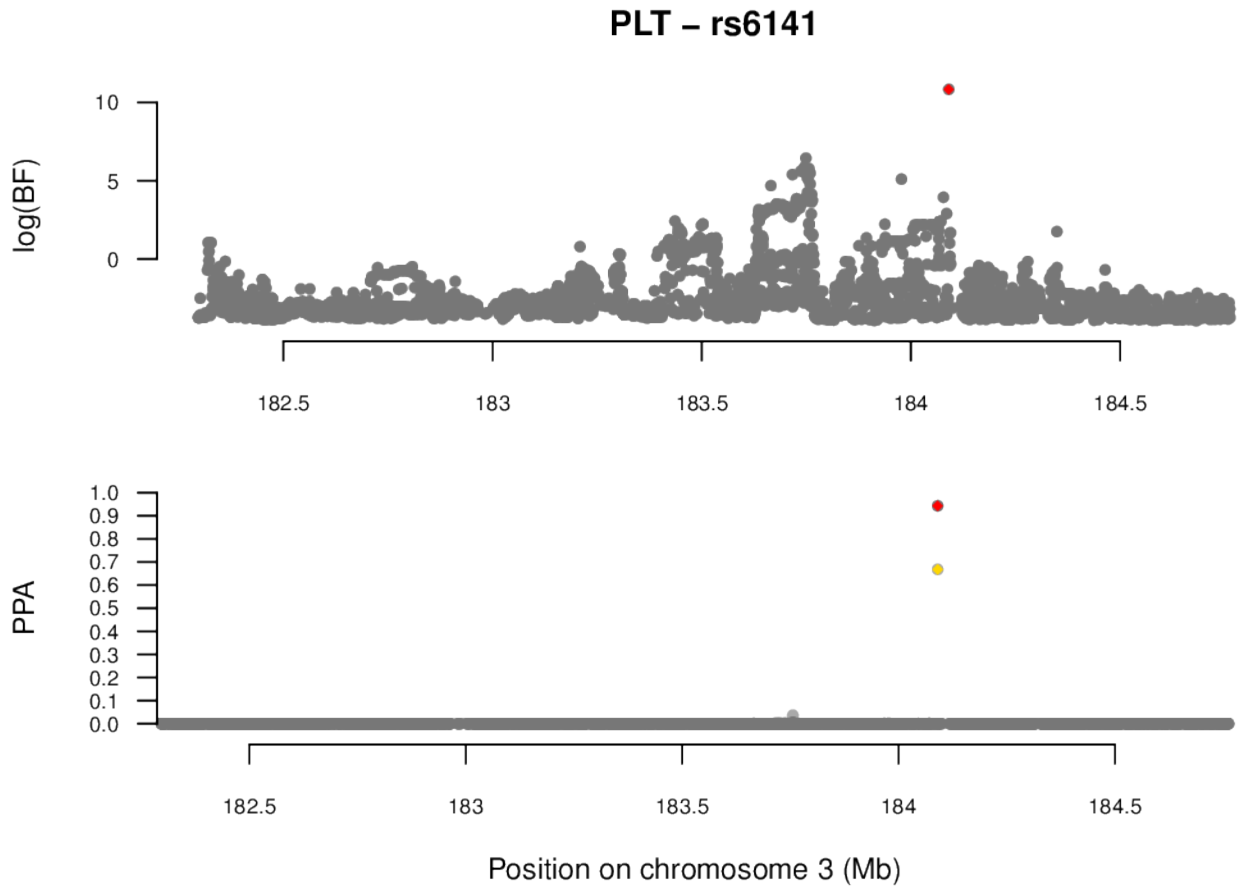

PLT – rs6141
log(BF)
10
5
0
182.5
183
183.5
184
184.5
PPA
1.0
0.9
0.8
0.7
0.6
0.5
0.4
0.3
0.2
0.1
0.0
182.5
183
183.5
184
184.5
Position on chromosome 3 (Mb)

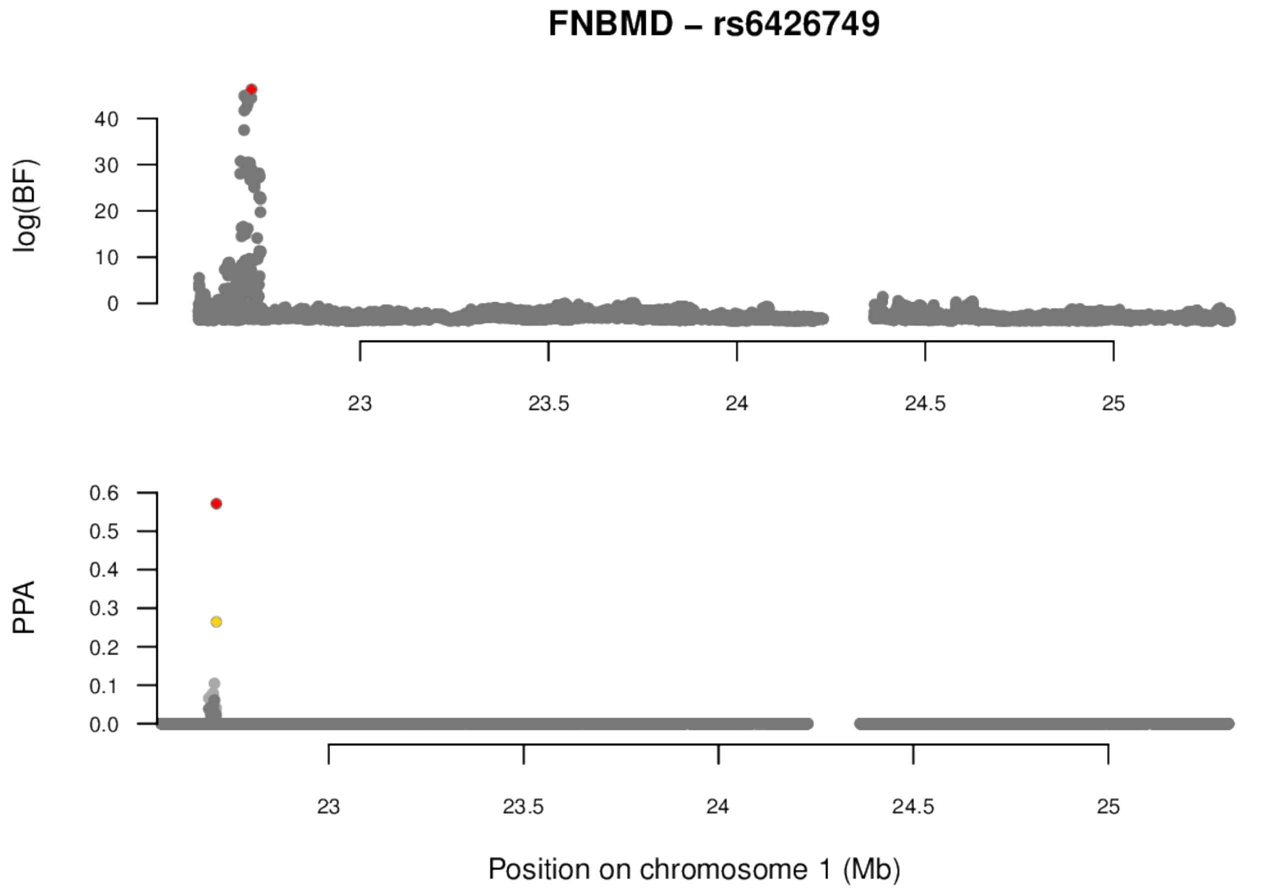
FNBMD – rs6426749
log(BF)
0
10
20
30
40
23
23.5
24
24.5
25
PPA
0.0
0.1
0.2
0.3
0.4
0.5
0.6
23
23.5
24
24.5
25
Position on chromosome 1 (Mb)

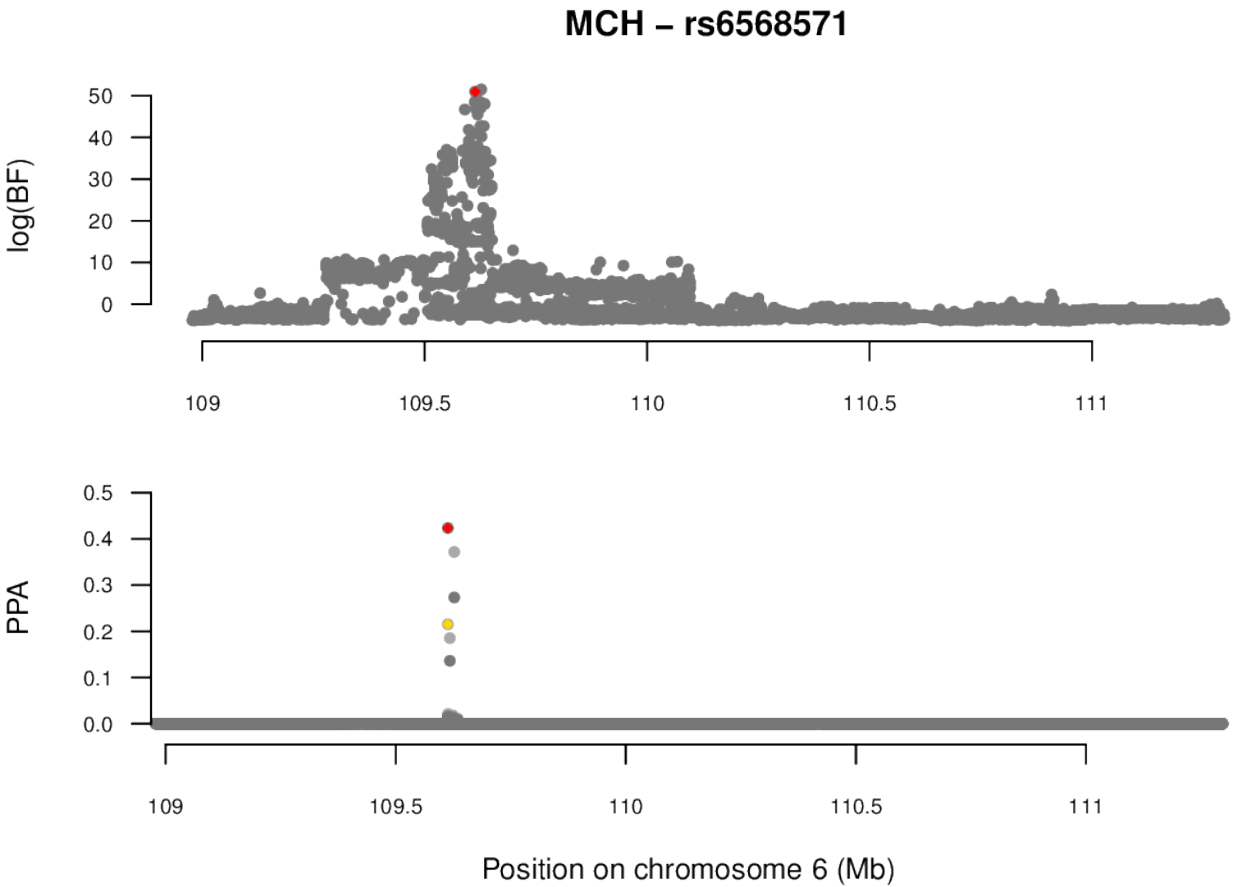
MCH – rs6568571
log(BF)
50
40
30
20
10
0
109
109.5
110
110.5
111
PPA
0.5
0.4
0.3
0.2
0.1
0.0
109
109.5
110
110.5
111
Position on chromosome 6 (Mb)

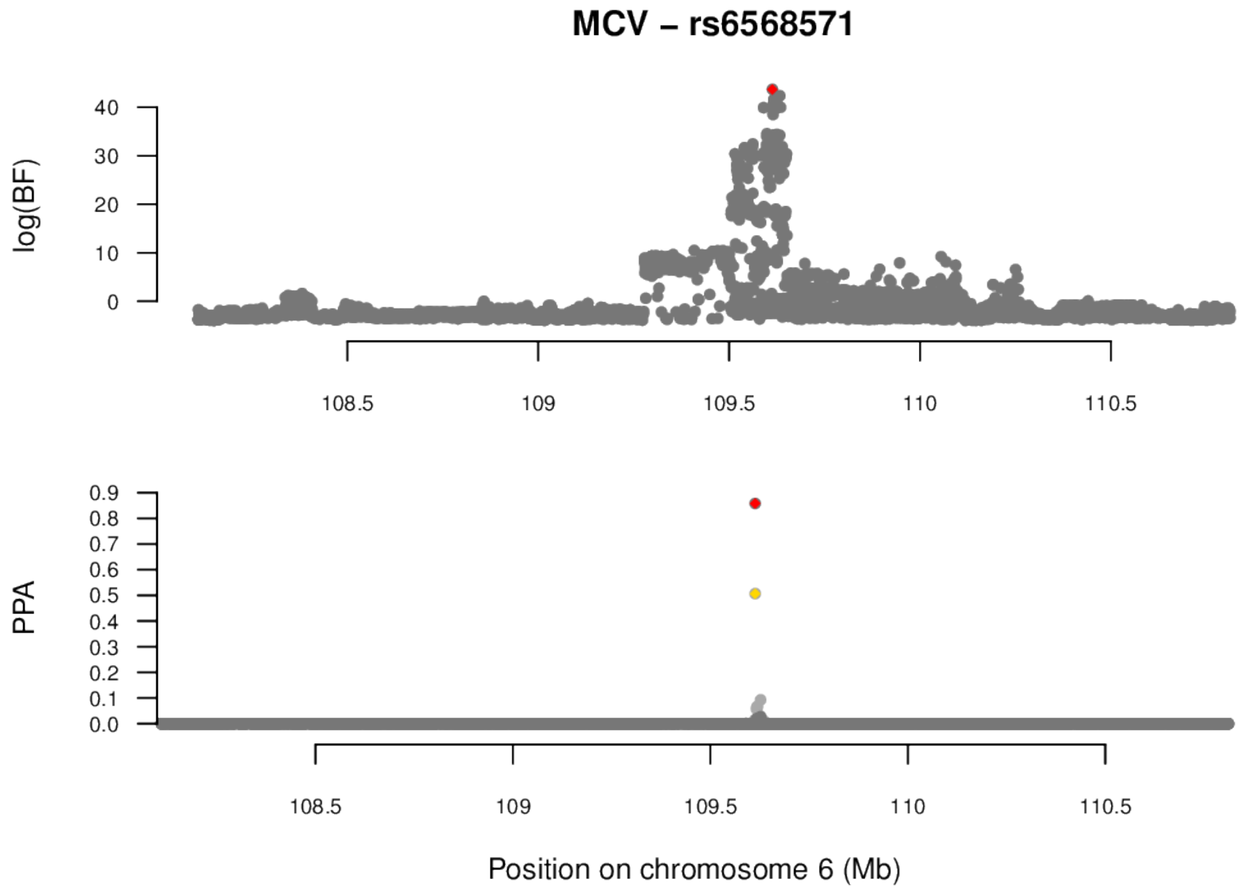
MCV – rs6568571
log(BF)
40
30
20
10
0
108.5
109
109.5
110
110.5
PPA
0.9
0.8
0.7
0.6
0.5
0.4
0.3
0.2
0.1
0.0
108.5
109
109.5
110
110.5
Position on chromosome 6 (Mb)

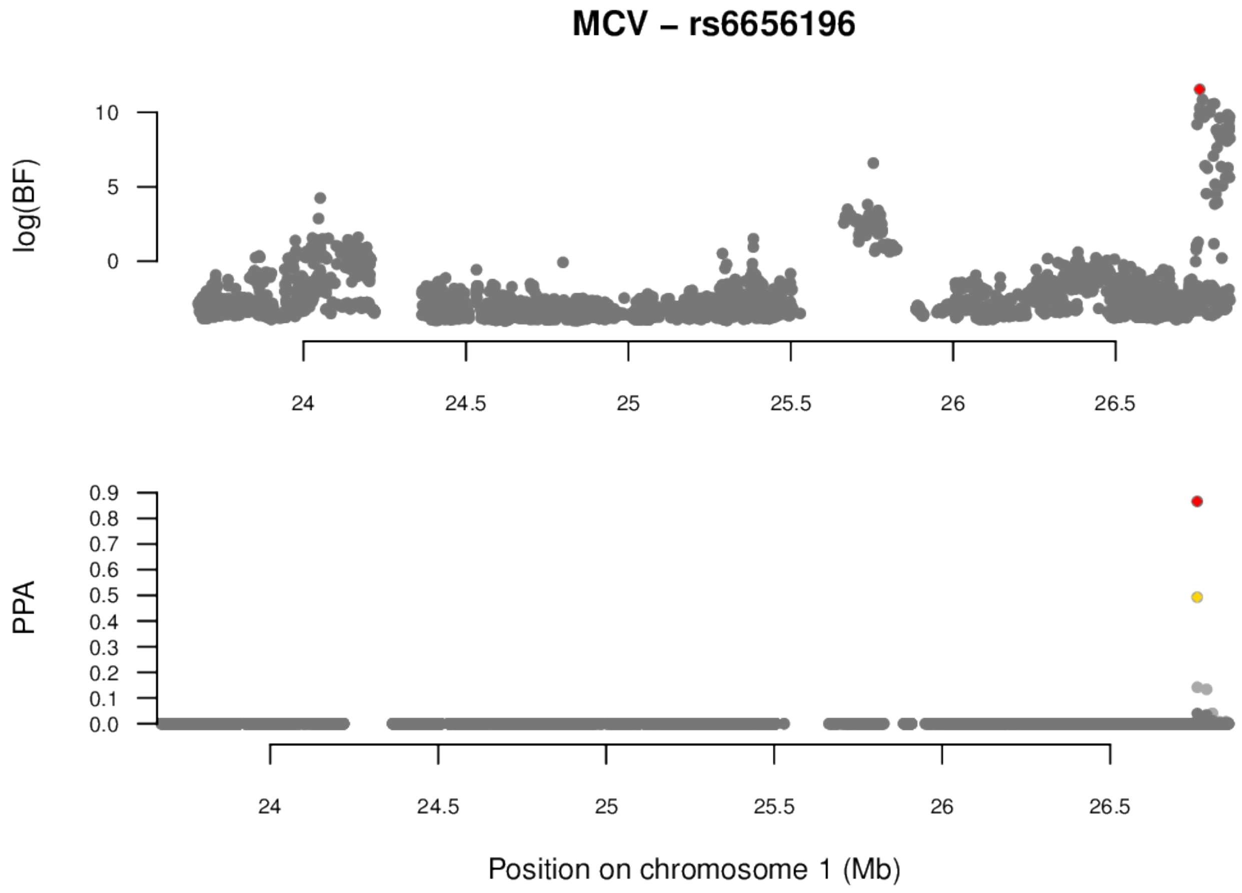

MCV – rs6656196
log(BF)
10
5
0
24
24.5
25
25.5
26
26.5
PPA
0.9
0.8
0.7
0.6
0.5
0.4
0.3
0.2
0.1
0.0
24
24.5
25
25.5
26
26.5
Position on chromosome 1 (Mb)

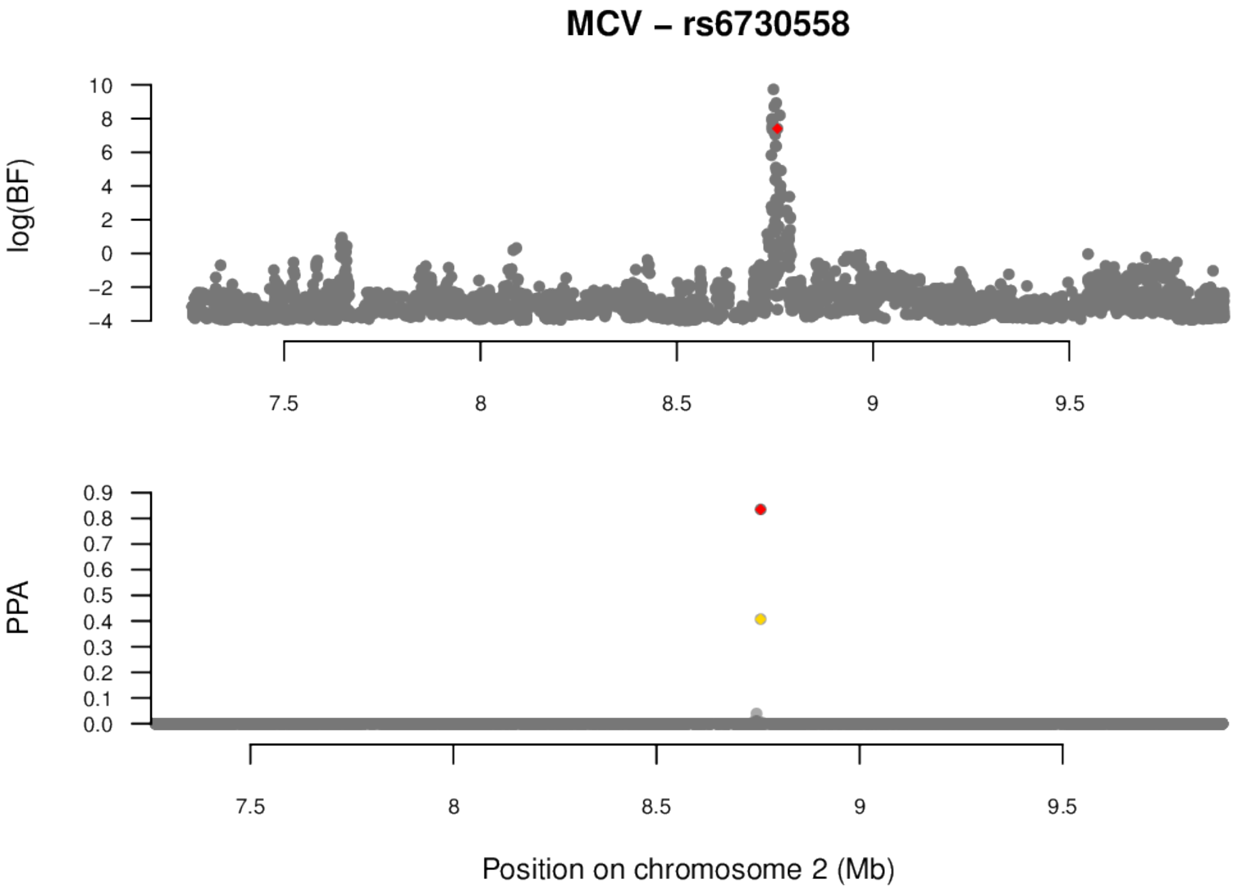

MCV – rs6730558
log(BF)
10
8
6
4
2
0
−2
−4
7.5
8
8.5
9
9.5
PPA
0.9
0.8
0.7
0.6
0.5
0.4
0.3
0.2
0.1
0.0
7.5
8
8.5
9
9.5
Position on chromosome 2 (Mb)

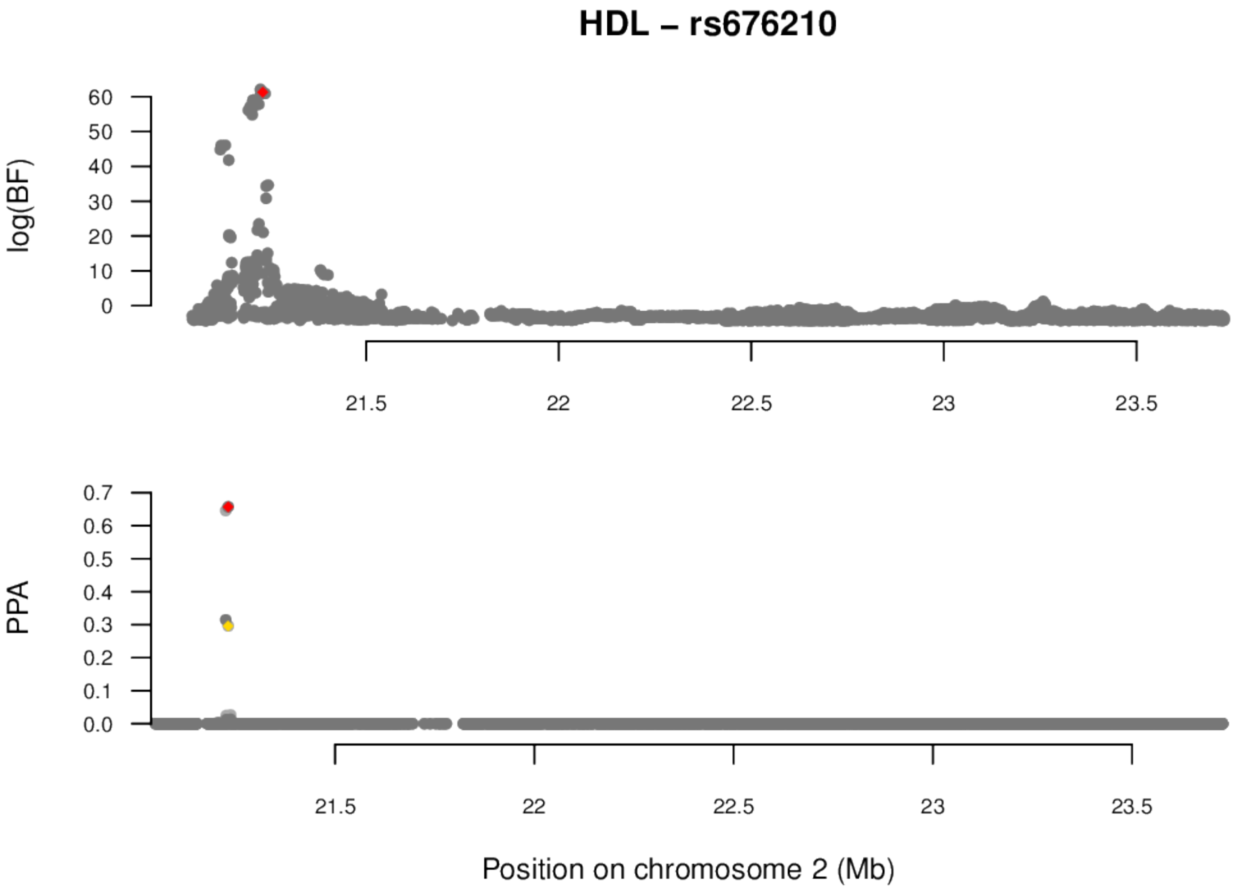

HDL – rs676210
log(BF)
60
50
40
30
20
10
0
21.5
22
22.5
23
23.5
PPA
0.7
0.6
0.5
0.4
0.3
0.2
0.1
0.0
21.5
22
22.5
23
23.5
Position on chromosome 2 (Mb)

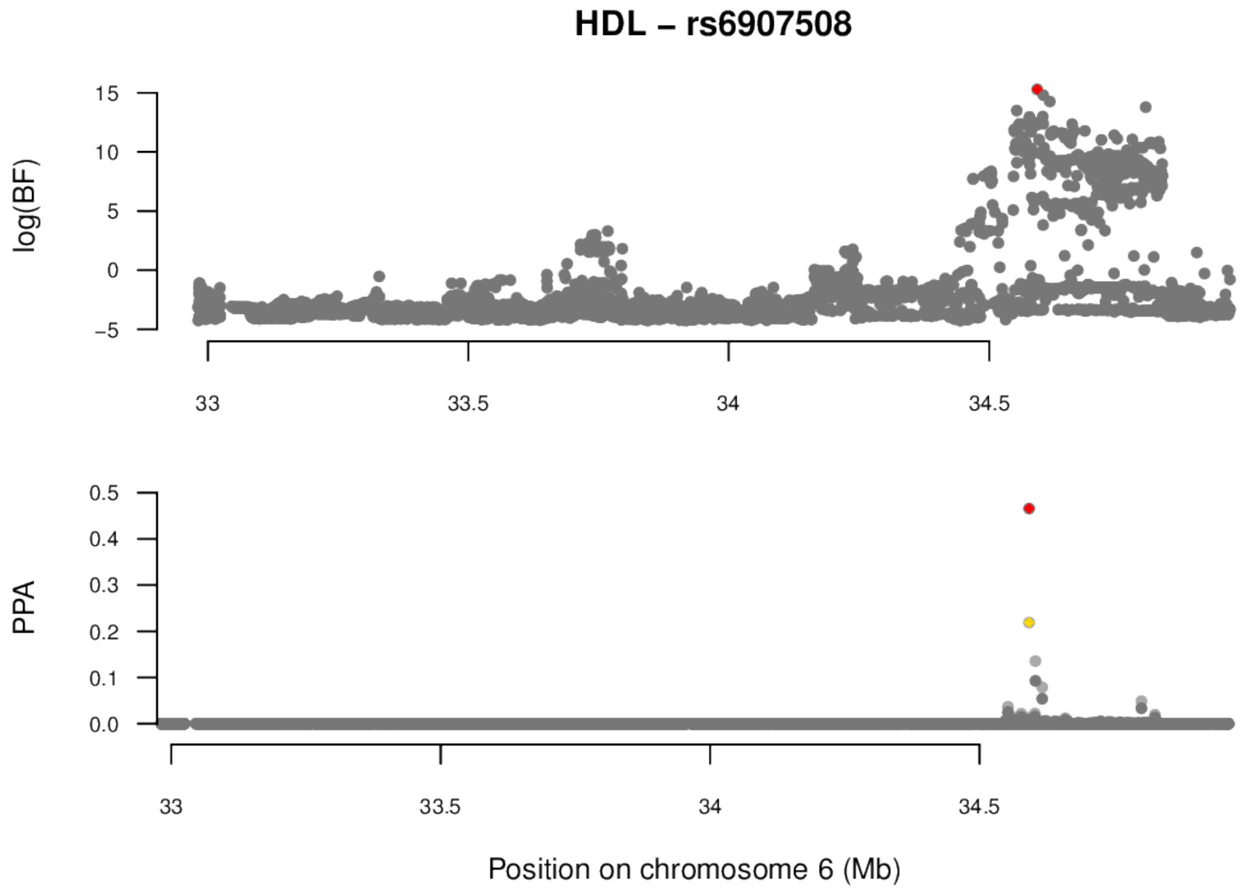

HDL – rs6907508
log(BF)
15
10
5
0
−5
33
33.5
34
34.5
PPA
0.5
0.4
0.3
0.2
0.1
0.0
33
33.5
34
34.5
Position on chromosome 6 (Mb)

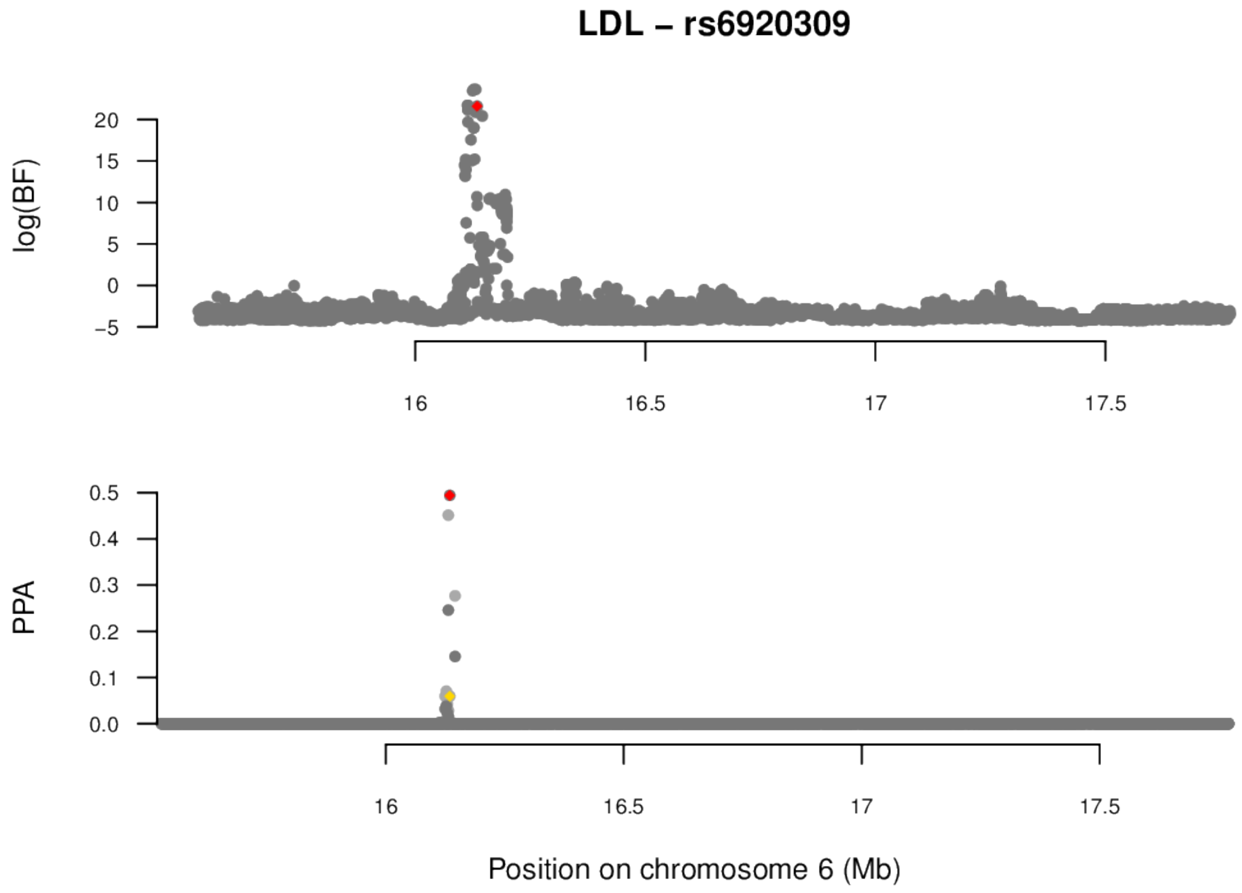

LDL – rs6920309
log(BF)
20
15
10
5
0
−5
16
16.5
17
17.5
PPA
0.5
0.4
0.3
0.2
0.1
0.0
16
16.5
17
17.5
Position on chromosome 6 (Mb)

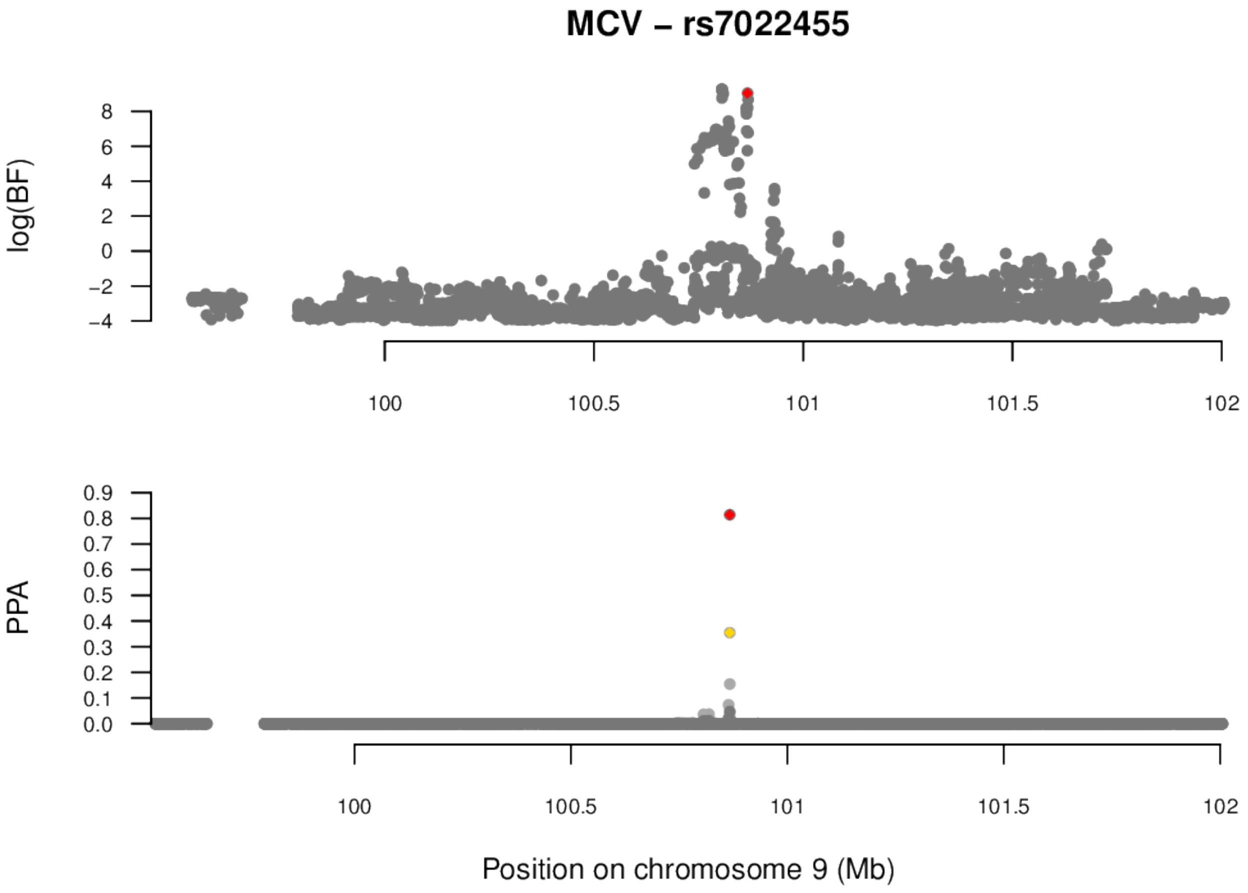
MCV – rs7022455
log(BF)
8
6
4
2
0
−2
−4
100
100.5
101
101.5
102
PPA
0.9
0.8
0.7
0.6
0.5
0.4
0.3
0.2
0.1
0.0
100
100.5
101
101.5
102
Position on chromosome 9 (Mb)

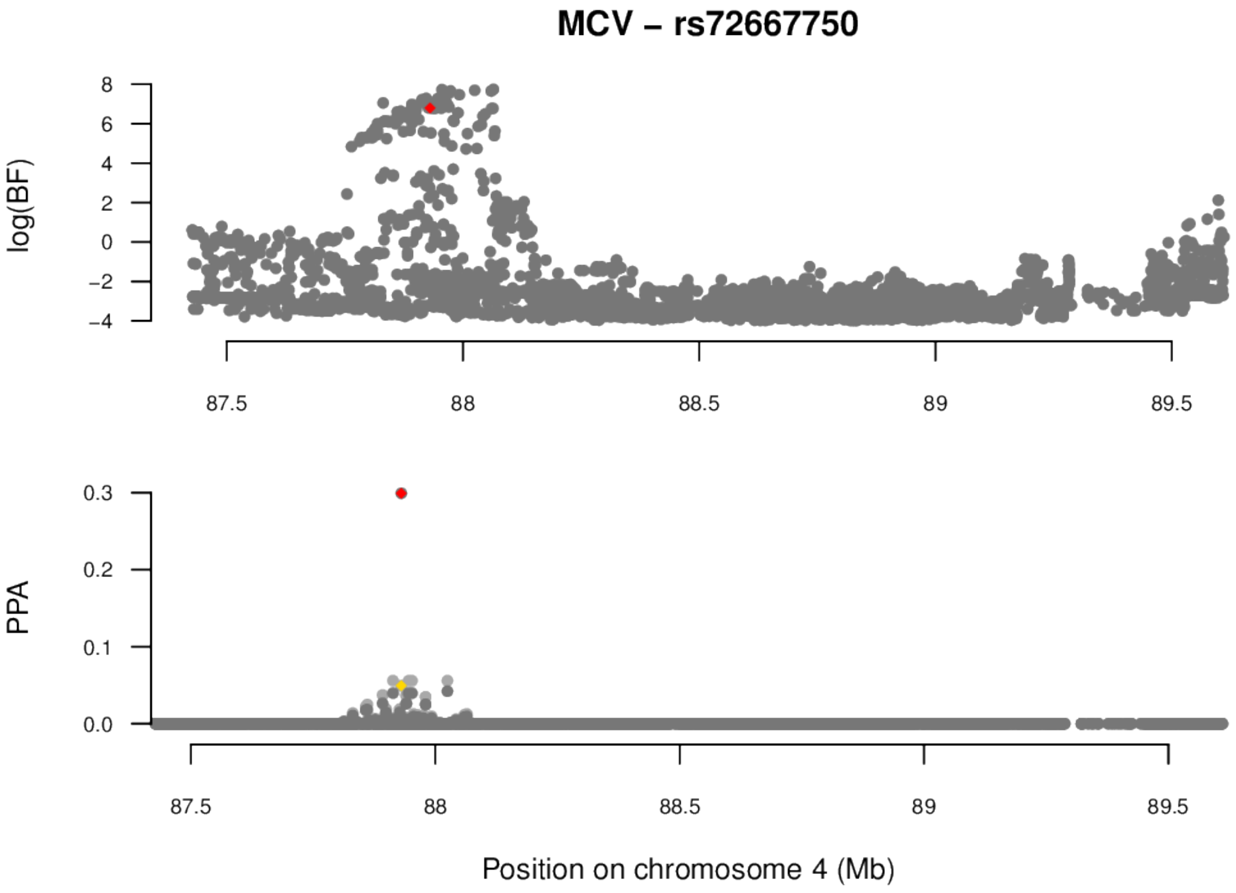

MCV – rs72667750
log(BF)
8
6
4
2
0
−2
−4
87.5
88
88.5
89
89.5
PPA
0.3
0.2
0.1
0.0
87.5
88
88.5
89
89.5
Position on chromosome 4 (Mb)

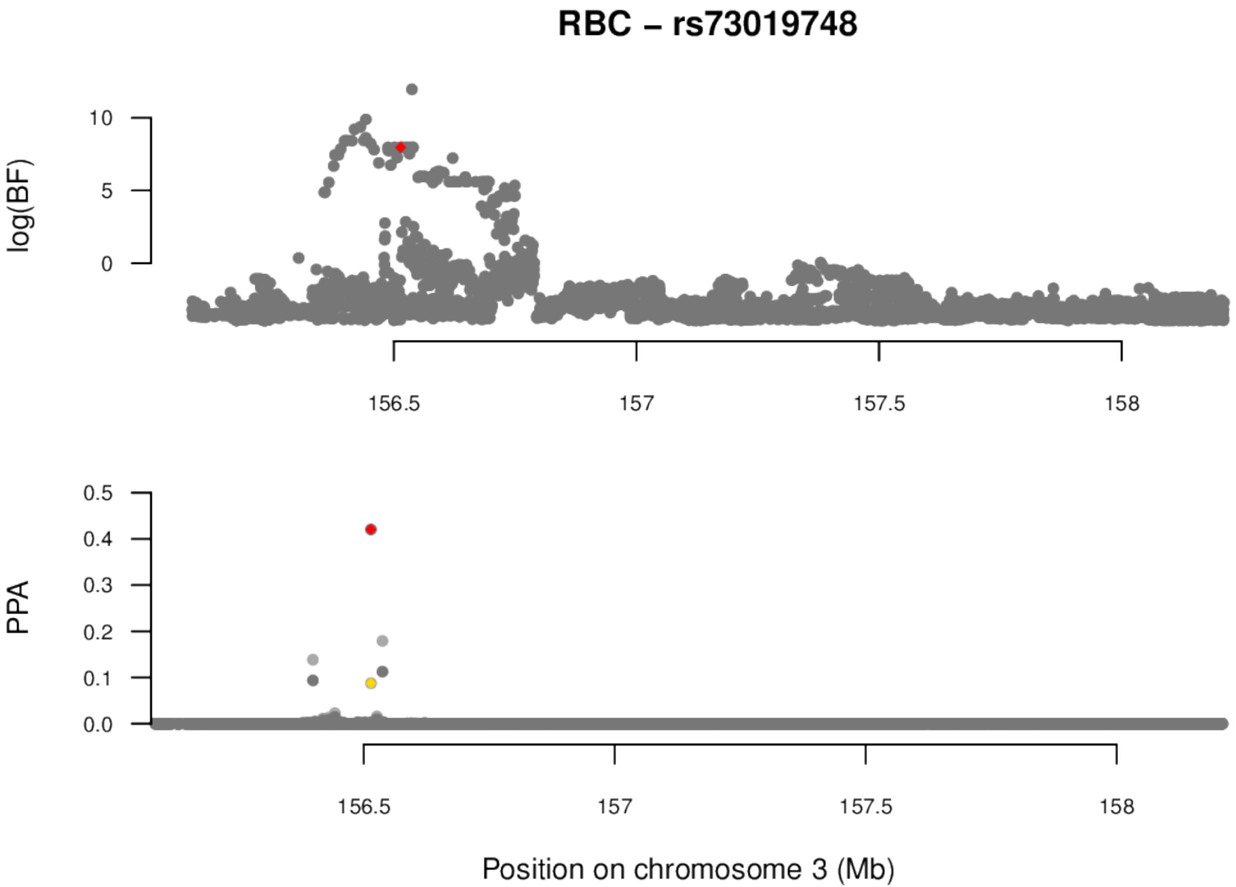

RBC – rs73019748
log(BF)
10
5
0
156.5
157
157.5
158
PPA
0.5
0.4
0.3
0.2
0.1
0.0
156.5
157
157.5
158
Position on chromosome 3 (Mb)

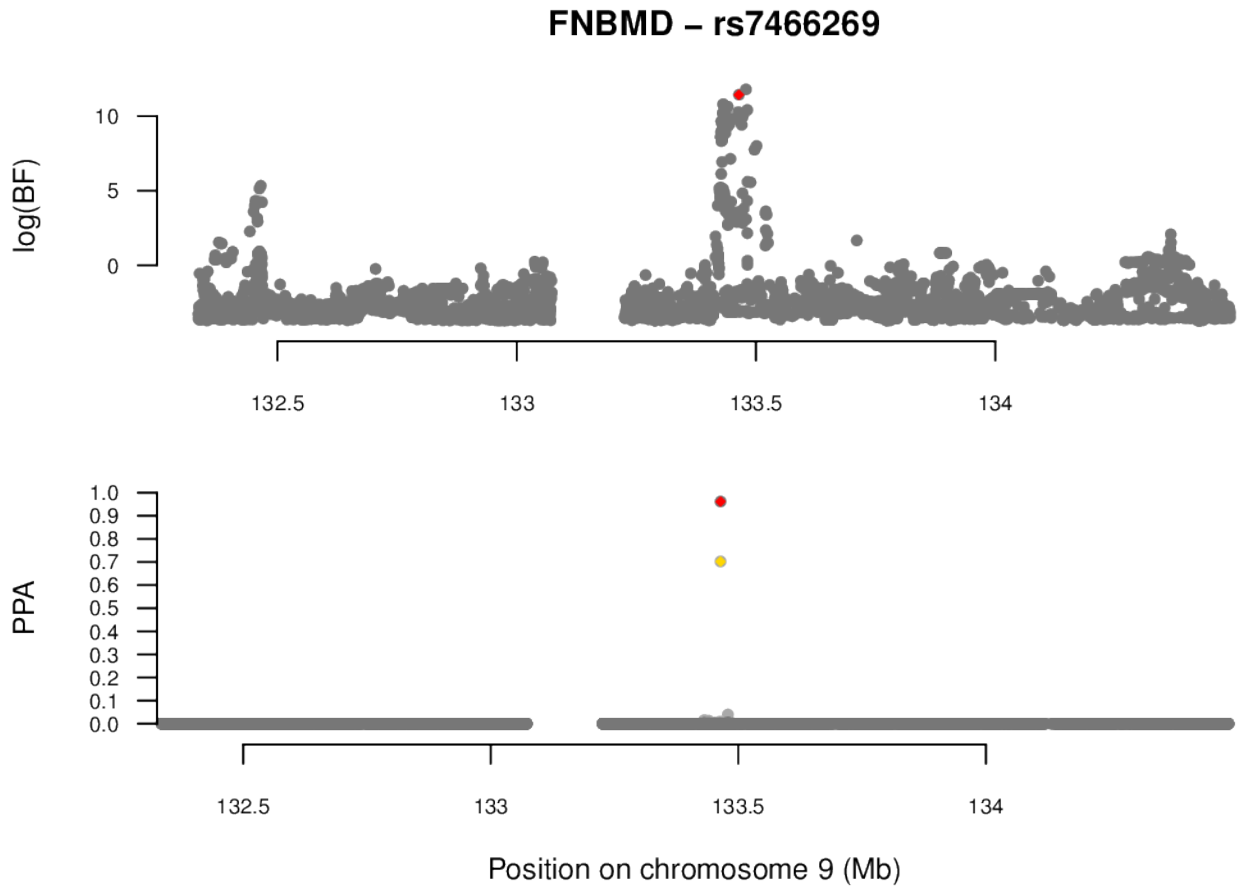
FNBMD – rs7466269
log(BF)
10
5
0
132.5
133
133.5
134
PPA
1.0
0.9
0.8
0.7
0.6
0.5
0.4
0.3
0.2
0.1
0.0
132.5
133
133.5
134
Position on chromosome 9 (Mb)

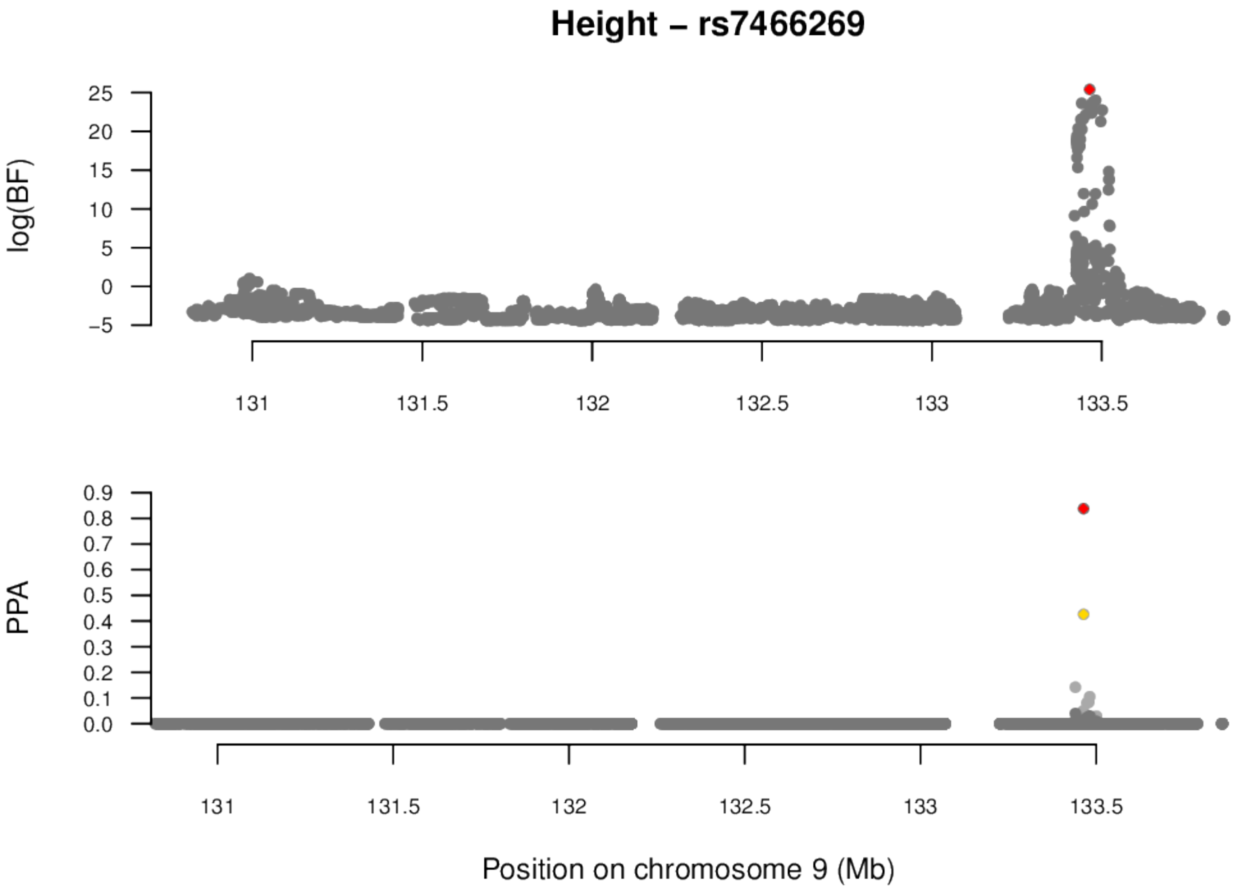
Height – rs7466269
log(BF)
25
20
15
10
5
0
−5
131
131.5
132
132.5
133
133.5
PPA
0.9
0.8
0.7
0.6
0.5
0.4
0.3
0.2
0.1
0.0
131
131.5
132
132.5
133
133.5
Position on chromosome 9 (Mb)

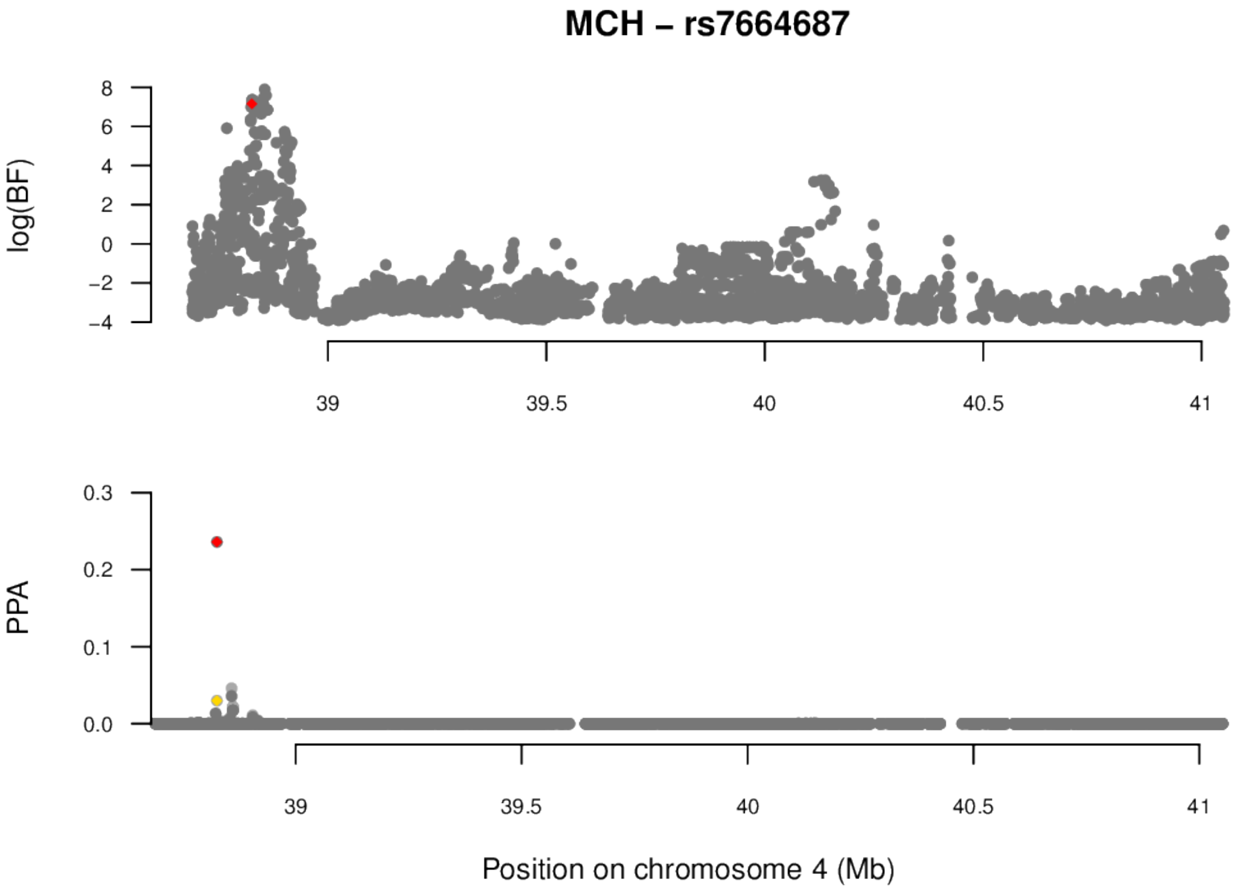
MCH – rs7664687
log(BF)
8
6
4
2
0
−2
−4
39
39.5
40
40.5
41
PPA
0.3
0.2
0.1
0.0
39
39.5
40
40.5
41
Position on chromosome 4 (Mb)

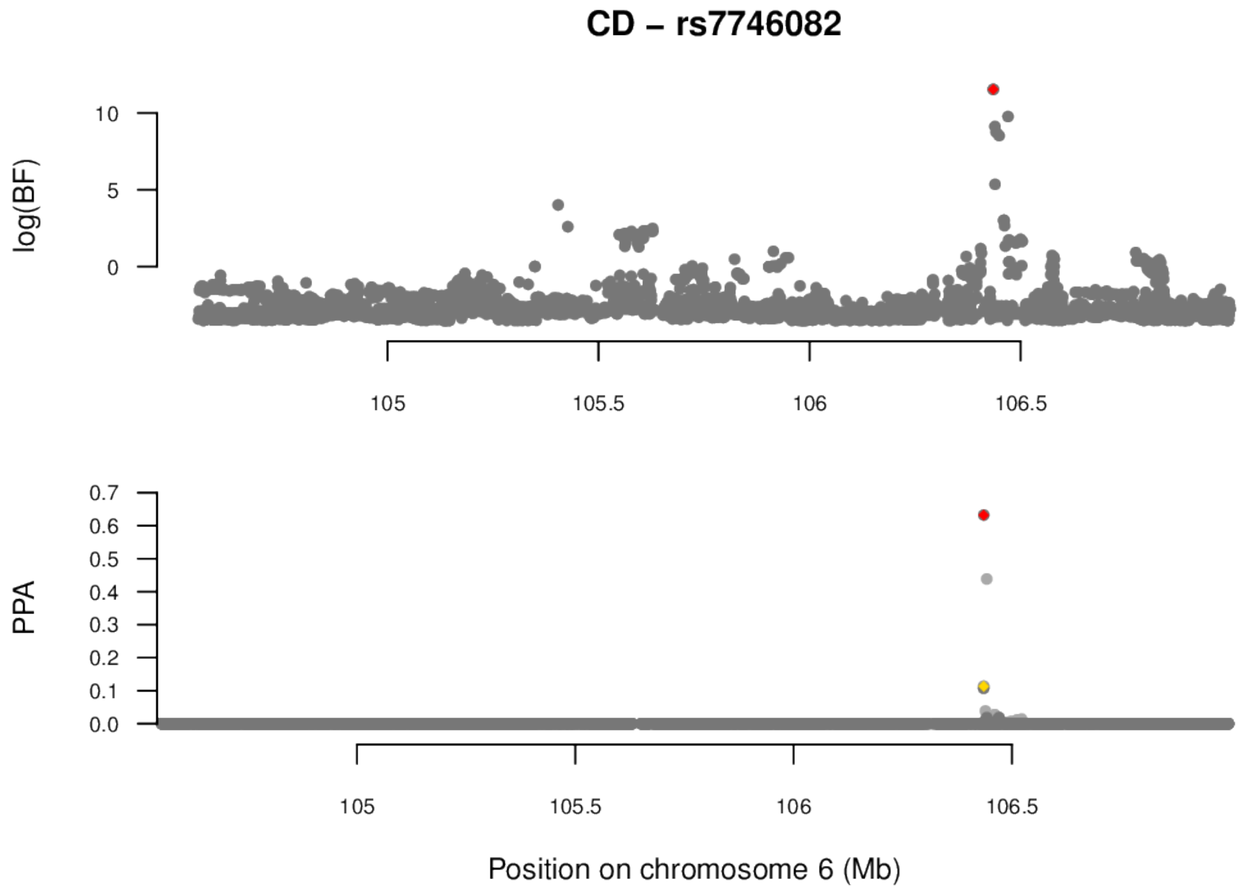
CD – rs7746082
log(BF)
10
5
0
105
105.5
106
106.5
PPA
0.7
0.6
0.5
0.4
0.3
0.2
0.1
0.0
105
105.5
106
106.5
Position on chromosome 6 (Mb)

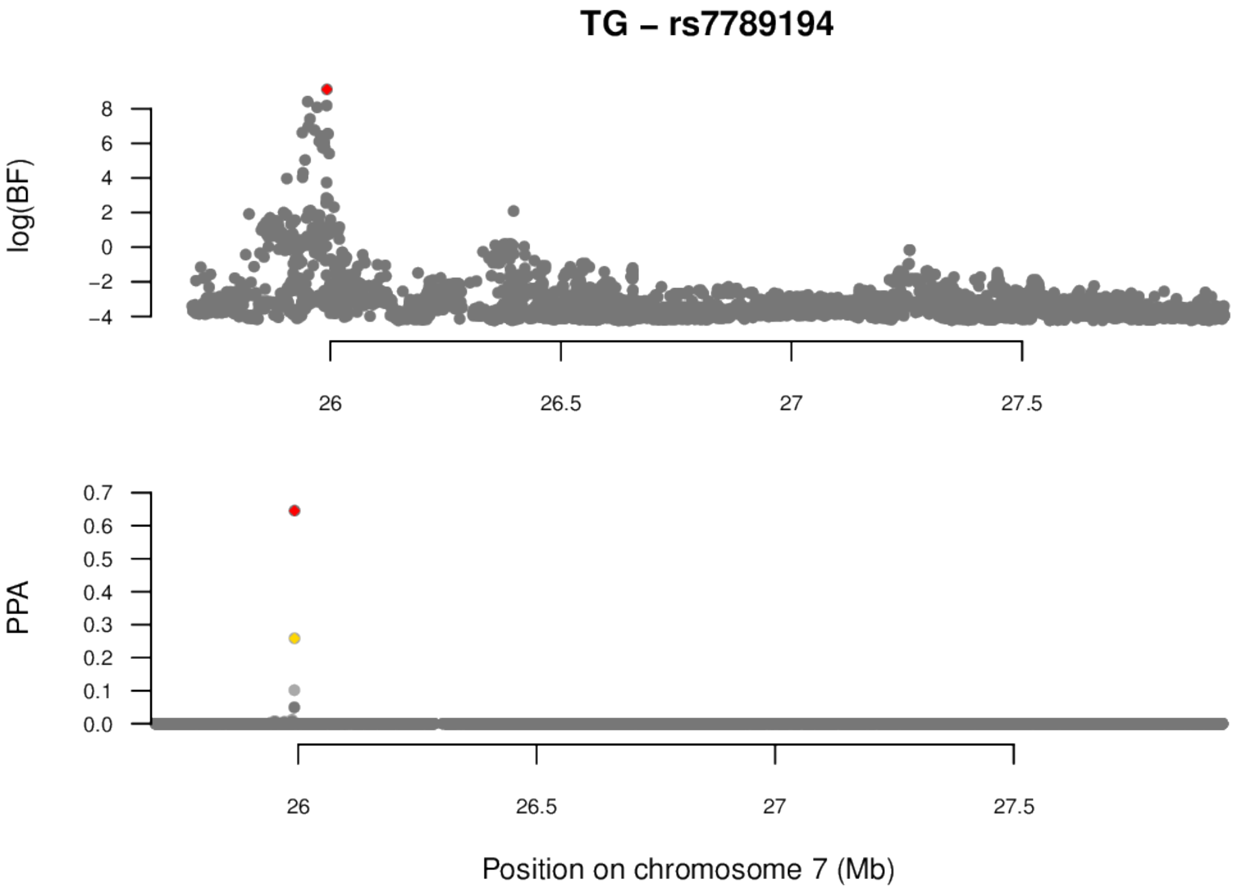

TG – rs7789194
log(BF)
8
6
4
2
0
−2
−4
26
26.5
27
27.5
PPA
0.7
0.6
0.5
0.4
0.3
0.2
0.1
0.0
26
26.5
27
27.5
Position on chromosome 7 (Mb)

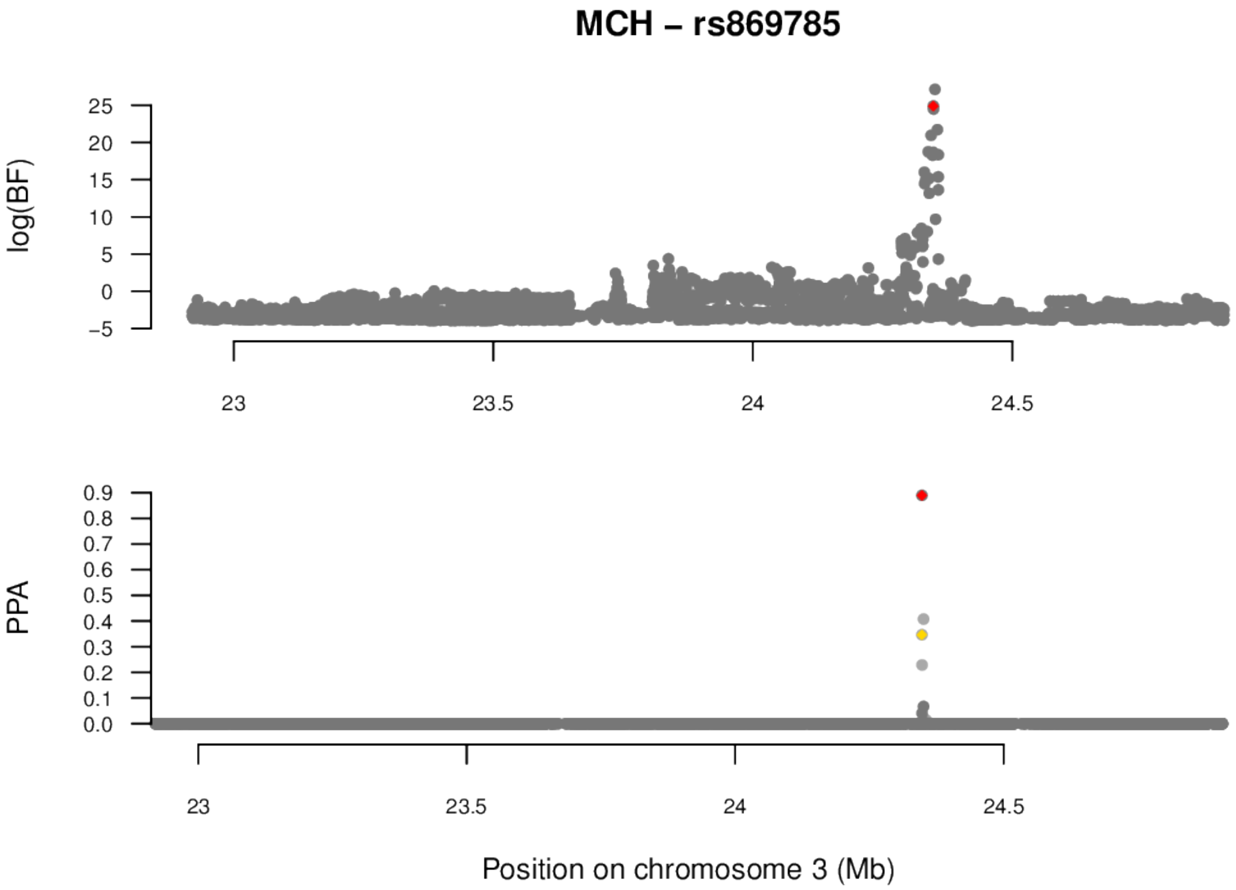

MCH – rs869785
log(BF)
25
20
15
10
5
0
−5
23
23.5
24
24.5
PPA
0.9
0.8
0.7
0.6
0.5
0.4
0.3
0.2
0.1
0.0
23
23.5
24
24.5
Position on chromosome 3 (Mb)

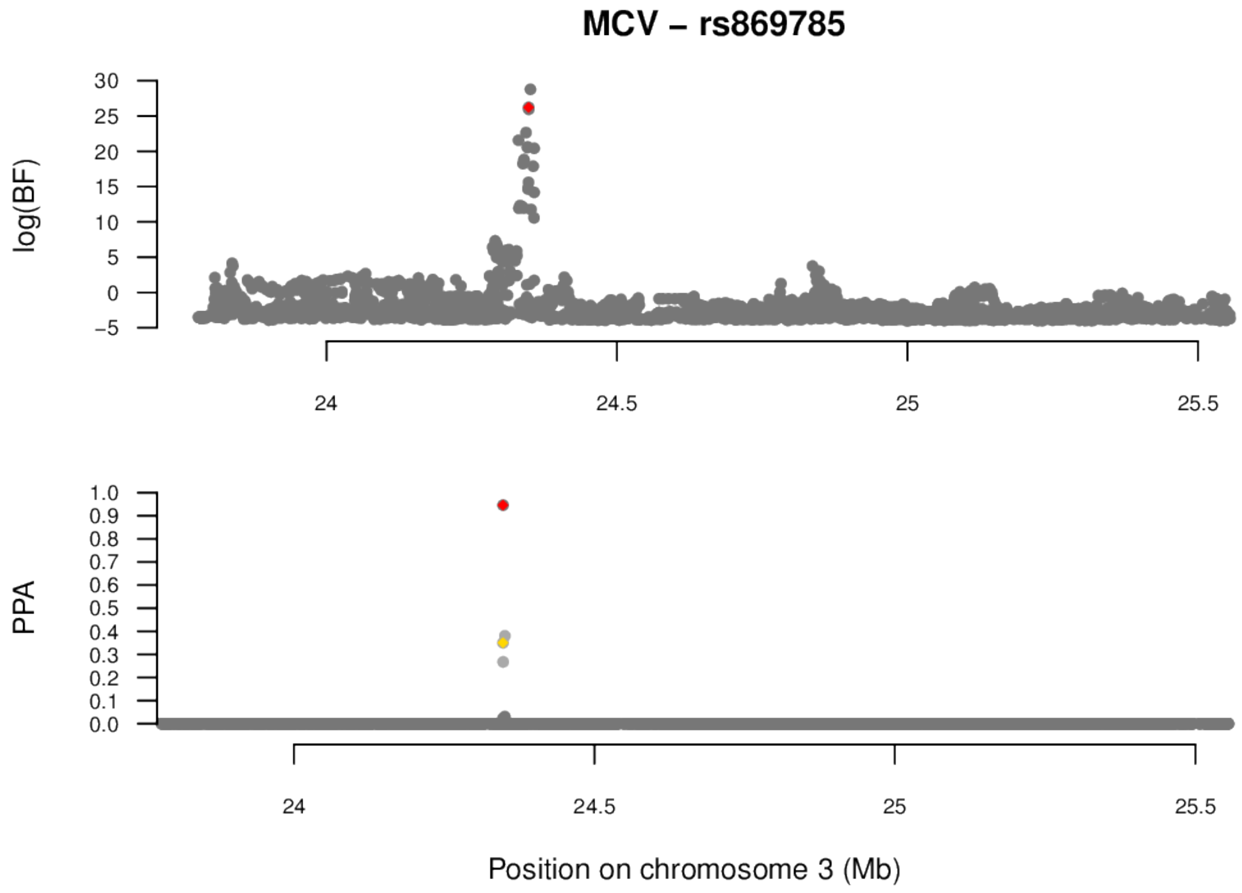

MCV – rs869785
log(BF)
30
25
20
15
10
5
0
–5
24
24.5
25
25.5
PPA
1.0
0.9
0.8
0.7
0.6
0.5
0.4
0.3
0.2
0.1
0.0
24
24.5
25
25.5
Position on chromosome 3 (Mb)

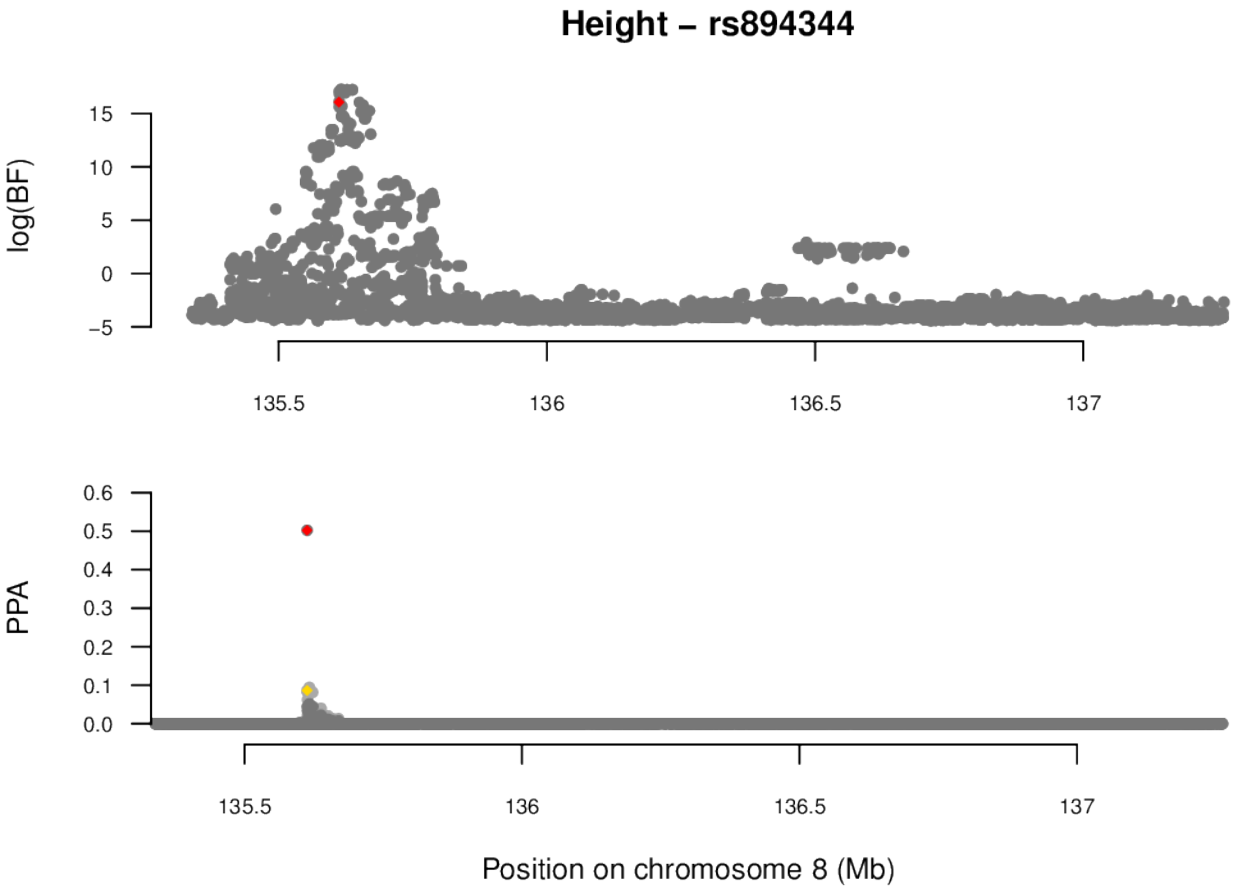
Height – rs894344
log(BF)
15
10
5
0
−5
135.5
136
136.5
137
PPA
0.6
0.5
0.4
0.3
0.2
0.1
0.0
135.5
136
136.5
137
Position on chromosome 8 (Mb)

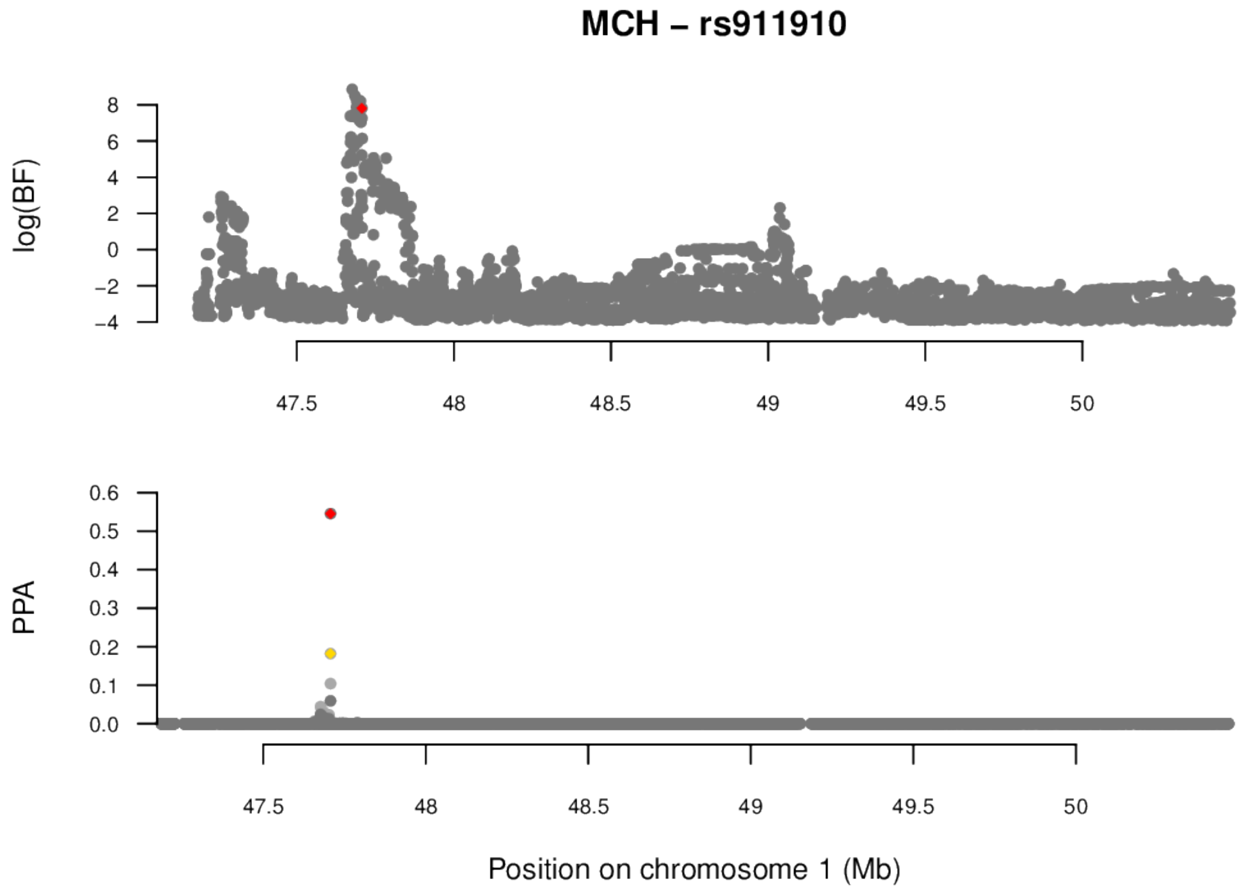
MCH – rs911910
log(BF)
8
6
4
2
0
−2
−4
47.5
48
48.5
49
49.5
50
PPA
0.6
0.5
0.4
0.3
0.2
0.1
0.0
47.5
48
48.5
49
49.5
50
Position on chromosome 1 (Mb)

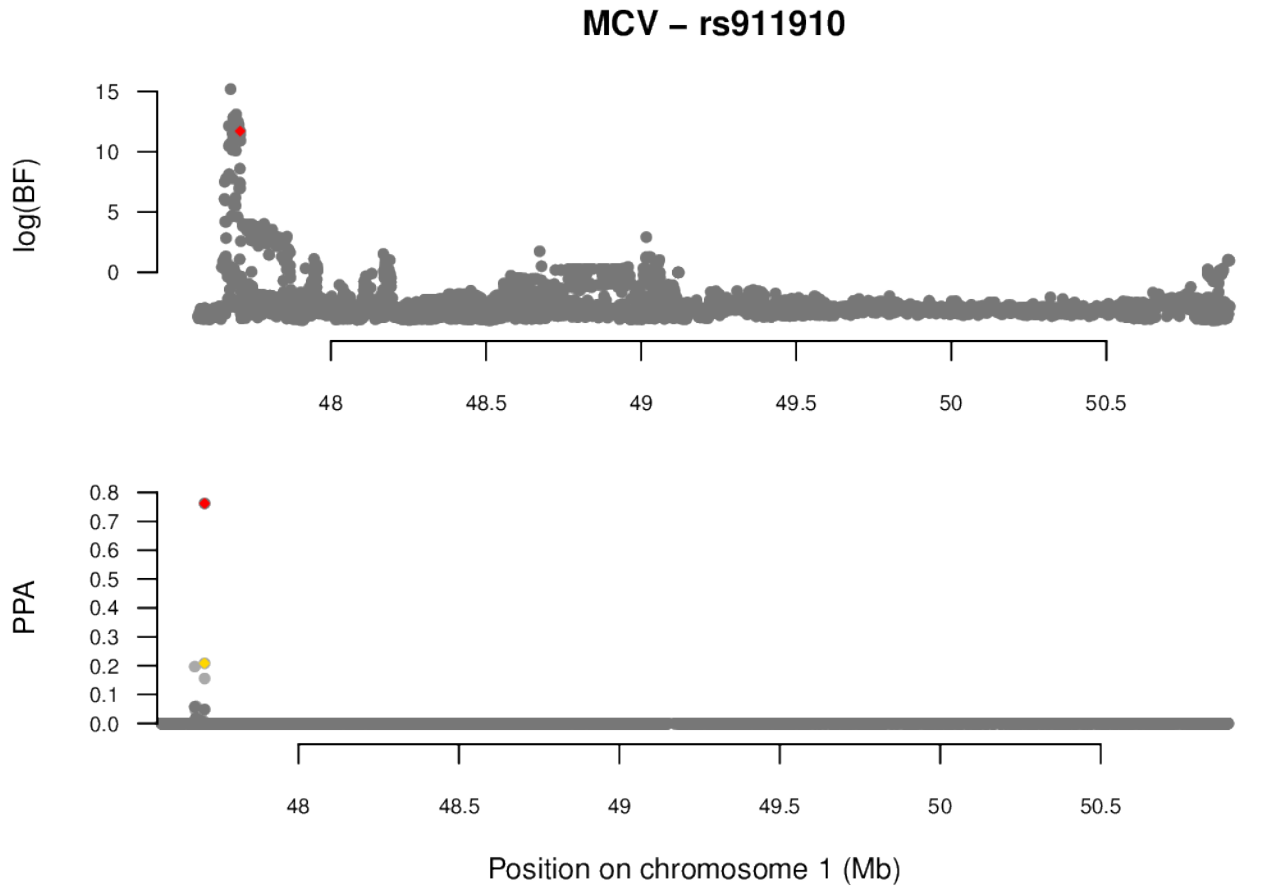
MCV – rs911910
log(BF)
15
10
5
0
48
48.5
49
49.5
50
50.5
PPA
0.8
0.7
0.6
0.5
0.4
0.3
0.2
0.1
0.0
48
48.5
49
49.5
50
50.5
Position on chromosome 1 (Mb)

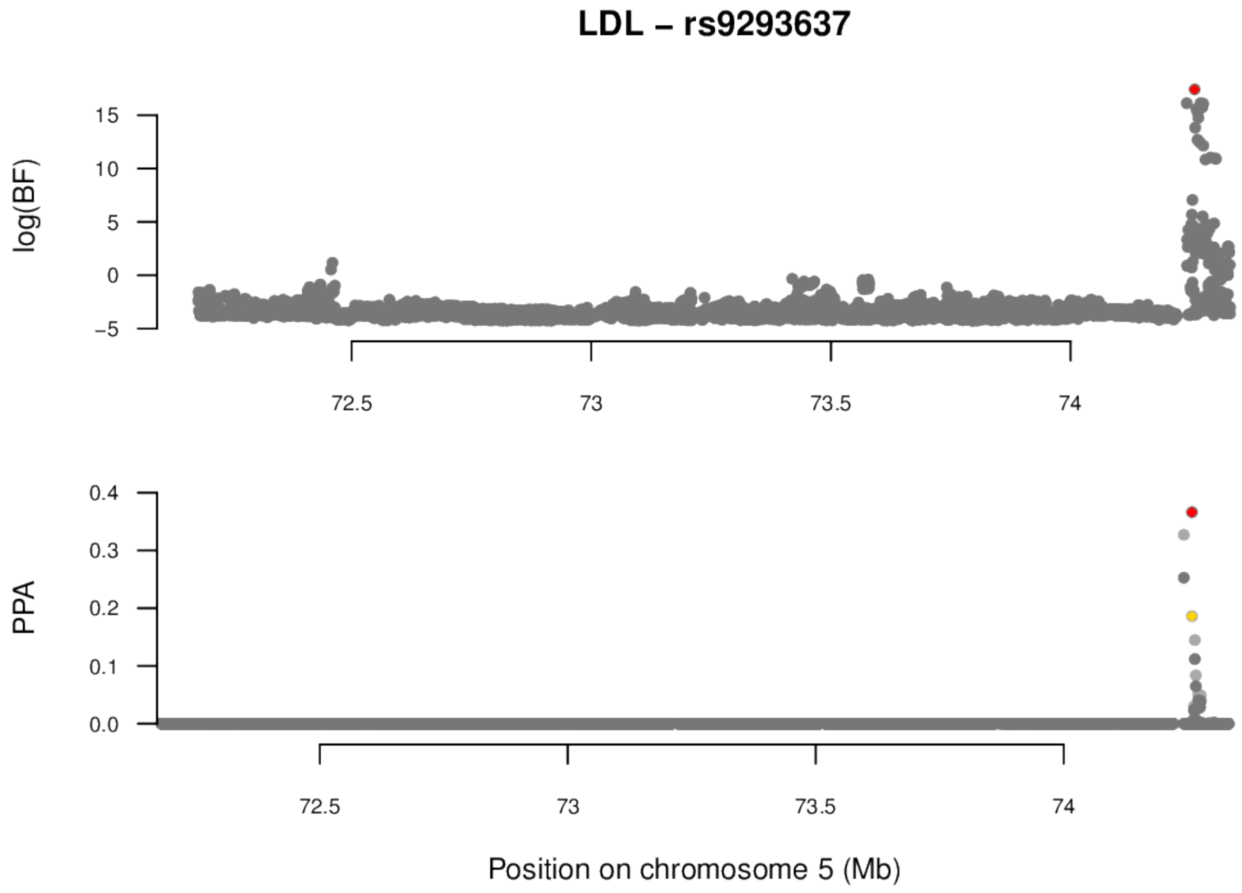

LDL – rs9293637
log(BF)
15
10
5
0
−5
72.5
73
73.5
74
PPA
0.4
0.3
0.2
0.1
0.0
72.5
73
73.5
74
Position on chromosome 5 (Mb)

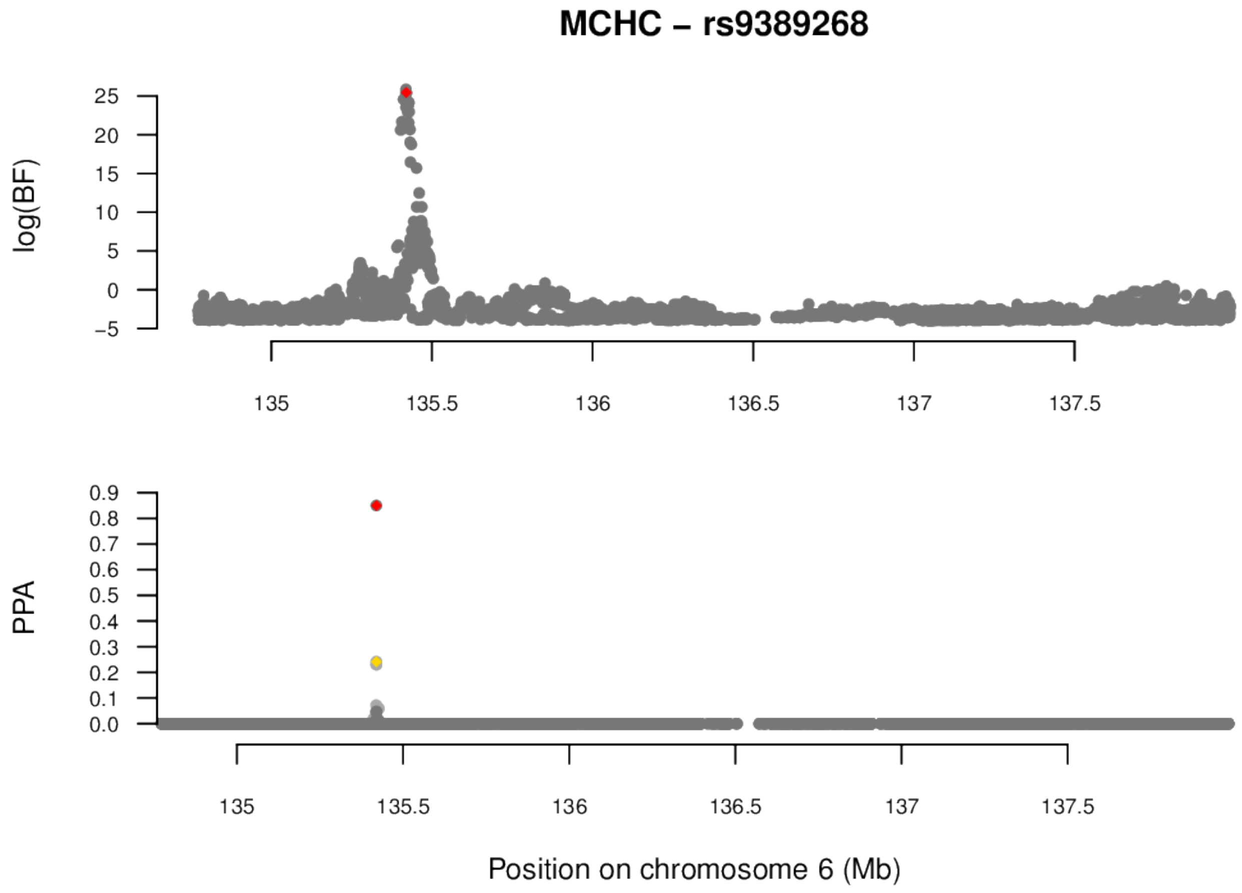

MCHC – rs9389268
log(BF)
25
20
15
10
5
0
−5
135
135.5
136
136.5
137
137.5
PPA
0.9
0.8
0.7
0.6
0.5
0.4
0.3
0.2
0.1
0.0
135
135.5
136
136.5
137
137.5
Position on chromosome 6 (Mb)

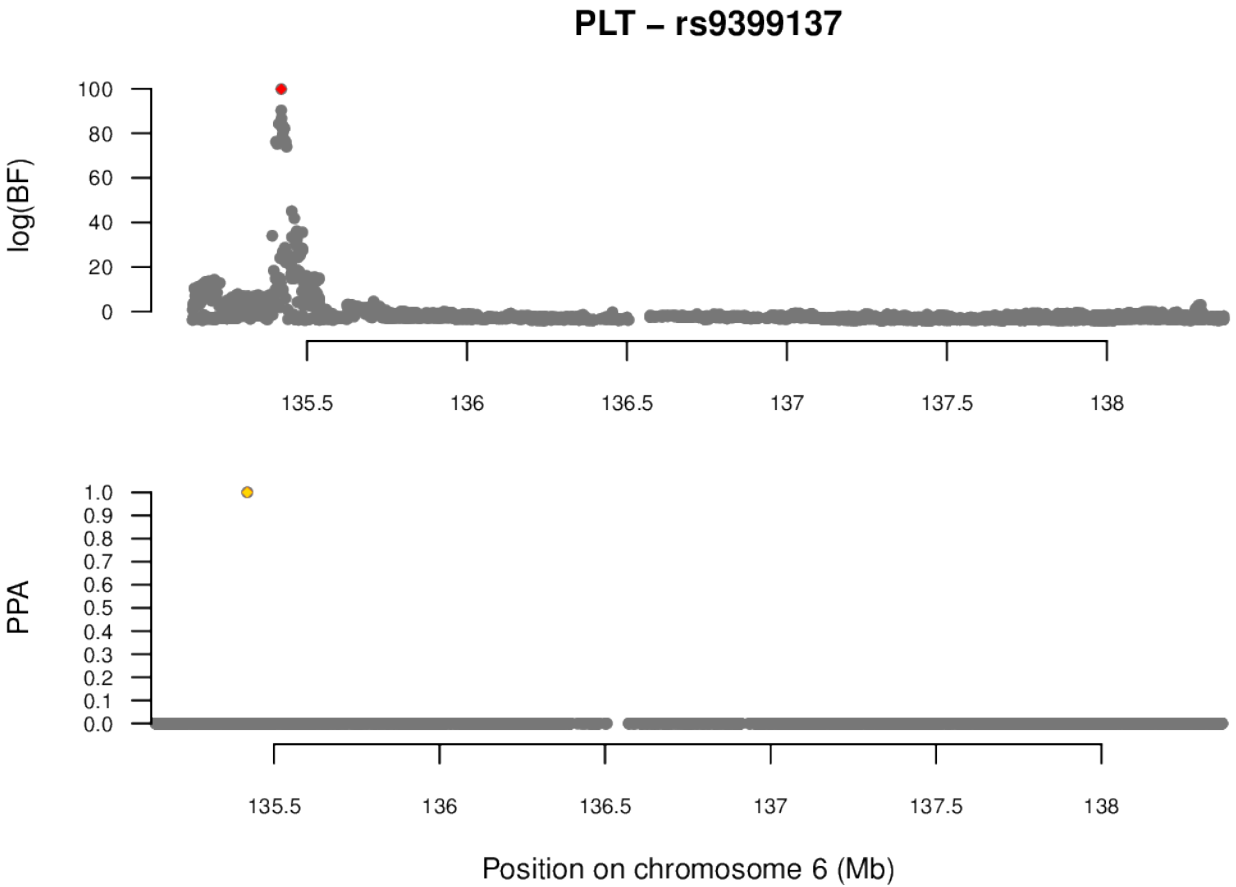
PLT – rs9399137
log(BF)
100
80
60
40
20
0
135.5
136
136.5
137
137.5
138
PPA
1.0
0.9
0.8
0.7
0.6
0.5
0.4
0.3
0.2
0.1
0.0
135.5
136
136.5
137
137.5
138
Position on chromosome 6 (Mb)

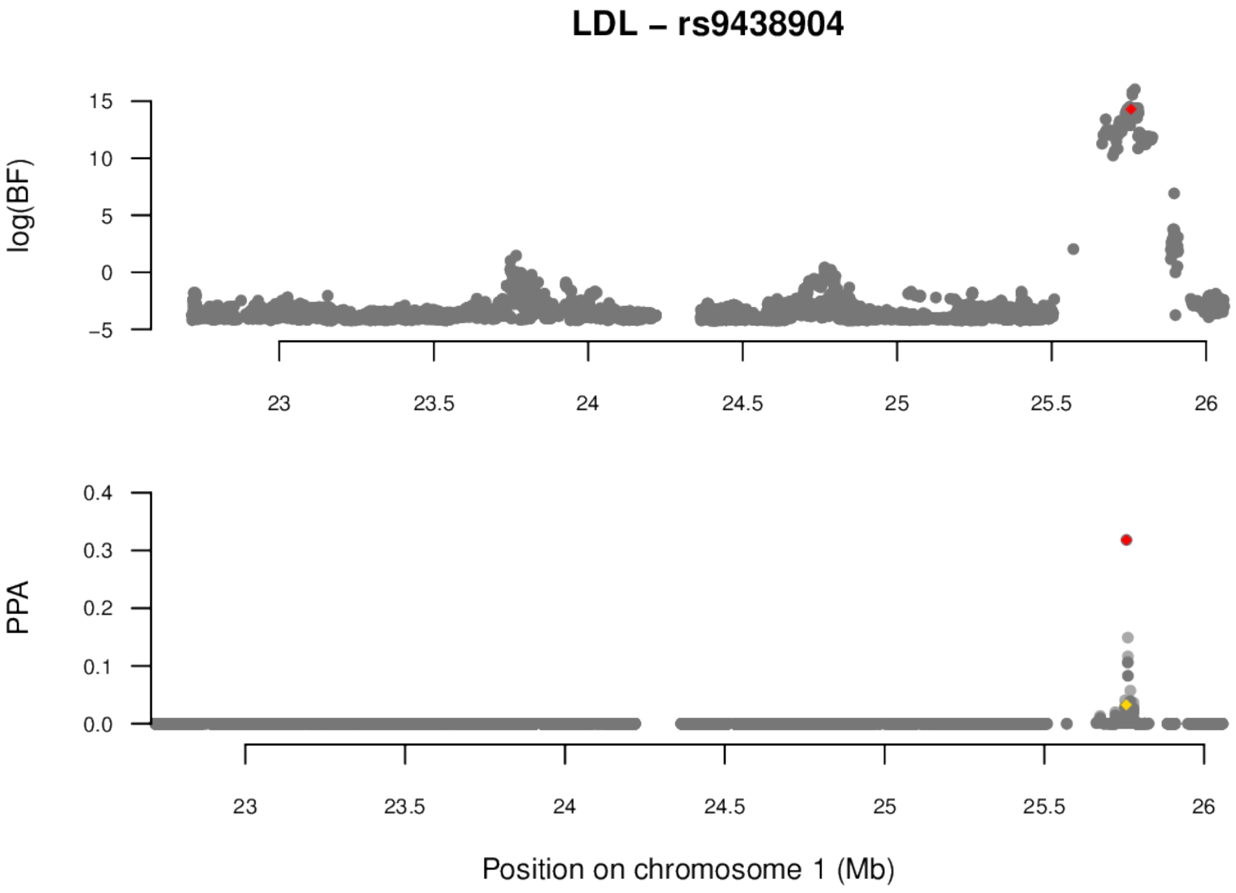
LDL – rs9438904
log(BF)
15
10
5
0
−5
23
23.5
24
24.5
25
25.5
26
PPA
0.4
0.3
0.2
0.1
0.0
23
23.5
24
24.5
25
25.5
26
Position on chromosome 1 (Mb)

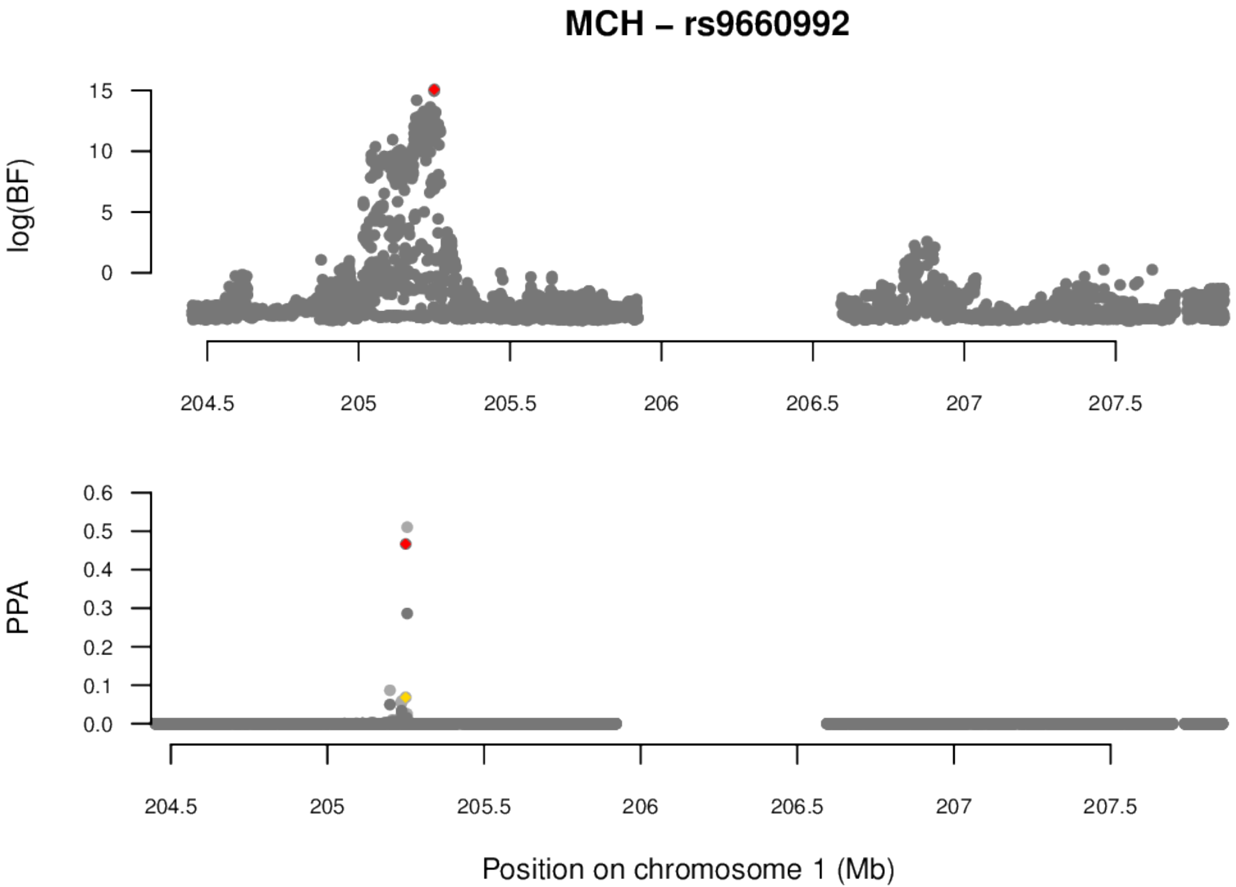

MCH – rs9660992
log(BF)
15
10
5
0
204.5
205
205.5
206
206.5
207
207.5
PPA
0.6
0.5
0.4
0.3
0.2
0.1
0.0
204.5
205
205.5
206
206.5
207
207.5
Position on chromosome 1 (Mb)

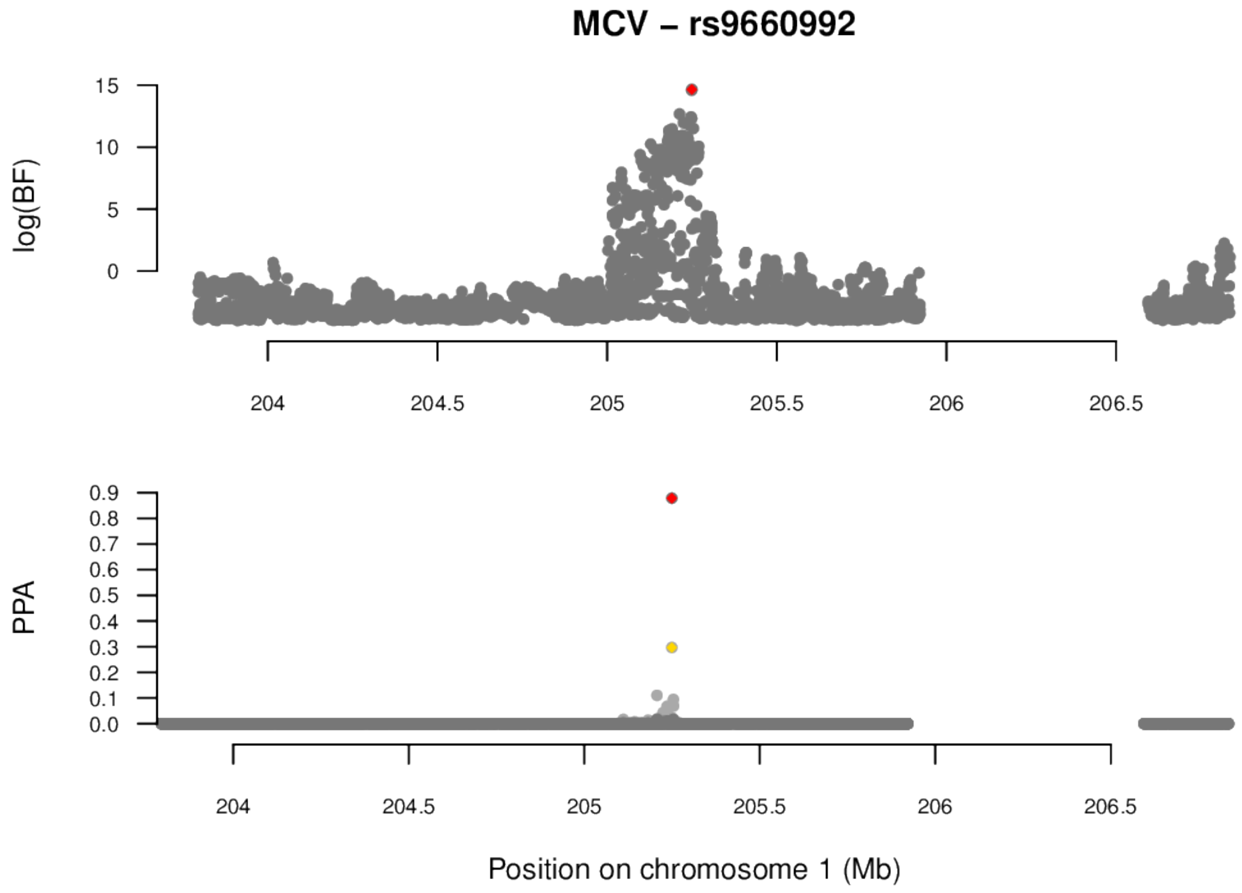

MCV – rs9660992
log(BF)
15
10
5
0
204
204.5
205
205.5
206
206.5
PPA
0.9
0.8
0.7
0.6
0.5
0.4
0.3
0.2
0.1
0.0
204
204.5
205
205.5
206
206.5
Position on chromosome 1 (Mb)

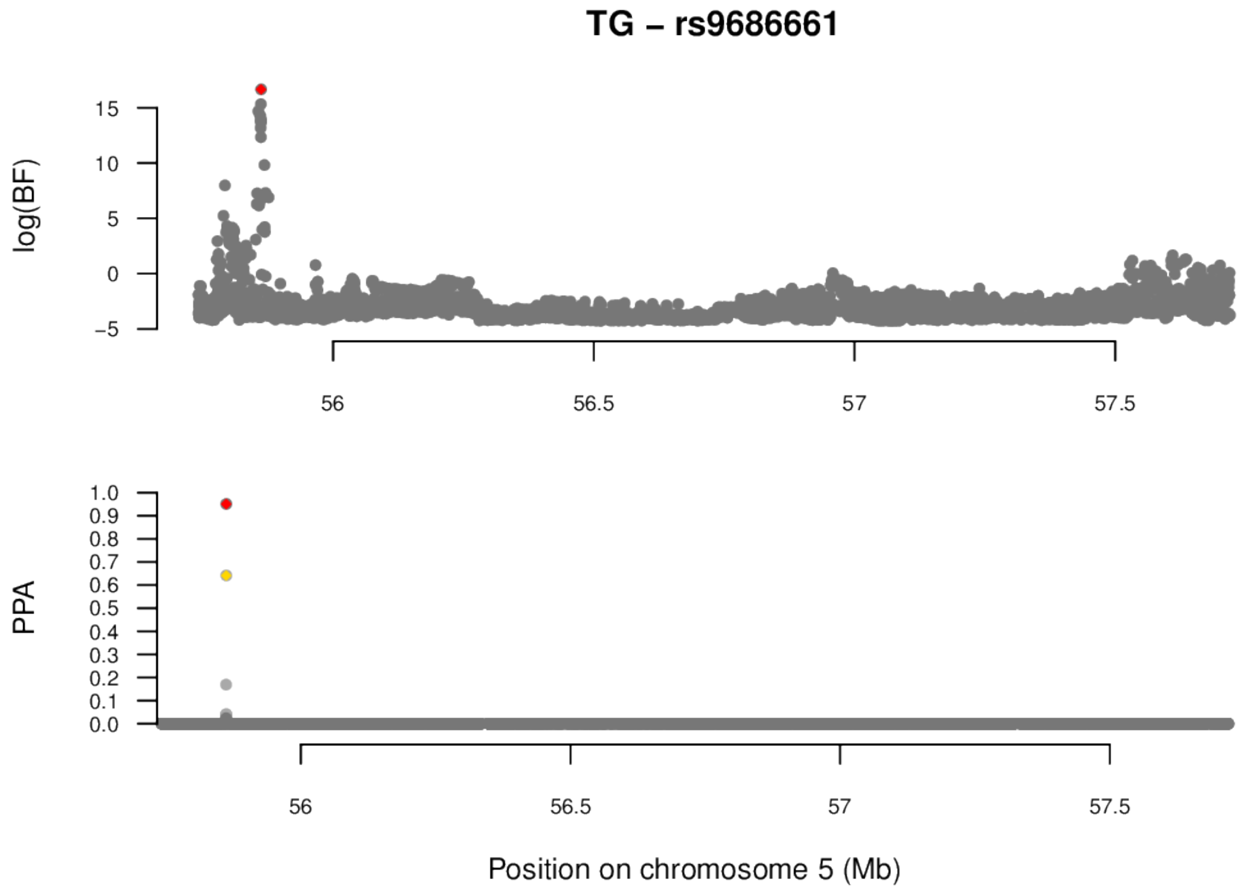

TG – rs9686661
log(BF)
15
10
5
0
−5
56
56.5
57
57.5
PPA
1.0
0.9
0.8
0.7
0.6
0.5
0.4
0.3
0.2
0.1
0.0
56
56.5
57
57.5
Position on chromosome 5 (Mb)

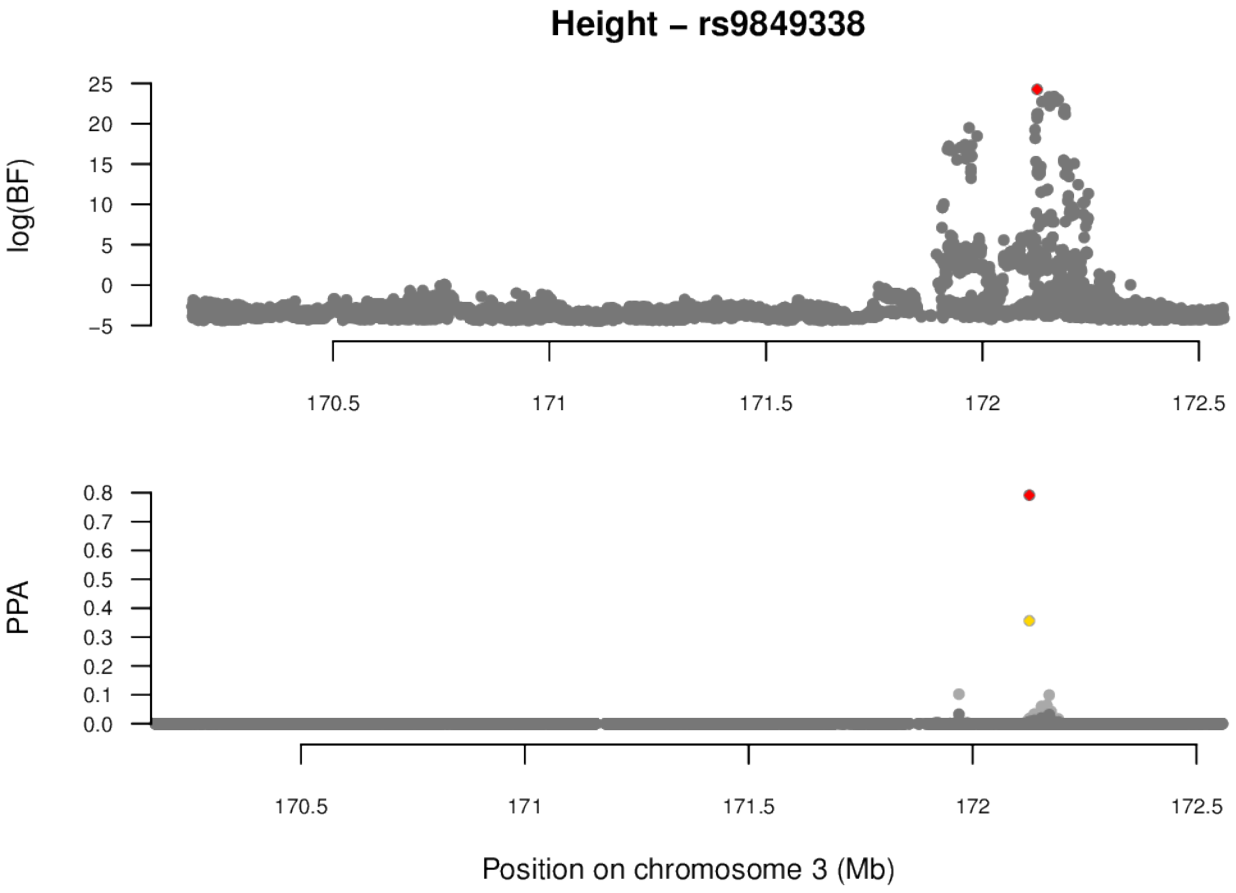

Height – rs9849338
log(BF)
25
20
15
10
5
0
–5
170.5
171
171.5
172
172.5
PPA
0.8
0.7
0.6
0.5
0.4
0.3
0.2
0.1
0.0
170.5
171
171.5
172
172.5
Position on chromosome 3 (Mb)
